# Physiological and molecular responses to drought stress in teak (*Tectona grandis* L.f.)

**DOI:** 10.1371/journal.pone.0221571

**Published:** 2019-09-09

**Authors:** Esteban Galeano, Tarcísio Sales Vasconcelos, Perla Novais de Oliveira, Helaine Carrer

**Affiliations:** Department of Biological Sciences, Luiz de Queiroz College of Agriculture (ESALQ), University of Sao Paulo, Piracicaba, Brazil; University of Vigo, SPAIN

## Abstract

Drought stress is an increasingly common and worrying phenomenon because it causes a loss of production in both agriculture and forestry. Teak is a tropical tree which needs alternating rainy and dry seasons to produce high-quality wood. However, a robust understanding about the physiological characteristics and genes related to drought stress in this species is lacking. Consequently, after applying moderate and severe drought stress to teak seedlings, an infrared gas analyzer (IRGA) was used to measure different parameters in the leaves. Additionally, using the root transcriptome allowed finding and analyzing the expression of several drought-related genes. As a result, in both water deficit treatments a reduction in photosynthesis, transpiration, stomatal conductance and leaf relative water content was found. As well, an increase in free proline levels and intrinsic water use efficiency was found when compared to the control treatment. Furthermore, 977 transcripts from the root contigs showed functional annotation related to drought stress, and of these, *TgTPS1*, *TgDREB1*, *TgAREB1* and *TgPIP1* were selected. The expression analysis of those genes along with *TgHSP1*, *TgHSP2*, *TgHSP3* and *TgBI* (other stress-related genes) showed that with moderate treatment, *TgTPS1*, *TgDREB1*, *TgAREB1*, *TgPIP1*, *TgHSP3* and *TgBI* genes had higher expression than the control treatment, but with severe treatment only *TgTPS1* and *TgDREB1* showed higher expression than the control treatment. At the end, a schematic model for the physiological and molecular strategies under drought stress in teak from this study is provided. In conclusion, these physiological and biochemical adjustments in leaves and genetic changes in roots under severe and prolonged water shortage situations can be a limiting factor for teak plantlets’ growth. Further studies of those genes under different biotic and abiotic stress treatments are needed.

## Introduction

Ecophysiological studies have shown that drought stress is considered the main environmental factor affecting plant growth and productivity worldwide [1]. In addition, the continuous increase of negative impacts caused by drought is expected, particularly in the case of forest species, because climate change is causing longer and more frequent periods of water deficit, reducing the viability of areas for planting trees [2].

By activating specific alterations at physiological, biochemical and molecular levels plants have evolved a number of acclimation responses to face drought stress [3]. Water deficit affects cell turgidity and stomatal aperture of leaves resulting in a decrease of photosynthetic and transpiration rates and an increase in water use efficiency [1]. Consequently, CO_2_ assimilation is severely reduced, restricting leaf metabolism and crop growth [4]. These alterations are highly complex and depend on the stress intensity and extent, as well as the species being studied [5]. Plants can also protect themselves against drought stress by accumulating osmolytes, producing specific enzymes and through enzymatic gene regulation with transcription factors. Proline is one of the most common compatible osmolytes which help plants to maintain their homeostasis under drought conditions [6].

For transcriptional control of plant assimilation to drought stress DREB and AREB appear as the main genes under water deficit. DREBs (Dehydration-Responsive Element-Binding Proteins) belong to the ERF (Ethylene-Responsive Element-Binding Factor) family, which is a subfamily of AP2 / EREBP transcription factors [7]. On the other hand, one of the main routes of response to stress due to water deficit is through the expression of the Abscisic acid Responsive Element Binding proteins (AREBs), transcription factors of the "Leucine Zipper" type (bZIP) subfamily, genes unique to plants [4]. The enzymes, TPS, PIP and BI appear mainly related with drought stress. TPS (Trehalose 6-Phosphate Synthase) is an enzyme that catalyzes the penultimate reaction in trehalose biosynthesis (a carbohydrate formed by two glucose molecules), which acts as an important osmoprotective agent in several species, and helps in drought tolerance and thermal stress [8]. PIP (Protein Intrinsic of Plasma Membrane) is part of the Major Intrinsic Proteins (MIP) family, known as aquaporins, which regulate membrane channels and pores in the passage of water and small neutral solutes such as glycerol, urea, formamide, as well as ammonia and CO_2_ [9]. BI (Bax Inhibitor) is a well-conserved transmembrane protein in plants and animals [10] that serves as a critical mediator of water stress-induced cell death [11]. In wheat, TaBI-1.1 was found to be interacting with the aquaporin TaPIP1, and both were localized in the ER membrane [12]. But in general, the mechanisms by which BI acts under abiotic stress in plants is unclear.

Furthermore, drought stress is a common factor affecting tree plantations and wood production, which is frequently included in tree genetic improvement and breeding programs. Teak (*Tectona grandis* L.f.) (Lamiaceae family) is an important wood source in the tropics, with a significant market around the world. This species has wide and well-performed studies involving irrigation impacts on teak plantation phenology [13], leaf gas exchange in the field [14], molecular diversity [15–18], tissue culture advances [19–21], advanced molecular markers [22], gene structure and function [23–26], transcriptome studies [27], and the first genome examinations [28,29]. In terms of the biochemistry and physiology related to drought stress, some studies have showed the impacts on its development [30–34]. In addition, previous work made the root transcriptome available [35], which allows for the possibility of annotating and find interesting transcripts, and extensively exploring this data.

So far, teak does not have an overview relating physiological, biochemical and molecular responses to drought stress. Thus, the objective of this work was to evaluate the influence of drought stress on different physiological, biochemical and gene expression parameters, in order to generate a more comprehensive view of this process, as well as to propose new hypotheses and give recommendations for this important forest species.

## Materials and methods

### Plant material and experimental conditions

Ten-month-old teak seedlings grown from randomly collected seed at “Areão” Plantation (lat. 22°42'23''S, long. 47°37'7''W, 650 m above sea level) were used. Those seedlings came from an open-pollinated seedlot (~200 seeds) obtained from 20 different trees. This plantation (~1 ha of 12-year-old trees coming from open-pollination) is an experimental field of the “Luiz de Queiroz” College of Agriculture (ESALQ), University of São Paulo, located in Piracicaba, São Paulo State, Brazil. All the seedlings were re-potted in 8 L pots containing soil composed mainly of pine bark. NPK fertilizer (20 g/pot with a 04-14-08 concentration) and Dolomitic Limestone (20 g/pot) were applied before applying drought treatments. Plants were kept in a greenhouse, with a maximum luminosity incidence of 200 μmol of photons m-2 s-1 (at noon), maximum temperature for exhaust fans activation of 27°C, and controlled humidity by automatic water curtain. In all the pots, daily irrigation was performed until complete substrate saturation was achieved. All experiments began in September 2014, when teak seedlings usually reactivate their development.

### Drought treatment

The field capacity of each pot was estimated by the gravimetric method [3]. The soil in each pot was saturated with distilled water. Pot weights were re-calculated after 48 h of drainage and the soil was dried for 24 h at 105°C. The soil moisture content at field capacity was calculated as the difference between the soil weight after drainage and soil weight after drying. Drought stress was initiated on the 20^th^ day after transplanting the teak seedlings. Irrigation volume for each treatment was estimated using the relative humidity mean, value 0.54, as the transformation coefficient for the calculation of the pot weight values and for water content contained in it (S1 File). Drought treatments were defined as moderate drought stress when soil moisture contents decreased by 60% for 30 days, and severe drought stress when soil moisture contents decreased by 60% for 20 days followed by 80% for 10 more days. In addition, the control treatment was defined as daily irrigation maintaining the soil near field capacity. The subsequent experiment was established as a complete randomized design, with five replicates per each treatment and a single plant per pot as a replicate.

### Physiological measurements

**Fig 1:**
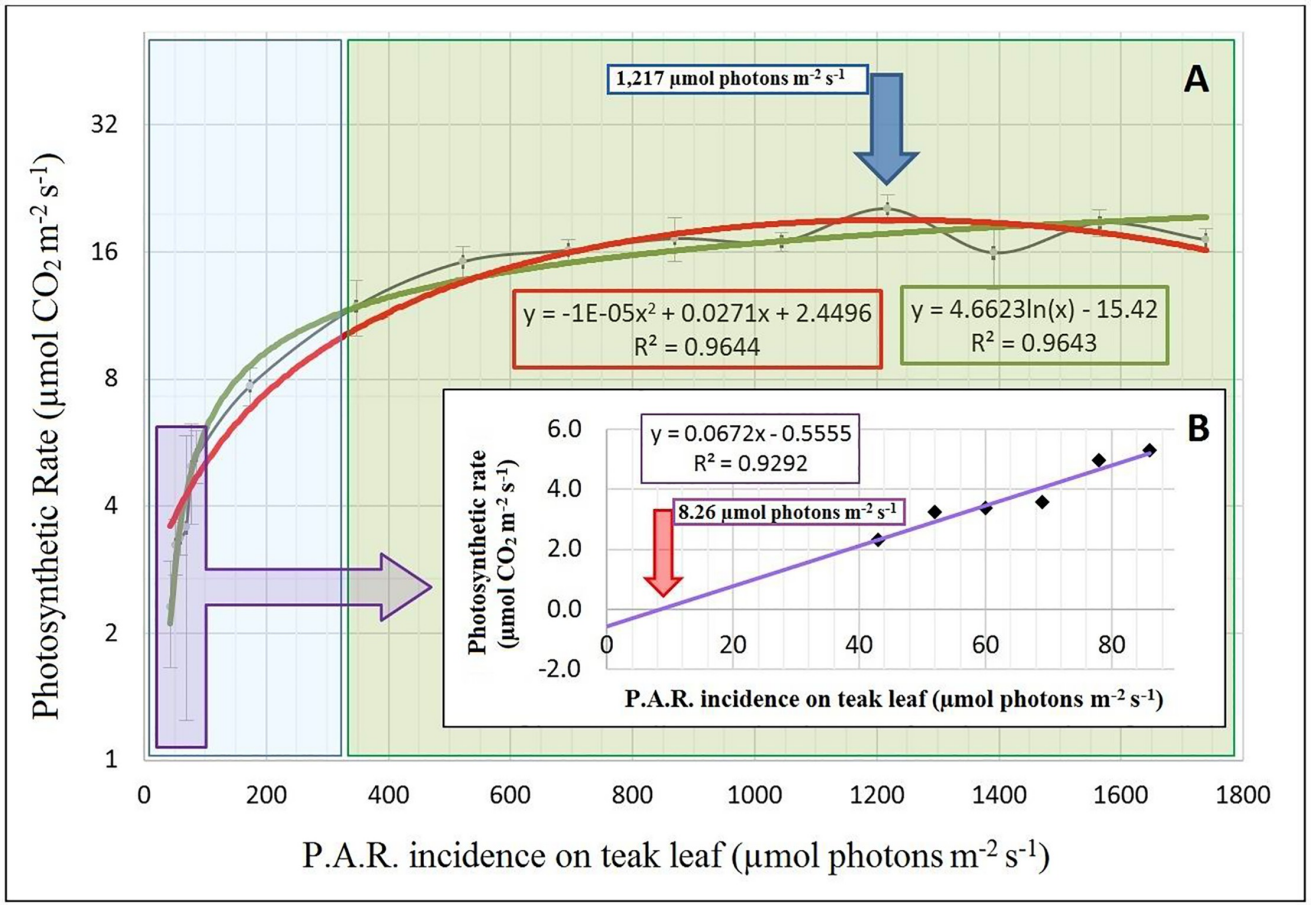
Photosynthetic rate (μmol CO_2_ m^-2^ s^-1^) in response to the Photosynthetically Active Radiation (P.A.R.) increase (μmol m^-2^ s^-1^). All measurements were obtained on the same day and light amount. External graphic (A): Each point represents the mean value of four replicates taken in different plants. The vertical bars correspond to the standard deviation from the mean. The red line is the quadratic tendency of the photosynthetic rate in relation to the irradiance of P.A.R. incident on the leaf (the corresponding equation is in the red square). The green line is the logarithmic trend of the photosynthetic rate relative to the irradiance of P.A.R. incident on the leaf (the corresponding equation is in the green square). The region shaded in blue corresponds to the approximate range of photosynthesis limitation by light in teak. The region shaded in green corresponds to the approximate range of photosynthesis limitation by CO_2_ in teak. The blue arrow indicates the light saturation point (1,217 μmol photons m^-2^ s^-1^ on teak leaf), which is also the irradiance of P.A.R. selected for the subsequent measurements and analyses. Internal graph (B): Photosynthetic response to light only with the linear values of the initial variation range (purple arrow). The purple line is the linear trend of the photosynthetic rate relative to the irradiance of P.A.R. incident on the leaf (the corresponding equation is in the purple square). The red arrow indicates the light compensation point in teak (8.26 μmol photons m^-2^ s^-1^ on teak leaf).

The net CO_2_ assimilation (photosynthesis) rate (A), stomatal conductance to water vapor (gs), transpiration (E), and internal leaf temperature (TI) were measured every two days, on the first pair of fully expanded leaves just below the apex, and with four replications. Water Use Efficiency (WUE) and Intrinsic Water Use Efficiency (IWUE) were calculated using the equations WUE = A/E and IWUE = A/gs, respectively. All leaf gas exchange was measured from 08:00 to 12:00 h (solar time), at 25°C, with a portable infrared CO_2_/H_2_O gas analyzer, IRGA (LCpro-32 070, ADCBioscientific Ltd., Great Amwell, U.K.) equipped with a broad leaf chamber. All measurements were conducted under an artificial saturating light that was determined by a photosynthetic light-response curve (Fig 1). The Photosynthetic Response to Light Intensity was measured on leaves exposed to a CO_2_ concentration of 400 ppm. The increasing Photosynthetically Active Radiation (P.A.R.) was measured under a stepwise process (17 steps) from 0 to 2,000 μmol m^-2^ s^-1^ at 25°C. The Light Compensating Point (LCP) and the Light Saturated Point (LSP) were determined from the graphic.

### Assessment of leaf relative water content

Leaf Relative Water Content (RWC) was measured using the first fully expanded leaf of four plants per treatment. Cut leaves were immediately weighed to obtain a leaf fresh mass (Fresh Weight, FW) and then leaves were immersed in deionized water in a petri dish and incubated overnight in the dark, below 8°C. Leaves were re-weighed to obtain Turgid Weight (TW). Next, leaves were dried for 2 days in an oven at 60°C and weighed again to determine the Dry Weight (DW). Finally, RWC was calculated as [(FW- DW) × (TW—DW) -1] × 100 according to Turner methodology [36].

### Measurements of leaf proline content

Total free proline was estimated using a modified method described by Bates and collaborators in 1973 [37]. About 1 g of frozen leaf sample was homogenized with 10 mL of 3% (*w/v*) sulphosalicylic acid. The homogenate was centrifuged and filtered through filter paper. The reaction mixture, which contained 2 mL of homogenate with 2 mL glacial acetic acid and 2 mL ninhydrin reagent was boiled for 60 minutes and then cooled on ice for 10 minutes. After cooling, 4 mL of toluene was added to the reaction mixture, followed by vigorous stirring for extraction of the chromophore in the supernatant phase. The absorbance of red color was read at 520 nm against toluene blank on UV–visible spectrophotometer (Amersham Bioscience, Ultrospec 2100 pro). Proline concentration was determined using a calibration curve and expressed as μg proline g^−1^ FW.

### RNA extraction and cDNA synthesis

Roots were harvested from seedlings with moderate and severe drought treatments and control plants (three replicates used per treatment). All substrate attached to the root system was carefully removed. Later, they were macerated in liquid nitrogen and 100 mg were used for RNA extraction. Total RNA was extracted using the TRIzol® commercial reagent (Invitrogen) following the manufacturer’s instructions. Samples were quantified using NanoDrop (Thermo Scientific). Since teak roots have several polyphenols, it was necessary to perform an additional cleaning of the RNA using the Oligotex Kit (Qiagen) for Purification of Poly(A)+ mRNA from total RNA using spin columns, following the manufacturer’s instructions. Then, 2 μg of total RNA from each sample were treated with DNAse I (Promega) and analyzed in agarose gels (to see RNA quality and DNA absence). Finally, nine cDNA samples (moderate, severe, and control) were synthesized with 1.0 μg of the treated RNA using the SuperScript ^™^ III First-Strand Synthesis System for RT-PCR (Invitrogen), following the manufacturer's instructions. The cDNA synthesis step was performed using an oligo dT primer.

### Transcriptome and subtractive gene selection

The teak transcriptome raw data previously obtained and described [35] was used for this study, which is referred to the Bioproject PRJNA287604 at the NCBI. Since the water stress that was applied in this experiment consisted of diminishing the availability of water in the soil, our interest was to explore the expression of widely known genes that respond to water stress in teak roots. The teak root transcript library (BioSample #SAMN03784593, SRA #SRX1074857 and Run #SRR2080155) was used for the de novo assembly of the root transcriptome using the Trinity program, version 2013 [38,39]. Then, it was annotated using the Blast2Go program [40] with the default parameters. Sequences that did not show homology were discarded and the remaining ones were submitted to the “Gene Ontology-GO” tool for functional annotations. Then, selection of transcripts related to drought stress was achieved by performing a search over the annotated genes using the “Response to stress” (0006950) and “Response to water deprivation” (0009414) GO terms. Later, for each gene selected, the related transcripts were obtained, and from this list, the one with the lowest e-value, highest similarity, largest nucleotide sequence size and the translated protein with the lowest number of stop codons was chosen. Then, BlastX and amino acid alignment using Clustal Omega Program, (http://www.ebi.ac.uk/) were performed to identify conserved regions of high similarity between *Tectona grandis*, *Populus trichocarpa*, *Theobroma cacao*, *Arabidopsis thaliana*, *Solanum lycopersicum*, *Brassica napus* and *Morus notabilis*. Finally, the “KEGG” database was used to obtain metabolic pathways.

### Gene expression analysis by quantitative real-time PCR

The qRT-PCR primers were designed flanking *TgTPS1*, *TgAREB1*, *TgDREB1* and *TgPIP1* teak sequences (S2 File). The qRT-PCR primers for *TgHSP1*, *TgHSP2*, *TgHSP3* and *TgBI* were obtained from previous teak expression studies [35]. The melting curve was obtained for all the primers (S3 File). The qRT-PCR mixture contained 125 ng of cDNA from each sample, primers to a final concentration of 50 μM each, 12.5 μl of the SYBR Green PCR Master Mix (Applied Biosystems, USA) and PCR-grade water up to a total volume of 25 μl. Each gene reaction was performed with three technical replicates for each treatment, with a total of 27 reactions. Also, PCR reactions without template were used as negative controls for each primer pair. The StepOnePlus^™^ System (Applied Biosystems, USA) was employed for the qRT-PCR, and the reactions were performed as follows: 2 min at 50°C, 2 min at 95°C, and 45 cycles of 15 s at 95°C and 1 min at 65°C in 96-well optical reaction plates (Applied Biosystems, USA). The control treatment was used as a calibrator to normalize the values between different plates. *TgEF1α* was used as the control gene following previous teak gene expression studies [35].

### Statistical analysis

The data normality was verified by the Lilliefors test. The variances homogeneity was observed by the Bartlett test. The analysis of the variance was performed through F tests (for data satisfying all ANOVA assumptions) and Kruskal-Wallis (non-parametric test). Tukey's test was used to analyze the ANOVA means comparison. Dunnett's bilateral test was used to evaluate the differences between the control and the other treatments. To perform a stepwise multiple comparison method (as a post hoc analysis), the Student-Newman-Keuls (SNK) test was performed. All analyzes were performed with the “Assistat” program (7.7 beta version), “Action” (2.8.29.357.515 version) supported in the R environment (3.0.2 version), and Microsoft Excel. All tests were run with a 95% confidence level.

## Results

### Light-response measurements

The light saturation curve of photosynthesis was recorded and adjusted giving information about rate of maximal oxygen, respiration rates, and the light compensation point (Fig 1). Net photosynthetic rate increased rapidly when P.A.R. increased from zero to 400 μmol m^-2^ s^-1^, which is the range of photosynthesis limitation by light in teak. The light compensation point (when the photosynthesis rate is 0) was 8.26 μmol of photons m^-2^ s^-1^ on a teak leaf (red arrow, Fig 1). The light saturation point of 1,217 μmol of photons m^-2^ s^-1^ on a teak leaf (blue arrow, Fig 1), produced a carbon assimilation around 21.93 μmol of CO_2_ m^-2^ s^-1^. A logarithmic trend curve (natural logarithm), which resembles the rectangular hyperbolic distribution, was the one that best adjusts to the variation of the photosynthetic rate that was deduced in this study (green line and equation in green square, Fig 1) following previous work [41]. The R^2^ index of the two functions (quadratic and logarithmic, red and green lines in Fig 1, respectively) showed that both fitted the data (R^2^> 0.96, for both equations, red and green squares, Fig 1), and both of them showed appropriate overlap, indicating a low discrepancy between the two study models.

### The effect of drought stress on photosynthetic and transpiration rate, stomatal conductance, water use efficiency and leaf temperature in teak

**Fig 2:**
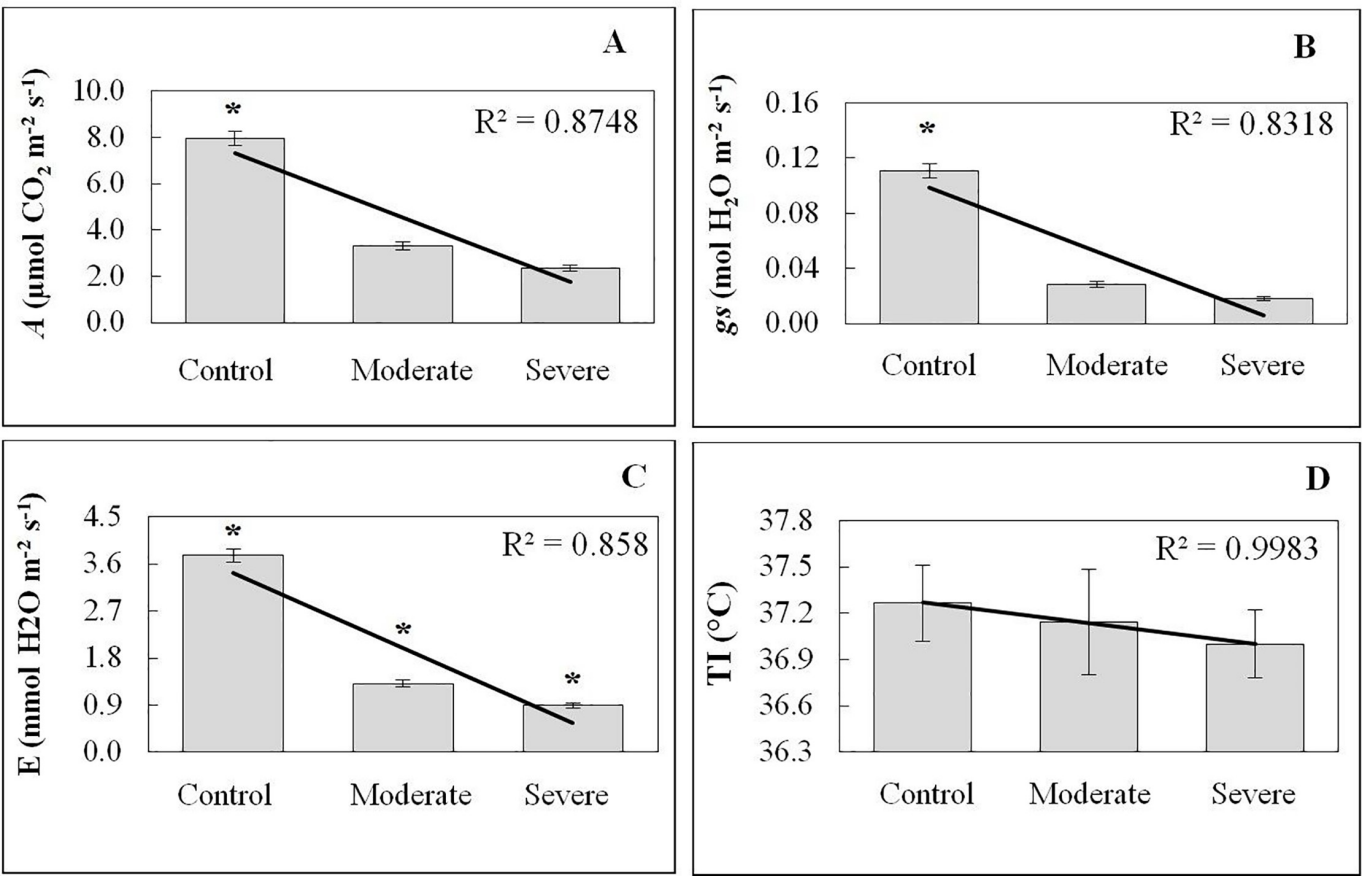
Photosynthetic leaf gas exchange characteristics of teak plants under different stress treatments (moderate and severe). A) Photosynthetic Rate, *A* (μmol CO_2_ m^-2^ s^-1^). B) Stomatal conductance, *gs* (mol H_2_O m^-2^ s^-1^). C) Transpiration, *E* (mmol H_2_O m^-2^ s^-1^). D) Leaf temperature, *TI* (°C)_._ Bars represent the standard deviations of the means from five replicates taken in different plants. * refers to the significant value at 0.01 probability, t-test.

**Fig 3:**
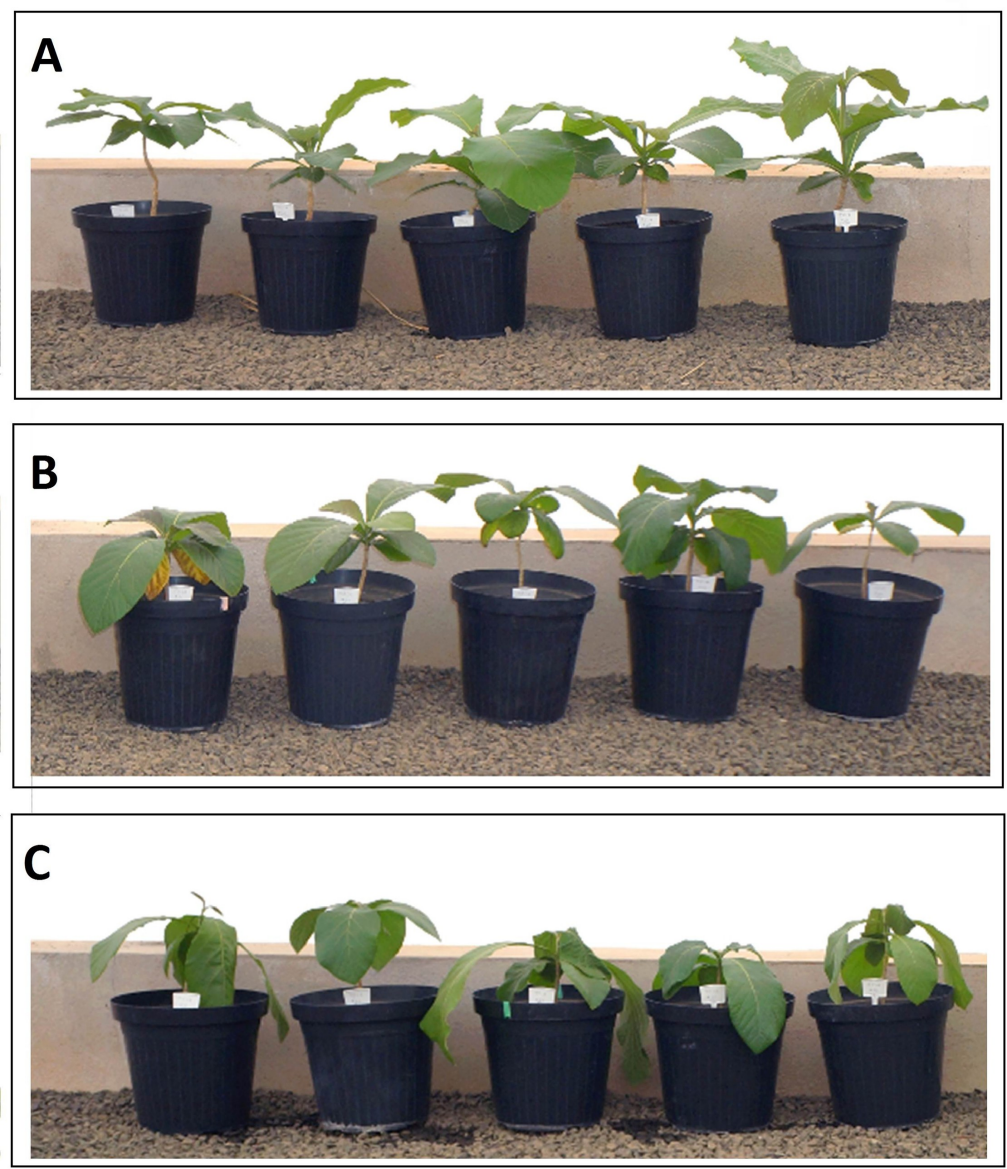
Effect of drought stress on teak vegetative growth in the greenhouse. A) Control plants. B). Plants under stress due to moderate water deficit (20 days). C) Plants under stress due to severe water deficit (30 days).

Photosynthetic rate (A), Stomatal conductance (gs) and Transpiration (E) over the course of the experiment were essentially similar in full irrigation (Fig 2). After water deficit, A significantly decreased (p<0.001, S4 File) by 59% and 71% for moderate and severe drought stress, respectively (Fig 2A), when comparing to control plants. Decreases in A were accompanied by sharp decreases in gs (p<0.001, S4 File), ranging from 75% in moderate drought stress to 84% in severe drought stress (Fig 2B). In parallel with the A and gs decreases, remarkable reductions (p<0.001, S5 File) in E were observed in water-stressed plants for both treatments (Fig 2C). In general, the stomatal conductance variations were more intense when comparing with the transpiration rate data (S6 File). For moderate drought stress, the transpiration was about 64.79% lower than the control plants, while for the severe drought stress, the reduction was 76.56%. The water use efficiency (WUE) means increased from the plants without drought stress to the ones under moderate and severe treatment (2.16, 2.42 and 2.54 μmol CO_2_ m^-2^ s^-1^ / mmol of H_2_O m^-2^ s^-1^), but with no statistical difference between them (S7 File). The intrinsic water use efficiency (IWUE) showed a statistical difference between the control and drought treatments, with means of 0.007 (control), 0.115 (moderate) and 0.132 (severe) μmol CO_2_ m^-2^ s^-1^ / mmol of H_2_O m^-2^ s^-1^ (S7 File). Leaf temperature (TI) did not show statistical differences between the treatments (Control = 37.26 ±0.25°C, Moderate = 37.14±0.34°C, Severe = 37.0±0.22°C) (S8 File, Fig 2D). Acclimation responses under water stress included a decrease of evaporative areas and low CO_2_ availability caused by lower stomatal conductance, which later induced a decrease in the carbon fixation rate. Plants subjected to severe drought stress had a much more intense developmental reduction with weak leaves than those subjected to moderate drought stress (and the control), as can be observed in Fig 3.

### The effect of drought stress on relative water and proline content in teak leaves

**Fig 4:**
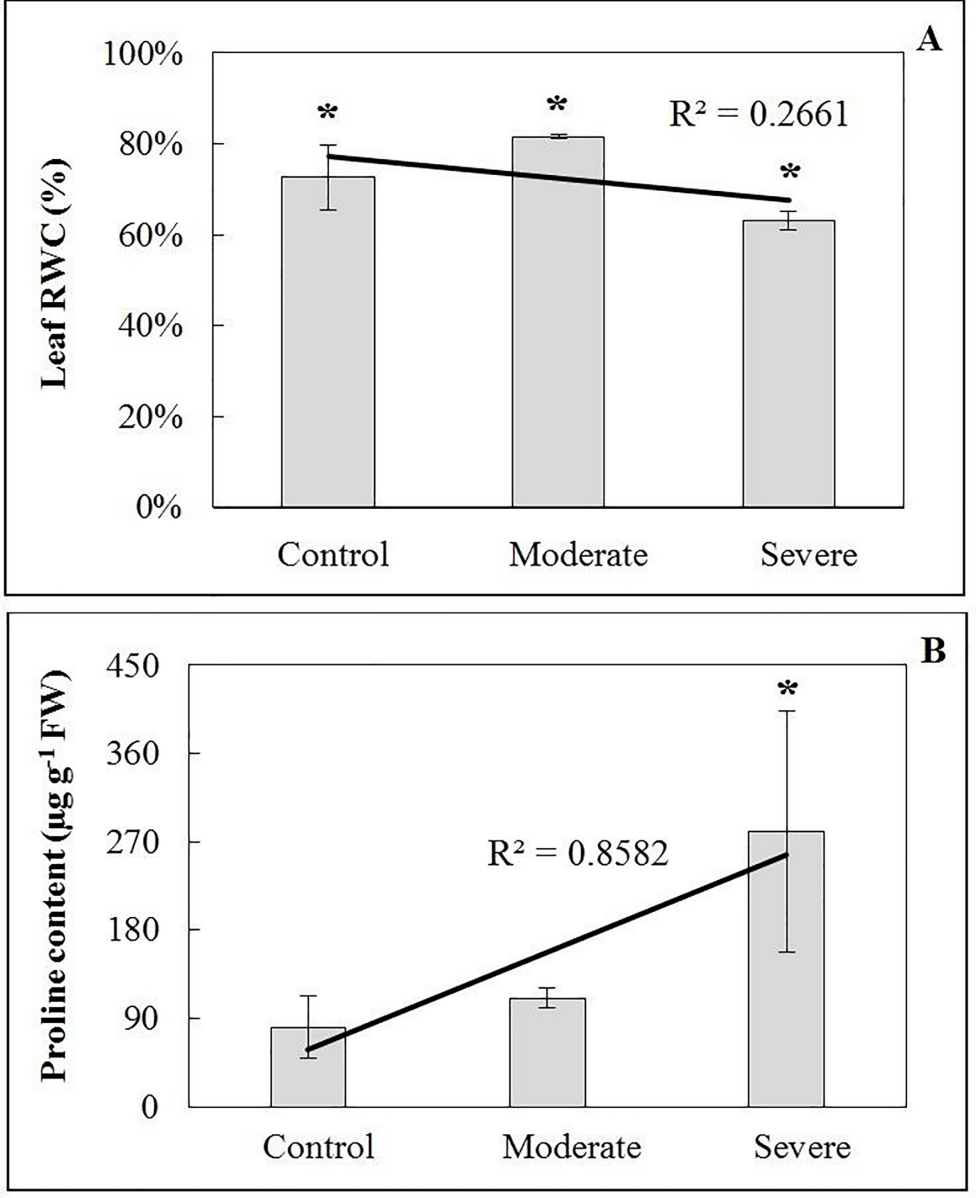
Leaf relative water and proline content under drought stress. **A**) Effect of drought stress on leaf relative water content (%). B) Leaf proline content (μg g^-1^ Fresh Weight of leaves) of teak plants under well-watered and water-stressed conditions (moderate and severe). Bars represent the standard deviations from the means for five replicates taken in different plants. * refers to the significant value at 0.01 probability, t-test.

According to the Tukey test (P<0.05), RWC showed significant differences between treatments of drought stress (S9 File). Variations can be seen in Fig 4A. Control plants had 11% lower RWC than those subjected to moderate drought stress, with an average value of 81.6±0.47%. Plants under severe drought stress showed a more remarkably reduction with a minimum of 52.07%, a maximum of 78.51% and a mean of 63.05±1.96%. The leaf proline content in the moderate and severe treatments was higher when compared to the control plants (S10 File); an increase of 36.5% was observed in the moderate drought stress plants, whereas an even higher increase of 347% was observed in response to severe drought stress when compared to the control (Fig 4B).

### Subtractive gene selection using root contigs

Trinity program assembled 132.647 contigs from the root raw data available at NCBI (SRR2080155). Later, Blast2Go was applied in the contigs and showed the highest sequence number for cellular and metabolic processes (for biological processes), binding and catalytic activity (for molecular function) and cell and organelle localization (for the cellular component) (S11 File). After searching with the GO terms “Response to stress” and “Response to water deprivation”, 977 genes related to water deficit stress were found (S12 File) and classified into 63 metabolic maps, with emphasis on glycerolipids metabolism (8 enzymes and 30 sequences), galactose pathway (6 enzymes and 23 sequences), glycolysis/gluconeogenesis (6 enzymes and 17 sequences), phenylalanine metabolism (3 enzymes and 24 sequences) and starch and sucrose synthesis (4 enzymes and 24 sequences) (S13 File). Following the identified sequences, four genes were selected (S14 File). The first gene is the *Trehalose 6-phosphate Synthase* (*TgTPS1*, comp49883_c0_seq1), with 1668 bp and 556 amino acids, which is related to the trehalose biosynthesis, an important carbohydrate with osmoregulatory function. In addition, above the TgTPS1 protein sequence, the "Glycosyl transferase 20" domain, covering a region of 244 amino acids, and the "Trehalose-phosphatase" domain, covering 236 amino acids were identified. The second gene is a water deficit stress-related transcription factor called *DREB* (*TgDREB1*, comp1924_c0_seq1), which is activated by cell dehydration. TgDREB1 has 417 bp and 139 amino acids, and the bZIP domain was identified in the protein sequence, spanning a central region of 33 amino acids. The third gene is another transcription factor called *AREB* (*TgAREB1*, comp32065_c11_seq6), activated by the presence of ABA. TgAREB1 has 756 bp and 252 amino acids, and the domain "AP2—Apetala2" was identified in its protein sequence, positioned in the first 49 amino acids. The fourth and last gene is a plasma membrane aquaporin (*TgPIP1*, comp30227_c3_seq10), with 287 amino acids and 861 bp, related to the entry of water molecules and other substances into the cell. In TgPIP1, the protein domain "Major Intrinsic Protein" was identified, covering a central region of 230 amino acids. Finally, the four genes of this study showed significant identity with their homologs of poplar, cocoa, arabidopsis, tomato, rapeseed and blackberry using BlastX. Also, they showed a high conservation of the residues in the regions of the protein domains using Clustal omega (S15 File).

### Expression of key genes involved in drought tolerance in teak roots: *TgTPS1*, *TgDREB1*, *TgAREB1*, *TgPIP1*, *TgHSPs*, *TgBI*.

**Fig 5:**
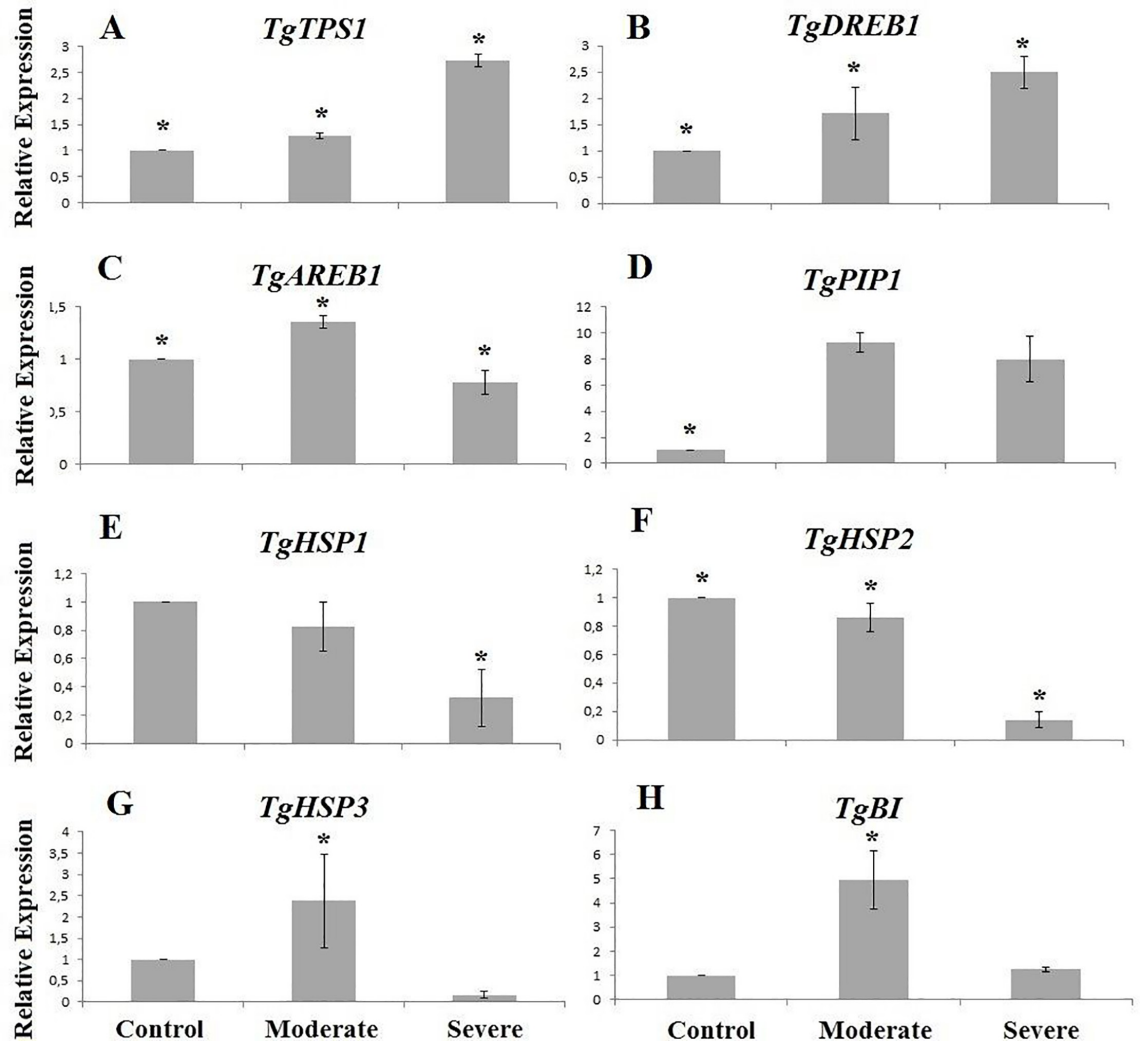
Relative expression of drought stress genes in teak. A) *TgTPS1* (Trehalose 6-Phosphate Synthase), B) *TgDREB1* (Dehydration-Responsive Element-binding Protein), C) *TgAREB1* (ABA Responsive Element Binding Protein), D) *TgPIP1* (Protein Intrinsic of Plasma Membrane), E) *TgHSP1* (Heat shock protein), F) *TgHSP2* (Heat shock protein), G) *TgHSP3* (Heat shock protein), H) *TgBI* (Bax inhibitor) in teak under different drought stress treatments. Control treatment was used as a calibrator, and *TgEF1α* (Elongation Factor) was used as the control gene. Bars represent the standard deviations from the means from three technical replicates for each treatment. * refers to the significant value at 0.01 probability, t-test.

A pair of primers above each sequence was prepared to determine the level of expression of these genes using the quantitative real-time PCR (S2 File). In general, all genes showed statistical differences between treatments at a 95% confidence level (S16 File). It was observed that for the *TgTPS1*, *TgDREB1*, *TgAREB1*, *TgPIP1* transcripts (chosen from the teak root transcriptome databases), the gene expression of the control plants was always lower when compared to the plants with moderate or severe water stress treatments. In the case of the *TgTPS1* and *TgDREB1* genes, when the stress treatment was changed from moderate to severe, these genes increased expression 2.5 times when compared to the control (Fig 5A and 5B). Differently, the *TgAREB1* and *TgPIP1* genes increased expression with moderate stress at 1.5 and 10 times, when compared to the control, respectively (Fig 5C and 5D). Then, when increasing the drought stress treatment to severe, the *TgAREB1* gene decreased in 1 unit its relative gene expression when compared to moderate. The *TgPIP1* gene in severe stress continued with an expression of 8 times greater when compared to the control. In addition to the genes found in the transcriptome involved with water stress, we went further when evaluating the gene expression of previous stress-related genes in teak [35]. Consequently, the *TgHSP1*, *TgHSP2*, *TgHSP3*, *TgBI* genes were analyzed, finding that the *TgHSP1* and *TgHSP2* genes showed lower expression with the water stress treatments (moderate and severe) when compared to the control (Fig 5E and 5F). However, the *TgHSP3* and *TgBI* genes showed up twice of the expression in the moderate stress treatment when compared to the control, but then with severe stress these genes reduced their expression to the same control values (Fig 5G and 5H).

## Discussion

### The teak response to light

In forest stands, the direct solar irradiance of P.A.R. can reach 2,000 μmol of photons m^-2^ s^-1^ at the top of the canopy, and remains at about 10 μmol of photons m^-2^ s^-1^ on the forest floor [42]. The results obtained by the photosynthetic light response curve in this study are in accordance with the teak pioneering habit, which does not develop properly under filtered light and can still be considered a heliophytic (sun-tolerant) plant. One parameter commonly related to the ecophysiological characteristics of plant photosynthesis is the light compensation point. This parameter determines the irradiance of P.A.R. capable of balancing the CO_2_ rates emitted by mitochondrial respiration with those absorbed by photosynthesis [43]. In heliophytic plants (such as teak), the light compensation point tends to be obtained with 10 to 20 μmol of photons m^-2^ s^-1^, whereas for sciophytic plants, it is between 1 and 5 μmol of photons m^- 2^ s^-1^ [44]. It was not possible to determine the precise light compensation point of teak, since P.A.R. irradiance values greater than 50 μmol of photons m^-2^ s^-1^ were used for the construction of the photosynthetic light response curve. Thus, the initial values of the photosynthetic rate variation were used to graph a linear relation and to obtain an estimated value of the light compensation point of Γ = 8.26 μmol of photons m^-2^ s^-1^ (R^2^ = 0.92) (red arrow, Fig 1). The value of the light compensation point for teak is lower when compared to the one reported for citrus, with 12 μmol of photons m^-2^ s^-1^ [45]. Also, the apparent quantum efficiency of teak was obtained, with a value of Φ = 0.0672 μmol of CO_2_ x μmol of photons-1 (constant from equation in purple square, Fig 1), which is close to the value of other species such as *Pilea microphyla* (Φ = 0.065), *Araucaria heterophyla* (Φ = 0.079), *Citrus spp*. (Φ = 0.03 to Φ = 0.05), *Picea abies* (Φ = 0.084), *Betula pendula* (Φ = 0.09) and *Ginkgo biloba* (Φ = 0.092), being an average for C3 plants of Φ = 0.0891 ± 0.0009 [46]. To obtain the most significant physiological profile for teak under drought stress, the P.A.R. value of 1,217 μmol of photons m^-2^ s^-1^ in the leaf (light saturation point) was chosen for the next measurements.

### Physiological adjustments under drought stress in teak

Plant responses to changing water conditions are highly complex, and cover many aspects including changes at the genetic molecular level, as well as in biochemical and physiological processes. The irradiance of 1,217 μmol of photons m^-2^ s^-1^ showed a mean photosynthetic rate of 8.69 μmol CO_2_ m^-2^ s^-1^. This value is similar to study results in *Pinus ponderosa* [47], where the average value of 8 μmol CO_2_ m^-2^ s^-1^ was obtained, and higher than those found in *Tabebuia aurea* [48], where maximum photosynthesis reached 9.9 μmol CO_2_ m^-2^ s^-1^, both under treatment without water deficit. Since the intensity of 1,217 μmol of photons m^-2^ s^-1^ corresponds to the maximum peak of the photosynthetic rate, it means that the metabolic energy of teak was altered due to water stress treatments and resulted in a photosynthetic rate reduction, as shown in Fig 2A. Photosynthesis is a key process of primary metabolism that is affected by water deficit [1]. Photosynthesis can also be affected by lower CO_2_ availability, stomatal closure and lower mesophyll conductance, as well as other non-stomatal limitations [49]. The relative impact of those limitations varies gradually with water stress intensity, persistence duration, and species [5,50]. It has been demonstrated that under mild or moderate drought stress, the leaf internal CO_2_ concentration is the major reason for reduced leaf photosynthetic rates, while for severe drought stress, biochemical limitations, and the damaging of photosynthetic apparatuses inhibits the photosynthetic process [51,52].

At the same time, stomatal conductance and transpiration decreased in all treatments when they were exposed to drought stress (Fig 2B and 2C). One of the first plant responses to drought is stomatal closure, restricting gas exchange between the atmosphere and the inside of the leaf [53]. Stomata are structures located in the leaves with the capacity to establish communication and gas exchange between the leaf mesophyll and the environment, being constituted by two guard cells, subsidiary cells and an ostiole (slit or stomatal pore) [43]. In orange trees under drought stress, a strong reduction in the transpiration rate, photosynthesis and stomatal conductance was observed [54]. Besides, drought stress decreased stomatal conductance, photosynthesis and transpiration rates in five species of eucalyptus (*E*. *grandis*, *E*. *urophylla*, *E*. *camaldulensis*, *E*. *torelliana*, and *E*. *phaeotrica*) [55]. The behavior of the transpiration rate and stomatal conductance results are in agreement with those previously obtained for teak [14,32]. These researchers observed a significant drop in transpiration, stomatal conductance and photosynthesis in trees submitted to drought stress; however, the values were completely restored after an irrigation shift. Higher values of A are usually accompanied by higher rates of E and gs for several species in the tropical region [56]. However, the maintenance of the photosynthetic rates associated with lower values of gs and E under conditions of water stress, indicates the existence of adaptation mechanisms. According to Chaves and Oliveira (2004), the initial phase of water deficit is the stomata partial closure, observed through a fast reduction of the stomatal conductance. Then, a later phase can occur with a decrease in the transpiration rate and photosynthetic carbon assimilation, which both causes an elevation in the water use efficiency and intrinsic water use efficiency, in order to diminish water loss [51].

In our study, IWUE of teak plants under severe stress increased by double when compared to control, with statistical significance (S7 File), which means a tolerance under water deficit with direct control of the stomatal opening. It should be noted that the lower the water availability, the greater the degree of stomatal closure to reduce water loss [57]. Consequently, WUE increases and succeeds to maintain the minimum water balance [43]. Moreover, the guard cell is able to keep the stoma open by reducing the water potential and increasing the solute potential, usually by increasing proline in the leaf [58]. Usually, this osmotic adjustment under drought conditions is the main factor contributing to maintain the highest transpiration efficiency [59,60].

In addition, we observed in this study a significant phenotypic reduction in the turgidity, strength and position of the leaves under severe drought stress (Fig 3C). A 40% reduction in teak growth under stress was observed in previous studies [14]. With this, it is possible to argue that teak is a tolerant species to drought stress but taking into account that teak plantings should be avoided in regions with a history of prolonged drought, under the risk of low plant growth rates and a decrease in productivity.

### Water dynamics and osmoprotection under drought stress in teak: Physiological and molecular insights

Relative water content (RWC), an indicator of turgidity, provides a measure of the plant water status. It is usually used as an index of dehydration in most plants [61]. Exposure of teak plants to drought conditions significantly altered relative water content. Relative water content was reduced by ±9.57% in response to severe drought stress (Fig 4A). It was found that teak trees without 20 days of irrigation showed a reduction of about 50% in RWC in relation to trees with full water availability [33]; this value was quickly recovered after one irrigation shift. In *Hevea brasiliensis* seedlings, there was a 20% decrease in RWC after 9 days without irrigation, when compared to control treatment; this decrease was positively correlated with malondialdehyde (MDA) increase, which is a toxic product of cell lipid peroxidation and, in turn, positively correlated with the increase of reactive oxygen species (ROS) [62]. In *Populus deltoides x Populus nigra* hybrids, it was shown that significant RWC variation existed between clones droughted to 30% of field capacity, and those droughted to 40% and 50% of field capacity [63]. In the same study, a decrease in growth vigor and an increase in the antioxidant enzymes activity (SOD—Superoxide dismutase; APX—Ascorbate peroxidase; POD—Peroxidase; and CAT—Catalase) were observed. Here, the RWC intensification under moderate stress treatment compared with control treatment can be explained by the high transpiration rate and high maximum temperature at the collection date (38°C), which may have caused a leaf transpiration outbreak during tissue preparation and photographing. This also explains the great RWC variability in the control plants, since both moderate and severe drought stress plants remained with their stomata closed during the collection day, and therefore the transpiration rate was less influenced by the air temperature and humidity [64]. Also, the RWC reduction can be positively correlated with the photosynthetic rate reduction and with the compatible solutes enhancement [65]. Regarding the Proline content, moderate and severe drought stress showed 1.3 and 3.5 times more of this amino acid when compared with the control (Fig 4B). The accumulation of osmoprotectants such as proline in plants under drought stress conditions have been reported to increase the osmotic adjustment capability, resulting in a stronger drought tolerance [66]. Previous studies of teak drought stress showed a significant increase of 220% in leaf proline content after 9 days of drought stress, with an increase in the soluble carbohydrate content, by 323% [67]. Two distinct teak clones showed increases in leaf proline content of 82% and 87% after 20 days without water [33]. Thus, mechanisms of drought stress tolerance in teak could have great variability among individuals, which can be explained by the genetic variation influenced by the open pollinated crosses that bring individuals that are more tolerant or susceptible to drought stress.

On the other hand, our work showed that in roots an osmotic adjustment mechanism occurs with a lack of water, which is helped by lipid- and carbohydrate-related genes and proteins, such as PIP (related to leaf water regulation) and TPS, HSP and BI (related to Osmoprotection). *TgTPS1*, *TgPIP1* and *TgBI* showed an appropriate performance and favorable activation in gene expression under severe drought stress (Fig 5), but the *TgPIP1* gene stood out more than the other transcripts, with 9-fold higher expression when compared to the control. *TgHSP3* and *TgBI* showed higher expression in moderate stress when compared to control, but they did not have good performance in severe drought.

Aquaporins play a key role in hydraulic conductivity dynamics between roots, stem and leaves, especially in situations of environmental changes, favoring a better water use efficiency and water distribution between plant organs and tissues [68]. Studies have shown a marked expression regulation of *PIPs* by abscisic acid induction. In rice, relevant aquaporin genes were found from the root transcriptome with down- and upregulation by water deficit [69], a strong induction of several *PIPs* and a high ABA production in roots in response to water deficit [70]. *EgTIP2* was also involved in eucalyptus’ response to drought and was down-regulated by abscisic acid [71]. *PIP* gene expression is usually more abundant in roots than in leaves and stems [72]. In addition, transgenic tobacco and Arabidopsis plants with *PIP* inhibition showed lower water potential in the leaves in relation to control under drought stress [73]. Heterologous expression of *PIP* genes in model plants have shown drought stress tolerance by maintaining osmotic balance [72,74]. Additionally, *PIPs* expression can be altered by other stresses such as salinity, cold and lack of nutrients and oxygen (flooding) [75,76].

The *TPS* gene has been frequently highlighted for its action against drought stress. Plants overexpressing the *TPS* gene have shown an increase in tolerance to drought stress without significant phenotypic alterations [8]. *TPS* gene overexpression in Arabidopsis resulted in plants with increased leaf size and number per rosette, as well as in trichomes number [77]. In tomato, a natural increase in *TPS* gene expression in leaves of plants subjected to salinity and temperature stress was demonstrated, although no increase in expression occurred when stresses were applied separately [78]. Trehalose exogenous application in wheat leaves also showed the capacity to reestablish plants after a stress period [79]. The overexpression of the *AtTPS1* gene in tobacco plants provided a better acclimatization of the plants in a hydroponic solution containing cadmium and copper, while a higher expression of the catalase enzyme was also observed in relation to the controls [80]. Several authors have contrasting results on how trehalose influences the expression of other genes related to stress. While increased gene expression of genes related to abiotic stress was associated with increased *TPS* expression [81], other studies showed that several genes associated with stress were repressed [82], such as peroxidases, endochitinases, endoglucanases, lipooxygenases, chitinases, a ferritin plasma membrane integral protein, and cysteine proteinase [83]. Recently, several terpene synthase genes (*TPSs*) were identified in teak and related with plant defense and woody tissues [84]. In our study, *TgTPS1* did not show a significant effect in regulating drought effects. We suggest assessing reactive oxygen species genes for future molecular teak studies, and comparing with our results. Finally, the expression of *Heat Shock Proteins* (*HSP*) is a plant strategy for adaptation to high temperatures, since these proteins help mount other proteins and provide protection against denaturation [85]. Plants from arid and semi-arid regions can accumulate large amounts of *HSPs* [86].

### ABA signaling under drought stress in teak: Molecular insights

The *DREB* and *AREB* are the main genes involved in ABA signaling under drought stress. These transcription factors are responsible for activating a cascade of physiological and molecular reactions to protect the plant, and usually it is one of the first mechanisms to be activated with a lack of water. Here, both transcription factors showed activation/gene expression under drought stress (Fig 5). Regarding the importance of *DREB* genes, in *Cichorium intybus*, two *DREBs* were induced by different signaling pathways, being *CiDREB1* strongly affected by abscisic acid and *CiDREB1b* having high expression during low temperature exposure [87]; in addition, only *CiDREB1a* obtained high leaf expression, whereas *CiDREB1b* was strongly expressed only in the roots. Studies in *Vitis vinifera* have shown that *DREB* genes can regulate the glucan metabolism, lipid transport, endomembrane system and cell wall biosynthesis, among others [87]. Soybean plants overexpressing *AtDREB1a* showed a significant seed production when exposed to drought stress in both the field and in the greenhouse [88]. Although the initial focus of the *DREB* studies were on herbaceous plants, *DREBs* have been characterized in trees such as of *Populus euphratica* [89], *Populus nigra* [90], *Populus alba* [91], *Caragana korshinskii* [92], *Eucalyptus* [93], oil palm [94] and *Prunus persica* [95,96].

On the other hand, the characterization of *AREBs* in *Populus trichocarpa* showed that 11 are preferentially expressed in leaves and two others are exclusive expressed in roots; after ABA exogenous application, 8 genes were more expressed, whereas 6 had reduced transcription [97]. In Arabidopsis, the *AREB/ABF* overexpression under water stress showed that this gene is related to ABA and glucose signaling [98]. In *Arachis hypogaea* seedlings, the *AhAREB* expression was enhanced by the action of polyethylene glycol, NaCl, gibberellic acid (GA), abscisic acid (ABA) and salicylic acid (SA) [99]. Also, two tomato *AREB* isoforms (*SlAREB1* and *SlAREB2*) were observed, but only the *SlAREB1* had high leaf and root gene expression coinciding with drought and saline stresses [100]. Overexpression of *SlAREB1* also was shown to affect several genes related to oxidative stress, lipid transfer proteins (LTPs), transcriptional regulators and abundant late embryogenesis proteins (LEAs), as well as genes related to abiotic stress, such as protease inhibitors, catabolic enzymes and pathogenesis-related proteins. In teak, further assessments of other genes from the AREB family are essential, given its complexity and different types of functions.

### First approach to a model for teak drought stress

**Fig 6:**
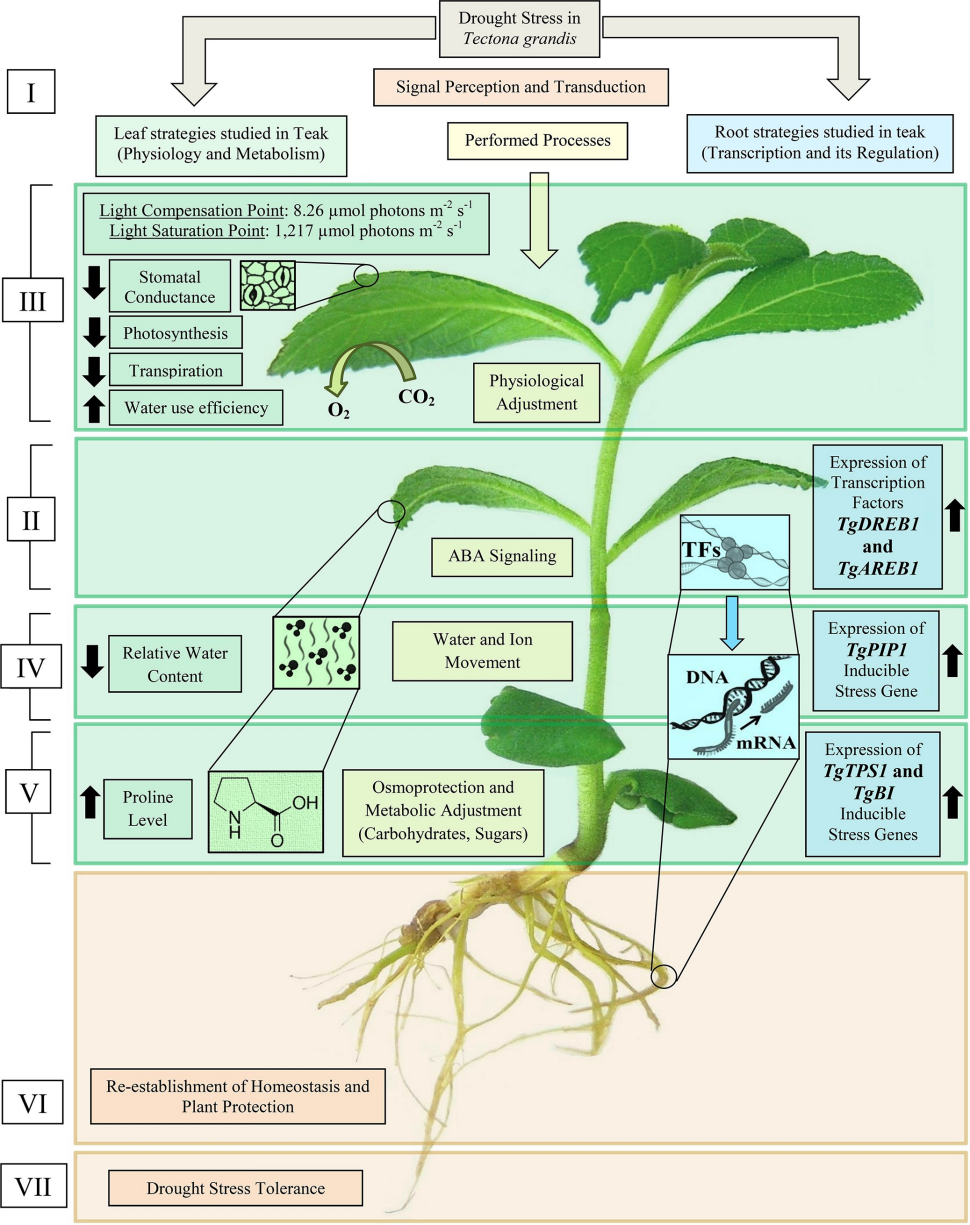
A schematic model for the physiological and molecular strategies under drought stress in teak deducted from this study. Roman numerals indicate the consecutive order of processes that the plant performs during drought stress, based on previous studies and reviews [101–104]: I. Signal Perception and Transduction (not studied here). II. ABA signaling. III. Physiological adjustment. IV. Water and Ion Movement. V. Osmoprotection and Metabolic Adjustment. VI. Re-establishment of Homeostasis and Plant Protection (not studied here). VII. Drought Stress Tolerance (not studied here). Green boxes denote the strategies studied in the teak leaf for some processes (at the physiological and metabolic level). Blue boxes denote the strategies studied in the teak root for some processes (at the transcriptional level).

It is usual that plants under drought stress show several metabolic pathway alterations, physiological modifications, transcriptional regulation and repairing with the expression of certain genes. We studied some of them in this work and could establish an initial schematic model for drought stress in teak (Fig 6). In this model, we propose seven processes/steps (yellow boxes, Fig 6) that occur during teak drought stress, based on previous knowledge [101–104]. The first (I: signal perception and transduction) and two last ones (VI: re-establishment of homeostasis and plant protection, VII: Drought Stress Tolerance) (Fig 6) were not studied here but were included in the model for didactic reasons.

In the second process, called ABA signaling (II, Fig 6), we found two transcription factors (*TgDREB1* and *TgAREB1*). Usually, this process follows immediately after the signal perception in order to activate several physiological adjustments (III, Fig 6). For teak, we found a significant dysfunction/reduction in stomatal conductance, photosynthesis, transpiration, and an increase in water use efficiency. Later, the water and ion movement (IV, Fig 6) and osmoprotection (V, Fig 6) can occur simultaneously to continue protecting the plant under a lack of water. The expression of the *TgPIP1* in the root rises after ABA induction to decrease water potential/content in the leaf, through the aquaporins. The expression of the *TgTPS1* and *TgBI* (carbohydrates- and sugar-related) genes in the root and proline content in the leaf rise after ABA induction to help with metabolic (and phenotypic) adjustment through osmolytes/solutes action. At the end, the plant is protected and tolerates the stress.

Previous studies in teak regarding drought stress have shown osmotic adjustment and fast mechanisms to avoid water loss, highlighting this tropical tree as desirable to be planted on a large scale in areas with possible drought problems, which is consistent with our proposed model. In India, six-month old teak seedlings with moderate and severe drought stress showed a significant reduction in relative chlorophyll content, height, diameter, leaf number, dry weight [31]. Two weeks of drought stress in teak seedlings decreased 50% of the growth rates (height and leaves length), but three weeks without watering significantly affected the photosynthetic rate, stomatal conductance and transpiration rate [32]. After re-watering, all plants completely recovered their development. Another similar study, with twenty days of drought, in two different teak clones, significantly increased carotenoid content and photosynthetic rate, but accumulated free proline and soluble sugars, demonstrating that the different physiological strategies (including antioxidative enzymes) protect photosynthetic apparatuses and alleviate drought stress [33].

Teak is a deciduous tree that has shown appropriate adaptation to climate change and dry seasons through its molecular and physiological apparatuses [105]. Although CO_2_ fixation in the cell decreases when seedlings are confronted by progressive drought (mainly by stomatal closure) [106], the strong protection by osmoprotectants (proline), transcription factors and enzymatic genes can allow homeostasis and sustain tissue metabolic activity in teak, even with tough events. The leaves of C3 plants commonly obtained a photosynthetic saturation in irradiances between 500 and 1,000 μmol of photons m^-2^ s^-1^, in this study the teak showed saturation at a irradiance above 1,200 μmol of m^-2^ s^-1^ photons of solar radiation, which indicates higher acclimatization efficiency to the greenhouse environment. This efficiency could also be expected in the field, but needs further study. At the same time, the maximum value of photosynthesis (A max) (Light Saturation Point) can be obtained by applying 1,217 μmol of photons m^-2^ s^-1^ at the leaf, producing carbon assimilation around 21.93 μmol CO_2_ m^-2^ s^-1^. This also means high teak quantum efficiency, similar to the one from several forest species of great economic value (see previous paragraphs).

Based on previous results and from this study, teak is still demonstrating high carbon assimilation efficiency, significant physiological plasticity under different water limitation conditions and the capability of fast drought stress recovery.

## Conclusions

Taken together, these results suggest that moderate and severe drought stress in teak tree seedling causes a reduction in stomatal conductance and leaf water content and triggers processes that lead to transpiration and photosynthesis reduction. The increase in water use efficiency, with control of the stomatal opening, maintain the minimum water balance necessary. *TgTPS1*, *TgDREB1*, *TgAREB1* and *TgPIP1* genes represent key points in the water stress response process. The physiological, biochemical and genetic study of stress due to water deficit in tropical trees is increasingly necessary, as climate change is predicted to become more intense and wood demand is increasing. From the experiments examining relative expression of genes involved in water stress, the most interesting genes to be studied for teak in the future are *TgTPS*, *TgDREB* and especially *TgPIP* due to the difference between moderate and severe stress when compared to the control. In addition to these genes, *TgHSP3* and *TgBI* are good candidates to be studied in possible genetic transformations with teak.

## Supporting information

S1 File Weighing of soil samples. This procedure was done for relative humidity determination.
(DOCX)

S2 File Primers for all genes. Primers were made for the amplification of the stress genes due to water deficit.
(DOCX)

S3 File Melting and standard curves. Those curves were obtained for the 8 genes studied in the article.
(DOCX)

S4 File Statistics of photosynthesis. Statistical analysis of the drought stress experiment as a function of the photosynthetic rate values.
(DOCX)

S5 File Statistics of stomatal conductance. Statistical analysis of the drought stress experiment as a function of the stomatal conductance values.
(DOCX)

S6 File Statistics of transpiration. Statistical analysis of the drought stress experiment as a function of the transpiration values.
(DOCX)

S7 File Statistics of WUE and IWUE. Statistical analysis of the drought stress experiment as a function of the Water Use Efficiency (WUE) and Intrinsic Water Use Efficiency (IWUE).
(DOCX)

S8 File Statistics of leaf temperature. Statistical analysis of the drought stress experiment as a function of the leaf temperature values.
(DOCX)

S9 File Statistics of RWC. Statistical analysis of the drought stress experiment as a function of the leaf relative water content (RWC) values.
(DOCX)

S10 File Statistics of proline. Statistical analysis of the drought stress experiment as a function of the proline values.
(DOCX)

S11 File Blast2Go. Gene ontology process annotations for Root transcriptome using Blast2Go.
(DOCX)

S12 File Annotation of drought genes. Drought Stress Annotated genes for Teak Root transcriptome.
(DOCX)

S13 File Metabolic pathways using KEGG. Number of metabolic maps of the KEGG platform for Root Library transcripts referenced for water stress. "S" is the number of sequences assigned to each map, and "E" is the number of localized Ecs. The five metabolisms with the highest number of transcripts are highlighted in grey.
(DOCX)

S14 File Gene submission to NCBI. Genes related with drought stress used in this study and submitted to the NCBI.
(DOCX)

S15 File Alignments of drought stress genes. Blastx and Alignment using Clustal Omega for TgTPS1, TgPIP1, TgAREB1 and TgDREB1 genes.
(DOCX)

S16 File Statistics of gene expression. Mean comparison for all genes under drought stress.
(XLSX)
