# Supplementary material for: Physiological and molecular responses to drought stress in teak (Tectona grandis L.f.)
Source: PLoS One. 2019 Sep 9;14(9):e0221571. doi: 10.1371/journal.pone.0221571 (PMC6733471; doi:10.1371/journal.pone.0221571)
Supplement: S1 File — This procedure was done for relative humidity determination. (DOCX) [file pone.0221571.s001.docx]

**S1 File. Weighing of soil samples.** This procedure was done for relative humidity determination.

| **Sample** | **Saturated Weight (g)** | **Dry Weight (g)** | **Water content** **(g)** | **Relative humidity (%)** |
| --- | --- | --- | --- | --- |
| **1** | 24.42 | 12.29 | 12.13 | 0.496724 |
| **2** | 22.86 | 10.52 | 12.34 | 0.539808 |
| **3** | 20.87 | 8.51 | 12.36 | 0.592238 |
| **4** | 30.07 | 12.46 | 17.61 | 0.585634 |
| **5** | 36.13 | 17.65 | 18.48 | 0.511486 |
