# Supplementary material for: Physiological and molecular responses to drought stress in teak (Tectona grandis L.f.)
Source: PLoS One. 2019 Sep 9;14(9):e0221571. doi: 10.1371/journal.pone.0221571 (PMC6733471; doi:10.1371/journal.pone.0221571)
Supplement: S2 File — Primers were made for the amplification of the stress genes due to water deficit. (DOCX) [file pone.0221571.s002.docx]

**S2 File. Primers for all genes.** Primers were made for the amplification of the stress genes due to water deficit.

| **Gene** | **Orientation** | **Sequence (5´-3´)** | **Primer Size** | **Amplicon Size** | **Efficiency** | **Accession Number**  **(protein Acc in parenthesis)** |
| --- | --- | --- | --- | --- | --- | --- |
| TgAREB1 | Forward | CAGGCATACACTATGGAGTTGG | 22 | 197 bp | 90% | MH003887.1  (AWW87323.1) |
| TgAREB1 | Reverse | GAATGCTACATCACCGCTTACCA | 23 |  |  |  |
| TgDREB1 | Forward | ATGGGTGGCTGAGATTAGAGAG | 22 | 183 bp | 93% | MH003888.1  (AWW87324.1) |
| TgDREB1 | Reverse | AGCGAAGAGGAGTCATTAGTGC | 22 |  |  |  |
| TgPIP1 | Forward | TCTTGGAGCAGCAATCATCTACA | 23 | 140 bp | 96% | MH003889.1  (AWW87325.1) |
| TgPIP1 | Reverse | ACCTGGACTTGAATGGAATGG | 21 |  |  |  |
| TgTPS1 | Forward | ATGTTTGAGGCGATAATGAGTGC | 23 | 158 bp | 96% | MH003890.1  (AWW87326.1) |
| TgTPS1 | Reverse | GAGGCATCAGCAAGGCTTTGAA | 22 |  |  |  |
| TgHSP1 | Forward | CAGGAGGAGAAGAACGACAAGT | 22 | 134 bp | 95% | KR092432.1  (AKT44363.1) |
| TgHSP1 | Reverse | GTCACTGTCAGCACACCATTCT | 22 |  |  |  |
| TgHSP2 | Forward | ATGACGAGTCAGAGGAAGAAAA | 22 | 171 bp | 96% | KR092433.1  (AKT44364.1) |
| TgHSP2 | Reverse | CTGTCCAACCATATTCTCCAGT | 22 |  |  |  |
| TgHSP3 | Forward | TTAACGCCGGAAATTGTAGC | 20 | 100 bp | 94% | KR092434.1  (AKT44365.1) |
| TgHSP3 | Reverse | ACCGTCACTACCAGCACTCC | 20 |  |  |  |
| TgBI | Forward | TGTCGTTCTTATGCTGTTTGCT | 22 | 149 bp | 96% | KR092435.1  (AKT44366.1) |
| TgBI | Reverse | CGTCGTATGTGTATCGCTTGAT | 22 |  |  |  |
