# Supplementary material for: Physiological and molecular responses to drought stress in teak (Tectona grandis L.f.)
Source: PLoS One. 2019 Sep 9;14(9):e0221571. doi: 10.1371/journal.pone.0221571 (PMC6733471; doi:10.1371/journal.pone.0221571)

**S3 File. Melting and standard curves.** Those curves were obtained for the 8 genes studied in the article.

**AREB DREB**


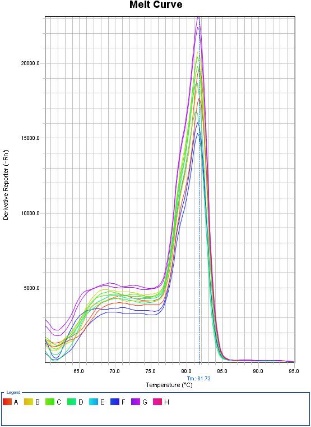

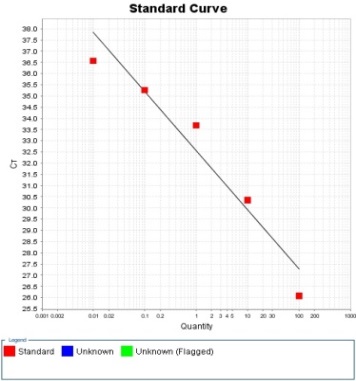

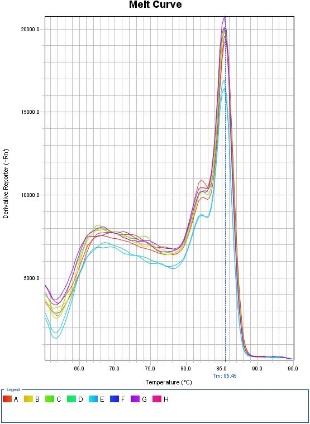

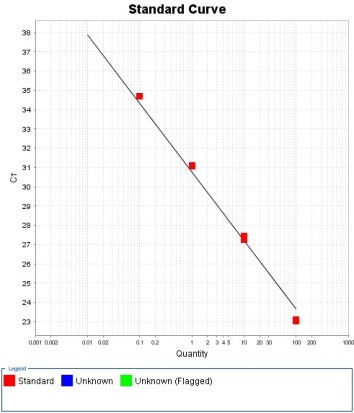


**PIP TPS**


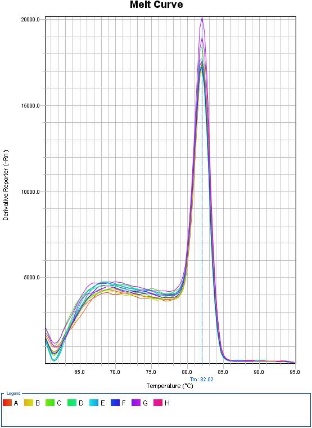

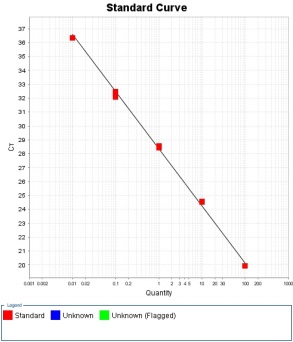

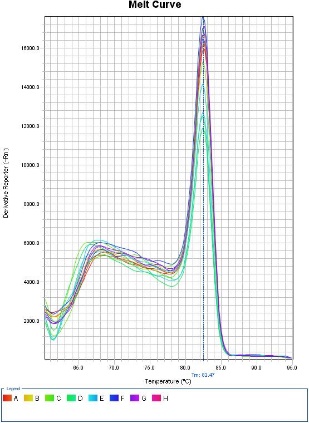

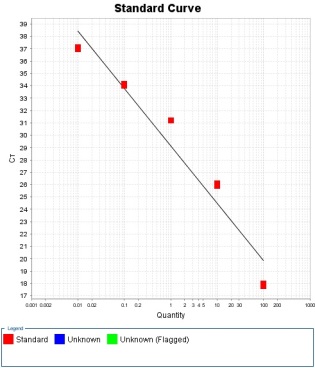


**HSP1 HSP2**


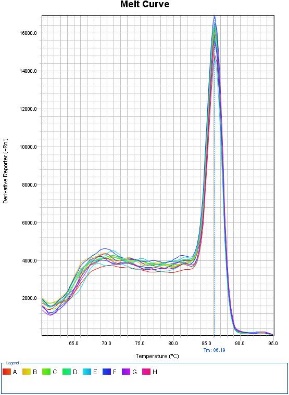

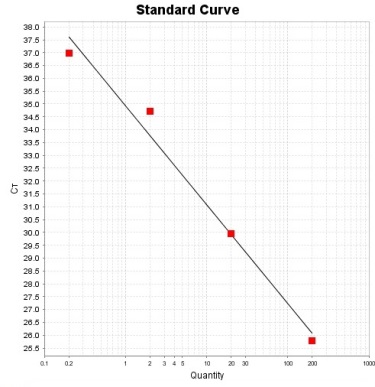

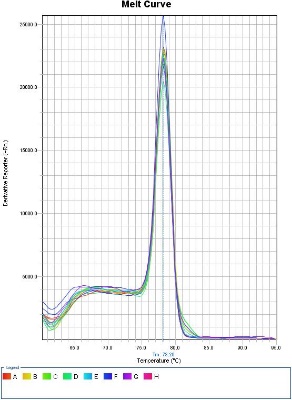

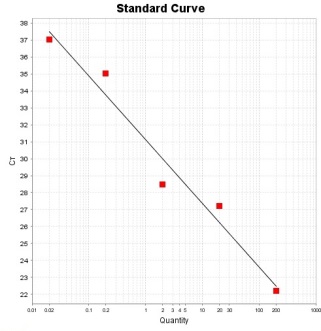


**HSP3 BI**


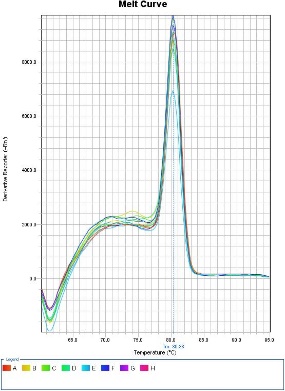

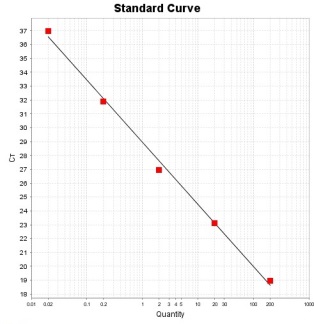

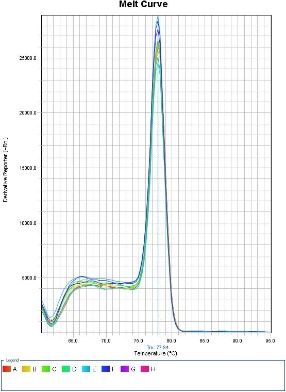

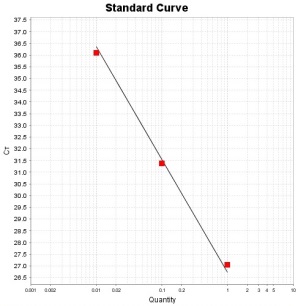

Supplement: S3 File — Those curves were obtained for the 8 genes studied in the article. (DOCX) [file pone.0221571.s003.docx]
