# Supplementary material for: Physiological and molecular responses to drought stress in teak (Tectona grandis L.f.)
Source: PLoS One. 2019 Sep 9;14(9):e0221571. doi: 10.1371/journal.pone.0221571 (PMC6733471; doi:10.1371/journal.pone.0221571)
Supplement: S4 File — Statistical analysis of the drought stress experiment as a function of the photosynthetic rate values. (DOCX) [file pone.0221571.s004.docx]

**S4 File. Statistics of photosynthesis.** Statistical analysis of the drought stress experiment as a function of the photosynthetic rate values.

| **HOMOGENEITY OF VARIANCE** | | | | | |
| --- | --- | --- | --- | --- | --- |
| Bartlett | | X^2^ = 155.73 ** | | P < 0.01 | |
| **NORMALITY OF DATA** | | | | | |
| Lilliefors | | D = 0.1189 ** | | P < 0.01 | |
| **ANALYSIS OF VARIANCE** | | | | | |
| ANOVA (F) | | F = 47.4728 **** | | P < 0.0001 | |
| Kruskal-Wallis | | H = 224.1199 ** | | P < 0.0001 | |
| **CONTRAST OF MEAN** | | | | | |
| Drought stress + Irradiance1 | Mean^2^ | Tukey | t | Dunn | SNK |
| T1 - Control + 1400 | 8.6942 | a | a | a | a |
| T2 - Moderate + 1400 | 3.2766 | b | bc | b | bc |
| T3 - Severe + 1400 | 2.1973 | b | c | b | c |

^1^ Irradiance value in μmol of photons s^-1^ m^-2^

^2^ Mean value in μmol CO_2_ m^-2^ s^-1^

^**^ Significance level α = 0.01
