# Supplementary material for: Physiological and molecular responses to drought stress in teak (Tectona grandis L.f.)
Source: PLoS One. 2019 Sep 9;14(9):e0221571. doi: 10.1371/journal.pone.0221571 (PMC6733471; doi:10.1371/journal.pone.0221571)
Supplement: S5 File — Statistical analysis of the drought stress experiment as a function of the stomatal conductance values. (DOCX) [file pone.0221571.s005.docx]

**S5 File.** **Statistics of stomatal conductance.** Statistical analysis of the drought stress experiment as a function of the stomatal conductance values.

| **HOMOGENEITY OF VARIANCE** | | | | | |
| --- | --- | --- | --- | --- | --- |
| Bartlett | | X^2^ = 399.466** | | P < 0.01 | |
| **NORMALITY OF DATA** | | | | | |
| Lilliefors | | D = 0.2038 ** | | P < 0.01 | |
| **ANALYSIS OF VARIANCE** | | | | | |
| ANOVA (F) | | F = 62.99 **** | | P < 0.0001 | |
| Kruskal-Wallis | | H = 280.63 ** | | P < 0.0001 | |
| **CONTRAST OF MEAN** | | | | | |
| Drought stress + Irradiance1 | Mean^2^ | Tukey | t | Dunn | SNK |
| T1 - Control + 1400 | 0.122 | a | a | a | a |
| T2 - Moderate + 1400 | 0.029 | b | b | b | b |
| T3 - Severe + 1400 | 0.017 | b | b | b | c |

^1^ Irradiance value in μmol of photons s^-1^ m^-2^

^2^ Mean value in mol m^-2^ s^-1^

^**^ Significance level α = 0.01
