# Supplementary material for: Physiological and molecular responses to drought stress in teak (Tectona grandis L.f.)
Source: PLoS One. 2019 Sep 9;14(9):e0221571. doi: 10.1371/journal.pone.0221571 (PMC6733471; doi:10.1371/journal.pone.0221571)
Supplement: S7 File — Statistical analysis of the drought stress experiment as a function of the Water Use Efficiency (WUE) and Intrinsic Water Use Efficiency (IWUE). (DOCX) [file pone.0221571.s007.docx]

**S7 File.** **Statistics of WUE and IWUE.** Statistical analysis of the drought stress experiment as a function of the Water Use Efficiency (WUE) and Intrinsic Water Use Efficiency (IWUE).

| **ANALYSIS OF VARIANCE- WUE**^1^ | | | | | | |
| --- | --- | --- | --- | --- | --- | --- |
| Source | Sum of  Squares | Df | Mean Square | F | | P -value |
| Between groups | 0.22219 | 2 | 0.111098 | 1.03 | | 0.4111 |
| Within groups | 0.64431 | 6 | 0.107385 |  | |  |
| Total (corr.) | 0.8665 | 8 |  |  | |  |
| **CONTRAST OF MEAN – WUE** | | | | | | |
| Drought stress + WUE | | Mean | Tukey | |  |  |
| Control + 1400 | | 2.1683 | a  a | |  |  |
| Moderate + 1400 | | 2.42606 |  |  |  |  |
| Severe + 1400 | | 2.54471 | a | |  |  |

| **ANALYSIS OF VARIANCE- IWUE**^1^ | | | | | | |
| --- | --- | --- | --- | --- | --- | --- |
| Source | Sum of  Squares | Df | Mean Square | F | | P -value |
| Between groups | 0.00588 | 2 | 0.002943 | 8.46 | | 0.018* |
| Within groups | 0.00208 | 6 | 0.000348 |  | |  |
| Total (corr.) | 0.00797 | 8 |  |  | |  |
| **CONTRAST OF MEAN - IWUE** | | | | | | |
| Drought stress + WUE | | Mean | Tukey | |  |  |
| Control + 1400 | | 0.07161 | a  b | |  |  |
| Moderate + 1400 | | 0.1157 |  |  |  |  |
| Severe + 1400 | | 0.13220 | b | |  |  |

^1^ WUE and IWUE values in µmol CO_2_ m^-2^ s^-1^ / mmol of H_2_O m^-2^ s^-1^

^*^ Significance level α = 0.05

^**^ Significance level α = 0.01
