# Supplementary material for: Physiological and molecular responses to drought stress in teak (Tectona grandis L.f.)
Source: PLoS One. 2019 Sep 9;14(9):e0221571. doi: 10.1371/journal.pone.0221571 (PMC6733471; doi:10.1371/journal.pone.0221571)
Supplement: S8 File — Statistical analysis of the drought stress experiment as a function of the leaf temperature values. (DOCX) [file pone.0221571.s008.docx]

**S8 File.** **Statistics of leaf temperature.** Statistical analysis of the drought stress experiment as a function of the leaf temperature values.

| **HOMOGENEITY OF VARIANCE** | | | | | |
| --- | --- | --- | --- | --- | --- |
| Bartlett | | X^2^ = 12.59^ns^ | | P > 0.05 | |
| **NORMALITY OF DATA** | | | | | |
| Lilliefors | | D = 0.1465 ** | | P < 0.01 | |
| **ANALYSIS OF VARIANCE** | | | | | |
| ANOVA | | F = 2.1957 * | | P = 0.0261 | |
| Kruskal-Wallis | | H = 19.6015 * | | P = 0.0120 | |
| **CONTRAST OF MEAN** | | | | | |
| Drought stress + Irradiance1 | Mean^2^ | Tukey | t | Dunn | SNK |
| Control + 1400 | 37.66 | a | a | a | a |
| Moderate + 1400 | 37.39 | a | ab | a | ab |
| Severe + 1400 | 37.42 | a | abc | a | b |

^1^ Irradiance value in μmol of photons s^-1^ m^-2^

^2^ Mean value in °C

^*^ Significance level α = 0.05

^**^ Significance level α = 0.01
