# Supplementary material for: Physiological and molecular responses to drought stress in teak (Tectona grandis L.f.)
Source: PLoS One. 2019 Sep 9;14(9):e0221571. doi: 10.1371/journal.pone.0221571 (PMC6733471; doi:10.1371/journal.pone.0221571)
Supplement: S9 File — Statistical analysis of the drought stress experiment as a function of the leaf relative water content (RWC) values. (DOCX) [file pone.0221571.s009.docx]

**S9 File.** **Statistics of RWC.** Statistical analysis of the drought stress experiment as a function of the leaf relative water content (RWC) values.

| **HOMOGENEITY OF VARIANCE** | | | | | | |
| --- | --- | --- | --- | --- | --- | --- |
| Bartlett | | | X^2^ = 22.73 ** | | P < 0.01 | |
| **NORMALITY OF DATA** | | | | | | |
| Lilliefors | | | D = 0.1406 * | | P < 0.05 | |
| **ANALYSIS OF VARIANCE** | | | | | | |
| ANOVA | | | F = 34.1528 ** | | P < 0.0001 | |
| Kruskal-Wallis | | | H = 30.9434 ** | | P < 0.0001 | |
| **CONTRAST OF MEAN** | | | | | | |
| Drought stress | Mean^1^ | Dunnett | Tukey | t | Dunn | SNK |
| Control | 72.62 | \| | a | a | a | a |
| Moderate | 81.60 | \| | b | b | b | b |
| Severe | 63.05 | \| | c | c | a | c |

^1^ Mean value in %

^*^ Significance level α = 0.05

^**^ Significance level α = 0.01
