# Supplementary material for: Physiological and molecular responses to drought stress in teak (Tectona grandis L.f.)
Source: PLoS One. 2019 Sep 9;14(9):e0221571. doi: 10.1371/journal.pone.0221571 (PMC6733471; doi:10.1371/journal.pone.0221571)
Supplement: S10 File — Statistical analysis of the drought stress experiment as a function of the proline values. (DOCX) [file pone.0221571.s010.docx]

**S10 File.** **Statistics of proline.** Statistical analysis of the drought stress experiment as a function of the proline values.

| **HOMOGENEITY OF VARIANCE** | | | | | | |
| --- | --- | --- | --- | --- | --- | --- |
| Bartlett | | | X^2^ = 6.20538^ns^ | | P > 0.01 | |
| **NORMALITY OF DATA** | | | | | | |
| Lilliefors | | | D = 0.19740^ns^ | | P > 0.1 | |
| **ANALYSIS OF VARIANCE** | | | | | | |
| ANOVA | | | F = 3.4687^ns^ | | P = 0.0638 | |
| Kruskal-Wallis | | | H = 2.0600^ns^ | | P = 0.3570 | |
| **CONTRAST OF MEAN** | | | | | | |
| Drought stress | Mean^1^ | Dunnett | Tukey | t | Dunn | SNK |
| Control | 80.91 | \| | a | a | a | a |
| Moderate | 110.47 | \| | a | ab | a | a |
| Severe | 280.62 | \| | a | b | a | a |

^1^ Mean value in μg proline g^−1^ FW

^*^ Significance level α = 0.05
