# Supplementary material for: Physiological and molecular responses to drought stress in teak (Tectona grandis L.f.)
Source: PLoS One. 2019 Sep 9;14(9):e0221571. doi: 10.1371/journal.pone.0221571 (PMC6733471; doi:10.1371/journal.pone.0221571)
Supplement: S11 File — Gene ontology process annotations for Root transcriptome using Blast2Go. (DOCX) [file pone.0221571.s011.docx]

**S11 File. Blast2Go.** Gene ontology process annotations for Root transcriptome using Blast2Go.

Graphic of Distribution of GO Terms for Biological Process (2nd level) between analyzed libraries

Graphic of Distribution of GO Terms for Molecular Function (2nd level) between analyzed libraries

Graphic of Distribution of GO Terms for Cell Component (2nd level) between analyzed libraries
