# Supplementary material for: Physiological and molecular responses to drought stress in teak (Tectona grandis L.f.)
Source: PLoS One. 2019 Sep 9;14(9):e0221571. doi: 10.1371/journal.pone.0221571 (PMC6733471; doi:10.1371/journal.pone.0221571)
Supplement: S12 File — Drought Stress Annotated genes for Teak Root transcriptome. (DOCX) [file pone.0221571.s012.docx]

**S12 File. Annotation of drought genes.** Drought Stress Annotated genes for Teak Root transcriptome.

>comp70_c0_seq1 molybdenum cofactor sulfurase

atgagtgatgcatacaatttatttgcatttccatcagaatgcaatttttcaggtctgagattcaaccttgacctcgtaaatgttataaaggacggttcatatgagatgcgaggaacttctccacgtgacagtggtcactggatggttctgattgatgctgcaaaaggaagtgcaacatccccacctgatttgtcaaaatacaaag

>comp311_c0_seq1 chaperone -domain superfamily isoform 1

atttcgccggcctgaacgcgtacgaagtactcggagtgtctcagacaagctcatttgcagaaatcaaagcttcatttcataagttggcaaaggaaacgcatcctgacctagctcagtcgcaaaaccattcgtcttatgcttcgaagaaattcattcaaattctcgctgcttatgagatcctctcagactctaccaagagagctcattatgatcgccatattttatctcaaagaattcccatcgagagacactctagacaagataccataatcttcaattataactcctatggacaccccactaaccagatggaagttgtggaatggctgaaatggtacagatacacaataaatgacattttgtctgaaaggagagtgacagctggatcaggttattttgatatcctggaaagagatttttactcggccattcatgcagcattctatggacctgagattgagtctatggatctccttcccgaccgttttgaagctgaggagaggtctatgcatactacattggaggtgctacacttagtttcaggtcgagacctttttgggatggtatgtctagctaa

>comp441_c0_seq1 poly(adp-ribose) glycohydrolase 1

atcctctgccgtattctggacgccggcggttgtggaagccctcaaatccctctccaagggaccgagtcacagcaatgtcaactccggtcaactctttgccctcgccattgccgacctacgcaattctcttgggtttcattctctccatcccttcgcctctctcggcttctctgtcttcttcggcgatttgatgaacaagaatgaggctgagaaatggtttggggaagtggttccacgacttgctgatttg

>comp545_c0_seq1 zeaxanthin epoxidase

tgggcggaggaaagattcagtggtatgcatttcacaacgaatcacctggaggtctggatgttcccaaaggtaaaaaggtgaggttactcaaattatttgaaggctggtgtgatgacgtcatagatctgttgcttgccaccgatgaagatgcgattcttcgacgcgacatatatgatcggactccaatctttacgtgggga

>comp790_c1_seq1 pto kinase interactor

tacagccaaacctgtgctagaaattcttcgtctggctgattgctggagtctagcttttttattgccacagcctgtctgcttttcaggatgccatggtatacccttccataagatccctgaccaatggaacactttaaactaaaattatcagttatgtctaccaattcatctactggaatttcagggacaacgattgatactggaatggcaggg

>comp1096_c1_seq1 zeaxanthin epoxidase

tttatccctcacctttgaccatatcccatcagcgccgacaagaacatctccttcataacactccccatttttaagtcttactttaaccttttcaccattatgctcaaagtccaccacattactctcgttcatgatagtatctgagccgaccgcacaagcaagaatttgttgcaaagtcatgcggctaatgactcttgtgactgg

>comp1105_c0_seq1 monoglyceride lipase-like

gattaagagggtggcggaggataagggagatggagatgaaaagggtacagtgagggagttttcattgtttgttacgtcgagaggtgacactatttttactcagtcatggacccctgttaaggttcaagtcaggggactggttgttctcttgcatggtctcaacgaacacagtggaagatataacaactttgccaagaaactgaacgcaaatggcttcaaagtttatggaatggactggattgga

>comp1167_c0_seq1 galactinol synthase

ggtggggttggccaagggtctgaggaaggcgaagacagcttatcctctagtggtggcggtgctgcccgacgtgccggtggggcaccgacgcatattggaggatcaaggctgtattgttagggagattgagcccgtgtacccgccggaaaaccagactcagtttgcgatggcgtattacgtcatcaactactccaaacttcgt

>comp1427_c0_seq1 polypyrimidine tract-binding protein

gtgcagatccagaaaggcccatacctgtaattcaacaaatataacaataacaaatcaacatgtaacaggtggatctggttgcgagttttgttcaatataacaacaacaaactaaatctttatcccatcaagtgcggtcagctatgtggatcatatctcgtcattgtatcaaagcacatgttctcatctacatttaagtacaacatgtctttcttattagtttcctaccaattctttttcggtctttttctttcccttttagtttgagatgatgattctctacatcatagacactgtatcaggatatccaaaggtctcttatgcacatgtcccaaccatcttaaacgtgtttctgtgattttctcatctattaaggctacccccactttctttataatgcatttgttacttattctatctaatctagtgtgttgcacgtccatctcaacattttcatttctacccttcattttttgt

>comp1679_c0_seq1 kda class i heat shock protein

gacaagtggcaccgcgtcgagaggccgcggggcggcttccggcggcggttttatctgccggcgaatgtgaagactgatgaagtgaaggcggcaatggagaatggagttctcaccgtcaccattcctaagcaggaaatcaagaagcctgagatcaagaacattgaaattgtcggaagttgattgatccaaagcttcactgtcgttgcaaatatgtgagatgtgtattgatcaactcgatcgtcgcttttctggaatgttgtttgtttttgcatcacaaggctgttgggtgtttccttttgttgtttgctgtcatgtaatattggtgtgttttgtaatga

>comp1702_c1_seq1 multidrug resistant abc transporter family protein

gggccaatattgcctatggaaaggaaggcaatgccacagaagcagaaattatagatgctgctgagttgtcaaatgcccataaatttattagtggcatgcaaaagggatatgagacgatggtgggtgaaagggggatccaattatccggtggacagaagcagcgagttgctactgcaagagctatagtaaaggctccgaagatactactacttgatgaggcaaccagtgctcttgatgctgagtctgagaaaatggttcaagatgccttagagagacttatggtgcatag

>comp1924_c0_seq1 dehydration-responsive element-binding protein

atgtatggatcttgtgcccggcttaatttccccagttatggtccatctgcaaaatgcactaatgactcctcttcgctgcttgctacatcaactgactcgacaaactctagcatctcagaggtatgttgtagtgatgagggaccaaattctgatgttcccaagataaaaactgaagaggctgaaggtttgtcacggatcattgacaataggcatgctgcgtcgcatgaagctggcaccccgatgagtgtagtcaaagaggaagtaatagaagaagctccaagggagaaagtgcaggaagaagcaatgaaggaggaaggtaagacagagtcgatggatcccttgtatggtggtgatgctgagaagaagcgtactgcggagaaaccttatataagccaccagagtgctgacgctgggcaac

>comp2181_c0_seq1 heat shock protein 70 -interacting

aaaatttgtcagcatcttgcaaattttctggtagcagcttgatgaggtcttagttactagtttttctgaagactggttaatcttatgagcatggataaagtatcccccgactgcccatatcctggttgcttcttctgtgtaatgaaggaaggaaacccaagcaagcgcagagcaagtgtattaaagttcttccgggaacttccttcacaggatgatgatggtcaagttcttcctattagtggtctatggaatactgcaatggcacatcctaacgatcctgagttcattgagctgggaatttttgaatgcatggcag

>comp2511_c0_seq1 probable disease resistance protein rdl6 rf9-like

tcgttggaagaaaacacaaagctttccacagtttatttgctaggaaaattgaatccacctgtcatacatagattccctaagagtctcactgatatcacactgtcaggatcagaattaacagtggacccaatggaatttctgggaagccttccaaatctccagattcttcgactactggctcagtctgtggtgcgcagtaggatacattgctccagaagatgttttcctgtgcttaaagttctttacatttggaagctggagagtctggagagatggga

>comp4676_c0_seq1 riboflavin biosynthesis protein chloroplastic-like

gtggtcccctgcgcatacaaatacccttcgccgaccaccaccctttctccattatctgaagtggtggcgatgacgcaaccaaagttggggtgcggcgccgtgaacccagccgatttgtctgctaactcggcggcgcgtttgatgtaggcggcgtcgaaggcaagtgatgatggtgatgaggaagaagatgcgagagttgatctgcaggtgaaggaagaagggacctttgcgggtgccaatgagaacgccatcatcgccg

>comp5038_c0_seq1 molybdenum cofactor sulfurase-like

cacaatgtcactggtcatcaagccacagttgctctggctatggggatttccataaagagtagaattcaaatccttgaaaatgttctccatctgcgcctcagaatataaagttgcacccgcatggtccaagtacacaacatcattcagtcgtttaaattcagttgctctgatcccatcaatgtttttggatgaatttggatacccataatcttgaccgaactctttcaagaattgtaccttctcagcgtcatcggccatgtttgaactcgacactaaacaagaattctactcagtcttgttaagacgaagaaaattggaagacaaaacaactaaaccaacgcgaccacgccgtaataggacatga

>comp6071_c0_seq1 heat shock protein binding isoform 1

accaacagttcccccatccagaacgactggaacgccatcaatttcagttatcattgctgtagttccaatgggatggctgcaggatccaactccacacttccttctggggcacctggagcgtcttggctggtaagaaagtgattcagaacccgatgagaaaatcgaaaaatttcttcctttgggatcctccttgtcgcatttaagtcagactttggaagaaaaaccgttctaaaaccagccactttcacaagaggagcaatagatacaccacgctcctcagtgtagttgtcgagtaccatcaccatgtcgtacttgtgcacaacatcatcaggagtattcttgttccaatctgaagaccaatttctataaactgcccaagaatcacccttctggggcaatatcacaatactcccacgcgggctttttgaccaattaaccctttgagaaaaggcatt

>comp6444_c0_seq1 octicosapeptide phox bem1p domain-containing protein tetratricopeptide repeat-containing protein

actttatggctttctcatactttaacatggcaccttcacagtccctcttctgaaataacttattaccctcatccttcaactcctgcgacatcgaaatgaatacggctgtgtccttatcataagctcttgggctatcttcgcccgccttagttttcttcagatttgattcatctgacttctgtccaggagattttttgctctttcctccatgtttccccatcctgaatcccaatagttcaaactaccgaaaccttgataacagacaattttaacaagctttctacactattccaacccaatttattctcaaaagtctactaattagcttcaaattccctggtatataactctatcaagccaataattatgaacaaaaactacagaaaatttaccaatccaatcaatctaaggataaagtaaatgaaccagaaccaacatcattcaaaacactaactcacatccaaaaactgtgaacaaaa

>comp7482_c0_seq1 probable serine threonine-protein kinase drkd-like

gatcttgaaccttcaacttgcttttccattttactaagataacaggaatatcctgcgtaatgattctggacacctgaaagttgcggattttggagttagcaagctgcttagagttgccaatagaattaaagaagacaggcctctgacttctcaagatacttcttgtcgctatgtggctcccgaggttttcaggaatgaagagtatgacactaaagtg

>comp7704_c0_seq1 endoplasmin homolog

aagccgcgacacctcagcctggaatttgaacttctcagcattcgcgcgcagtgttcttctagacatcgattcagcttctctcttaacaacatcagaatcggtagataatccatgtggaacggctccgatcttctcttccaccttcggcggatcattcgaagcatctgaatcaacttccgcatttgcatgtattctcctaccttggtcagggagaagaaacaagaggcagagcagaaat

>comp8323_c0_seq1 transmembrane protein

atgaaacaccaaaatataccaggaggcaaactgcagcaagatcatcaagaggcaaatcagcatctccaaacctgaatggaaggatgtcatcaacatagtgaaaagttcgtccaagaacaacagatataattgtcattaccgcaagcgcaccaaaagtcccaataaaagtcacggcagcagagttcctggctgctagtagtgccgcaataaagaaggttttatcaccaagttccgaaaagaatatcaacaagaatgcctgtactaatatcgccggtat

>comp8664_c0_seq1 glutathione s-

aaaaaggaattcatgggggctctcaagttgttggaaggtgagctaggggataagacttactttggtggtgagaagttcgggtatgtcgatgtggccttagttactttctacagctggtttcatgcctatgagacttgcggcaactttagcattgaagcgcattgtcccaaattgatcgcatgggccaagagatgcatggaaagagagagtgttcgcaag

>comp11664_c0_seq1 kda class i heat shock

gaaattccactaacaaaatctttatctgacaaatcaagccatccattgttgttgcaagaaagctgaaaaaatgtctctgattccgagcttttttagcagccgccggagcaacgttttcgatccattttcgttggatatttgggatccatttgagggctttcctttctccaccaccctcgacaaccttccctcctccgcccaagaaaccagggccgtggcgaatgctcgcatcgactggaaaga

>comp11815_c0_seq1 monoglyceride lipase-like

cgtggtgtctgatactatggagtctttaggaattgtattatcgttctactactgttattcctcaagacaagtgtttcatcgcctgctcaactctcttaagtatctgagtgcacggacagtggcatcaatggtcgctgcctcttcgacctccaatatctatatcacaatctactactcaaccatgcaatgatactctctgtgacttcttccttttcaggttcgaaaagtagatcatgcaaaagcccttgatagagcacaatgcttttgtcagtcgatgaagcctcatcgtagagcttctgagacgcttcaggatcagttactgaatcgccagtgccatgaagaactagaaatggcactgtcaacttgcccaagttctgctgcaaataagttgttatccgaagaatttcatagcctgttcttaccctaatagatccagtgaataccagtggatccgaatacttggccaccagtgcatctggatctctggagactgcaatacccctcttgtttgctgcactgaattgaaaccttggcaacaagaaggaaaaaactggagcaagaacagcaaaaattggatgggatggctg

>comp11870_c0_seq1 defective in meristem silencing 3

aaaacatacgaaactaacatgtttaactaatctgcccatttattcagcatacaactatggatggattcctccaccaatcaagcatccctgaaaattgagaaaccctatcctcaagttcaaccgtgttgccagactcacgccacaagggttccaaagaagatcagatagaagataaaaccagttagccagtgttgtgaagctcttcaatttcctcccctattttgccagaatgaaatcatcttggggttgatccacgtccagctggatactgagagacatgtgatgagctttcagcaagaaactggagaaagtcccgcttcttggtttcgtagttgaaccttgcacggtttaatagatcctgctctctccgcatatcttcccacattcgatccctcttccattttgtttccttcatcctgttttcaatctcaaggtagctctcagcaaggttcaacctttccgagccaattgggaatttgacacctatgtcttccttatggtgacccaaggagaacactccatgacttttgatcattcctccatccagagatattgctccatttcttatacaagaaagagcctttaccatgtcttcccttgatttatatacttgcaaatccgagaaaagatgataaaatagtgtttcccttaggctgtggccattctttgagatacaatacaagttagtactgtcaatggtgatcatattaacagcaaaacctacaaagccaggaggagtttctccattggttaatcttggttttggaagcgcaagcctccgttgaggataatcagctatgaactcgccagcatatggtcttatatcctcaagacagataacaagaaatcgatcagccaggggcctcccaat

>comp11890_c0_seq1 overexpressor of cationic peroxidase

actcccagaagtgctttaaccaattgtttcctgccacgtcggccgccgccgcggcgtttcgccggtctccgctgctccagcctccgtcgagagagcgccaacccttccgtaactcccaagaaaattaacaagaaaaaaatcgctcgcgaaaatgtgggacaagatgaggatatagatgaagatgcttttgaggcacttttccgtcagcttgaggaagatttgaaaaatgacgaattatatggtaatgacggtggggatgatgatttaagtgaggaagagcttgcgaaacttgagcaagaattggctgaggctctgggggatgatgaattatttggggcattggactctgctgcctttggagaaaccaagaatgaagatgatgatgttgctgataatggggatggatatgaagttgacgatgataatgaagaagaggaaaggcctataaagcttaaaaattggcaactcaagaggctggcctatgcgttgaaaaatggtcgtcgcaagactagcatcaaaaatcttgctgctgacctatgtcttgatagagcagttgttctcaaattacttcgtgaccctcctcctaatcttgtcatgatgagtgctactttgcctgataaacctgcaacaacaatcttagatcctgtggagaaacgcgtggaaactgttcctttggagaaaacgacccatgctgcaaagcctgaagttgaggttaaagtgccagttcatgtcatgcaaaacaactggtctgctcgaaagaggctgaagaaagtgcaagttgaaactcttgagcaagtctacagacgaacaaagcgcccaactaatgccatgattagcagcatcgttcatgtcacaaacctgcctcgcaaaagagttgtgaaatggttcgaagataaacgagctgaagatggggtacctgaacatcgacttccatatcaacgatctgctagcgaatctgtctttaccagctagaacatcccctgttcttttcaggcatttccagcttcgagtgcagtgtacaggaaacctatttctcattgcccttcctggtcttgagttagttgaggttctttactgtgtagctttgctgtatctagttactgattgatttgcatgctcttcttgtgcggaatgttgtgagacgtattttatttcaccaatacaagttccggaagagaagcaaaatcattcacaaacatgtgcatgcatatatgcacttcattaatttcaaaaaaattttgcagcacacaaattgagggaagattggtccataa

>comp11941_c0_seq1 9-cis-epoxycarotenoid dioxygenase

aactttaggccatggctctgcaatggcgagatatgcatatcgtgttttccttccaagtttgtttctgttcaccattcctgcctctaaattaacttgatcatcatcggacatgatcggcctcctcgtagacttgccggttttcaaatttagccggatttcagacaaaacgctcttcaatccctcatcgcattcattgaaaatggagtccggcggagtcatacacgaaccgaccacgacgacttcatctgtttcaggctcctcccatgcattccagagg

>comp12089_c0_seq1 transcription regulatory protein snf2-like

gtgcaagctattctggataggagtaccagtcaaaagaagtctccgccgaattcgatagcctgaaaccagtgtccgtgcaagaacacaatcataatttttaagtcgatgtccttcgtcaacaaccaggtagtaccagtgaattttcttcagaaatgctttgtctctaatgatgagatcgtaatgggtaatcaaaacattgaacttcccttctccagaatactcttccctcagggcctttctctcatctagacgtccatcatagagaacagcagaaatgctaggagcccaggtagtaaattcatttatccaatttggcaggacagcctttggggccacaatcaagtggggcccagtcacacccttgttttccatgagataagctatcaaagagatagtctgaattgttttccccaaccccatttcatcagccaaaattccat

>comp12112_c0_seq1 kda class vi heat shock

caatcaacgttgcgtttgctggagctgtgggaactcggcagcaacacatccggttcaaactcccataacggaaaggcatccgatggatcaaagaaatttccaaacaacaaagggctgaagagtgatcctgtcttatgaatagtcgagttttctttgctcgtaagtgttgcaaatacaccttctttgagaggttgacaccatttctgtggatttggatcttcaaatttgatctcaagttgtttgctactggtcatctaatgtttgtg

>comp12193_c1_seq1 probable glutathione s-transferase

gattaaatggtagcctctgtgttctcaaccatataacaaaatcacgaagcttgtggggttctgtaagagacttggagaaactctctcttttcatgcaccttttagtccacgctataagtttagggaactcagtttctatgttaatgtttcccaagtcctgataggcacagaaccagctgcagaatcctccaagaacaacgtccacatatcccagtgtctcgcctccaaaataaggcttctctccaagttcttcttccagcagcttcaggctatctctcaactccttttcagcttcttccaattcttctttgctcttgtaccatgttgtcttcatcccagcagcatgtagcttcttgtcaatgaaatcagcccagaacctagcttgagctttctcataaggatcagaggggaggaaaagtggagaatgattgcagacctcatcgatgtactcgatgatgttccaagactcacatatcggtttcccgttgtgaatcagaactgggattttcttgtgaactggattcatttccaatagaagtgggcttttgttccgaagatcctcttccctggactcgtaattgattcctttctctgccagtgcaattctaaccctgccaccgaacgcgcttggccagaagtccagtagaatccactcatcattcattttctttcaaactacttcccac

>comp12218_c0_seq1 54s ribosomal protein mitochondrial-like

aaggatcaccaggcaggtctttaattctaagacacatataacccaattgctaattgaagtaatataagcaaccctatcacatcatcatgatcataacagggtatcagacaccaaaaccgtaatttcttactttctaagaatacaaaacgcgataattgatatgaagctgcaattggcatctccaatagaaatgccaagaacactgtaacatacagatcagatgcatattcaagcattggccaccatcggttcatggaagtcatcatgagggcttccattttcacttcttgaagttccttcactggcttccccagcatctgctccatcctttattggctcatgtttggatgttgaaccaaacatatttctcctggcatctctgcgtgcagccttcttttctaatttgagttctttgaattgttcttcgaagttggcctcgtcctctggagaaaagaaaactacctccatcttcccaaggtcttcatacatcttctcaaccttagttttccagaagaggcccatttccgtgtccatcttatggtaaggtgtcttcagcaagtactcatcaattccacctgccttgtctatgcagcgcagtgcatgagtagtaaccttaacacggatgtgacgatccaaaatgtagctgaagagccgcttctcctgtacattaggtttccaagacctccttgtcttgttgcctccgtcttcactgacgcgattcccgaattggatgtgtcgtcccgcgaaaagcccgcggtgagctcggcccattacaattttgcgatccggcaccgattttttcaaatggtctttcactcccggcgctaaattctgctcgccgccgattttcttaacaattttcttcatcagttctcgtgacctgaacgccattcttgtggatttctttctctctctccagttctgtatttcgcggttatatagattcaaaattggatttagggtttagtctctgatttagggtttttgagggaggaagaagctctgataatgattcggaaatggattttgaagaaataaaaatgcaactctcaatttcaacatttgttgaaatgaactttcaaaaattagtgaaaaatagtggagaggggcca

>comp12359_c0_seq1 cbl-interacting protein kinase 6

gaaacacgtcgaagacgggtgaaccgccatcgcagccaacatccacaaccacaactcacatcttttttattttattttagttttttaatctttttttactaatcattagcaaaatccttttaaatcttgctctcactggggaattaacgacgatgggatccgaagaaaaatgcggcgtgctgcatggcaaatacgaattggggcggcttctcggtcacggtacgtttgcgaaagtgtaccatgccaagaatctgcagatgaataagagcgtggcgatgaaagttgtgggcaaggagaaggtgattcgtgtggggatgatggagcaagtcaagcgagagatc

>comp12450_c0_seq1 dihydroxyacetone kinase family protein

tgctctaccacctccaagaggcatagctggaagaagaggtttagctggaaccattcttgttcataagattgctggggcagcagcagcagctggtctctctttagaagaagttgccgctgaagctaaacgggcatcagaaatggttggcacgatgggtgttgcattgtctgtttgcacgttgcccggtcaagcgacttcagatcgtttaggccctggaaagatggagcttggtcttggaattcatggagaaccaggtgctgctgtggctgacttccaaccagttgatattgtggtctctcatgtccttaagcaaatattgtcaatggaaacaaattacgttcccataacacgcggcagtagagttgtactcatggttaatgggttaggggccactcctcttatggaactgatgattgcagctggaaaggctgttcctagactgcagctggaacatggacttgctgttgagagagtgtatactggttcatttatgacctctctcgatatggcaggattttcaattactgtgatgaaggcagaccaagcaattttgaaccgcttggatgctccaactaaggctccaaattggcctgtcggtgctgatggtaaccacccacctgccaagattcctgttcctcttccaccatctcgtttgaaaaagaatgacgagacattgtgtcggcccgaacagctaagtcctcagggtcatattcttgaagtggctattgaggcagcagcaactgcagttatgaatctcaaggacagtttaaatgaatgggacagcaaagtgggtgatggtgactgcggatcaacaatgtacagaggtgcttcagcaattcttgaagacataaaaaaatgttatccactgaatgatcctgcagagactgtcagtgaaattggatcttctatcagaaaagtcatgggaggaacaagtggcatcatatatgacatattctttaaggcggcatacgcacatctgaaagcaaacagtcattctggcattacagtgctacactgggctggtgctcttgaagctgctattgctgcagtcagtaaatatgggggtgccaaagcaggttatcggacattattggatgcccttattccagcatcctctgtccttaaagagaaattggctgctggagaagatcctgttgaggcatttgttttttctgctgaaacagcagtcactggcgctgagtcaactaaaagcatgcaagcacaggcgggacggtcatcgtatgttgcagctgatattctggcatcagttccagacccaggggccatggctgcagcttcttggtacaaagcagcagcattagctgtaaagaataactacaaggcttcatgagacagatggtctcaaacattccatccttccaccttccccttttttgttctcgaggagggaatttttacagagctaaatttttgttccaaacgctttcctaactgatcagctttccgcttatgcagttgatgcataaagcagagagagcgtaaaactcgtcccataccaaaatctaatggatcatgtgtttcttgaagttctgatttcgaatgctgcatttacttgttagctggaactatttcttgattactcagctgcg

>comp12450_c1_seq1 -dihydroxy-2-butanone kinase-like

tggtagagcacagtcatcaccaacaatcaccatctccactttataaccttcagatttagcctgctcagctgccaaaccaaaatttaaccggtcgccagtgtaattcgtgacaattagaaggcacccgggaggaccagtcacagctcggatgcctgcaagaatggagtcaacaggaggagaggcaaaaacatccccacagattgctgctgtcagcatcccttctccaacatatccagcagtagcaggctcatgaccacttccacctcctgatattacggctactttgtcgtatgtcccaccagaaacatcagcacgtaaaacaactttgacttcaggaaaaccatccaaatactgcaacccaggataggtttcgaccagaccctcaatgaattctatcacgacatcgtttgggtcgttaatgagtttcttgctctgaaaatccattgataaatgcgagtgtgcgctccaatgaacaatccctaatgctgaattagaaaaaccccacttatttttttcctagtgtgagagagagagagagagag

>comp12676_c0_seq1 prohibitin- mitochondrial-like

atgcttaagatgtagaaatgtaatgccggaaagtgttacaattgaaacaatatctttggaaaatattgccctgctttttgaatcaagatatttacctgatatatgcaaccaaagtgtcatcagacatcaaaataaactaaagacgaagcagaaaaattaccaaatgagctaccatcctagggctacttgctgttcaaagaaacaccaaggcaatccttgaaacagcagacttaagtttagtttattaaggctgtcgaaaatgcggccttttatgtaactaatggcaatcccacatcaggaatctgtgggatgaagtctatattatcaggtgaacaaattcattgcttcttgctccgcggggaattcagatcgcgaaggtttagcaacaactcatccgagtttaagtacaccttgttcgttgaagctgaaatggtgcgtgaaatttccttagatgcttcaactctcctaagtgtaatgaatgcaggattgttggcaatggcttccccaatcagctgggcactcttagcctccccctgtgctctaattatagcacttcgtttgtcttgctca

>comp12694_c1_seq1 coenzyme q-binding protein coq10 mitochondrial-like

gaatttccaaaaacccagttggaattctctcatttccaccaaattctcaacagattcgtatctccaaaattccatgacagcaattcgttagggttccaattgaagcccaaaataatagtcaaaatccaatcttttttttgtccaatgtctccattccattccgtatcgaaggcggtgacgcgcttggtcacccaccggaattcattccggcaacggacaagaagttttccgagttacaatcagattcggtgtttgagtagcattgccggcgggactaatgtgttttctttggtggataaaattgtaggcggaggtcacagggatcgcagtttcctggattgtggaaaatcattgaatattggtgatgttttagtccaaaaaaggggatttttgggatgtggtgatggtgaagagggtaatatgttatcaaaagtacacgaagaaagacggattttaggatattcaccagagcaattgttcaatgtggttgctgctgtggacctgtatgaagattttcttccttggtgtcagcgctcacagataattcgccgcaatcctgatggaagttttgatgcagagctagaaattggctttaaatttctcaatgaaagttatatatctcatgtggagctaaaaaggccaaaatccattaagacaacttcgtcccagagtagcctttttgatcatttgatcaatgtttgggaatttaatcctggacctgttcctggatcctccagcgtctattttttggtggacttcaagtttcaatcaccgttttatcgacaaattgcaactatgttcttccaggaagtggtttctcggcttgttagttcattcagtgaccggtgtcgacttatatatggacctggagtgccagttcttgaaaatgcttatgagcagagaacatgaaagtttatgaatgtcttgatcatcttgttcaaacccagtgcctctgaatactatctctggattttcatcaacagaagattaattctaaacaagataggagcacttgcctatgttctgggttgtctacttgcaccatgttcctgttgcctaggagttttctcatttactatttgaatgctggaaaagttactatctgcaccgatgctggaattcttttggatcggttgtatcgatccatctttcaaacaaatacatcgtctttcttttgtttttccatgtggtcgttgagagaagtgctgaaaagggtacatgcgtgtacatcatgaaggatgaattactaaggcttcactgccttaatagaactactgtattctagtttccatcaatgatttctatttccatagctgatatcttgctcctgctgtatctgtcttgggctgcagcccatgggccataaatcgagactgcctcgtgtt

>comp12895_c0_seq1 histidine kinase 3

gaatctgcaatgcatttcatgctttctgaatggatctccgaagttgagggcactaacatgatgcagcccatcagatgagctggttgaaccatacatgctaataggatgggaggaattagtagtgtcgtacacattgacaaggatggtctgtttgctagcgagttgctgaagcagtttttccacaagtgattcaatatcaaagatgccaccaaaataccctgcagttgcttggatcctttcggctggggttgcactaagcagaagatctcttttgtagacagcaaaagttagtattactccaaggcgatttgttttgagtaacctaaacggagcagtgaggacaccttttcctgattctcttgctcgcagtacattatcgcgatcttcctttccagaaagcacgtctatagaaattatgtgagcaacagtatcttgagcaaag

>comp12945_c0_seq1 glutathione s-transferase

ttgttgccttctgatccttaccacagagcaaaagctaggttctgggctgattttgttgacaaaaaggtgtttgatgctggaagaggaatgtggacaagaaaagaagaagagcgagaagctgccaagaaagaattgattgatgctcttgagctgttggaaggagaactgggagacaagccttactttggtggggtcaactttgg

>comp13030_c1_seq1 protein iojap- mitochondrial-like

aaaaaggttcatcccaagacctacagtatttactggagcaaagaattgtaatgcctttgtgtaatgaggcctatactatgcgcatgtgtgtggacgtttcttggagttattcttccggcgaaccttgacaaatgccttgtccaaatcctgagaatgttctgtgcttgatgtgtttgcagtccaaagctcctcaaaattgtaataagctcttgccttctcatcaacagcatgaacaatcagctttccagagtcaatgacaatccatttgcctccctcttgccccaccacactaggcagcaacatcctttctgctcctttctgcttttgcttaaccttgtaaattagggcttgagagatattgcgcacgtgccatggagatcgaccagtggcgatgaccatgcaatcggtgaattcacaacgttcgggggctcgaataactttcacatcatccgctccgacgtctttcagtattttctcaacttccgacaaggtcagcacgtccttgttcttctcgacgctgttgccgtccccattgttaacggcggatgaagagaaatggcgagcgaaaatggagtgagaagaggctaggtttttccactgatgagtgacggcggaatgcgaaggggatagagctccgccgcgtaaagctgaccacattgtatcgccgactgtcgccgatctttcgcttctagagattttcgtgcgacaaccggaa

>comp13169_c0_seq1 cytoskeletal protein mrna

aagcaccatcctcaatcgcccagcaatctccaaggcatcaccatgattaggatcagcaaccaacaatgactgcacatcttgcatagccatttcatacttccctattgcctcaaatgcacgagccctcctaagaagggctcggacgaaactcggctgcacctgaagcgccaaactacactcagatatcacagtttcatagtcaataggcttcatttgcatcaaacaggcagctctattgctgtgaaagacagctcgatcaggatgcatttttggagtaagcttaagggcattctcgtattgttgaagagcaccaacataatctttggcctgaaaccttctgttaccctcttccttgagttcatgggctctttt

>comp13177_c0_seq1 phosphomannomutase phosphoglucomutase-like

aaagatggcagccctataaacggcgacaaacttatcgcgcttatgtctgctattgtgctgaaggagcatccaaatacaactatagtgactgatgcgcgtacgagcatggcgctaacgcgtttcatcacggacagaggagggcatcattgcttgtacagggttggctaccgcaatgtgattgataaaggagttcagcttaacaaggatggaattgaaacacatcttatgatggagacatccggacatggtgccctcaaagaaaaccattttcttgatgatggcgcgtatatggtggtgaaaattatcattgaaatggtgaggatgaaacttgaaggatcggaggacggcattggaagcctaataaaggatcttgaagagccactggaatctgtg

>comp13390_c0_seq1 glutathione transferase family protein

tttgtcgatgtagtcagcccagaaccttgcgtgggcttgctcgtagggatcagaaggcagcagaggagctttgtccctccagatttcatcgatgtattgaatgatgatcatggactcaatgacaggcttcccgttgtggatgaggactggtatttgcttatacacagggttcatctgtaggagtagtgagctcttgttgctcagatcttcttctatgtagttaaactccaatcccttctctcttagagctattctcacccttaaaccaaaggggctactccagagatcaagaagagtcaa

>comp13393_c1_seq1 glutathione s-transferase

ggagagcatgcttgaagcaaaaaaacgcaaaacaagaacaaatgtaagcatacctgctgcgacactgggagctagcagaccgatcagaggataaactgcctattattcaaagccatacttcttcttgtacatcaagacaaatttgtaaaccttcctaggatcggccaaggacttggagacactctctttctccatgcaccttttggcccatctgatcagtttggggcagtgctcttcaat

>comp13416_c0_seq1 calcium-dependent protein kinase 1

cgaacgctaccccaatccctacgcccaatcccccgccccgatccgcgtgctgaaagacttctttccccaaacaagaatttccgacaagtacatcctcggccgcgagctcggccgtggcgaatttgggattacgtatctctgcaccgaccgcgagacccgtgaggcgctcgcgtgcaagtcaatctcgaagaagaagctccggacggcg

>comp13736_c1_seq1 histone-lysine n-methyltransferase eza1-like

atggagataagacaggtctgccttcctcatcggatgctttatgcccgtctagcatatctacttcggagcgcttgggcaagtctttaaaaccattcaactgaagataacattgatctccacatggtttgcggtcctcttcaggatcagatgggtaaggttgcttttcattaggataaactagagcttgtgaacaaccatgcaaacggcagtcaaacaccagacaacggcggcaaaatagattatcaaaagaatctaaagcagcactgaggctttggtctaggaatatatcatcttcagattcctcttcctttgaatgtttcttctgtttctctatttgttgatccttttccataagcaaattgcagcgctcctggatttccagggaggatcctccaacaaactgggtcaacagatcaagaacttcattgcccattccatactctttaaaagccatactcataagacgatcttctccctcggaaaattcacgtttatcaccctctggttgccccagttcttcttcactgtcactacagatcagtgcctcactaccatgttgatcatagta

>comp13930_c1_seq1 starch phosphorylase

agaactggaccaagatgtctatccttaacacggctggttcttccaagtttagcagcgataggacgatccatcagtatgctagagacatatggatgattgaacctgtcatgttaccataagatagattaatattaccataagatagattaataagtagagcgtgaagccagcctttaatggtaccctcccacgatattactcagtaaattttcttattatcgtgactaaatcagtataaatacagagaaatcgccctggtccttgaatgcaatcccaaactctttctatgcaaatatgtattactacag

>comp14084_c0_seq1 universal stress protein a-like protein

acaatgcacaatccagaattcttcaaatctaagtctcaaaattaaattccctcaaaaaaaagaaaaaaaaaattcaagaatgtcgataatggaagaaaataaccacatgaaggagaagaaagtagtggtggcagtggatgaaagtaaagaaagcatgtatgccttatcatggtgcctcaccaatcttttttcaggaaaagaccataatccaaacagtaccttaattctcctctacattaaaccgcctgttcctgtttattcctctctagatgtcacagggtatttatttgctggagatgtgattgcaaccatggagaaatacagcaaggatttggccaattcagtgatgaaaagggctgaggaagtttatagaagcttcaatagaacagtgaaagtggagaagaaagttgggagtggagatgccaaggatgtaatatgcacagcagtagacaaacttggagcagacatcctagtcatgggaagccatgattatggtttcttcaagagggctcttctgggaagcgtgagtgattactgtgctaagcatgttaagtgcccattagtggtagtaaagcgcccaacgacagcctaggatgataatcaacactatgatgatcttcttttgtggagctgtatatgttgtcatgtaaagaagtgtttacgttcttgtagtgtgtgtgtcccccccccccaaaagatcatggctggaa

>comp14096_c0_seq1 ribonuclease t2

caagtccgatatctgcaagaaaatatacgaacagaaaagtggatatctcggaagtttgtagttcgaatgcaaaagaagttgtatggtattaccttggattgatcatagggattgttgggatcacaatttgaaggataagagccatctctgtcattaggccaaagtccatggataccaaaatctgatgggggcttccccgtcgtcggatagcaacagctttgttttgtgtcacagtatgatccaggccactgctgaacaaaataaaagaaatcgaaatcttgagcggcagacaacacagagagacactgtaagagcaggagcttcatcagaatggaacgatgggaattcttgtccatttttgcggatttcttaactcagttgcagtagaaaaactcttctgattttaccatttttataggcatgaattagaaatgtgaacgaagttaatgtccgatgtatgaacataatcaatctgctacgctgtctttgaatgtgaa

>comp14105_c1_seq1 mediator of rna polymerase ii transcription subunit 32-like

gtggcatgcatcctcttcctatattgcattatgctgtctcaaaggagaaggtgaaagaagatttcatgatttaagttttaacacatggaagtgtcccaacaatatgacaaagccaaaaagcatttcatatagctcttgagcttcactcatttagtcatttgcagctgtaacagtcagagacgtgctgaagtaaaattcagtggtctctacaatattcccatgaacatgaacaacagcttttgattgcctaactacacagcaggaagactgaaaccttctttcctattgagcagcatcttcagtaaatctagcatcgaaaggagcagtagagttcggatgcgatgaaccacctccagctcccgatccttgttgcagttcaatcactagccatctgacagctttactcatttgctccaagcgaacggcactaataggaggaagaccacttggagcaggctgtcctggtttagcaacaacagggccagtggcctcatccacaagacactcagacccaattctttgctttacagattccacaaattcttctgcttgatcacaagcaactctgaacagttcccaccgttgcttgaagttctcaagtgcagcatcggtagctggagttttctgaccaccagaattctcttttgcctccaacgtactagctgccgccgaaataaactcctgatacgcattattcaaggaatctactatgttgtccatgttatgtctcgattagaattttctgcgccggaaattcagcggagtttttacggagggtttgt

>comp14276_c1_seq1 probable membrane-associated kinase regulator 2-like

agccgatcggcatccgcggctgtgggtgtaacgacgccgtcgatccgccggagagacgattcattgctggaacaacacgacggaatccaaggcgccattcttcattgcaaaaaatcctacaattcttcatctcaagaatattcgcaattatttcgagctggcagcgatccatcccacgaaaaacagagaacgccatggagaaattcatt

>comp14308_c0_seq1 non-symbiotic hemoglobin class 1

ataattttcaaacatggctgctctcgtcctaggtcattttctatctttatctcatattttatttaatcattgaaccttattatgagttggtcaattttgggtcaagggttacttttcttacgagatttactaaatatcgatcgtagcacatatgtaataagtaatagagtaaattattcatcttgacaataattattgaattgaaaatagaaaataaaattaatttcaagaaggaggcttcatttccatcttgattgcagtcgccaactgatcgtaggcttcggcccaagcgctcttcatttccggcgaccacatttcgggcactgcttcctttattgtttccagcaatgcatattttgtaacctcaaaatgttcatcaaccacaccatacttaaagtgtgtagcgcccaaatccttgagagttgaatctttcacagtaactttgccagctttccgaagttgcactgctgattcacaagtcatgacaaaaactgtgacggcatggggtttgagctttggattctgctccacaggaacatctgaatccttcaagaatgtaaaaagcttcttcgcagatggtgcaatctcaaagatcctcaaaaagaacttaaatcccaaatctgcagcattcttcttcattatattccatgattttgttaccaaagcttcttgttcttcagtaaaagctttcacttttagcctcccatttttctcaatgcatggtcggatcccaggagttttcagaactagacaacaatctgaagcatcccttcgaagccatgacaagctgatcgaccggcgatgtcgaaatcctaaggtagaaatctccttggccgcgataattgagcagtgggaataagggcctgctgagattccattgcagtgcagcttgggagcagtagatccaatgctactgatcttcaagatgctcatgattgatgataggagcaagagttggcgctcaaggagtcctgcaacagcctagaaaagcacaggaaaggagagagagagagacagaaacag

>comp14486_c0_seq1 dnaj homolog subfamily c grv2-like isoform x1

ttgctattgaatttaatgatggatgtcctattcatgtgtatgctagcacctctcgtgatagcttactcgcagcagttctggatgttttgcaaaccgaaggtcaatgtccggtgccggtattaccaaggctgacgatgcctggtcatcggattgatcctccttgtggcagagcttatttacaatttccccaacagagacatcgccctgttgctgatatggaatctgcaacaatgcatttaaaacaccttgcagcagctgcaaaagatgctgtagctgaaggaggctctattcctggttcaagagctaaattgtggcgcaggataagagaatttaatgcatgcattccttttagtggagtcccccccactattgaagtgcctgaggtgactttgatggccttga

>comp14493_c0_seq1 kda class i heat shock

agtattgagagatttcacatatcaggcagctaggcaaaatttcacagcaatcgtccaaaaacaccatagccaaaaccccaattcacattatagatacttacactactatattaacaaacgcaaacatcacagcagcaacaaagttgagaaccatactcgaatccaattgatctgatgcagacaaaaatagattatccagaaatgtcaatggatttgatatcgggcttcttgctgtcaaccttcggaacagtaacagtcaaaacgccatcctccatactcgccttaacctcctccagttttgcattttccggcagcctgaaccgccgcacaaatctgccgctgctgcgctccactcgatgccaagtatcgcccttctcttccatctcccgcttccgctggccgctgatctgcaaaatccggtcatcttccaactccaccttcacctcctcctttctcaggccgggaacgtcggctttgaacacgtgagctttgggagtttctctccagtcgaccatcgcagtcgcgaacgacgtagtttcggaggaggaggggaaattcgaaccgaggttgccggagagttgccgaaagtcggaattgaacggccagccctcgaatgggtcccatgcttcgagcgagaacggatcgaaaacgccgttggatctgcggccgaagaactttggaatcagcgacatatttctagccaattgatttcgatttttctgctgcagtgatttttcagtggatgaatataaaggtggaattgggattttacgtcaggagttggttcgagagagttctgct

>comp14792_c0_seq1 ferritin precursor family protein

acagtactccacattgatctgctcgttaagtgcagcctcacacgcttcggagaacctctgccgggcgagtgaaacattaggagctattggaatcataagctcatccttcttaacctcttcaaatggctgaaacaccacaccagtaagtggcatggtcacagcgttattcgacgaagtaactaccacaccattgccgcgctttttcgccgaaaggagaccagaaaaggaagatgaatggtgtgcacctgacaagggcccaagactagcagaaccctgaaaagtaggtagagatactgctg

>comp14803_c0_seq1 SNI1

tagatggatttgtcattgatacgtatttctctttgcgtacaaatctcaaaacaagtctacaaatattaaacaagttctacaaatatcttctagcacaataaaaggatatccttacacgtagattcgtgattcattgttctctgtaccattacagagaatcatttctttcgaccgcactgggttgtgttgatccttaagcagagctgtggtaactggatcttagcgacaaaaaaatccctacgatttggccgagaggatggtagcagcagtaatcagtgcgtccttgccaaagcatgaaacctcagtgcccggatcttcttgtctgagacaagtaaaggcagatatgatgtttttgcaaacttccacaacagagtcgcttttgacatcttgcatcatatcagacgagccttcagttacatcatatgatagcaccacatatgcctaaaaaagagatcaaggtcagaaaatagagcagagcagtggtttg

>comp14806_c0_seq1 heat shock cognate 70 kda

atcaatgaaccctctgctgccgcccttgcatatggtctagataaaagaagtggcggctctcatggagaaaagaatgtgcttgttttcgaccttggtggtggtacctttgatgtgtcttttctaactattgaggatggtgtgttcacagtcaaggccatagctggtgacactcacctgggaggggaggatttcgataatagaatggtgaaccactttgttcaggggttcaagaggaggcataagaaggatattagtgggtatccaaaagctcttaggagattgcgaaatgcctgtggcagggcaaagagggacctttcatcgacca

>comp14869_c0_seq2 heat shock protein with tetratricopeptide repeat isoform 1

ggagcatttgaaactgttgtataattcaatgttgcgcgacagaaaattgccagggcctgtttggaagcgacaaaacgttcagtacagagaaattccagggaagttgtgttcgttggcgtcgaaaatccagcagttaaagcaaagggttgcagctggtgaaactggaaatgtagattattatgcattaatcgggttgcgaaggggttgctcgagatcagaactagaaagagctcatttgctgttgacactaaaacacaagcctgataaatcttcaagtttcatagaaaagtgcgagtttgctgatgagaaggacatggattcagttcgcgaccgggcgaaaatgtctgcattgttgttgtaccgattgattcagaaggggtacactagtgtaatggcgacgatcgtggacgaagaggcatctgagaagcagagaaagaaagctgctgcagccttgcaggctgcagttcaagctcaacaagctcatgagcaatcgtctctcgccaaaatcgagacgattcaggtggttattgcagatgtggctggcggtagtgataataggattgagaacaaagctgccgtaacgacaccaccaacat

>comp14970_c0_seq1 universal stress protein

ggtccttactgctttttcccgccgtagtgacaaaaactcgtgacaacaagactaaaatcacgagaaaaaagtaaaaaagaggaaagagaaagaggaagatggggaaagacaggaaaatcggtgtggccatggatttctccaagagcagcaaggcggcgctgcaatgggcgatcgataacttggccgacaaaggcgacacgttctacatcatacacgtcaaatcgcactcttccgacgagtcccgccatagtctctggtctcaatccggttctccgctaattccattggtggagtttcgagaaccagaggtgatgaagaagtacgatttgcctactgatattgaagttctcgacattcttgacaccgccgccaggcagaaagagataattgtggtaacaaaactgtattggggagacgcaagagagaagctgcttgaagctgttgaagatttgaagctggattctttgattatgggtagcaggggactcagcaccatcaagaggataatcttgggaagcgtgactaactatgtgatggcgaatgcaacttgcccagttaccatagtgaaggatcctgactttcacaagaagtgaatgtggggataactatgttcgaacagctcatgaaatcaccttttcaaaaaattggattctctctttcttacaataaaatggtttagactgtgagaatcttttgtttgagtttcataaagttgaatgtattacagctcaactttgaattctagtattgtatatgttcgacttgtttgcttcatatgttttggttgtgaactcgtacgtctttgtgttacatcgagaagattcgaactcatacttttgtgccggggaagacttgcaatcatgaattactgctagggttgctgagattgatccaggcaaagatctctagtgcttaaaatataaacgggtactggacaaaatttccaatgcttaaaattggatcgggaaatatttattaagttgtgtaaaatagtaagattcatatgaagaataaattatctagtttcatagatgctatatttagtgacgtatattaggtgcagtatttattatgaatttacatttgtatagatggatatgtgtgtggttgtgtgacattttgggtggagtgctccgatgaccttaaatgcaaaggtaatgtgattttatgttggttttactcaattatgtgctaaaacatgcat

>comp14976_c0_seq1 chaperone -domain superfamily isoform 2

agccatagtttccaacatcagcagtaatgatccaatcaagcactaattgctcatgaactaagtgactttcttgaaccaactgtatacccttgaaatttctgcttcaactctatccttaattgtctttttccctcttctcacatcttcaatatctagagtcctataggccagagcaagcaagtacattgctggatgaagtctctcctcaaaatgttgatttgtatccaaaccgtcccagtatctctgggctggaatagtctttcctttattatctcttccgaatgtctcaatgtaccatcctcctatatcatgcattgcactgcgcggctcaaatagccaatatttgcttggtggtaaacaggctcgactgcatctgcattcacaaactgaaattttctcaccaactcggaaccagtgcaagtgtttcaccagcaaagttctatgcttcataatcatatgcgtcttcatgccactgttgttgtagacatagcaagttccttcttcagaggtggttcccaaaccttttataactcccaggggaattctatatccaactgagggatctgtaaacatatctccagtgtgtggatgccttgcatgcaatcgatctccacat

>comp15032_c1_seq1 dnaj heat shock n-terminal domain-containing isoform 1

tgggaaaaaaagaaaaaatactcaacaatttacctcttgagcagtgaaacgatcctgttttggaaatccaatctgaaaggaaaatttggggcaaattcgaaataattggaattattttccagtgatgaaactctgcgcttcgatttcatagagacaatacgaatttctctcgcatacggcaaccctcgctctactctacgagggaagtggtggagtgaggggcaaagggtagatggagtgcaacagagatgaggctttgagagcgaaagaaattgcagagaggaagtttctggcaagggacattaagggggcaaaaaaatttgctctcaaggctcagaatttatatcctgaactagagggcatatctcaaatggtgatgacccttgaggtttatatttctgctgaagaggaaaaattacatggagaatcaaattggtatggtgttcttggtgtgacttctctggccgatg

>comp15182_c0_seq1 nad -binding rossmann-fold superfamily protein isoform 1

tggtgattgctggctcacaagagagccgctttccactgcattaagacatatagataaatacactgaccactcggctcttacttcgcaagaattcggaagggttggagaaatggcttcactttcttgcatcgcctccatttccgcagcttcttcacaatctagcagagccctctttgcctccttttgtgggacccctcattttgtcaatttctcttgccatcgatcatgcagggttgctgtcaagtgcagttacacagaccctggcattaaggaggattctaatgtcagtacgattgatgttgtggctgatgttaagactgaacgaattgtagtattaggaggcagtggctttgtgggttctgcaatatgcaaagctgcagtctcaaagggtatagaggtcattagtcttagcaggtcagggcggccttcttactctggctcatgggtagatcaggtcacttggctgacaggagatgtcttttatgcaaattgggatgaagtgctaggtggtgctacagcagttgtttccacacttggaggttttggcagtgaggaacagatgcaaagaatcaacggcgaggctaatgttgtggctgtaaatgctgctaaagaatttggtattcccaagtttgtattgatctcagttcatgattacaatctgccatcatttctactctcaactgggtacttcactggaaaaaggaaagctgaatctgaagttctgtccaaatatccaatgtcaggtgttgtcttcaggccagggttcatatatgggaaaaggaaggttgatggattcgagatccctcttgatctgataggggaaccaatagagaaagttctgaatgctatagagaacttcactaaacctttgaactctctgccggcctctgatttacttttggcccctcctgtcagtgtagatgatgtcgcatttgcagtcataaatgccatcactgatgatgatttcttcggtgttttcacaatcgagcaaatcaaggaggcggcagcagcagtaaaggtgtgagactgtgtgttaggtcctattgtcgaaactcaacttgcatgtcacagttcacatttctgattattagaattacagccatgacagatatgctgaagaattaaaaccatgtccagtattcctgttttttctttttaatgacttgcaagttaaaaaatataaatttcttgtaaatacacacatatatggaaag

>comp15182_c0_seq2 nad -binding rossmann-fold superfamily protein isoform 1

tggtgattgctggctcacaagagagccgctttccactgcattaagacatatagataaatacactgaccactcggctcttacttcgcaagaattcggaagggttggagaaatggcttcactttcttgcatcgcctccatttccgcagcttcttcacaatctagcagagccctctttgcctccttttgtgggacccctcattttgtcaatttctcttgccatcgatcatgcagggttgctgtcaagtgcagttacacagaccctggcattaaggaggattctaatgtcagtacgattgatgttgtggctgatgttaagactgaacgaattgtagtattaggaggcagtggctttgtgggttctgcaatatgcaaagctgcagtctcaaagggtatagaggtcattagtcttagcaggtgtaagaatttctctccgagaagctgaataacgatttcttttttattatgacagtaaagtcctatttttaaatatcttacaaaattactcttgacataatgaatgttcgcagtatgcacggcatcagaatgaaag

>comp15215_c1_seq1 kinase family protein

gaagttgccgaagcccattttgcaagcccaaagtcagaaagctgtggctcaaaatcatcagatagaagaatatttgatgacttaatgtccctgtggattacaggttgaccttctctcttgtgaagataatccaaagcctcggctacgccaatagcaaccctatatctctcgctccacccgaatgccagtggttcctttttattaccatgaagattctcttcaaggcttcctctcggtagaaaatcatataccaagagaagatgattgtcctcaaaacagaatccaaagagtgagatgatgtttttatgatgtaaagtggtaatgatctcgatttccaggacaaactcttttagagcagcctcagatggctttaaaatcttgacagcaagttcctttccgtcaggaaggcagcctcggtaaacctgactgctgcctcctttacctatcatatttcctggtgtgaaattggatgttgctgattctaattcctggaatttaaacagtcggcatgtggccgagtacttct

>comp15233_c0_seq1 histone-lysine n-methyltransferase eza1-like

attcattcaggaaacagaatcatggtctcgaagtctaaattcaggaagtctcatggggaacaggggaatgatgctatggcaagcttggctaataaacttacacagctgaaaaggcagattcaatctgaaagagttgtttcagttggagaaaaacttgagcaaaatagcaagaggattcaaacttatgttgctcacctcgaagatttggctgcatcaaggactgattgtacagttacaacaagtaatagttcaggcaatttcctttctttgagcatgaacaatcctctctgcaaaattggcgggcttattcacggatctgagagcagagatgatgataatagtgaagttgttttttctacaactgccaaacttccgcttattgagaggatacctccttatactacttggattttcttggatagaaatcagagaatggctgatgaccagt

>comp15336_c0_seq1 monoglyceride lipase-like

ggactggattggacatggtggaagtgatgggctgcacgcttacattcattcacttgattatgctgttagtgatatgaaaatgtttcttggcaaaattttagctgagaattcaacagtgccctgcttctgctttggacactcaactggtggagctatagtcttgaaggcagttcttgatccaaaagttcgacaacatatagctggcattgtcttgacctcacctgccgttggagttcagccatcccatccaatttttgctatacttgccccagtttttgccttcttgttgccaagattccaattcagcgctgcaaacaagaggggtgttgcggtctccagggatccaaaggcactgttagacaaatattccgatccacttgtgttcactggatctataagggtaagaacaggctacgaaattctacgaatcaccgcccacttgcagcaaaacctgagccggctgacagtgccatttcttgtcctccatggcacggatgactcggtaactgaccctgaagcctcccagaagctctaccaggaagcatcctcgactgacaaaacgatcaagctttacaagggattgctacacgatctgcttttcgaaccggagaaggaagatgtcaccaacagtatcatcacatggttgaatgctagactgtgaggttcatg

>comp15573_c0_seq1 cysteine proteinase inhibitor

agcataaacattaactgatatgatattcttcctacttaacagtgatcagcgtccatcttgttcaaatggaatccaccttcgctattcctatgcacctcaaccttgaacttctcttcttttcctcctcttttgaccttgagaagcatgtcaaatttagccaatgtctcagcaacctctgcattagcatggactatctcactaagttcataagggaataacgagttggatctctcctggattgtcttgacagcatgatgagcggcatcttgaacaacaggatcatgcactggcactgattgccattcagacacctgttcatctttcttaacgcccaggtctgatgaggtaaaggaaggaacatctccaacatgcttgaattcttgaagttccttaaaattcatccatggttttacccaaacctttgcctcataaagtttcttcttcccagcatcaaggacctcaagtgtaagatgatgaagtgtaccggagaccacttgttcttgcgccttcacaaccctcaccagctcaagtagtgcattctctttggtgttatgctgatcgacagcgaatcgggcgagggagtcgatctcggcattggactcgctcaggcctccgagggtagccatctgaatatcgtcgttgtgttgttgttcggcgcaaaaccctaagttccgttcggaattaatgggaaccactgatgaagaagaaaacaggaggaggaggaggagggggaggaagaagaagttggtgcaggaggggaaacgcaatgcaaaagcgaatactctcatttgatgtgattttagaagaaaacggggggcgtatacagaaaaagtagtggttgtttggcaggactacaat

>comp15791_c0_seq1 hsp20-like chaperones superfamily protein

caattatttgcatcagccaccctacccttctctctctataaattcgatctctctcctcctcactttcctcagggtaaatcaattcaatcgtacagctctctagaagttaatcaatctctgcagtaatattgcaggaaacatgtcgctgatcccaagtgttttcggtggtcgacgtagcagcatctgcgacccattctccctcgacgtgtgggaccctttccgtgattggtctatgagctcctccgacgagacgtcgcagttcgccgccacccgcgtcgactggaaggagactccggacgcccacgtgttcaaggcggatttgccagggcttaagaaggaggaagtgaaggtggaggtggaggaagggaatgttcttcagataagcggcgaacgcagccgtgagaaggaggagaagaacgacacctggcaccgcatggagaggagctccggcaagttcctccgccgcttccgcctgccggagaatgtgaagatggatcagatcaaagcgtgcatggagaatggggtgctgactgtcactgttcctaaggaggaagtgaagaagcccgaggtcaaagccattgagatatctggttgaaaagtctgtccctgcgcttgctagtcttatatacatatataacatatatatatatatatacatacatatataacatatatatatatatatacatacatatataacatatatatatatgtatcagcatgtttatgtgtgcaatatgaattgtgtcgtctttgtattgttggttttaatgaattgatggtttgattttacactgcttctataatgtattacatgaaaaattatagggagtggcacttt

>comp15821_c0_seq1 conserved hypothetical protein

gcgacttggtgaaggaaacaagccaactgctatcatcggagaggttgctgatgaaatgaatatggatctggttattatgagtatggaggccatccattccaaacatgtagacgccaacctgttggcggaattcatcccttgtcccgtcctacttttgccactatgataaatacttttccatggaaattagtattttgagttctgatatgtctgattgttggaaattgttttcctcttttcccagttttaatggaaccatcgacgtatcaagctagccattttctgttgtcctttaaatcggtggagattaaaccaattagaacagaggccctttctttaaatttccaatagcagtggtttgtgtaattatactatttggaaatttagaattatttttattt

>comp15821_c1_seq1 hypothetical protein PHAVU_003G266300g

gataaatgccaccgaatattggttagttgagtttcatgctccgacaaattctccttctccttttcgtcgataactacaactgttatctcagccccatatttctttgccatagcagcagttgttgatgttgcctgctttgtaccatcagtgagataaggattacgatctatgaccgggagaagcaactgcttgaactcagtaaaggtatctgcaagagggctgtcttcagattcgtctggcttcgatttcacttgaaacgaggttctcaagatttcagaagaaagagaaaatttgcgggttttaagcttccgggaacggggaagagagagaactggaagcggtggggtgagaatcgacgcagtggtaaagaggggttcagcagtttcaggcggaagcgcgagggagcatcgggcgagggccatgggagaacaggaggaggggct

>comp15835_c0_seq1 PREDICTED: uncharacterized protein LOC102587732

aaaaattctaatgccatgaacggaagggatgccgaaatgatggaggcggaggcggcagcgccgccgtctgctccgccgcgggaggctggttctggttctgaccctaacgatgtcaggaatttgctgacgacggctcgccaactcgttgatcaagggaagccttcgcaggcccttcaggcggtggtgatggcaatgaaaatacaaggtggagatgaagctgtatctggagctctaagtcgtgctcgagagctctatgtgaataaaattcaagctagtgctgctgctgatgagttggctacactctttgctgaatgtgcaattgcggaggctgttcctttgcagcctggattaactcaacataacatgaatgagcagccagttgaacctgatcctcaaggaacttcaatcctcgctgagacagggaggaagcaggttgtgctcgatgcgttctccgatggaagcaggttgtgctcg

>comp15941_c0_seq1 endoplasmin homolog

tattgtgccatataattcaaattgagtagaaaaaaaggccccccacaaagccaacctccagagttcccatagacaatttatgcatatcggaataatctgcagttgcgacaacaggccacttgtacattttcaaactcctattagcggaaagacgaccatttcaactcatcaactgcaaggaagctgcaataacagcgaaaaatgtctctataagaaacaacagtgctttggctaccaatgttccagtaatttatcttcgctaaattttctgcaacttctatccctcatttgcacatcaatcagtcaccacggtaatatcagaacactagctccaccaatctaatatgcatatctgtgagatggcattgatgtatgatagcccttcacctatcatttttgaccaccggcttcagcaggctcaacaacctcagcttcaactgcctctgggaaagatgtagttgggtttacttgttcctgaaacccatcagcagatgaaccccacttgcccaaaagtgccatgttcatcatctcataaatctttccaccaagctgtgcaggactctcaggagtgaaaccactagaaattaaagctgtgtcatacaaaagatcaatggctctcaaagcttcttcatcatttggactactcctgcaagcagcatttaaacttttgatgattggatgttctgggttgatttcaaaaactctcctgcttctcataaactcgaggctggatggatcaccaactgtttgtgccttcatcagcctttccatattcgcagaccacccaaacttcccagatgcaagaacacaaggtgaggtgctcagacgatttgagacttgaacattggcaactttatcacccaagcgttttttaatccagtcacaagtctggccaaactcttgctttatctctttttccctctcctcatccttgtcccctagatctaaatcttctttgctgatgtcaacaaagttcttgtccttgtatgatttcagattttggacggcaacctcatctatcggatccactaagaacagaacttcaatatccttttcaacaagtttctccaagaagggagtattccttgcactagctacgctgtcagcagcaatgtaataaatatctttctgctcagccttcatgttctcaacatactcatccaagctaatcatatcctcctcactttgagatgagaaaaatcttagcaatggagcgatgcgtttgtggttctcacggtcctcaatgcagcctaactttaagtgtttgccaaaattctcccagaatttcacatagtcatctctatcctcactcatagttatgcccaaaatcatgtcgaaggccttgcgcaccaaacgtttcctcataatgcgaacaatacgactttcttgaaggatttcacgtgagacgttcaatggaagatcattcgagtccacaacacctttgatgaagctcaagtaccgcgggaacaattcaccatcaaaatcatcagagatgaatactcgtttgacatagagccttatattctttgttttggggttgactatgtcatccttccctgtgggtgcaatagatggcacatacagaatagacctgaactccacttcaccctcagttgtaaagtgtgaagaggcaagaggctccaagtactcattaaatgttttcttgtagaactcattgtattcctcagtagtgacttctttagggttacgaagccatattggctgggtctcatttgtaagctcccaatcccaatatcgttcaactacagtcttagtcttcttctttttcttcgttttcccgtcttgctgatctttattggcttcagcaggatcctcatccacctccacctcttttgtgtatcccttctcttgccatgtgtaaattgggaaggaaacaaactgtgaatagttcttcacaagcttctggattctttcgggatgcgcaaatcctttgtcgtcatgcttaagataaagagtgagacgggttcccctcgggatgagcttagcaggatcagtctcttctcggatggtataggaactagagtttgcttcactttcccatacatattgtctatcagatttaggactcttggtcgagacttcaactctctcagaaacaaggaaggctgaatagaatccaactccaaattgaccaattaaattgctgtcagcaccagcatccttgctatccttcaatgcttttaagaatttagcagttccactctgtgcaatagtaccaaggcaatcaacaagctcctgcctcgtcatgccaatcccagtatctataattgtaattattccattatctttgtcagtctggatacgaatatcaagatcaacagcatccttcaaaagctggggctcagttacactaaggaaacgcagcttgtccaaggcatcacttgcattgctgataagctcacgaagaaacacttccttgttgctgtataaactattcacgataagatccatgagacggctaacctctgcttggtactcaaatttttcgactgaccgggttggtccatcagctgcagcagcagttgactcataacgacagcctataacaggctcattttttaagttaaacggctgggtggcaccaataggtttgctgctactagttgttagcacagagcaccatctctttgttgtatcactctcaccagacaggtgatgatcgaggggggatgatgacgaaagcgggcgatagcgcgtggcggcgctgcggaggacggcagagacggaacgccttgacaacctgtgcattttaggcgcttgggacggaaagaattggagattttcggaggttcagttgtgggctctgcaaacgtag

>comp15991_c1_seq1 encodes a chloroplast protein that induces tolerance to multiple environmental stresses and reduces photooxidative damage

cgatgctttagtacaagtaaatgaaggaaaaatccctaaaaatcgtctcgctttgcaaatgttagctgaggaaatggttcagtggcctaatttagaggttgaagcaccaaagaagaaacaaccaggcaaatccctatatgcaaaattcacagacacgggtgttgatccacaagaggctgccaagagactgaatattgattgggattcagctgctgaaattgaggatgctgagaccgatgatgtagaagtgccccctgctgtgggatatggagctctttacttggttacagcattcccggtcattattggtatctctgttgtgctgattctattttataattctctccaataaatagtagtgatgatgcatcggaagattgttacttgaacttgtacattatagttgtttataatggcagattaatgaaatcatatatgttggaatagttccagttttacttgttctgtttgggaattggtagttaattaatttgtctgtgtgtgtggtgacttgtttaaacaagtctatgcttctggaatt

>comp15991_c1_seq2 encodes a chloroplast protein that induces tolerance to multiple environmental stresses and reduces photooxidative damage

agagagagagagagagagaaggagagagagccagggcgtgcgtgttggctattgggattgcgacggtgatggtggtatccggtgtcttccaggcgccagcggtggcggtttcatcattgttctatcaccgctgtcagtccctgccgcctttgtctaccaacacagctcgccggaggcacgggaatggccgtatttcaaatctggtgttggttaacaaggaggactccccgtccacttccgcttcaatttccacccaacaagaagaggatgacccagatccccaggaccttgaatatgtctcacaaattaaaacagttttagagcttctcaggaaaaacagagatatgctcttcaatgagatcaaattaactataacgattgaggaccccagagatgttgaaaggaggcgtcttcttggcatagatgatgaaaatgcgcctaccagggacgatctagccgatgctttagtacaagttgaagcaccaaagaagaaacaaccaggcaaatccctatatgcaaaattcacagacacgggtgttgatccacaagaggctgccaagagactgaatattgattgggattcagctgctgaaattgaggatgctgagaccgatgatgtagaagtgccccctgctgtgggatatggagctctttacttggttacagcattcccggtcattattggtatctctgttgtgctgattctattttataattctctccaataaatagtagtgatgatgcatcggaagattgttacttgaacttgtacattatagttgtttataatggcagattaatgaaatcatatatgttggaatagttccagttttacttgttctgtttgggaattggtagttaattaatttgtctgtgtgtgtggtgacttgtttaaacaagtctatgcttctggaatt

>comp16029_c2_seq1 amino acid selective channel protein

caatatgcaccttctttacacattttcttcagcgagtgttgaaagtcgtggtatgaaagatgcccttgtttaaccataaagcaagtttcctgcgcagctacttttgccgccgccaccgagcccgtcttcatgaaaccagctacagctacgttgaggaaagggttccccaaatccgcctccacatcaatttttgaagtaaatactccagaaaatgtattccgacccattctttctcactcaaaaactcttgttaaatcctgaaattacctctccgtctcgtgttgtttcctcttcaaattttaaaagaaggaaggcgtgtgtttccagcagtcaagcatttggagagagggagagaggg

>comp16075_c0_seq1 e3 ubiquitin-protein ligase chip-like

taggcactgtacaagattgaaattcaactcagaatttcagaagaacaaggtgacttctaatatgccacaataagactgcactgtgcagtttaggataataagatcatacattagaacctaagaatgttttaacatgcccgatgcaacaagaattacatcgaatgcaacatccgctctttttttgccagatagataacgaaaaaccatatcaatttaagcaaaccataatacagtcaatccattttataagcccacttgtgcttctccagatatgctgttacagcttcttttatggccaaatttggtatcagctgagatgggtaaagcaattcacgggtgattgggtcaaatttgcccaccttctccagatgatcgagaatcactgctctctcatacgtaaacccacttggagctattacaggatcacgaaagatgtctaaagtgattttacagcacagatagtctggcacctcagttggagtatcatcttcagcagctttgttgaatactttgcctaaagcttccaactgttctagattagactctgcattttcatcttcaaagccttcattctgagaagcatcaggtaaatgtttctccttcagcgcttccgcgcatgcttctttcaagttttggagttcccaacaacgcttggtggattcatcttcccatgccaagtattttgctcttgcaagctcctgccatatttcctctaccatgtatccctttgggtctgcaccccttccaaggtccaatgctttttccaattgtttgacaccttctgcatattctttcctttgtagcaacgcaagaccaagcatataacaggccttcacagagtgatgatctagctgaatagcttttctacaatcctcctccaatcttgtccagtcactgctcgatcaaaataaaacagaaaactcacgaatttataaacccccccaacaaaaaaaaaaaaaa

>comp16075_c0_seq2 e3 ubiquitin-protein ligase chip-like

taggcactgtacaagattgaaattcaactcagaatttcagaagaacaaggtgacttctaatatgccacaataagactgcactgtgcagtttaggataataagatcatacattagaacctaagaatgttttaacatgcccgatgcaacaagaattacatcgaatgcaacatccgctctttttttgccagatagataacgaaaaaccatatcaatttaagcaaaccataatacagtcaatccattttataagcccacttgtgcttctccagatatgctgttacagcttcttttatggccaaatttggtatcagctgagatgggtaaagcaattcacgggtgattgggtcaaatttgcccaccttctccagatgatcgagaatcactgctctctcatacgtaaacccacttggagctattacaggatcacgaaagatgtctaaagtgattttacagcacagatagtctggcacctcagttggagtatcatcttcagcagctttgttgaatactttgcctaaagcttccaactgttctagattagactctgcattttcatcttcaaagccttcattctgagaagcatcaggtaaatgtttctccttcagcgcttccgcgcatgcttctttcaagttttggagttcccaacaacgcttggtggattcatcttcccatgccaagtattttgctcttgcaagctcctgccatatttcctctaccatgtatccctttgggtctgcaccccttccaaggtccaatgctttttccaattgtttgacaccttctgcatattctttcctttgtagcaacgcaagaccaagcatataacaggccttcacagagtgatgatctagctgaatagcttttctacaatcctcctccaatcttgtccagtcattccgcttgagatgacagagagcgcgattactccagtacacggaaacgttagggcacaaaacaatagcctcggtataagcatcaatggcggctccaaatcgttcttttttaaagtaactgtttccgtctagtctgagctgctccgcctgctccgccgctgtcgccaccactttcaaagccatttttttacttctccactctccgtagctattatcagaaaaaaaggccaaaaaacatgcagatttctagaaatatacgttacggtaagagggcgaaatcggattacactattacagaaaccaggagaaaattcgaacggaattttttcagtttctgtttattgacgggtgaaggccccgggtaaacg

>comp16126_c0_seq1 protein farnesyltransferase geranylgeranyltransferase type-1 subunit alpha

gtggtgccgattgcttacaccgacaagttcagcgaaacgatggattacttcagagctgtatacttggccgatgagcgctccccgcgtgcgcttcaactcactaaagaggccattgtcttgaattcagggaattacactgtgtggcaattcagacgtctaatacttgaggcacttaatgctgatttgcatgaagagctgcattttgttgaccagattacaagagaaaatccaaaaaactatcaaatatggcatcacagacggtgggttgctgagaagcttgggactcaagctgcaagcaaggagcttgaatttaccaggaaaatcttctcttcggatgcaaagaactatcatgcctggtcccataggcagtgggttcttcaggctcttggagaatgggaagatgagcttgcctactgtgatgaactcattgaagatgatattttcaataactctgcttggaatcagagatattttgttgtaagtagatctcctgtcctaggggggttggaggtcatgagggactctgaagttgcttatgcaattaaagccattttaaccaaacctgagaatgaaagtccttggagatatctccggggacttcacaagaatgatgtaaaatctctaagtaatgatcctcgagtggcatcagtttgcttggatattttgatggataagagggattatgttcatgctttgaacatggttttggatcttctctgtcatcactatcaaccaagcaccgagttgataaatgccattgatgctgtgtctccagacccgaatccctcaaattcaagcttagccgaaagagtatgttccatcttgaaggtggtcgatcccatcagagcaaattattgggagtggcgcaagaacactattcctgctcaagattaacacccaccatggagaaacagtgtcttgtttgaatctatgaagaatattcaagaaattacaagacaattaagcctgtgtaactgctttcttattcttgccagttgggtttgtttttcagtctaatgtaaaagtattatgtttggttgacaggattttttccatttggctaaagga

>comp16322_c2_seq1 12-oxophytodienoate reductase 3

tactcggtcagcaccaacggcagaaacaactgctcgaaccacctgcgtaatgaatttgcagcggttctcaagagatccaccatactcatctgtccgctcattgatcccatccttcataaactgatcgaggagataaccatgagcgccatggatctcaataccatcaaatcctgcttcaatggcatttatggcagcctggcgatagtcttctaccaattcaggtatttcatatttctccagtttgcgtggctttggataaatctcatatctcccatcaggaagtagcactctccacctcttggatattggcttgtccgtggaggatgctggggcaattccaccaggctggagtgctggatgggaagccctccccacatgccatagctgacagaatataacggcgcctttagcatgcacagcatccaccaccttcttccatgcctccacttgtgccttgttgaagatgcccggcacgtgcggaaaccctgcagcagtgggggagatcatggtgccctcagtgatgagaaatccgccactagtagctctttgcgtgtagtactccacaagggcggagttcggcatagtgttcagcgctctgcatctggtcatcggagccagcaccaccctgtgagaaagattgaacttgcccatcttgtagggggagaagagagatggggttgcttttgctccatcggttgtcgtttccgccattgctctgtttctctctctactactgaaagatgatggggttttgtgtttttacgtatatggagaaaaagtgggtacttactatataaaagaggccggcgtgggggtgcacgatatagaggtcggtggagcttgaccgtggacggtggtgggcggaggaggaagaggagggggttgaaatgttgaataaagtcagcaaaattaaagtagaattatcagagctccaccg

>comp16322_c3_seq1 nadh:flavin oxidoreductase

atcgagagactcaacaaactccaaacagattgtggttccagactcacttatctgcatgtgacccagccacgatatacagcctatgggcagacagaatccggcatgcatgggagtgcagatgaggaggcacgattcatgagaacttggcgaagtgcttatcagggaaccttcatttgtagtggtggattcaccagacggctaggaattgatgctgtggctgagggtgacgctgatttggtagcgtatggccggttatttatttcaaacccagatttagttctgagactcaagcttaatgcacctctaaataaatatgtgagggctacctttta

>comp16377_c0_seq1 probable wrky transcription factor 57-like

gtattgagccgattggggtggaggagaaagtagaggcttcggcttcgccgtcgaatccctcgatatcttccagctcgtccgaggatcccccggagaagtcgactgcctccggtgtctcatccgcctccgccaccgccgccaatccgccatcggacacagcgagcaagactaagaagaagggacaaaagcgaattcgacagccgcgttttgcatttgttactaaaagtgaagttgatcatcttgaagatggttatagatggcgaaagtacggacagaaggctgtgaaaaatagcccatttccaaggagctattatcgttgtacgaacagtaaatgcatggttaaaaagagaatcgaacgatcatctgaggatgcctcagttgtaataacaacatatgaaggacaacactgtcaccactcagtcacatatccgagaggc

>comp16395_c1_seq1 zinc ion binding

aaggggaaaaatttatctcgttttacataatttctcttcttccttacctattcaatttcctctgcgattctgataggtgagtatttctgcattggcgccggaaattatagtattactatcttctgatattaaagagctaaagaaatccaagaaaagtgggatttggaggtgaagaattcagtttcaggttatagatcaagatgacacctgtttgcccttttatcaaggcttctcggcctgatgatggcaagaaacctagtgaaacccaaagtaaacagcagacagtcaatgataacaaggctcagcaagatgcggcaatttcaccaaaatgcccttttggatatgattctcagacattcaagttgggtcctctgagctgcatgatatgtcaagcacttctttttgaatgcagcaaatgtgtgccttgttcccatgtgtattgcaaagcatgcatatcaaggtttaaggattgtcctttatgtggtgctgacattgagaagattcaagatgatcctaatcttcagagtgttgttgatcgtttcattgagggtcatgcaaggatcaagaggtctcaaattgacactgacaaggaggaaaaagaaggtgagaagaaaacagtaatatatgaggatgtgtcacttgaacgaggtgctttcttgatgcagcaggccatgagggcacttcgtggcaataacatagaaagtgcaaaatcaaggcttagtgtatgtgctaaagatgttagagagcaattgcaaagaactggaaacactcctgaattatgctctcaactaggtgccgttttgggtatgcttggtgattgctgtcgagctactggggatgctagttctgcaatttcttactttgaggagagtgttgatttccttatgaaagtacccaaggatgatttggagataacacatacactttcggtctccctaaataaaattggggacctgaaatactatgagggggatttacaagctgcacagtcctattattttcaggctttagatgttcgccgcagtgcaatcaagaatcattcaactgttccttctcaagtcattgatgcagccacatcccttgctaaagttgctgacgtagatagaaatctgggaaatgaggatacagcaattgctggtttccaagaggccattaaactgttggaatcattaacaataagtg

>comp16407_c0_seq1 sodium-dependent phosphate transport protein chloroplastic-like

gagccatgttgattcccccgcaatggcagttttgtgtatggcatgcagccagggaactgatgctttttcacaatcagggctatattcaaaccatcaagatattgctcctcggtattcaggtgtattgcttggtctatctaatactgctggagtgctggctggtgtttttggtactgctgcaactggttatatcttgcaacatggttcttgggatgatgtcttca

>comp16492_c0_seq1 u-box domain-containing protein 52-like

aggccatctttaatgaactgaaattacatcccttttttcattctcataagatttgttatatgtttccatgcctttctcgagaccccagaaaaccgaaacttttaaaaacttctgcatataaagtctaaattttgatcatgtgaatttgcctgagaagcacacacattcaaagaagtgcctttcagaatgtcttgtgacttgtcgcctttgatttgatgcagtcttggatttggatagagccatttcctccttattctgtgaatgctgatccttcatccttttgccatcatataccacagtaacttcacaatattctggtgcatatttctgcacatattctccttttcctagcccatttcttgatagccttgagaacggtgattgcttagttcccatgacaagattggtgatattagcaacaggaatcagttctagaatggccttggctgatgcattactctccactagcatggtgtccaccaccaccttcgactcattacacagtcgaatgtacttttccagtagatgttttctcttgttgctttcctcccttatataaacctgcacctgatctttgctcagctggctt

>comp16492_c1_seq1 u-box domain-containing protein 52-like

gcttcttgatagcctccccactggtgtagggatgtaggtgatcggagggaagacgtgcacgagaaaaacacgacttccaggcgagacagcatgatcaagtgcccatttgaccacatgtaaatcatttttaccaacagcaacataaacatcactagctcctccatctctgctgcctaccatgctcttgctttcctctccaatctctactatttctggcgacgtcgctggccaattacggtattgcccctcttctatagccattgaattctccccctccatctatccctttgatcctcctttttgctttagacaagagattcatacacaaaaagaaaagaacaaatttgatagaggatgtaaaaggggtcgt

>comp16507_c2_seq1 stromal 70 kda heat shock-related chloroplastic-like

accagcctgaactgcagctccaagtgcgacaacctcatctggattaacagtgacattaggttcttttccagtcatcttcctgacaagatcttggacagcaggaattcgagttgacccaccaacgagaattacttcgtctatgtccttgatagaaagcttcgcatctcttaaggcagtttcaacaggagttttgagcctgtctagtaagtcagagcataagtcttcgaatttagccctcgttaatgttgtatcaatatgttttggaccatctgcggtagcagtgataaagggtagacttatattagtttgagttaaagaagacaattcaatctttgccttttcagcagtttcagtaagacgctggagagcctgcttgtctttgaggagatcaataccttcttctttcctgaagttatcggcaagccaatcaacaattctcttatcaaaatcatcaccccccagatgtgtgtcccctgaggtggaaagcacctcgaaaactccatcaccaacctcaagaactgaa

>comp16526_c1_seq1 plastid-lipid-associated chloroplastic-like

tgtggaggaaaagcagtcggaaatcgatgtattgaagaagcaactggtggactctttttatggaacgaacaggggattgagcgcgtccagtgaaaccagggcggagattgtggagctcatcacgcagctcgaagccaaaaacccgactcctgctccgacagaggctttgactctactcaatgggaaatggattcttgcgtatacaacatttgcgggtttgtttcctttgttatcaaggggtgcagctctgccactggtgaaacttgaagagatatcacagacaattgactcggagaatttcaccgtccagaattccgtcctgttttctggaccactagcaacaacttcaataacgaccaatgccaaatttgaagttcgaagtcccaagcgtgtgcagattaagtttgaagaaggcgtcattggaacacctcagttgacagactccattgaattgccagaaaatatcgagtttctgggacaaaagattgatctctcaccatttaaaggtttgctcacctctgtacaggacacagcctcctctgttgccaagaccatttctagccgaccaccactgaagttctccatctccaatagaaatgctgagtcatggctgctcacaacatacctcgatgatgagcttcgcatttcacgaggagatgcaggcagtatatttgtgcttatcaaggaaggctgccctcttttgacaccttagactccactattttcaaaaactgccattttatcactcttttcgttttttgggtgttagtaagattgcatgtaattaatgagtgattgggaagaaattgaattagaaaccaagatagtgatcccggtatatgtatagtgtcatttatctgatgtatttaaaaatatccacattcatagcaggctgataaatagtttgtcgt

>comp16572_c0_seq1 aux iaa family protein

cagggaatgttggatttcatgaacgagagtaagcttatggaccttctgaatagctccgattatgtgccaacttacgaagacaaggatggagactggatgcttgtcggcgacgtgccgtggcagatgtttgttgaatcgtgcaagcgcctgcgcataatgaaaggaactgaggcgaagggactcgcacctagagccatggagaaatgcaagag

>comp16590_c1_seq1 adenine nucleotide alpha hydrolases-like protein

ccaccaccaccaccaccaccaccgttgaaatgcagcagcagcagccatctcctgactctgacctgccaccactcgcagcaatcaaggttcgctcctcctcgccgcgcttccctcccccaacgaacccactctccaccgaaactcccaccgcgaacgcacagcgcaagatcggcatcgccgtcgatctcagtgatgaatccgctttcgccgtcaagtggtccgtccatcactacctccgcccgggtgatgctgttgttctggtccacgtccgccccacatccatcctctacggtgctgactggggttccgccgacctttccatagtcgaccccaaaaacgaggagtctcagcaaaagcttgaagacgatttcgatacctttaccactactaaggcgtccgatctggcccagccgcttgtgga

>comp16635_c0_seq2 dnaj homolog subfamily c member 8-like

cttcacaattattgagtggatataatagcctcctttcacaaatgccatatatgctcaatttaatttataatcagaccttctcgttgtaaagatgctatgaaatctcatgcaaccatgacaagcaatagtgattgcacaagccattccaattcaaggtgctcggccatgttcagagatcattataaaatgtaagcactttcactgcatatgatttaagtgaaatgaccagctctaattggctcataacagagacaatacaattctagcactacaccttcaaagctttctcaaataataccacctggtcaatttacagaaagcagaagtcacgcccgtttgaccggcctttggacgtaggatttatttggatcctcagtcttgagcttaggaggtcgaagctctcctttcttaactttctttcctcccttcataaagtccctccagcttgaaaccctttgttctcttgtgccttcccactgctcctcatgttcacgttttcttttccacatctcttttgtttcttcttcatctttctttaacctaccctcctcctctgatatcctcatctgcatctttcttctcctccattcttgttctgttaagagttctcgaaccttcaattttaactgctgctggaattcctctgatcgttcataatcctgctcgtattttccctcatccactaatgacttcaacttagtagcagtgtcctttttcagctgcttcttcctctttgctctaagttcctcttttgcagcattaacttggttcagaagataatctctctcttgcgggtccagtaatatttgctgagcttttgctaatgctccaaatgcctccttagcttgtggatgtttgcatttgtcagggtgaacaagcaaagacaacttacgatattgccttttcacatcatccacagaggaatcaaatggtaggttaagatattcaaaagcatttaacttgaagcatgaaaggatcctgttgacttcgttatcgcgctcaacctcgctgacttcggcgaagaattgcttgagcagctgatcttcgtcggcggttgccgtggcggaggaggcggttcctctgatgtcacccatcgggtttctg

>comp16672_c0_seq1 glutathione s-transferase u17

ctagttatataccaggacttcttatctgagaaagtagaaaattttgtcattttttctgcgttttatatcagtttgtgtacatcatattttgaagtggaaaaatggcttccagtcaagtgaaagtcttgggtgcatggccaagcccgtatgtgatacgggccagaattgcactgaacatcaagtgtgtgaattatgagtttcttgaggaaaattttggttccaaaagtgatcttcttctcaaatccaatcctgtctacaaaaaaattccagttctcatccatggtgacaagcccatctctgaatcactcatcattgtcgagtatatcgatgaagtttgggcttcaggacccgcaatcctcccttctgatccctacgatcgggccattgcacgattttggggctcttatattgataagtggtatgcatctgtaagggcaatactaatggctgatggaggggaagctaaaaaggcagtggaggaagcacatcaggggctggctttattag

>comp16707_c0_seq1 dead-box atp-dependent rna helicase 31-like

aatcgttctttaagcaggggaaatgaggggaggagagagaagagacagagcgagagaggtttggaaatgcagtatagaagtggtgggggtagaggtcgtgactatggtggcttagcaacaaagagagaagggaaagggatggaaagaagagggtttgttgttacagatgatgaggatgtggaggaggaggaggaagataagggatatatgagttttaaagaattaattgacagtgatgaagcagatgacgatgatgaggaggaaggggaagaggaagaggaagaggtaagagctgaatttgagaatgaaagattttcttctcaatttcctcaaagcttaccccaaaaaagtgattcataccttagtgagagcaggtttgatcaatgtcccatctcaccgttgtcgcttaagggaatcaaagatgctggatatgaaaagatgactttggtgcaggaggcaacactgcctgtcattctaaaaggtaaggatgtgctggccaaggcaaaaactggta

>comp16774_c0_seq1 glycine-rich family protein

tttgagtgatccctcacctcatttctaacggcggtgaaggtctcggagtttggccgcttcggaaacagatgaactctaaaagttgcttaattctgcagttctgcctttagagtgcagcaaggggattatgaagtaagccatttgttgagatgagttgcattcacatcactgcctgccagcttgtttcttcaccttctgcattccaagtgcagtcttttcaacttagtaaggcatctatatgcccatccatttacctgggaacaaaatttcctaagatatctccgaagttggcctctgccacatctcgacaccagcaagctgcagttgtctgtttgtttggtggtaaaggaaagtcagataatgggaatgagggttctccatggaaagctcttgagaatgccatgggtaattttaaaaaggatcaatcaattgagaatgtattgaggcagcagattcagaaacaagaatactatgatgatggaggtacaggtg

>comp16774_c0_seq2 glycine-rich family protein

tttgagtgatccctcacctcatttctaacggcggtgaaggtctcggagtttggccgcttcggaaacagttctgcctttagagtgcagcaaggggattatgaagtaagccatttgttgagatgagttgcattcacatcactgcctgccagcttgtttcttcaccttctgcattccaagtgcagtcttttcaacttagtaaggcatctatatgcccatccatttacctgggaacaaaatttcctaagatatctccgaagttggcctctgccacatctcgacaccagcaagctgcagttgtctgtttgtttggtggtaaaggaaagtcagataatgggaatgagggttctccatggaaagctcttgagaatgccatgggtaattttaaaaaggatcaatcaattgagaatgtattgaggcagcagattcagaaacaagaatactatgatgatggaggtacaggtg

>comp16836_c0_seq1 acidic endochitinase

taaaattacagttaacttctctcaagaaaaaaaaaaaaattgcagttaactatgattgccaaggaaaaattaaaatcttttatttgtaaatcgtgtaaatgaaatttcccaaattcacacacttttaacaatgacggaactataaccactttgatcatcccaatacttcgaccatagcatcactccaccatacttcctcgacctctgaattgctggcagaatctccctcgtcagcacatccgctggaatgaatccactccctgcggcctgcggcgccgctggcagaccgagaaatatctttccagcattaacggaactcgtccagcgtatccatgaattcttcaaattattcgtgtttcctgaagtgtactggcatggtggattattgtaaaattgaacccaaacatagtcgaaaaggctcgtatttagggccgccgccaataatcggtcggggaacgggcattgcggtgcgccggtaatgtacacttttctaccatttttgctgaatcgtttcaggtatttcacgagatcgtcgtagtagagcgacgatccgagctcgatatcgaagtcgatgccgtccaatacagcatctccgagcggccgggtggtggagccactgccgccgaggaaattgttccataaataaatggataaagcttgggcatcttctctggaggagagagagtagtttccaacgccgccgccaatagagagcatgactttgatgccgcggctctggcaggcgcggattccattgctgacaacgcggcaggagttggtggctggattacagtggccggcgaggttgagttcgggggtttggccaccgccgaatttgtagaggaaagcgatgttaatgtaggaaaatctgcctgtggcacaggtttcggcgagagtgccctcgttgccattttggccccaatagactg

>comp16883_c0_seq1 cold-regulated 413 inner membrane protein chloroplastic-like

tagtgcctctgtccagaatacagcatatctctctttattgaactcagctgagccaaaaagaagaaaagagaaaactaacgaaaaagagaaaaccacttgttacagaaataataacagttcctcaacaagacagctacataccacctatgggcaaacaacacaatgaagaatgtaaggaagcaaaatttcttcagtaaagacttcagacttattgacagaattacaagactctgacttaattctgttagtaacaaagtatcttcaccatcacaagagaaaagggcagccgtctagaaatttattgtcatgatacccacataggagaccaacactttacagaaaatgatttagacatgtcatctatacaactcaaagttagatcagaagcaagcacggcacaacaacaatacagatggtagctaaggtggcaatgattgaaccttgatcaaaagattttcccaagctgcctgctcttgtaaaatgctggaaagccagataagccgctattaccaacgaaagaatagcaccttcttgtgttcctctcagcctcataatttggtagggagccacaatcaccaaaagcaatgttataaatggcagctccagctctccaggaataaagaagaagagacgcacgagaagcgccaagaaagcagtccatatgccatactcacccttcatccatgagacaatgcttgcaggtgcttgtagcgcaaataaaggaacaagaaaggatttgtggatagctgtcccctttgccaacatcagaactgcagtcgaaacagcagaaatccactgaagattcccaggtgtgagcggcgcggaatagcaaacagcaccagacccacgacgcctcttgctcatctccaatagccgaagtgggtgataactgagaccgcaaaaccgtcgctgtatccgaagtcgttgaagctgaggagaaacccgacaaaaggccctgctttgataagagttgggactcctgattgagaacactttgttgttaatggacagagatgagcaagagagagaaaggctcaacattttcttgcagagaagttagctttgtagccggcgaattggtgaaagacgagatgacaaatttctgggtcagttcggagagcagtgaggggtatgggagatttacaggtactctgccacgcgttcgatggtggttcgtctttcgagtttggctgccaatcaacgcttcgatcatcgcacattcacccagaaatcgtctaaa

>comp16974_c0_seq1 10 kda chaperonin-like

ggtaaagaaaagctgtatcagaatgctttaaatcacatctgattaaacagccacaaacagccagatgaatgtggtgttccaatttggaaaatctagtcaatgatcatctcatacagcgaaattttccatcaaaaaaatccactgattgccaaccaactatcaagttgtcaggctacgaaagcatagttgaaagataaataacttaaactagcaatggttcttcactaatgtcctttggcatggtgagagtaacgacaaattagtcgtgcaatgttcccagaatgtcatcatcacggtaaagatggtactctttttcaccaagttttacttcagttcccccatattcaggcagcaaaacagtatctccttccttgacactcacaggtataagctttccttccctgtcatggcagccaggaccagcagcaacaacttttccagagttcaacttgctggttttctcagggagaagaattccggcggtggtttttgacggcggagtgattttttcaaccaaaacgcggttcagaagtgggatcaagcgcttcgccatttgtttccctgaagaacaagaaagcagaacgaaaactgttgagagaagtactagggttttggggtttggtaagaaattggggaagggtttaagaagagggggagcctttca

>comp17001_c0_seq1 protein suppressor of gene silencing 3-like

aagttccaagtcagtttctagtttggctgctgccctactgccctactttgtttcagctggagtgtattcctccaacagcctgttgaactcctcatcaaatttcttctcaagttgcatctcctcatccaaatatcggcgcttcagatccaacattcgctcttcatgggccttcatcagctcatccctcttcttagcaaactcctccatttctttgtcctgcagctgaatgaagttgacaacttcttcagccctgcgttgacgctcctccacagaagtcacgtctgccactgattgggttgctttttcacgttcatattgctggattttctcaaagttctcctcctttgcatttctctcatcatagagatgttggagttgctctctatagaactggtcttgactatccatctcctctttgttttgttcatgatgttttttggtcctcagcttcaccacacgattctctaccaatgtcttccgcagcttttcagtaagtataccaacagattcttcaagtgctttcttactcgtctgctctttaataactttgttcttgaaccatatcagcttttgattgtcctcactcatctgcttcatttgatttacgaccatttcttgatacgaccgcatctcatatttcagtttggatttgccttgagaatgatggttgaaggtatccaaatcttgcttctctgccatgtatccata

>comp17034_c1_seq1 bes1 bzr1 homolog protein 4-like

tttacgctctacttaaatgatcagttgtggcctcaagttttaatgtattgttttaatcttccggggaatagctatggtgatttacctggtttttgagcttccaagagtaagctcgagttcatctgagccacactcctcatggatcctctccccttcccaaggctttacaagacctgttatgttgcttccaaatgcaaactcatttgagatcgcttcagccattggaatatcagaagccgccgcaacagcaggagaacacgtcccactttgcccaggagtgcacatgagagatccacctcggctcaaatcttctactctgaagccaaatggatttgaagaaacaaggctaaatgttggagaagttggaccactttgagggacttgaactccagcataccattttgaatctggtgggacctggcggccagggcttggaggcgtggacaatggaaggaaggggtgttgaggccctccccacccagggcgagcaaaagagttctcccagtcagtcttaagtcgcggggttcgagcagttggagagctcaagggaggggttacaggagcacttatggagccaccatgcatataatgatgtatgaactttgatgatgatgctgacgaagatgatgacgagaggtttttgagccatggaatgagggaattgccatctgcaataatgctgggagcataagtggaagaaactggacttgggaaggaagaggaggctgggcttgggttatatgaagtgtgcggacttggctggtaagatgagcacggactaggtgatgcagatccacctatgatatccatattttccactggtcggcaacccttgcgataggtggtgccgtcgggctcaaccatccaaccagcctcgttgcagagagccttgagcacctcattgttgtcgcagtgcttcgggagcttgtagttgccgtagaggcggaggccggcgaatatcttggctgcgatggccctccgccgccgctcccgacgtttg

>comp17092_c1_seq1 calmodulin-binding heat-shock

gttttcgcggggcattctttggggtctggtgttgcagctttgttgagtgtgattgtggtgaatcatgggaatgaactgggggggattcagaggagtttggtgaggtgttatgcggttgcacccgcacgatgtatgtcgcttaacttagcggttaagtatgctgatgttatatcctctgttatattgcaggacgatttcttgcctag

>comp17248_c1_seq1 tetratricopeptide repeat protein 4 homolog

atgatcttgcttcatccaacaggttgagtgataatgatgctttgaccgccctgtaaagagccttaacatttgtggggcacagtttaatggcctcttcagcatcctggagagctcgtctgtaatttcccaagagcagattgacatgcgccctattggaataaacaatagagttctctgaatcacttaaggccttctgattaatagccctagtgtagcaatcaatagcatctgagtaatgctccttcccctttttcacatactcatttcccttttccttaagttcaatagcagcagattctttgagcgcagcgatagcctcaagatcaacaatttcttctttggttttgggttcagaaccagcctccatccaa

>comp17278_c1_seq1 lrr receptor-like serine threonine-protein kinase rpk2-like

gggattgatccacggagtagattataagacaagtaaagccgcctcaaatctctaaacccaccaataaaccgtggaattgttccattaacctgattcccagctagattaacaatttgcaactcgacacaattcgacaatgaattcggtatacccccaaaaatctgattgaatcccaggttaagaaccttcaaattcctcaaccca

>comp17428_c1_seq1 separase-like isoform x2

aaactggtgacgctccaatgagaaagccaagagtgcaagcatctcgagcttgccccataaaggaaccaatccttggtctatgattacatattctggacttacatttgtttacaggtacattacacagagcacactcagctgatctctctcttaaccatgcattgagcattgccttcccaaacctgtcaatgtctttgtcggtcacttcccataaattagccactataacaggagagcccgccaatatataagagaggggagccccttgaggcatatagcatcctctcaaataaagagaaccactactacatcccagaagtagagtagctgcacaaccatccaatttctgaatctcgtgcccggggatatattgcgtaccacttccatgaccaaagtagatgaagaggtcatggtttttcaaggctaggctgagttcctcaactgtgggca

>comp17559_c2_seq1 desiccation-related protein pcc13-62-like

gggaataacataggatgcaataagatagttaatatcagtggcatagggatcaaacggaggctctagagcttttccgaatgcattattcataacagtggcaaacgatgctgcacttagattcagaagtggtcttggaaatccgggaacagtcttctcaattgccctcaaatgtccaaactcttggaatgcaaactgtgtgatgatatctcttatcagtgggctgagattgg

>comp17599_c0_seq1 negative regulator of systemic acquired isoform 3

gcttcccctttctcattgtgaccatccaatccaaaattaaaaggtaaccaagcctcttcgaccacaataatttgggagagaatttctaaaggagaagatgaatctgaacgttctcctttagataggtacacgcgaggaaagcgcttatctaattcaacgagaagctgataactttcaattatcagatctattgaatctgcatccttcagaatatgaaaaat

>comp17599_c1_seq1 negative regulator of systemic acquired isoform 3

aagaccttgctgatctgctatccgccgtgaagaatctagctccatgatcattacgaaaagttccttcaggatggtgcaagtagacttttttacttcaggtaaagctaatgctatagcactattgacttcatgcagtagccccccagaagatttttcattgtagctagtttcttcagaaaataaaggagaatgcgccatattgcacatgatggacaaacagtccttcattaatcctttgtacagtatttttcttgatcccagaagcatgtttagcaatgactccctcaaaaaggtccagttcatattttcttgataagcgcaattacgaggcaaaaaatccgcttcaagaacagtgatgagatattgaagcagtaacatgttcttgaagaacattatttctgctgcttcagacttcttttccttattcacattagctatttcttgtataagagcatggaatcccaaggaatccaccgaatctcttgaatgatttgtagcttcccctttctcattgtga

>comp17723_c0_seq1 heat shock factor protein hsf30-like

ggttccctgatgtggaaagtgcaagatactcatcagacacacttaaaatgctacgctgctgaatttaatattcacagcatcataacctataaaactaaactggaaagtcttgtggaggccgaagaaactacattatctactgccggttagtatctttgtggcgtcaactacagaaattccaattgatcaacaagatcttgtaaatcctcaccccaatatgatgtttttgcaaccaaatcttctacttccacatccttggtctgatcagcagtgaggttttctactgcctcatcatcagctatgaggtcttcaccaagcaacttctccaatatatcatcagtaatgggattcaagctggcgtcactagtggagcgaacggcatctgacaaaagatctttcacatcgccacttgattcatcatccagtgcagctgaaattagtgtctccatctccattccagcatctacaagctcctcttggctttggctggtgtaattcgaatagtgatcactgttgatgtcaacagttaaaacattctccaagattggactcatagtcagtcttctcttttgtccgatttcaacacgactttgatcattcttcgccgcatgtttatccatatatcgctgcatgaacaaaggattgacaaaagctttagcaatgaaactcagaatctgttgttgctttctctctgaagcacgtatcgtttcttcgatgaacatgactcgctccctcgagttgtgctgctgttgtctaagcttcactatttcagccattaacagactccggtctcttttcaatctttcgagctcttcttccattccatagtgccctaactccacacaggggcctccaccttgctgactcatgctctgcattacatttctcttccttttgatggtcttcaagagatgcttctgtcctcccaaaaac

>comp17742_c1_seq1 nf-x1-type zinc finger protein nfxl1-like

agccatttgtcaccctgccactccatgtcctgatggaagatgtgagttccctgtcacaatcacttgttcttgtggccgaataagtgcaacagttccttgtgatgctggaggtagcagtggtagctataatgccgataccgttcttgaagcatctataatccaaaagttgccagtatctctacaacttgctgaagaaaatggccagaaaattccacttggtcagaggaagatcatgtgtgatgatgaatgtgcaaaagtggagcgcaaaaaggttcttgctgatgcttttggcgtaattcccccgagcttggatgcacttcactttggtgagactgcatccgtatctgaggtgctttcagatcttctcaggcgtgaccctaagtgggtcttgtctgtggaagagagatgcaggtatcttgtccttggtaggggaagaggggggataaatgctgttaaagtccatgtattctgtgtgatgaccaaggaaaagagagatgcagtgaggttgatagcagataggtggaagctttctgtgaatgcagctggatgggagccaaagagatttgttattgttcatgttacacccaagtcaaaggccccagctcgtatgcttggtgttaaatcttgcaatcctagtaacatgttgcagccaccaatttttgatccacttgtagacatggacccccgacttgttgttgctttatttgatttacctagggatgcagatgttagtgcattagtcctgaggtttggtggggaatgcgaactagtctggttgaatgataagaacgcgctggccgtcttcagtgatccaggccgagcagcaactgctatgaggagattggatcaggggtcggtgtattatggtgctgttgctattcctcaaaatggtggtgcttcagcagtcagttcaggtgccagtgcatggggatctgctgcagcgtcaaaagatactgcttcaggaacagcattaaaggcaaacccatggaagaaggtcgtcttgcaggactctgattataaggatagttcgtggggtgctgaggagtggtctgctaatgctgctgattccaaattacatcatcggaaagagaaggaagttccaattgctgcttcaagcaacaggtggagtgtcctgcaatcaggaagcacctcgaaatcgtccgatgcttctgcaaaaattgagaaccttcaaagacagacagaaaatccttccatgtcaggttctaatgtggaagaatccagtttgaatttgccattgcagcaggaaggtgctcacaccgacatgtcaggtgatgtagttgacgactgggagaatgcctatgattgagaaagtatgcatacaaactttatctctcaaataatgcattcaggtgctatatgagagaggagatatcttttagttgcttttcctatttacattttatattctcccatcaaattccttatagttggttgcgtttccattacacaaccatatttccagcaggctatattgctctgtagaagtgttggcagagaattttgcggctgcctcgtgattttctttaaaaatatggatgaaggaaagcatttttagcagttccctcatttcttcttgtatccaagtatgctgtttatgtgattcttcggacgagcttggttt

>comp18107_c0_seq1 chaperonin-like protein

ttcaggccctggaaaaccagaactgctactcactccaaaactctccaatgtcacaaaatgtatgtgcctgggtttggagaagcctccccagaagccaaagcagcaaatcatctgcacgactttttcacctacattgcagttaaaattgtcaatgcgcagcttcagagctacaaccgagaggcgtatgcagatttgatgcgattcctggaaagtcaccccttgaatgatggggataagttttgtgccgatttgatgcgagaatcatcacgccacaaaagtttagctctgcgcatattagaggttcgatccgcatactgccagagtgattttgaatgggacaacttgaaacgattgtcatcaaagatggtggaggatcgtaatacaagactgatgagggattatgtgtcggaaactagcgcattgaagaccgagagtgagaagtaatctctcctcagcctcaggcaaagatacataatatactttatcattagtgtgtctgtgtgtgtgcgcgtatacattggaaattagacagcagcgtagttcgaaaggtagtagttttattcaaattatgtaatgcaaacgtgatggtttaaaaatatgagcaaagaaatagacaccat

>comp18107_c0_seq2 chaperonin-like protein

aggactattacgttttactatgattatgtaaatacgggcataacatggctgatgtttttattttgcttgtaatctgagccactcctgccgtgcagctctgcgcatattagaggttcgatccgcatactgccagagtgattttgaatgggacaacttgaaacgattgtcatcaaagatggtggaggatcgtaatacaagactgatgagggattatgtgtcggaaactagcgcattgaagaccgagagtgagaagtaatctctcctcagcctcaggcaaagatacataatatactttatcattagtgtgtctgtgtgtgtgcgcgtatacattggaaattagacagcagcgtagttcgaaaggtagtagttttattcaaattatgtaatgcaaacgtgatggtttaaaaatatgagcaaagaaatagacaccat

>comp18383_c0_seq1 pto kinase interactor 1

ggtagtcgccattcagtctagcatcaaccaactgctttatcttgtttttgctcagatatggcatcgcccatgtcacaagagactgctgtccgcttggccgtgtatgatcgaatgctttatggccggtcgagagttccaggagtgccataccaaagctgtaaacgcgcaactccagtttagaacgccataccatgtgtattctgggggatgataaccatgactccacggaatggtaaactcat

>comp18418_c0_seq1 extra-large guanine nucleotide-binding protein 1-like

gtatcacctgcattgggcatcggattgatgagtcaaagcgaggatcacttgggaagtgttctagaatgctgaagaaactgcttgccgatgatgtagttaaacaaataatgagatctgaactctcatgtgaagcaaatcagctcccatcacgtctaatatgtgtaaacggcaagcctttgcctattcaagagttggttatgttgcaaagctgtccatatcctccaaagaagcttagacctggaaagtactggtatgataaggtttctggattctggggcaaggtgaggagtttcttcctcttagattcacttctgaaacttgtaacatgtattct

>comp19077_c0_seq1 dual specificity protein kinase spla-like

tcaactcaagtgcagaccgtttaaaggtcggagattttgggttattaaccaggttcattaggatgcggcattctcatgatgtttacaacatgattggaagctaccgttatatggcacctgaagtcttcaagcaccggaaatatgataagaaggtcgatgttttctcttttgcaatgatattatatgagatgcttgaaggtgatccaccgatgtcaaattatgaaccatatgaagctgccagatatgtggctgaaggaca

>comp19077_c1_seq1 dual specificity protein kinase spla-like

tcaaaaatcaaataaatcaaggaggttggaccataaaacacaaaatactaatgggactacagaaagtgaggaacctaacaacataaacactttcgagatttagatcccagtgtcacaacagctaccagtctagcaaacctctactaatggagtctattcacaatccatctcaatcccacatgttttggtgtgaaaggagaagttaagtaacttaagtcgaaggatctttataccttatccagtcaggttttggattatgatgcaaatatgtgccagtgatgatcagatggtagcatttccttgattttttcgagtctccttaaaatctccaagaaagaaggtctctgattcatgtccggtgcccaacactgctcagttaactctcttaattcaggaatgaaaccttttgctctgatcattggtctgtgtccttcagccacatatctg

>comp19101_c1_seq1 chaperone protein dnaj 10-like

aaaatagcttcagctgatgaagatgacagcagttctgatagtagtgatgacgaggactccctcagaccactgaattacagaacccctttagttacacagggcattggcagactcttcaggtgtctttgtaacccagccttcgacgtagatgatgatgagattgtgtatagaggaagatgagtacatgaactatttatacaacactcatttgtggaccggtgatcaccccttttgtttgtaca

>comp19101_c3_seq1 chaperone protein dnaj 10-like

gctgaagctattttcttttttgatgttccagcattttgcatgtacttctgactctgaaaaacttttcctaggattttcagagctaatgcacgcatcttgagttcttccttcctaacattgctctcctgtagcaccatttgacacacatgtactagagttatttcaatgtccaccacattcagtttccatagtgagttcataagcgtgtcttgatttagtcttagatgtgattccacatcattttctggaccattgccgtccatcttgcattggcgtcgaatgtcatcctgaagttggagcaactgaaaagcaccttttgctgctgtaaattgtgacttccaaaagtgtcccttattgcgaacccactcagccaaaaatggcactcctagataaatagcttttt

>comp19238_c1_seq1 rna pseudourine synthase 1-like

attctccccaaattcagccggaaaacgccccccaaacaatcgataattacccagtgccactctcaccgccgccgccgctcatatcgaagaatgtagagttgaatagagctttaacagcttcttcgaagtcttccctgttttctctctctagaaaagatgtgttattcgaagatgagtggctcatcgctgtaaataagcctcagggaatctattgcgaaagcgttttatcttcggttcacagttttcttattgccgatcaaggaaagcaagtaaaattgcaagaactccatctgtctaataggcttgatcgtgacacaagtggtgttactgtcataaccaagtcacacaaagtggcagcgaagcttgtgaaagcatttaccgatcacatggtcaagaagacttaccttgcattgtgtattggcacggttccagaatggcaaaatattatcataaaatctggtcatggtcgatcaaggcatggggcatggcgagtctatgctttttcagatgttggtcggacactacctggtggatcgtttgttcgagatatggagacattttttgaagttctgtcagtgaatgggaaaagttgtgtccaagagttctctggactggaaaaacatggagagaaaatcatgattgttgaggaaaaatctgagattatctcttgtattgagaaagccgacattctagtaagagcgtatcctagaagtggaagaacacaccaaattcgtttgcattgccagtatcttggaattccta

>comp19263_c3_seq1 protein nlp6-like

gaaacaaagaacagctaacatcatgccagccagtgatttttgaggttggggctaagagatgtggattggaaaatgctaagataaaggaaaatgctgctagttcatcatctatgtttgaatgttctcaaaatgcagaggtggtgcagtcacatcatactatcacacaaaaaaatgatgctacagaaacattaatcttgggtgagccagttagcactaaggttcttgaaaaacaatcagggaagagacgaacaaattttccagatgatcccagtgttaacaaatctacatcaaagaatttgagccctgtacctaacaaaaacagatgtgcaatgaggaagagaagtaaagttgactgtccttttactgagcataaatccatgaaagaggcaaagaaacatgtggtagaatctggtcagcaacctgagtcatgctatgtgagttctctagaggaagctcgctctactttttcttctctttctcctgtatctacacctatgcaaaaccaaggaagaatcatgactgtgaaggcagaatatgaaggtgatattataaagtttcaggttcctgttgcctctggaatcatgattttgaaagatgaagtgatgaagaggctaagattgacagtgggaacttttgaaatcaagtgtcatcgtgaagataacatttggatcgtacttgatagtgatgcagaattgcaggagtatgtgagtgctatcagatcattgggtataagcacaatcaagctttttattgtgtcaattgcgtcacgaactgaggtaaaaaaaaacgttgaaatgactgtaaaagcatcatatcaagatgatcttataaagtttcagctttctctttcagcgggaatattggaactaaagaacaaaatcatatataggttgaaattggatgatgaaagttttgaaatcaagtatgttgatgaaaacaattgtcagattttactagacactgatgcagcactgcagaattgcatcagtaaagtgacatcattgcagaaaactacaataaaactctccattcagccaactagatcacaaccagcaatgagtaatgaatgcataattatggtgaaggcctcttatggagatgatatcataaagtttcacctatctccttcttgcggaatgatgaacttgaaggatgaagtgacaaagaggttaaagttagaggctggaagttttgtgatcaagtatctggatggacataacaatgaagttttactggacggcaatgcagcactggccaagtatgtgaggacaatgacatcaatgggcatatacacaacaaaaatctctattcatcctcaagaacaatagatttaacctcatacaagatgcacatatggaatgataacagggaacatgaacttatgtgttcatttaggtccataaacactcaaatgaatttggagcagacaattagagtgttttgaagggtggtatgcatgactacaagcaaccaactaaaagatgcccttgtttctggatgtcttatctttctatttcaaatttgttgcttcaacttttgtacatagagtcgaggtaattatggaattgcaatggaggtatttcaaaccgacaaattctttactgtttgatttgacagtatgcaatgtgctgttttcctt

>comp19442_c0_seq1 heat shock protein 70 -interacting

aggacaaaccgagtccgcttgtatcagtgactggattattaaatttgctcagttattcaagaaccatgttggatttgatattgatgcttatcttgatctccatgaggtggggatgaagctctattcagatgctatggaggagacagtcactagcgaagaggctcaagaccttttctgcagtgctgcagagaaattccaggagatggcagctttagctctgttcaattggggcaatgttcacatgtccagggcaagaaagagggtatattttacagaagactcttcaagagaatctgtgcttacacaggtcaagtctgcacatgactgg

>comp19442_c1_seq1 octicosapeptide phox bem1p domain-containing protein tetratricopeptide repeat-containing protein

ttctcctcagccctgttataaagctcaagaacttctgttgaaggccatgattccaaatcaacatcagttccaactgcataataccaagagagttttgccagctcaaattgttgctgtccaagagctaggacagcttcgtagaaattaggtttaatttttaaagcctcctcatatcttttccctgcattcacaaactccatttgtgcccag

>comp19515_c0_seq1 nedd8-activating enzyme e1 regulatory subunit-like

gttttcaatctggagatctcctcatccatctcaccatcaaactgccctgggaaactattatagttcgcagcaaaacgatctacggctcgtagaagaatataaaatccaacggaaatgcagtagtcttcatctgttagatacttttgtaactccaactgagccggcgaatgatattcatcctcaatcgggcgatatctgcataccttgagtttcctagcatttttgcaaaagctttttatggctgcttttgagatgctatctggatctctacctatcttcttcaatatttgcctgacatgcttctccatgacaagaaagtcagcctcagccttagcctgatagattttttgcaggctgacatacaactcggtagaagatgtcat

>comp19608_c0_seq1 chaperone -domain superfamily protein

aagaagataacatatattatacaacatatagagaacaaaatgaccacaatatcaaaggtgagagcttgctttgcttgatcattgagctccatgttcctcccagccagtcgatccaccatgtctggaaccgattcccatttttctacatcttgcatgtatgggtcttccagcttcatcctcatcttctccgcttctctgcttgctgcttcctgagctagcgtccaatcataaaacttacgcttctcttcattgcttaggacatcataaatttctcttaatttcatgaacttgtccgatgctgctcttagaggaagtgcagttgtgtctggatgatattcctttgacaatctccggtaggcagctttaatttcttccagatcagcatttgcagatactcctaagaactggtaatatgaatcagatgaactgttgagcaagtctgagaatttttcgcctagccaatcgtcttcctctttaacctcctcctgtgttgagctagttccccctatccatccctcatcctcattctcccaatgaattcttgtatcgactccgggaggtgccctctgcttccgcccgccacttgaaccttgcacagcca

>comp19723_c2_seq1 phospholipase d p1-like isoform x2

atttcgtgctcatgcatcctatcggcttgattctttgctggaatcgaaagccaagcaaggtgttcaggtatacattcttctatacaaagaggttgccctagctctcaaaattaatagtgtctatagcaagagaaagcttctgggcattcatgagaatatcagagtacttcgatatcctgaccatttttctactggtgtctatctatggtcccaccatgagaaaattgttattgtggatcaccaaatatgttttattggaggacttgatctgtgctttggtcgtta

>comp19730_c0_seq1 potassium channel

aaaacgattaagccagctattgcgactgaaccgcaccacatttttgaacattgttcaggccaatgttggagatgggacaataatcatgaataatcttctacagcacttgaagcaattaaaggacccaatgatggagggagttcttctggagactgagaacatgcttgctcgaggaagaatggacctacctctcactctatgttttgcagcacttagaggagacgacttgctgttgcatcatttgttgaagaggggtctagatccaaatgaatccgataataatggaagaacagctctgcatatagcagcctcgaaaggaaatgagaactgtgtgcttcttttgctggattttggggcagatgccaacagcagagattcagaaggaagtgtaccgctttgggaggccatgtttggcatgcacaaaccagttattaagctattgttggataatggtgctaag

>comp19749_c0_seq1 ribonuclease t2

ttgcactctatgaaaggttcataccctacaccttcttcaattgccgtctttatgctttccatactgtaaaagtttccaggacgaattcctgcatttctaaggacttggagcaagttggatttattcttgaggtcaagggcagcttgaaaataagtgtgctgatcaaaggaggtacatgttccatgtttctcccattcatgaccccagaatttcattccatccccggatgggcaggctagagttggccaatctttttgcattctgctgacaagatctgaaatctctgatctgtctaaagaactctcacgatcacaattttgtggccatttaccagtcttgtaatttggccacagcccatgaatactgaaatcttcatcaggctttccagtagttggatagcagcaacttcgtctcgtgtcacaataagaagcaggccactgttggacaaaatagaagaaatcaaagtcctgggaagagcccaaaatagatagacattgtaaaatgaatagctggatcaatgctgaaccctttcctctcattgtgaaattcaacactggaacaag

>comp19749_c1_seq1 ribonuclease t2

atagagtgcaacgtcgatcgggaagggaatcaccagcttttccaggtttatttgtgcgttgagaaatctgcttccagcttcattcactgcccaattctcccacatggaagagcatgtggttccagcattgagttcccttccttctcttcggattcggaatcgcaagccgaactctgagtttcttgcattgatactgtctcagttttaattttctcaactatgtacatgttgagagtttgatcatacatttggttgttcagattgatttcctttttacttgattataataatgttgcattcttgaggcaa

>comp19779_c0_seq1 at3g20300 mqc12_5

tatccagttcatcatcttcatctccttcattttctgtatcccaattagaagtgactggataccccacctggactgaagcaatctgaggcgtaggagtttcatcattcatttcatcgaatgtactgattgaggcacaggcatgccattttgcagcgagcgatgaaatagcctgagctttgtgcgttatttttgcagcactgcgcaagcagataaaaagccctgttaccagggtgatggagcataacgtaagttccccagctgtgggaatgtcaattttagagcttggttttaacgtaactagcaaggaaacaaattggctcattgtcacaaggatcagagttgacaaaatgaagagtctaaaccggtgacttatgacccgaaggtttcttctgattctcaaatgttggatcagtataaaagcaacatcagatcctcgctcg

>comp19779_c1_seq1 extracellular ligand-gated ion channel

agctctgataaactcttccttaatctcggtgcacaagcgcgtcactgtcctgcctttatcttcaagcatattcatatatctttgattgaatcattcattgatcagtaattatgcaagcactcccataaattttcaaagcagtagaataaactgaagaactcaaaaccccaatggaggcagaggccactgaaaattcatcgtccaaaaacaaatcagtttcttcaggccactcatcccatgaagccaattttggattgaggagttacatttcacctctaaaatgggtatttcttgatcactctagtctgtggagagctgggctttcttggtcgatcttttttctgctaaatattggtgttccacttgcatcccactttttgttttcttgctcaaattgtgatcccaaccatcaaaaaccttatgatgcaattgtgcaattgtctctctcagtatttgcagcactttctttcttcagtctttcttcttttgcacacaaatacggattccggaaatttctgtttcttgatagattgcgtgatgaaagtgagaaggttcgacaaggatatgctcatcaacttaatagatcagtgaagctcctgtccgcttttgttctcccttgtttcctggctgatagtgtctacaaaatatggtggttcagctcagtgggaaaccaaattccatatttgtataacatacatttgaccaaacttgttgcatgcacactgttgatgtgttcatggttgtaccgcacctcaatcagttttcttgcgtgtatcctcttccgccttgcgtgttatctgcaaatcctaagattggaggactatgttctggtcttcgagcgaggatctgatgttg

>comp19938_c2_seq1 transcription factor vip1-like

gaaggcggcggggcaccatttcaggagcctgtcggtggacgcggacctctttgagggacttgtgttggacgggccgccgacggtttcggcggcggcggaggtcggcccgcgtccgaggcacagacatagcaactcgatggatggttattgcagtgcgacgtcgtctgaggtggagtctcatgcgaagaaggctatggctgctgataggttggcggagcttgctttgattgaccccaagagagcaaaaaggatacttgcgaatagacaatctgctgcacgatcgaaagagagaaaaacacgttacacaaatgaactggagaggaaagttcaaactctgcagactgaagcaaccacactgtcggctcagatcacattgttgcagagagacaccagtggactgacagctgagaacaaagaacttaaactgaagttacaggccatggagcagcaagcacaccttagagatgctttgcatgaagcattgaggaacgaactgcagtggctcaagatcacagcaggccaagctcctggtgtcgatggaaacaacagggggtcatcttcccaattttcctcccagccttcagcaatcaactactttggtaatgaccaggcacaacagcagcagccgcagcaacagcatcaccaaccacttcacatgcttcgttccagccccaataaccagagtcccagcccgcagtcccaaccaaacttccagaattttaatcacagggcttgatgacgtctccaagatattgctgttctatgactggcgtttggaaatgggaagtaactatccagctaggctcttgtatataaaatgtatacatgtgttgcaggctgcagcatgacctagtaatgggtgttgct

>comp20027_c1_seq1 zeaxanthin epoxidase

acacgacgtttcctgcctgcattatcagtgatccaagtgccatgctcgctccttaagtcggtcaggaaaaaggctccatctttgcagcttattcgagcgtgcaatttggagacctgcggcgaaggtatagttattgataatccgggaaagtttgcatgtggtacactcccgattaagcatggtttcttctcatctcggcttaagtggattgtttcggatgcagcagtgaaatctccaatggggcatagaaaccaatctgcatcaagag

>comp20056_c2_seq1 probable folate-biopterin transporter 7-like

atttatttgtaaagggggtttatcatagggttggccttccagactctttgtatgttatagtcttctccggcttgcttgaggttctctacttcttcaagattctaccgttcagtgttctaatggctcagctctgtccaaaagggtgtgagggttctatcatggcatttgtcatgtctgctattgcacttgcatttatagtg

>comp20160_c2_seq1 gdt1-like protein chloroplastic-like

tcacctcttggagtcattgcaggggcactagcgggtcatggagtcgcgacattgcttgctgttttggggggctctttcctgggaacatacctctctgagaaggctattgcatacataggcggcactctcttccttgtcttcgctgcagttacattggttgagatcgtgagctagaatctatcaaaattctttctctattggcaacggcgactttctttgaactgcatttggttcaatacgacaaagagatttgaagagcgactgcagacatcttgattttaactgtataacaaccaaagtttagcatgctgataactttcaggggaaatacaacttccattattaagcttcaatctttggtatgtcagttaggttcctatccttcactttctttttggaagaatgcaaattcaaagcatgtttttgaaaacatgagtagattcacggaataacatctttatttg

>comp20173_c0_seq1 phospholipase d p1-like

aaatacttagaaattatacaagggacaaagattcaaatacgtcgaccgcaaaatattccaatgttcaatacatttgagtcttttgatgtaaacaaagaataatctgcctctgaataattagaggggaaaaaggggtgtcattgttatagctgccaaaatacatggatgaggcctttatttattgctgcaactttctgcctgatgatgtgcaaatcatttcctatatgggttaagatacaacaatctaattgtgttgaatggtaagtatcttgttactgaagatatggattatttctattgaaataattgtaccacgacactatcttggtcgatgagttaatcttaatgaaaaacctgaggtgatgcatagtattcgctttcattaaatacgggtctcaaatcttctttgcacataaaatccagaggaaatgaaacaagatgtcccttcacagattttaatctttccatgggatctgtgcctgtaacatctccatcttggtagctttctagcttgtctggggcaattcctaaatcactagtggtatggccgagtttta

>comp20196_c1_seq1 histidine kinase 3

atgtgtgatgagagggccaggatgctgcaagatcagttcaatgtcagcatgaatcatattcaggcaatgtctgtaatggtctcaatctttcaccatgggaagaacccatccgccattgatcagagaactttcgcaaggtttactgaaagaacaggatttgagcgacctctcacaagtggagtggcgtatgctgttagggtgctacatactgagagagagctatttgagattcaacaaggctggactattaagaagatggataacattgaacaaactccagttcataaagatgaatatgatcctgcagat

>comp20209_c2_seq1 heat shock protein binding

ggtgtttggtctgcaagccattccctgacatatagcccactccgacacatcaaagatcctgctttcagcacatacaaaagcacgaggtatttccaccttttgtggccgatcaaacacgagtgaacctttgtgctcaacccatccatcaccatctttggcttgatgatactgacagcaatcctgacaccatcttgctttcgccttggttcgatttgtacaaacccagatgtgtgacttgccacactttgtgcactgtatgcgccttgactcctcagagcaaaagtcagtagcattccgataagaagtgctagctgacttctgcataacacacttagattcttcttttctgagttgctcatcatagtccctcttcttcacagcatcagaaagaacctcatatgcacattgaagtttcttaaatgattcacttgctagtggacttcccatatttttgtctgggtgcacaagcatggcctttttatggtattccttcttcaataacacaacatcaattttcttttgctgtggaaacccaagggcttcatagtgattgccactatttaaaatcctctccatctcaatcatggaattcacatcttctctgaccacactcttaccaacagattcttttggcttttcaagaatagatgaaggagtagcaggtttggtggctgatttacatgacttcagcttctcttcttcttctgtttctacttcagtagggacagaatagtcacacttcgtgcagaaatcatcttctgtgaatgaatcagattctttatgctcctcaaaatgagtgctttcactcaaattatcacaccatttgatcaagtagttcaacacatcattagataggaaagcaagattaattgcgagaaaaactccaagccatcctactcggattttgacgcaatacattgaatatagggttgccataaacaccaccagccgtgcatgatttaatgaaaatagataaccaccgactatgaacaaggtacccgtaatccaaaagttagcatacatccacaaaatcaaaatggcaaacagccccacaataaaaagtccaggagtgtagcctaaatactgaacagcaaccccagctgctcccatactaagaagcacatatagaagacatgacattgatgtcaaactcagaaagcaactccacataatgacaagcagagctgctgatcccagcccaataaaagactgcaacccagaaacaaaacagtttctccagtaaaccaacaagaacagaaggcctctcccaaattttgcgcatccac

>comp20216_c1_seq1 iaa13 - auxin-responsive aux iaa family member

gagattggatgttggttggtgatgttccatggataatgttcgttgaatcatgtaagaggttgaggatgaagagaagccctgaggccattggagtagctccaaagactatatcttcaaagggctttcaagcagcatcatcatcatgcagataaagaagtgattgatcttggcaatcctttgctgagatttgcttgtatagaagcactaattttgttg

>comp20400_c1_seq1 protein cpr-5-like

gccattgatttcggcatccaccaagacttagattccatgtaaggagagcaagcttcagtggcttcaacgattcttctataagaataaacataagctccatacgcaagggaaaacaacatgataattaaaccagcaactagaaagtccaagcagtttttaagtagctccacttgttttgtatcttgtaactgagttttaaatttttcagctttgaaggaagcttttgaaaaacccattgataatttccatctctccaggagatttgcatgagaattaagctccagttgtctttccttaagctgcatctttctcatgataagaccaatctccaatgtctttaggtcattagaacgagtctgctccatcacagatttttttatagtgcttagcatagaaaattgattgttggcagaattagaagagcttgtccttgcagaagcacaagccaactgctgctctatttgtttatcatgcagaacaagctgttgatttatcaagttcatcttcacattctcctccatttttgagtgagaatggcattgtctgtcagtgctagttctatggggaaatgctgctgggccatctgtatcaccagaaggacatgcagtgtcttccattctatcatgagatgagaatgtgctcacttcaaaagaactatccttatatccagctctttgctgttgttcaatattagtaccatgtggaagtgattctcccccaaccgtagcctcacaaggacatgcagaatctttgagtgcgtcaagagacaataaattacttgtcactgagcaagaacactctctttggagttgttctccatcattttgagatgaaccactgattaatctcaaagtcatcaatgtactccgaaatgatgtctcgaagtttgtcacaaaattatcaaatttgtccccgaaaacatttcctagagattctctgacagccaaaatgcagatcccagatagataatccacagacatcttctctccagctgcactttttctctccagaacctgagcaacgacagcggcaatcgacattcccaaaggaaggccaagggcg

>comp20401_c0_seq1 cinnamoyl- reductase

taccagttccacctcaggatttgaagcagatgcaggaggaaccgggcaagcaacatgaaatacaccctcgcagcctttaactgctgcaagaatggagtcgaaatccaataagtccgccttgaaaactttcagcttttcagctgcattctcaaggttcttcaaatgagcatttttttcatccccaggatttctgacggtgccatgaacattgtagctctcagaaagtagaatcttcatcagccatgaagcaacgaaccctccagcgccagttacacatacattgccacctttttttgccatttttaccttaaaatgaaa

>comp20465_c2_seq1 h-protein promoter binding factor-1

gtttgaaatctaaaccggcaggggcacttgctgtagttcctgcaaatccgaaacctaaacgtactgaggtttcacaacgtagaacaagaaggccgttctctgtggcagaagtggaagcactggttgaagcagttgaaaagctaggaactggaaggtggcgtgatgttaaaatgcgtgcttttgagaatgcagatcatagaacatatgttgacttgaaggataaatgg

>comp20465_c4_seq1 telomere repeat-binding protein 4

gacattttaattaagcaaattaacaactaacctttagccatgtgatgtggtttgggttgtttattacaggataaatggaagaccctagttcatacagctagcattgccccacagcaaagaaggggagagcctgttccacctgagctgttaacccgagttttatctgcgcactcttattggtcgcagcatcagtcgaaacaacacggaaagcatccggttgaacctctgaaaatcgtcgattctcgtgaggagaccgttggagcttgaaggatcagaacccttctgattcttgattgtaacatgttcagtattgtagaggatagtataacaatgaagttgtaaaaatgtgatttgtttgattcagcattcttacgctcactgatttgttatctagtatttgaccgactttgaatagactggtcttttgtaaatgattcattgaataaaaggggtt

>comp20469_c0_seq1 curved dna-binding protein

aaagctcaaaccttgtgtcctgaactggatggtgtatctcaaatggtggccacatttggagtttacatggcttccgaggcgaccattaatggagaactggatttctattcaattcttgggctggacccttctgtggacaaatccaagttaaagaaacagtataagaaaatggcagtactgctacatcctgataagaataaaactgttggagctgatggggctttccgtctga

>comp20600_c0_seq1 gtp-binding protein alpha

gttggcaccggtggatgttcgccgcattccgacggcggcggtggcggcaaaggctgtgatgttgagtaatttgtcgataccaattgttcaaccaatagttaaaaagagcgataagtttaacaaaagattgtcgggtgagatgaagtcgggtattgaagttgttagctctcaggttcaggggtcacatttaggtaaatctggggggatttattcaagaaaagtggtgaattgtggtgagacggaagatgcattagagacagcggacaaaggtggtagtgtatccaatgtggcagatgaaagttcgagtggttctttggcattttctgatggtcgtgatgattcaaatccgttgtctgggagctcggatgttgaggacttggatgatgataataaggcgaatgtatcgtatggtgatttatcggatgccaccacttcgaattcgattgaacaggaggaagaatgtgtagctgaagtttctggccaaggcaataggacatctgtcgtgaagtttgc

>comp20664_c2_seq1 methionine gamma-lyase-like

tccatgacggtgaaggtggcggaggcctcaattgacatgttgacgccaccatgctcgccgaactcgtggcgggcattggccagcgccgccgcgggatcctccgcgcgcggtggcttgttgatcgtttggtgcttcttggcagcggcgaattggtcccactgatcgtcccctgatcgtttcttgctcgacatgttaactatgtcgcggttgaaagttgcgtccgccattgttggtttgttgagcgagagattttgagcgatagttggctctctgggtgggtgtgggtgtgtgtgtgtgtgtgtttgtagttttgtctgagttggagaaagtatagaaagattgggtggcttgttgatcg

>comp20702_c0_seq1 protein chloroplastic-like

actaaagacccgccccattatccgagattggacgacccggatttccggaaatggaaagacaaagaagcagaaatacttgaagatattgagcccattatttgcctcaccaaagaaatacttcactcaaatagatatttggatggagaacgtcttaccccagaagatgagaaagcggtagtagataagcttcttgtgcatcaccctcattctgaagataagattggctgtggacttgattcaattatggttgatcggcatccccaattcagacattcaaggtgcctctttgttgtaagaactgatggtggatggatcgacttctcctaccagaagtgccttagagcctacatccgagataagtacccttctcatgcagaaagattcataaaagagcactttaaacgtggcagtagctgaatcttcttgaagagggttttgcctgatgttgatattgaagtaaaaatatcattccagcattactagagaactttttgatctgggcttatgatatggttgtcgctatggacagctaatatttagaagtaatgcatattcttgcgcttgtgggagaaaatgtgatgtccagtggactcaatggctaacgtttccccatcaactccagttttttacttaaccgtagatataattcgaccggcttggcatgtttatttattttgcgcaacaggaaaacggttcaaccatttcctatttcccttgacatctatacactactatcttgtggattccatgttagatataacaatataaaatcacaaagacatgtttgatgttatggagctctgttacataaatccaggaaactgacattagaaaagaaaaa

>comp20715_c0_seq1 stromal 70 kda heat shock-related chloroplastic-like

ttctgagcatgaaaacgatcaagaaaacgcagtttatcccacttcgtgcgagaaaacacagtgatcacaggagtttgaggcataagcaacacaagcccttaagagtagtgagtgaaaaagtggtgggaattgacctgggcaccaccaactccgccgtggcggcgatggaaggtggccagccgactattgtcaccaacgccgaggggcagcggacgacgccgtccgtcgtggcgtatacg

>comp20715_c2_seq1 stromal 70 kda heat shock-related chloroplastic-like

gtcgggcagattgctaagagacaggcggtggtgaatcctgaaaatacgtttttttcggtgaagaggtttattgggaggaagatgagtgaggttgatgaggagtctaagcaggtttcgtataaggtggtgagagatgagaatgggaatgtgaagctggagtgtcctgctacggggaagttgtttgctcctgaggagatttctgctcaggtcttgcgaaagctagtggatgatgcctcgaagtttttgaatgataaggtcactaaagctgttattacggtaccagcctattttaatgactctcaaaggactgcaacaaaagatgctggtcgtatagctggtctggatgtcctccgcattatcaatgaacctacagctgcttcattggcttatggatttgagaagaaaaataatgaaaccatcctagtttttgacctaggaggtggtacatttgatgtttcagttcttgaggtt

>comp20868_c2_seq1 probable protein phosphatase 2c 4-like

aggattctccattaacatcatatcagctatctccaaatatgatgcttctgtcttcctcaatgcctcggataaagctttcaaaactcccaaatgatcaacggtcatggtgttgcctgcaacaccatcagctaccaaattgctcaatttctcccttaactggcgatcaagctccatcctttccttgctatccaaactttttgatataaaagaagagctattaaccgtattttcacaagattcacctctatcacacaatagcaaccccttaagctccttgtaaacattggcataaagattattcaaaagaaagtccgtagcatcagggccgttaaatccatcataaatccccacaaaa

>comp20933_c2_seq1 propyzamide-hypersensitive 1 family protein

gtagcttgctcacttggcgaatttttttgtatcaatgttatatttttctagactcaggggcatactctcagtgtggtgaggccgatgaggaaaccctgaggcccgatacttgctactgcgcttgcggacaagctcaagccattgagcaaaccgataagcacgccctgctgtaatgtcccctaacatatacaacacccttgatgtgacagtgaggggcaaaggggcatctggctcggtgtttgccatatcaaaatctctgtcttcctcctctttggagagagagatctgaaaactgaagctgccttcttctttttgatcatcagcggccatttctctctttttctctttttgcctattattttttcccccagttgaatggttgctgagaaactgggtacaaaaagtaagtggatctagaggctattttttggctagttggcgttgacggtgccggaggaaatgagcaagagaagcaaggagcccaatt

>comp20979_c2_seq1 glutathione s-transferase

gttttgggtcaagttgttggaacgagagataggggacaagacttacttcggtggtgacaactttgggttcgttgatgcggccttaatcactttctacagctggttcgaagcctatgagacttgtggcaaatttagtgtcgacgagtactgtcctaaattgatctcgtgggcaaagagatgcatggagagagacagtgtccgcaagtccttggctgatcctaagaa

>comp20983_c0_seq1 probable anion transporter chloroplastic-like

gagggcccgtttttgctccattttctcccggaatgggattgtgtggaggaataagagtggaagagtgcgggctgacgtgaaatcggagccgtacgatattgcggaatcgccgccggagtctgttcagtttgaccaagttcttccgacggatgatgatggcggcggcgactccggtgcggttccgtggtgggagcagtttccaaagagatgggtgattgtgcttctct

>comp20983_c1_seq1 sodium-dependent phosphate transport protein chloroplastic-like isoform x1

ggcatccaagttagaagaataaatgttccccaattgtgacaaaaatgagatactatcagggcccatacaggtgctttagatagtattaatctccatggtattgatttaacaggttcctttgacgtgcagttgcgaacgatcagcttcttttctccaggcagcagttcaggatcctcaagaggtgaactttgtgccttattaagccacaaagcaaaccaaactgtgcctagagaaccaaatgaaaaaaaaacagatggccagccaaacttatggatcaacattggtgaaaatgccagaccagttacagatccaagatacatcccactgtaaaccaatgctaatgacctacttctctctgatacaggaacccattttgacagtatattattcattgcaggcatcgcaacaccctcaccaatacccatgaaagcacgaaccacgagtaaaaaaggcaaaccaactttcgcagcaattggtgtaagcactgttgctattgaccaccaaactacaccaaatcctaaaacaaacttgccaccaacagtgtctgcccatatacctcctgcaatctgagtgagaaggtaaccccaaaaaaaggaagattgaatcaaaccaacagttgccggactccaatggaactctgatgacatcggaagtattgcaatactcatat

>comp21001_c1_seq1 wrky dna-binding protein 57 isoform 1

tccaaggagctattatcgttgtacaaacagtaaatgcgtggtgaaaaagagagttgaacgatcctcggaggatccttcagttgtgatcaccacgtatgaggggcagcattgtcatcactcagttggatttcccagaggtggacttgttcctcaggaaggcgcatttgcaaggcacatagcgccttccaacacgcagttttatgatccagggcttcaatatcctcttggaaattctgctcctattacacagtcaccaacctatattcaaggaaaaggtggagaattccataaagggaaagagtcgagttcacagtctccggctgatgaaggactccttggggatatcgtgccaccaggaatgcgaaacggatagtatgagggtttcccatgtacaagaaacaggctacatatagcataagtgttgagaaaacgcaccagttaatacggagcaatgtgctttccaggagctgtatgatgagctcaactcaatctcataagctcatgaaaaataggagtctgacagagctggagaagttatgacaacag

>comp21153_c2_seq1 dehydration-responsive element-binding protein 1d-like

tcttacactttcttcatccagtccaaataattacgtacatggaatattcaccaagctcgaattctgatccctgctttccaacgccgactctcgattttcccgaatcttcatcatccacgctcaaagcgtcgtcgcactcagacgaagaggtcatcctcgccgccagccacccgaagaggagggcggggaggaagaagttcaaggagacgcgccacccggtttaccgcggcgtgcggatgaggaactccaacaagtgggtgtgcgagctgcgcgagccgagcaagcagaagaggatttggttggggacttaccccacaccggaaatggcggcgagggctcacgacgtggcggcattggcgctgagggggaatatggcgtgcttgaatttcgcggactccgtgtggcggttgccggttccggagtcgaaggatgcgaaggatcttaggaaggcggcggcggaggctgcg

>comp21167_c1_seq1 universal stress protein family protein

aacaacggcggcgatccttcgtccgtcttggtcgccttccgtcacaacaatctcagtcttggcgttggggaaggcgttgcagatatctctgaaggaaagcgctaagtggaagccgttgaggcgaagtcgtctaagctttttcttgttcctggatgtttgtttgggatagacgtgcagaagtgtaatcacatccccacaacgaagaatgttgtgaagtgcccactggagagctgttctcgcaatctcagcctcttccaccacca

>comp21174_c3_seq1 6-phosphogluconate decarboxylating 3

tgtggacccagagtttgcaaaggagataattgatcggcagtctgcttggcgaagagtagtttgcctcgctattaatgcaggtatcagcactcctggtatgtcttcaagtcttgcttattttgacacatacaggaggggaaggctacctgctaatttggtgcaagctcaaagagattactttggtgctcatacttatgagaggactgatatgccaggctcattccataccgaatggtttaagattgccagacg

>comp21561_c0_seq1 kd heat shock family protein

tgcaaaactcacatacgtcactggtaacttattggtacgcctaattacaaactaagattcatcagaaacatagtcactggagaccgatcatttagccggagatgtcgatcgccttaactccggccctcttcgtttcctccttaggtacaaccacagtaagcacaccattctccatcgacgccttcacctgatccatcttcgcatcttcaggcagcctgaaacggcggaggaactgcccgctgctcctctccaggcggtgccaagtatccgtcttctcctcctgttccctggtcctctcgccgctgatttgcagaattctgccgtcctcaacttccaccttcacttcctctttcctcagccccggcag

>comp21561_c2_seq1 kda class i heat shock

gggacccccggggttcccccgccagtctatccgagtgttggcgacggcgtaagtatcgcgtgtggtggtcgggaagttggacacggcggaggagaaagggaagccctcgaaggggtcccagagatcgagagaaaacgggtcgaaaacgttgctccggcggccggtgaagaagctcggaattagcgccatatttttacagtggttggaactaaaatacagagattaattcccgaaaatgcttagaaattgcactcacaatgtgtgatgaatac

>comp21583_c1_seq1 digalactosyldiacylglycerol synthase chloroplastic-like

aattttaagccgccggaggcgcggtcggcctcgtcgacggcaaggtcgtcggggacgactagctcgatcgtgacggcggagaaagctttctccttcatatccaagggctggcgtgaggtgcgctcttcagccgacgccgatctccagctgctcaagaaccgcgcaaactccttcaaaaaccgtgccgacagagaactggagaatttcctcaactctgcttcgaaatcagcctttggtgttcccacaattacggcctccgccactctgaaccccgctccgccggcggagatcgatttcgt

>comp21631_c2_seq1 u-box domain-containing protein 35-like

agcaccttaaggccaattttgccaaagataaagcctcctcaactggccagtcttttactgtttgatcaagtatctctgcgaatcggtcattctctattgcagtctctacgagatgtgtcaaacccataggtggtttggctgtgatgatttgcaacaacactacacctaatgaatatatgtctgattttgttcccaacattccagtttgttggtactctggatcaatatagcagaatgtgccggctgctgctgtcatgcaacactgagtgatgctatcagctactgatggtggaaccagcctggatagtccgacatcactgatctta

>comp21631_c3_seq1 tpa: protein kinase superfamily protein

agtttctgtctaagagaatgttggcaggtttgagatcacgatgcacaaggggttctggccttgttcgatgaagaaaattcaaggcagtggcgatttctacagctattctaaagcgggtgtgccatggcaagggtgaagtgccatttttgcgattcagcctgtcttctaaactgccattatccatgtactcatatacaaggcaaccgtattcaggacaggcacctaaaaggataaccatattgggatgtctcatacggctcagaacctcaacctgcagaatgtccatcat

>comp21648_c0_seq1 alpha- glucan phosphorylase l-2 chloroplastic amyloplastic-like

gctggcttcttgctttctggactccattgcaacacttaattacccagcttggggttatggactcagatacagatatggtttattcaagcagcttatcaccaaaaagggtcaagaggaagttgctgaaaattggcttgaaatggggaacccctgggaaatagtaagaaatgatatctcctatcctgttaaattttatggagaagtcattgaaggtccagatggaaagaaagaatgggttggaggc

>comp21653_c2_seq1 annexin d3-like

attattgtcaacttccactctcacaccagctaacctgcactttaatttagatgtattttacaatcaactcataaggaatattcagacaaaagagaatcagacatttgctcctagcaaagccaaaaggaagttcttgtagtcccctgaagtatctccgatcacagcattgtctaggctagtcttgttcgcgttgacatattcacctctgactttcatcatatcgatttcagctcgagttacgatggctctagtcaatgaatcttcatcagtcccaagtcctataattgcagctctcacaacctcagcaaagtgtttctcaggggagtctatgcaccaaatgaccactttcataatcgactcgagaatgccctctccacaagcca

>comp21764_c1_seq1 er lumen protein retaining receptor

tatttcttcgcgttaaaagtttgaactccaaaacccccccagcccaattcttctccctttctgcaaacaaccaaaagaaaaaatattaaaaaaaaataaatctcgaactcttctcagaaattagcaaccatgaatatttttagatttgccggtgatatgacgcatttggtcagtattttagtgctgcttctcaaaatttatgccactaaatcatgctcagggatttccttaaagactcaggaattgtatgcaattgtcttcttggctcg

>comp21764_c2_seq1 er lumen protein retaining receptor

ttgtcttcttggctcggtacctcgatttgtttacagatttcatttcagtttataacactgtaatgaaactggtgttcgttggaagctctttggcaattgtttggtgcatgaggtttcacagagtcgtgagacgatcctatgaccgtgagctggacacctttagacattatatacttgcaggtgcctgtttcctcttggcgcttcttattcatgagaagttctcatttcaggagatattctgggcattctcgatatacttggaggcagtagcaattctaccacagttggttctattgcaaagaagtggaaatgtggacaatctcactggacaatatgttttctttcttggggcctatcgagcattctacatcctgaactggatataccgctatttcactgaggaacatttcagtcgatggattgcttgcgtctctgggcttatccaaacagctctttatgcagacttcttctactattactttatcagctggaaaaataatgcaaaactgaagttgccagcctgacataaacaagatgg

>comp21869_c1_seq1 trichome birefringence-like 18 isoform 1

agcttaaccacaccagcaggagcaaaatcaaatggttcaggtgtttgatgaacaagccaggaggaccatatgcgaacaatcattgtagatgtagatctgaaatagtagcgttgcattttccggttacctctgtttttggggacctcaacctgccacaggatgcacagcattgattccatctgatttcttgcaactgagtcaccaataaaagctaatgtttttcctctcataagctccaaaaacttctttgcgtcaaacctagggaggtcacactgggatggtttccatctccaattctcgtaatctttgtctggtcttccattcccttggcagttctgcatctgagtgagcacgggacaggagttatttgtatacaatggtccagtggggtcataaatccatttcccatggtaaagatcacaatctgtatcagcaacattaggttgactcaagt

>comp22040_c2_seq1 heat shock protein 70 -interacting

ggacaaatctagcttgttcagtgcttcataacacctagctctcttcaagagcgccttgctgtatttcggtgtgacttcaagagccaaattgcactcatggattgcccttggatattcagtaatacccagttgcatatagcatgcagccatattactcctaagataggagacatctatgtggttcctcggaagcaactttatggctttct

>comp22043_c0_seq1 transcription initiation factor iif isoform 3

ggcctcatacgcattccagtatcattgtcaatgaactgtatttgtctagttctagcattatacttattcgtcctctctctgcaaagttttctatagtcttcaatattttcattatgaggcctcatgtcaaatttatggtagattttcccctccacagaaggcttcccttgagatgactcggaaaatacagacataggaagaaaatctgtggacatatccattgagtagtgcttgggcatgtttctatctccagttccagccaactccattgtaaactgggtggaagacaagtcgtcgttgggacgtagtggatcgacggcgacgatgactttggcgacgattgggccg

>comp22082_c0_seq1 dnaj homolog subfamily c grv2-like isoform x1

gaaggtaaaaagcatgtaacagatgtctcagcaatgcaccatcacgtaaagcagcatccctcattgatgcagcagcaacagcatcttcctccgctattgaacgcataactacagccaccgtttctctgacactttcagcaggatgtccaaataatgcaaacaagcgacggcgtaacccagctacaagacgtagcaattcaacaaaaactgggtattgggtagtttcactgtgtggttcacatatcattgcttcaagaacttcaacgacagccattgacaacaatggtgaaacagagtttggttttaatctgttaacaagaacagttaggttactctgttctgcaaatagaacagacttggtatgcatgattgttgcatgttgctctccttttgtatctgataacacacttgtatcccctggaccaccaccaataagcatagcgataagccctattgtttcagcagctactccctcagaaccatttcgaagca

>comp22169_c2_seq1 kda class ii heat shock

gtcctcgagctgcaccttaatgtctccggcggcaatcccgggcatgtcaatgacgaaaacgtaggaattggggtactctttgacgtcggctggcgtcgccgccatggccttggcgtcgcgtatgtaggctttggaagggttgttttggataggcttttcgtggtcgtcggcgaaatccagcatgtcgtggagcgtggagaggagggttgtgtctatgccgaagtttctcaaatccatgattttgttttgacacagaataccaagaactgtttgtgtttgatggatttgtgtgacctctgaagatttactgaaattttgagatgatagag

>comp22198_c0_seq1 9-cis-epoxycarotenoid dioxygenase

tctccctagagatcccaactctgaagcagaagacgatggctatatactcgcctttacccacgacgaaatggcctgcaaatcggagctgcaaatagtcaatgccatgactatgaaacttgaagcctctgtccagcttccatcaagagttccatatggttttcatggcacattcattagtgctaaggagttggcaaaccaggcctaattgttatccgaatcgcgttttcgactgattgccctggagaattaccagagggatggttagatatgcatccccggaatctctctgctgtgatcatatggg

>comp22323_c0_seq2 cryptochrome 1

gaatccagcaaaagttgtgaatggtcgaccttcctgatcatgaacttcccaaggttcgtaaagcaagtcagcgttgaacgaatggacaccgacaccttcagcggttaaaacttcctttacccggtgatccctaacaagcgatatgggatcatacaaatggttgaaaaagagttgcctagctccagtcaatctgataacttcaagaagagaagaaacactatcagtagacctctttgtgacaagagaagtgccaaggctcctcagagagacatcaaggtgtgataaactctgcttaagccaccatcttgaaaccctcccaggatagaagtgcccttcctcctcaggagcccatataaacactgccaccactgcccctgcccttaccccagcagctagtgctggattatcttccactcttagatctctcctaaaccatactatactacaaccacttcctgacattcccatcaacccagaggtgaaaaaaaaaaaaggaaaaagaaaagaaaattaaacggcaaccatggttgctactttcaggaaccttagttaggtcgaattaatcaagcctactcccgttgaccgaccattgaccgcctcgtgaaccggcgaagccgatggagtgaaatatacaggcgaagcgcaaggcgtcgaaaacggcgtcgcatcatccacgccacgatcgtcaatatccaccgccaccaccgactcttcggcagccgactgaaaacattcgtccaagctcacgccgcctccattctcatcatcgatatttttctccgcttctttttcagcagcagaatagagagatatttttgctggcgattcgaggtcttctctctcctcttctttgatcgtaaacaaaaatctcggtggtccaaacattccttgaatcttcaacaaatcaacatcgatgacttcgacatccgattgctgattcgaattgagatcattatttccagaattagcagtcaaggagttgcgctcgaggcgaaattgcggtcttatgcagaagaaatagaagagctccttggatgatgcgatggaggagaaagcggataccgacgaagaattgtgagagacttcttcatctccaccgccaacgaaagtcgtggtttgacgccggacgacgcggcggcgccaaaagacgtagaagagctgggcgaagagaagcagcagtgcgatcgcaaaaattattacgagcgcggtgcctagcttgctcagtccgccaccaccaccgccggtcattttggtaggtatcagccttcaactacacttgtgtatagcaataatcatgtgaaagtgatcggtaaaattttaaacgcagggctatttcactaaatttaacaatcagaatgtagcaagaaaaattgtgctggttctgaagtgatggttgagacaggaaaccggaagtacccggttttggggtgatgaacggataagtttgcgggaaatggagttttcggagtgggt

>comp22323_c0_seq3 cryptochrome 1

gaatccagcaaaagttgtgaatggtcgaccttcctgatcatgaacttcccaaggttcgtaaagcaagtcagcgttgaacgaatggacaccgacaccttcagcggttaaaacttcctttacccggtgatccctaacaagcgatatgggatcatacaaatggttgaaaaagagttgcctagctccagtcaatctgataacttcaagaagagaagaaacactatcagtagacctctttgtgacaagagaagtgccaaggctcctcagagagacatcaaggtgtgataaactctgcttaagccaccatcttgaaaccctcccaggatagaagtgcccttcctcctcaggagcccatataaacactgccaccactgcccctgcccttaccccagcagctagtgctggattatcttccactcttagatctctcctaaaccatactatactacaaccacttcctgacattcccatcaacccagaggtgaaaaaaaaaaaaggaaaaagaaaaggcaaaggaaaaaaaaaagtgaaaaaatgaattcctctagcacttatgcgctatccagagaagaaattcaatacaaacaaaactaagactctccttccctccctccctcctgtgctatctagacaccataattgagtctcttcttggggtaggctgtaagtttggtgcttgctattggattcaaagattgaaactttcagactatagaccataaaaactgcatataagctgcaattcttgatagggatgagcaccaaaaaactggaccttaactttcctaagatggatggatagcaaagaaccagtctataacttttctttgtac

>comp22481_c0_seq1 glutathione s-transferase

acaagccttactttggtggaaacggttttggccttgtggatgtgagtcttataccattctacagctggttttatgccctggaaacctgtggagatatcagtctgatcaacgagtgcccgaagcttgtgtcgtgggcgaagagatgcatggagagggagagtgtgtccaagtctttacctgatcagtataagatttatgatgtgcttatggag

>comp22481_c3_seq1 glutathione s-transferase

aactgcctggaaattgtccctggaaaattctacaactcgaactctctcaagtttcatcgttatcgtctcaaagtttctctctcatgagctttaaattgcaccaatttctatccttattccgccgaccataagaatcacgagtccctatcttactctagttgttactccataaaattttttcccaaaatttcacagcagtggcggatgaattgacccttcttgacctctggagtagcccctttgccatgagagtgcgaatagcacttagagagaagggattggaatttgaatcgatagaagaagatctcagcaacaaaagcccactacttctacaaatgaaccctgtccacaagcaaataccagtcctgatccacaatggaaagcctgtctgtgagtcgacaatcatcgttcaatacatcgacga

>comp22551_c0_seq1 glutathione s-transferase tau 25

aaagtaaggcttgtcaccaagctcagattcgagcagtttgaagcactctatcagctctttcttggctgcttcttggacttcgcctgtaggcccgcacactcgtcttgcagtactatatatctgtatacaattccatatatgatcatgtatataaactggattttgctagaagaacagtgctacatataaatgaaggtgtcaatagtttacctttttgtcgatgtagtcagccca

>comp22551_c1_seq1 glutathione s-

gcttggtgacaagccttactttggtggaaacacattcggctgtgtggatgtgagtctcataccattttacagctggttttgtgccctagaaacttgtggaaatctcagtttgatcgacgaatgcccgacgctggtatcatgggcaaagaggtgcatggaaagggagagcgtgtctaaatctctacctgatcagcagaagatttatgatgtgcttatggagctcaacaattaagaaagatctcaatagaaaatttaataggcatagtactgtttgatctttgtcatcttcattttccagataaagttggatgcagttcatcagttaacatcttggccttttcgctttatatggtagtagaataaagaatttcaagttgtattggtgtgaaattttaagataatattttttctgttttttcccccgaggccagtagattcattatatttttaggtatgtgacaaggttgattatttattaaattataaa

>comp22591_c1_seq1 extracellular ligand-gated ion channel

atttaagatatgcctaccgtcttcccaagcagccatagtactagggacagctctatcccaaaaattgtatgaagagaagttctgtccagcatgaagccatagatcgtgataccagctctattgttttcaaaatatgttactgcaacaccaaaccgaattcaatattatcagacacaggttacattttatgttttattgcatcaagtatgtgaaccatgtatctattaccaagttctttgatcaagaaagtataaaggtccaagtagcctaacttacccaatgcctgtcttttctgaaatgagattgtgctataagcatatgatggaacaaatttggagttgtccagttcatcttcttcatcaccaacatcgtctaaatcagaagatccatcagaactcgctgtgaaaagttgttcatcacctaccctagccactggagtctcagtttcaacggtatcaaatgagtcaattgtagcgcacacatgccactttgctgcaaggcatgttacagcttgtgccttgtgggtaattcttgttgcacttcgcaacaaaatcatgaggccggctagaaggctgacagagcaaaccgctaattctccagcatggtatatattaacatctgcattggaccgtgtggtcatgagaagagaagcaaattgacttgctgtgatgaatatcagagcccacagtatgaatgatctgtacctatggcttattattcgcaaatgtcttctgattctgaggtgttccctcagaaccgactcaacatcagaatcaacttggaagacctgagcaaagtcttggagacgaagaatttgaaggcaacagatgaggcgaaagagaatacagacgaggaaaaacaccactgttctataaaaccatgagcatagctcaaggatacacgcgacagtattgctcacaataacgttgcccaaaaaggggatgcgcgtcccacctgagccataccaccatatcttgtatgcactctcagcagcaaaacagggcagcacgaagatgaataagagcttcaacgatctattgaattgtttggtataaccctttctgacggtctcactctcatcgcagagcttatcaaagaacaagaacctgcggaggccatacttactcacaaaattcgacaaacaaatgaaggagagggcggcgatgctgctgagggagagctgcgcca

>comp22591_c1_seq2 extracellular ligand-gated ion channel

atttaagatatgcctaccgtcttcccaagcagccatagtactagggacagctctatcccaaaaattgtatgaagagaagttctgtccagcatgaagccatagatcgtgataccagctctattgttttcaaaatatgttaccaatgcctgtcttttctgaaatgagattgtgctataagcatatgatggaacaaatttggagttgtccagttcatcttcttcatcaccaacatcgtctaaatcagaagatccatcagaactcgctgtgaaaagttgttcatcacctaccctagccactggagtctcagtttcaacggtatcaaatgagtcaattgtagcgcacacatgccactttgctgcaaggcatgttacagcttgtgccttgtgggtaattcttgttgcacttcgcaacaaaatcatgaggccggctagaaggctgacagagcaaaccgctaattctccagcatggtatatattaacatctgcattggaccgtgtggtcatgagaagagaagcaaattgacttgctgtgatgaatatcagagcccacagtatgaatgatctgtacctatggcttattattcgcaaatgtcttctgattctgaggtgttccctcagaaccgactcaacatcagaatcaacttggaagacctgagcaaagtcttggagacgaagaatttgaaggcaacagatgaggcgaaagagaatacagacgaggaaaaacaccactgttctataaaaccatgagcatagctcaaggatacacgcgacagtattgctcacaataacgttgcccaaaaaggggatgcgcgtcccacctgagccataccaccatatcttgtatgcactctcagcagcaaaacagggcagcacgaagatgaataagagcttcaacgatctattgaattgtttggtataaccctttctgacggtctcactctcatcgcagagcttatcaaagaacaagaacctgcggaggccatacttactcacaaaattcgacaaacaaatgaaggagagggcggcgatgctgctgagggagagctgcgcca

>comp22591_c2_seq1 extracellular ligand-gated ion channel

atttcacatagttcacgcaaggggctagattccacgcttcccagataaactagtctctttcttttttgccactttccgcaatcccattcgtcgctgttccagaaactcgctgaatatacaactcatatcaaatttcatcaacacaaaagatttcatttagacgcagaaacatctcagctcatccttgccagattcacctaaaaaatttgttttgattgtcttgtgatcatatagcttttggttatgggggatgaaactgaaagagctacgccgctgatgcactccaacaagaacaggccgttggcgcgatccgtgtcgtatgcaaaggatgaacttcaggtcttccggacgtggctgaaatggctttgcgtcgatcaatccaatcccatgacggcctgtctgtcgtggttcgtgttcgtggggctggcgatcgtagttcctgctctatcgcatttcgtgctgtcctgcagtgat

>comp22606_c1_seq1 at3g62770 f26k9_200

acagctccccgaaggtcacaaccgtctattcatcccagcactactccctcaacttccctcatcgtttcgatctctctactcccaaatccgatccactcctccacatctcccgtaattccatccctatccctcgccatggccaccatttccagcctctcttctcacccaaaccctaattccaatcccaatttcatttcccccatgctccaaccctacctcgagcagcagcaggagcggatccaacccgacccgccaccggatgacggagccacctacggcgacggtgcggataacgacggccgcgactcatccactactgccaacaataattccggcatggcgaatcctgtccctaatgcgaaccctaacccaattaagctactccacgtctcgttcaaccaggactacggttgctttgccaccggcactgatcgcgggttccgaatctacaactgtgatccattccgcgagatcttccgccgcgatttcgatgggaacggcggaggggttggcgccgtcgagatgcttttccgatgcaatatcctcgcgttggttggtggtggcgattcgccgcagtatccgttgaataaggtcatgatttgggacgatcatcagagccggtgtattggggagttatcctttaggtctgaagtgcgtggggttcgattgcgaagggatcgtattgttgtcgttctcgagcagaagattttcgtgtacaatttcgcggatttgaagctgttgcatcagattgagacttttgcaaaccctaaggggctctgtgcggtgtcgcaggttaccgggtccttcgtgttgg

>comp22649_c0_seq1 chloroplast stem-loop binding protein of 41 kda chloroplastic-like

aaggtgccaataatctcagcacccgcacaatctagaataaggatttggatttgcttcatgcctttttacaagaacatgtacaggaagattcagtttttgtcttaaaatagttttgaacttacagatttgaaagaaaaactcgaaaagttttagtaagataaagaagaataggtgaaaaataataataatgacagaagagagaaccaatggcctagttctggagaacaaggctcttccctagaataatgtcgtcggtcgagaaatcagcttctttcctgtacgttcccctaccaaaatctaggttgtaagagtccgcaagacctgccaccagatcaaattctggtttccatccgagcaggctcttggctttctcgatcgatgcaaagaaatgctggtcacggaaaggaaatggtttcttctttccgaaatcgaactctttagggttgaaatggataatctcaggttcagggaagccagcagccttagcacatgctcttgctaatccatcaaacgtaacatatttctctcctgagatgttaaacacctccttgcttgctttctcattaccaagaaccttaacaaaagccgttgcaaggtccttaacatgaccaagctgtgtaacttgcatccctgagttgggaatgggaatgggacgacctgctttcaaccggtggaagaaccattcttcgacggggttgtagttcaatggtccatagatgtagactggcctcagagaagtccagttcacgcctcgtgatcgcaacaagctctctgtctcaagcttgcccttgtgtctgctctttggatcaactgcatccgtctcaaaatgtggtagataatcagatttcagataaaccccagctgaagagcagtatatgtactgttctagatttggtaaagcgtccaatatcggtaccacttcatctgcctcacgtccattgatatcatatacaacatcaaatccttctgctgagagactgcttctcacgaaatcaaagtcttttctgtctcccttcaagtgcaagatcttagacgcaaaatcagcaaaatcagcatcagattcaccaggcaattgctgagcaatgggtgattttcctctggtgaagagtgtgacctggtgtccttctttcacaagaagtctggacaaaaatatgccgatgaatcgagtacctcccattataagaattttcttggcacttgatgcagtaacacgtaatgctccttttggctgccatagtttcctcttatactggactgaggcggtgagcctggcaccattgaagtcgcagagtgatgatgagaggacggcggaagacggctgtttgtgctgcaccaccaccaaacttgccatcgtgaatttcagtcaccaccacgaccacgaccagccctgatcagtttgttgcaagaa

>comp22779_c2_seq1 small heat shock protein

caaaaattagtttcgcaatcttggcaagtcccattcctaaaagagtttcaagaacaagtaaacattttgatcaatgccattctgcagttctaatccaatgtcaatcggttcgggcaaattggaaaagcatggctagacaaaaagctatagtcgtattcataacgaactcaaaagatacaagaaaatcatatcgcatgaacattcacattctcttgtcactccttgatcctcattggacattgatatcaaaaaccttggcactgccactagcttttggtatggtaatatacaagacaccatccttaacctcagccttaattttctcaaaatcaacattctcaggcaaagcaattctactactatacctcccatagcttttagcagaccagtcctcaccattctcttcctcatttgcatccttctttttcggcaacttctcagccttgacaaccagcattttctcctcaacccaaaccttgacatcctctttagtcatgccgggcatgtcgaacctcatcttgtactctgtttcggcttccttaatctcccatggtgttcttccccggccgtaacctacggtgtctgtcggaggtaaag

>comp22856_c0_seq1 universal stress protein a-like protein

atatatatatgtatatatatatatatatattgtatgtattatgtccatattaaaacccagattaaggctgtagaaaccctgtctgattaatttatttttccatcaagttagagaaattatcagaaatggcaaaggcacgttcagttgggattggaatggactattcagcagcaagcaaatcagctttgaattgggcaatcaataatctgattgaagagggtgatcagattatcataatccatgtcgtttctccaaaagccgatcccactaataagcagctttttgaagatactggctcaccgttgataccactggaagaatttagagaaattagtgtatccaaacgctatgggctgacccctgatcctgaggttcttgatctgcttgagacagtagccaggagcaaactagtgaaggttgtagcaaaggtgtattggggagatgcaagagagaagttgtgtgacgctgtggaacaactcaagcttgatacacttgttgtaggaagcaggggtttgggagcaattaagagggtgttacttggaagcgtgagcaactatgtggtgcaaaatgccagttgtccggtcactgtggtgaaaggggctgcactgaagaaatgaggcttaggattttggctccaattataatattgcagagcttaggctttgcataaatttacataatatttgagtgtaatttgttgtttgcttgtctgcatttatatcattaatttttttaatttcttttttgagtgaaatggtggagttgtagctgcactgcatattgtatggaaagaattccaatgatggcatttgttgtggtacttcataaaatttgaaatcccatgatttgagattttg

>comp22929_c0_seq1 lrr receptor-like serine threonine-protein kinase rpk2-like

ggccgtgccaaggaggtctttgccgccggcttgtgggacacaggcccacgcgatgacttggttgaagtattacacttggctgttgtatgtacagttgacactctttctaaccggccaacaatgaagcaggtggtaaggcggttaaaacaacttcaacctccatcttgtcaagctataaagagtcaagagaggattcatgctc

>comp22978_c1_seq1 hydroxypyruvate reductase

ggggcatgcagcaggtggcgaagagttttcatttaggaatggatccacgcggtttggatcgaaccaaatgggatatccctttatttttcccaggacattgagtgcagcaagtgttgccattccttcacgagtccactttgaagcagaggctatgtgaggcacaactatagcattcttcatttccgcaagcccgggtttcatatatggctcatcctcaaatacatcaagaccgactcggaacataggatttgctttcaaatgctcgacaacagctgcttcatcaataacgggccccctgctgcagtttacaaggatcgcttccttcttcatctttgcaagcctttccttattgacaagatggtaagttgttttatcgagcaccggatgaagacttatcacatcagcctctcgaagcacctcatccattgttgaggcccttttccatgtgacaggctgtt

>comp23107_c0_seq1 e3 ubiquitin-protein ligase sdir1-like

gcatccaatgctcgcaaagtttcataatctaagtcatcaaattccctgtcaagaagtgcaagttggagtctgagaccttgcaaccttcccctggtggcaagagctatagatggtggcatgtgaagtcgcaattcagtgtgtccaagaagaccactagctgcaacagcatgagcttgtgcttgagcttgaagttgctgacaagttgcatacatccttaaagttgttgccatcaaaaagacaccgagcaccagccagagcagaaagttaggagacatttggtgcgagttcagaatcatgaacagcaaaagcaccgtgacaagaaaagctagggagtttgtattgactggtcgagcagcatgcactcgcaccgcacgccgctcaggaataagtcctgggaacccagtttctatatctcctcttgtgcctctgaaaacaaaactcatggtttccaaaaactgcagcaatgccaagcgaaaaagttgtctttggtaagatgcacctggaactggtgccaaaagaatttaaatcactaatatacagcaggcagagatgggttccacaggatggatccgaatgcaaataatcaattcaaaaaatttgattttggtgaagaaaaatttggtggatctctagtgagttgaacgagttgagagagaagaagataatgagtggactcggccgcgttaggctttttctacatgaatg

>comp23107_c1_seq1 ring u-box superfamily protein isoform 1

gcttcttcttcggccgcatgccgacaatgttcctacagcctccatgactgacgaagagatcaatgctcttcctgtccacaagtataaggtgtctggccctcagagcatgggcccatcagtgcagcaggcttcttcttcggccgcagttgagaagaagcaagaccttccgaatgcacaaggaggattaaaggcttccgatgatgatctgacttgtagcgtttgcttggaacaagttaatgctggagaacttattcgtagcttgccatgcttgcatcagttccatgttaattgcatagatccgtggctgcggcagcaagggacgtgtccggtttgcaagtttagagctgggtctagatggtctgggatcactaatggagaaattgatgcttcagacatggtttaagtctttaatatttgttttaacttatgtatacatacaagtatatacatgtatgctgtcccaacttgtaagcactgattacctttatgtggcatcaaaaagattactgaaaatcgtagtttgatgtcaaatgcactgtatatcctctccgtgaaagagcatcagccatgctgaaaatggccatttagatatacaacattctccaatggctagtatcttggatatattg

>comp23157_c0_seq1 heat shock protein binding

agtgaaaataaagtttattacgcaacgtaaccaaccaacagttcttttgcagccagccaccaaactaacaagttacttaatttaccaatctacccctcaatgacccccttaattccctccaccccacaattaccacatggtgattaactgttggtacaactaaaggaaagaggaatgtgataatgcaaaagtccgcctaagaaacggggttagacttgggggcagaaaagtcaaatcacacatgggctgcgcgcttgggtgcagcccgtgcattgctcccactgtcacgacccctcttccttaccgttggatcgtcgctctcctgtatcatatcggccagatcctcaccgaacaattccacaaatgttttctgcagatcttctaagctctcttccttctgagcccccacgttctgctccatcatgttcatcaaatcatgaaggaaatcacccattccctcgtcttcctcaaagagatcgaggaatccggcatcgtacattgacctcttccccttgtcggacaatactgcgtaggcttcttggattttctggaaccggcgtttggcttctccggcggcggaggggtttttcgtccacctatctggatgccatttcagagcgagcttgcggtaagcggagcgaatgtctgagaaggaggcgtccttgcgaatcccaagcacggagtagtagcaataagactcgacgtgggatcctccctctcgatccatacccgaacccaattaacttcggaccttccaattatcgcagattaatcgaacagaagcagttatttatgagcaagcatggactgatacacaaagggatctccacgaattacggtttgcttaagcatctatagaattctggggaacgggcagtcgggaaaacgacgaagcccaattgaatatatacggggggggcggcgagaaaatggaaatgtagaa

>comp23173_c1_seq1 uncharacterized loc101221163

aaaaacctcagttcctctctctttctccccatctgtatctctatctgcaagccgaatttgtgggaaaatgtttgccggaagattcaaagagctgatgaaaaaatatggtaaagtaggattgggagttcatttctcagtctcggcagcctcaattacgggcctttacatcgcgattaaaaacaacgttgatgttgaatcgatgctcgaaaaggttggactgcctagcgttgcgaaggggaaggatagcaaaagctccgatccgccaccggaaaatcctgaaatgacgctgagcactgattcagtaatcagaaacgatcaaacggtgaaggaaaggaatcggacg

>comp23217_c0_seq2 heat stress transcription factor a-4b-like

atttcttagcatcaatttttgagttccatgttatggtaattgaactctttttcttgcttttgatgtatttgtttgtgatgtgtgttcgtgcttctcttgctcgtgtcgtatgctggatgttgtctttagattcttggaaatagagagattaatggaaatattgcggccttctatcgtgttcattagcctgtcgttggaactcctaggtttgtaaatgacaaaaaatgcacctgaaataaatggaaatgatgaagagttgatttaaccaagtctgtggccgtcagggtttgggacagggcttttatgttgaagtagctgagttactggaatcaattagatcttgaaaagtgcggcgtaattggcctttgatgtattagttttgtgacttgtgaaattgcttttgtgctggtagtttaattttgctcaaaaattttctttctacttgtagggttttagaaaggttgatcctgatcagtgggagtttgcaaatgaggagtttattcgaggacaaaggcagctactaaagaatatacacaggcgaaagcctatccatagccattcaggtcaaggaaatgctgtcccattaatcgattcagagagagaggagtttgaaaaggtgattgaaaagctaaaacaagaaaagatttcccttcagacagaagtagagaggcataaacagaagaatagaggatatgaacatcaactgagatctctggggcagactttgcgaaatattgatgaaaggcaacggcaattggtggtcaacttgagtcatttgttggagaaacctggatatgcctcaaatctgatagaacagtccgaatctccaaacaagaagagaaggttgctagctttgcactatttacacgatgaagcaaatttaagggagaaccagggtatgactttcaaagagaa

>comp23250_c1_seq1 at1g70420 f17o7_4

cggaggtctgcaagaagagcaactcaaccggattttccaagatttggcggctcaaggaattgaaggggagatgtaacagtgatgggagggacgcttttgtcttcttaaacagcagtcacgcgccgcccacggcgaagaagcaggcagcgccaaaggcggaggagaaggcagagacgacggagaaagtcgaacggaaggtgaagaagagtgggaagagtaaaccggcggaggcgtcggctcatgaagtgtatttgaggagtaaggcgaaggagggggaccggcggcggtcctacttgccgtaccggccggagctaatgggtttcttcactaacgttaatggcggattaaccagaaacgtacatcctttctgagagctaatgcaattgatgattgaaatttgacttttgattcattcaggtgttgatgatttcgtttatttgtacgtatgaaaaaaatatgatgttaaataacaatgtaagaatgccaaatatgtattgtaagtataaattttttagaattatgtgacagtcttttgtatgtttttgatattacatatagatcccacatg

>comp23255_c0_seq1 zinc finger a20 and an1 domain-containing stress-associated protein 8 isoform 1

agaaaaaacaattatgtcgccttgctccacccaagatcagtactagagagctactaaggcgattggccaactcatacgaagatgcagaaatatacaaagtgagcatcatattcctcttcacgacttttagatcatacttctagatcttatcaagcttttcagctttaacaagaggatttgctttcgctatagcatccctagcagcagtccggtaatcaaatgggcagtcatgttggtctgagtaacgatgaattgaacaaaagaggttcccacacttacacttgaatcctgttatacccacgcgcttgcaacaactagtgcacctacttggtccctccttcttcttctctgaattctggcctggcaacaactcggaggatgactgtgcgggtgcagccttcaactcaacagatccagcttctacattaattgcatcagcgacaacaggcactttctcgttgctacttgagctgccactaacaatgctttcaatggaagatgctgcaaacttcgcctgttcctgtttcaaaatcatgtccttatgacacttggagcacatgttcattgttgtagcactcccaaagaagccacagttgttaatgcagagaatgggtccttctggagtttggcaaccagtctcttttgaagactccataatgcaagtttacttaatctaacgaataatttgagcaaaatgttaaaggtgaacgcggaaaagaggagaggaactcgggcttgatttttaatcagatgatattctccccttctgcctctcagtatttataggggattcattaattaattattcgtaatctgccatagatgaaaaaggaaaaggaaaagagaaagacagagaaagacccagagagagaaagagagagagaga

>comp23255_c0_seq3 zinc finger a20 and an1 domain-containing stress-associated protein 8 isoform 1

agaaaaaacaattatgtcgccttgctccacccaagatcagtactagagagctactaaggcgattggccaactcatacgaagatgcagaaatatacaaagtgagcatcatattcctcttcacgacttttagatcatacttctagatcttatcaagcttttcagctttaacaagaggatttgctttcgctatagcatccctagcagcagtccggtaatcaaatgggcagtcatgttggtctgagtaacgatgaattgaacaaaagaggttcccacacttacacttgaatcctgttatacccacgcgcttgcaacaactagtgcacctacttggtccctccttcttcttctctgaattctggcctggcaacaactcggaggatgactgtgcgggtgcagccttcaactcaacagatccagcttctacattaattgcatcagcgacaacaggcactttctcgttgctacttgagctgccactaacaatgctttcaatggaagatgctgcaaacttcgcctgttcctgtttcaaaatcatgtccttatgacacttggagcacatgttcattgttgtagcactcccaaagaagccacagttgttaatgcagagaatgggtccttctggagtttggcaaccagtctcttttgaagactccataatgcaagtttactgccattgacagcaatatgtacaatgtgaaaggtgatcaagaaagacacaagagtatcaggctcattcaattagtgaagaaaatcagcatgcaggcaatgatattccagaaaagagaaagtcctgtttcaggttcaataatgtaattaagtcttcaatcccaatctgttcatcaacaatttgaacattggggatccaatatgttgcataatgttatcctcaaggtttctaaaaggcaacagcatcaagaaatttggacttcttgacaggcactccgcaaatcctcaggcaaaacattccaatacactctttgaaagagaactgttttttttgatggagcat

>comp23280_c0_seq1 arginine decarboxylase

accttgagaagatccatttcctggtgggccagggtgtcaactccataaggacgaacagaaacgttgccgttagcattaaccgtgaagtacggcgcaccccacccgtccacccggtaaagcaacgccgaatgggcgggtgaccaggccgggacggcggcggtcacggcggcggcggctggtggcagaggagcggggagagtgctatcccagctggcgaaagagtagggaggaggagaaacagcagcgtctacgcagcaagcgagggcaggcatctctttccccaatctacgatctctctcttttcctcaaatgggttgtaagaaaaaaagaaaagaatcaaagaatcgaaaatctagaattagttatgaaatcaaattgaacgatgttgtgaaggtgggggctttagaacccgccgaggccggagccccggctaccccctcaaaagaagcaaaaatggatccgggtcaaaacaaaacggtatccaaaaccacgattacaggacccagaaatccccacgcctatccctcagaagaagaggaatttggccgcacaaatgggggaaaaatggctgaaattgaatcgaaaacaactctaaatcctgacaaagaaccctaccatgtagataattctagatcgagagaagaaattggggagagggaaatggacggaaacctaacggctactgagaggaagatgtgaacgt

>comp23441_c0_seq1 serine threonine-protein kinase-like

agaacagttcctcgtagagaaggtagcaaaacttttatcaaatctaaaatctatgttgtactagtaatggagaaaacgaacaaattttaacattatcctgtggcaggcatgggaactgtacaatgccaagaaccttttggagctggtggattctgaattaaagggcgaattctcacaagaagaagcgcttcggttcttgaaaattgggttgctttgtgtgcaagaaatcaccagactccggcctgcaatgtctgcagccttaaagatgctgaacaacgagattgatacagaagatgttgagatatctcagcctgggcttgttgctgatctgatggaagttaaaatacgtaggaagaatttatccaactttacttgttccccagcatccagcagtacaggcagtttccaggcacgataacataagaatgcatgacaggtttccggacatattgcataaatcgtagttagtgtaggcgctagagatacataagctttcggctgtaattggtgaattcttgaagcagaaggttcattgtacaaaaggatgacttgtgaagtagttctgtgtatcataacatttttttcccaggaagcagctgtaaagtgaaaacatttttgctcaatatcactgttttatccttgttagtacaagatgaggataag

>comp23448_c0_seq1 eid1-like f-box protein 3-like

gaatagaagcttcgccagcgcaggccatccgccgataatcctgctgttcgacgatccttccatcaatgtcgacatcatccgcggcgcgcggtaaatgcagagctcccgccacaggaggcgtctcgccagcgcgcggagcttccggttcaccgccgccgcccggcagagcgtctggatgtcccatttcagacactcgaacaccaaaacaagaactcgctcgttgagtatccccgagtcagccgactcgttcagcctcgtcacctgactcgcgctcccact

>comp23448_c1_seq1 eid1-like f-box protein 3-like

ggcgaagcttctattcttctgcggtggctgcgagtcgactcggaatttcgggctgagtcagccatcgccgggtcatttcgtcgaatcatcccggttttcgaagacttcgggccggagctttctggcgaagagttgcagaggtgatgtgctgtacgtgagcgatccgtgcgagcacccgtcgggggaccagcaagaccacgtcggaatataccgaggggtatttcaggcattcatgaagtcgcggacgcgggaatgtttgataaggaaacaggtggagtttgaggcgggggtgcggtgcccgtattgc

>comp23448_c2_seq1 eid1-like f-box protein 3-like

ggggtgcggtgcccgtattgcggcgcccgaatgtggagcatgacagctgcgcggcttattccgaggaagagcgccgcgcggcggctgggctccaaggacgactcgttggagtatttcgtgtgcttgaacgggcatctccacggtacgtgctggttggtgcccctctcctccgacgaggatcaagacgatagcgaagagcgtagcgacgaggattacgacgaggaggaggaggaggacgatgaggggtctcctaatcgtggtcatacccatgttagcaacggtaattaacccattaattacatcgttatgactgtcagagacatagaattaacttaatctgagaggctatttgggattcggacattcttagcaggtgaattagttgaattagaatgaacttcagttgaaaatagcgcagatagtttttgttggggttcac

>comp23503_c3_seq1 translocation protein sec63 homolog

ctgaatctgatgctccaggttctaatccaagtatgctgaatggctcaaatacttggatctcagtgttgatatgcttgatgtagaaacccaaaagcacaaccaccaaccagagtaacaaaactgtcaggttactgtacgttgaaaagtttgaaatacgtttgaatatggacttccgatacttcccagagcggaagcaagctgaacaacggcaattgacattcttcgtcttctttctaaatgtgaggtataaattgacgaaagtataaggcactagaggcagcgcaattattgtcaatacaaagatggggaacagtgcgctgttctcttcagatgcagccatacgtaaacttctagcttctcacactgcaattctggccggaaaactgccggagtttcgtcggaaagcagacgagctgagctgcgggaaatagaattcgaaattgaggaagcgtagctcccgctgtacaatcagccctag

>comp23508_c1_seq1 translocon-associated protein subunit beta-like

ggaaaagaaaaaggaaagatgcacaaccaaaaaataaataaataaataaaaaaaatcttcaattcaattccgtcgaattcaatggcgaaatctttccctttgtcgatcattgccgtcgcaatcgtgattttcatttgtgcttcaccgtttgtagaatccggtgattcgccatttctcgtggtccacaaaagggtttctcagagaaagctgaattcggatctcgagcggctagccgtctccatcgacatttataatgctggatccgacactgcctatgatgttacactcaatgatgataactgggcccaagagattttcg

>comp23596_c0_seq1 heat shock 70 kda protein 17-like

actgtttcagttaatcagtttcaggtgaaggatgtcaaatgggatgcagaacttggaggtcagaatatggaatcgaggttgatggagtattttgcagatgagttcaacaaacaagtaggaaatggggttgatattagatactctcccaaggcgatggctaaattgaagaaacaagttaagcgcacgaaagaaatcttgagtgccaatttgatggctccaatatctgtagaatctctttatgatgatcgtgacttcaggagcacaataactcgtgagaagttcgaagagatttgtgaagatctttgggaaaaagctcttgttcccataaaagaagtccttaagcattctggactaaatgctgatgatttatatgcggtggagttgattggagg

>comp23596_c2_seq1 heat shock 70 kda protein 17-like

tatgcggtggagttgattggaggtgccactcgggttccaaagctgcaggctaagcttcaggaatttcttgggaggaaggagttggacaagcatctggatgctgatgaggctattgttctaggagcctcactgcatgctgcaaatttaagtgatggaatcaaattaaaccgtaagctaggaatgatcgatggctctacttatggttttgtgtttgagttgaatggtgacggtctttcaaaagatgaaaacaccagacagctgattgcaccgagaatgaaaaagttgcccagcaagatgttcagatctgttgttcacaacaaagattttgaagtttcacttgcttatgaaagtgaagatctgattccgccgggtgccttatctcttacatttgcacgttatgatgtcttaggtcttacagacgctagtgaaaagtactcatcacggaacctttcctccccaattaaagccaatttacacttctctctcagtagaagtggtgtattttccttggatcgagctgaagctgtcgtcgagataactgaatgggtggaagtcccacgaaagaatctcacagtagacaactcaacttctgcttccgcaaacactactgatgctgatgctgggaatactacagatgagagcagtggcaaattggatacaaataatggcaatagtaactcgttggatcccagtgctaatgattctagcagtgcggatcttggtcctgaaaaaaaactgaaaaagcggacttttagagttccccttaaggttattgagaagacaacaggtcctggaatgcctctctcaaaagaatcttttgctgaggctaaacagaaattagaagcattggacaagaaggatgcagaaaggagaagaacggctgaattgaaaaataaccttgaaggatacatttattctactaaagacaagcttgcatctgaagagtttgaaaaaatatcttctgaaaaagagcggcaatctttcattgaaaaacttaacgaggtggaagattggttgtatactgatggtgaagatgcttctgccactgaatttcaagaacatctggacaagttgaaagctatcggcgaccccatcttttttaggtatagtgaactcactgcacggccagctgcatctgaacatgcacagagatacctgatggagttacagcagattgtgcaaggatgggacaaagacaaaccttggctgccgagggagagaatagatgaggtactaagtggaggcgaaaagttgaagaattggttaagtgacaaggaggctgagcagaagaagacttctggatttagcaaaccagcattcacctctgatgaagtatatgggaagatttttgatctccaagacaaggttgcaagtgtaaacagaatccccaagccaaaacctaaagccgagaaacctgccaaggcggaaacagagagtggtggtgatagagcaaataccacaggctctgcatctgaagagacaacctcttcaaaagaccagacaacaagtgattcagacaatgtggctaatgataaggcagatgctgagcccaaggttcatgatgagttgtaataatcatgtagagacatacaaaaccagagctagacaatgcttctgcaatgcaaggacagatataaacgcaactgatgattagatgtagtaatctgatatggttagaaagcgtagagttaagcccagttttgatggtcggctttttcttgcgatccttgggattgtcaatatcctgccagcttgggttcaaagagtacttaggcaaaccattatgcttgtgctattcattaactatctacttgtaatattgaaaaacccacacgaaa

>comp23691_c0_seq1 extracellular ligand-gated ion channel

gtttcatcagccgaaggaatctgagctgaaggagtttcatcctctaattcatcaaatgagttgattgtggcacaagcatgccatttagcggccaaagatgtaactgcttgtgctttgtgcgttatctttgctgcacttcgtaagcagataaaaagccccattaccagggtgatggaggatagtgcgagttctccagctgtgtagatgttaatcctggagcttggtctggttgtaaccagcaaggaagcaaattggctgattgttactaggatcaaagttgacagaataaacagtctaaaacggtggcttatgactcgaagatttcttctgatgctgagatgttctatcaagattgaagcaacatctgattctctctcaaagacctgagcaaactcatccaatcttagtatttgcagataacacgtgaggcggaagaggacgcacacgagaaaacagattgagatgcgatatagccacgaacacatcaacagtatgcaaacaatcactttgctcaagtagatgttgtacaaataaggaatttggtttcctcctgtgcagaaccaccatatcttgtacacactatctgcaaggaaacaggggagaacaaaagctgaaaggagcttcattgatctctgaagctgctgagtatacccttgtcggaccttctcactttcatcagacaacttattaagaaacagaaacctgcgaagaccatactttcgcgcaaaagacgagagactaagaaaagaaattgctgcaaatagcgagagagataattgaattattgcatcgtacggcctctgatggtcaggatcacaatcagagcaagaaaatacgaagtgtgatgccaatggaacaccaacattcagcagaaaaaagattgaccaagaaagcccagctctccagaaactagagtaatcaagaaatacccattttagacctgaactcgaactcttcaactctaaatttgcttcactgaaagaatccaaagcaggtagtgatttgtttttgggcattaaaatctctgccacctctccctccatttcagctctcagttaatttcttcagcagaaaagggtttccaa

>comp23691_c0_seq2 extracellular ligand-gated ion channel

caaaaaattcactaaagtactttcatcattagccagttcttgtctagactaatacaacttcaacatttatgtactcaaataactcatcacgcattcggactatatttgattcgtagacagtttatatgagccacaaactttgacaggaattcttacacaaatgaaattattttacctaaggctgcgcacggtaatagtgcaaactgagaaaattcaaaactcggctgcagtaatgagaataccaaaattgaacttgacgtcttcgactacacctgagcaccagaagatgattacaagtcatgaaatgccaattgtcttgttcaaaatccaaagtgtgagggaaagttgaatggcaaatattgtatgtagccgagtcctatccagcataaatccatacacggtgattccagctctgttgtgctcaaaatatgtcactaaagcttgcctcttctggtaggaaatagtatttgcataaatcggaaccaggtttgtgttgtccagtgcatcatctccatcaccttcttcgctgtcagtatcacaattgggagttactggatatcctgcctgagctgaagcaatctgagctgaaggagtttcatcctctaattcatcaaatgagttgattgtggcacaagcatgccatttagcggccaaagatgtaactgcttgtgctttgtgcgttatctttgctgcacttcgtaagcagataaaaagccccattaccagggtgatggaggatagtgcgagttctccagctgtgtagatgttaatcctggagcttggtctggttgtaaccagcaaggaagcaaattggctgattgttactaggatcaaagttgacagaataaacagtctaaaacggtggcttatgactcgaagatttcttctgatgctgagatgttctatcaagattgaagcaacatctgattctctctcaaagacctgagcaaactcatccaatcttagtatttgcagataacacgtgaggcggaagaggacgcacacgagaaaacagattgagatgcgatatagccacgaacacatcaacagtatgcaaacaatcactttgctcaagtagatgttgtacaaataaggaatttggtttcctcctgtgcagaaccaccatatcttgtacacactatctgcaaggaaacaggggagaacaaaagctgaaaggagcttcattgatctctgaagctgctgagtatacccttgtcggaccttctcactttcatcagacaacttattaagaaacagaaacctgcgaagaccatactttcgcgcaaaagacgagagactaagaaaagaaattgctgcaaatagcgagagagataattgaattattgcatcgtacggcctctgatggtcaggatcacaatcagagcaagaaaatacgaagtgtgatgccaatggaacaccaacattcagcagaaaaaagattgaccaagaaagcccagctctccagaaactagagtaatcaagaaatacccattttagacctgaactcgaactcttcaactctaaatttgcttcactgaaagaatccaaagcaggtagtgatttgtttttgggcattaaaatctctgccacctctccctccatttcagctctcagttaatttcttcagcagaaaagggtttccaa

>comp23746_c0_seq1 phosphomannomutase phosphoglucomutase-like

gggagctggattcttgtggggattgctgggtaagtgaaggctgccttgtcgactccaatgacactccagctgccatagacgcccatatgtacagggccaaggtttcagcagcagggcagggcgaacatggctgggtacacctgcggcagagcattcacaacccaaatatagctgttaatctgcagtccataattccaggaggttgccattccatgacaaaagctctcagagataagtttcttgtagcaagtgggatggataaattccttgatgtttctcaaattgataagtacgccagaaatgggaaactgtaagc

>comp23779_c1_seq1 u-box domain-containing protein 33-like

agtgtaaggaggatgaggtctagaaaaggcaatggagtggtggatcagatactgcagaatgcaccggagttctgtgaggttaagatcgtatgtgaaggaaaggaaatgtctgagctgacggtggattccccttctccgtctccctctccctcaccgagggcaactgatcctaccccaaaattagcccaaggaggagaacaaacaaga

>comp23943_c2_seq1 peptidyl-prolyl cis-trans isomerase cwc27 homolog

gacaaattccttcactttcagcaatcagtgcctatgtccaacgcttgaacagaggttcactggaaaatcaatgcagtgtgttgggataggatatatgtacatttacaacatgtattgagcagttcaagagcacaagtacacttttcaacataaatagaagttgatgctgaaaggaagggcagcaccaccaaaaatgagatcactaatggttcagcacattgtttgctcttcattcatgctttgagacaaatcaggctgctactttgtaaaaaactttggcctcagttcaaccaccgtgttgctgttgtctcagtgacatgatcattgttttgagagctacaacgactcaaatgtcatttttaagaagcaaaaatcatttttaagtaagagacttccccgcccattctcgttgtcttcgcttttcctttgcctggattctgttaaatttctcttttcctttttctaaaagaggatcatgcaccacataatcattcggatcgtcactgcgtgacatatcattcttgccaggctcaggagcaaacttcagctcaactgccatccaacctgacacatcatccttcttggcaccagcatcttcaccatttgatcctttaagtttggttgagaacgctgacttaaatttctcaagtcttgctagaacctcatcttcatgtccctgacgcctgcgcttcttctgcttctgcagttgtctttctcgttctgcttctccaagcaactgcaaatctgcatctgcattagccatgagctctgccctggcttcagagcctattcctttcttcttcagcaacaacttatctgcttttggcctttcatcacggttatcaacattggaccttggtggagatgctgcacggttcctataacctacatgctcattgggcaactctttcttagctggcaggtctcctagctcctgccttcttctcagtatctgctggcgcattcgtgcgtcaaagctagcctcatcttcatcactattttctagagattcagaaaatccaccttttgattcttttcggtgctctttctttgagcttagagcttccctcaccattaactgcgcatccttcgatttctggccttcagctgagttcaattcctttacgaggccctccttcaagag

>comp23952_c0_seq1 nedd8-activating enzyme e1 regulatory subunit-like

gcccaaaacctcaactaaattctctgtccactctctgcgtatcaaacaagcaaattgtcaatggcggaacccaaaaccaaatacgatcgtcaactcagaatttggggcgagcaaggacaagaagccctcgagaaatcaagcatatgtttgttgaactgtggccccacgggctcagagaccttgaaaaatttagttcttggtggagttgggagtattactgttgttgatggctccaaagttgaagttggcgaccttggaaataatttcatggtggatgagtcaagtgttgggcaatccaaggcgaaaacggtttgcgcctttctagaggagttgaatgatgcagtcaaggcaaaatttatcgaggagtatcccgaaaagttaatcgaatccaacccatcgttcttttcgcaatttactttggttgttgccacgcagcttgttgaatcttcaatggtgaaactggatcgaatctgtcgtgctgccaatgtcatgttgatatttgcacgctcttatggccttactggttttgttcgaatcagcgtaaaggaacatgcagtaatagaatcaaagcctgatcattttctagatgatcttcggctaaataacccatggccagagctaaggagatttgcagaaacaattgatttggatacagctgatcctgtcactcataaacacactccatatgttatcattctcattaagatggcagagcaatgggcgaaaactcacaatgtaaaccttccatcaacaagggaagagaaaaaagtatttaaggatttaatcaaggccaggatgattgcatcagatgaggacaattacaaagaagccatagaagcttcattcaaagtcttttctcctcaaggaattagtccaaacctacagcagattattaatgatagctgtgctgaagttaatcccaattcatcagatttttgggttttggtggcagctcttaaggaatttatagctaatgaaggtggtggagaggcacctctggagggggcaatcccagatatgacatcttctaccgag

>comp23952_c0_seq3 nedd8-activating enzyme e1 regulatory subunit-like

gcccaaaacctcaactaaattctctgtccactctctgcgtatcaaacaagcaaattgtcaatggcggaacccaaaaccaaatacgatcgtcaactcagaatttggggcgagcaaggacaagaagccctcgagaaatcaagcatatgtttgttgaactgtggccccacgggctcagagaccttgaaaaatttagttcttggtggagttgggagtattactgttgttgatggctccaaagttgaagttggcgaccttggaaataatttcatggtggatgagtcaagtgttgggcaatccaaggcgaaaacggtttgcgcctttctagaggagttgaatgatgcagtcaaggcaaaatttatcgaggagtatcccgaaaagttaatcgaatccaacccatcgttcttttcgcaatttactttggttgttgccacgcagcttgttgaatcttcaatggtgaaactggatcgaatctgtcgtgctgccaatgtcatgttgatatttgcacgctcttatggccttactggttttgttcgaatcagcgtaaaggaacatgcagtaatagaatcaaagcctgatcattttctagatgatcttcggctaaataacccatggccagagctaaggagatttgcagaaacaattgatttggatacagctgatcctgtcactcataaacacactccatatgttatcattctcattaagatggcagagcaatgggcgaaaactcacaatgtaaaccttccatcaacaagggaagagaaaaaagtatttaaggatttaatcaaggccaggatgattgcatcagatgaggacaattacaaagaagccatagaagcttcattcaaagtcttttctcctcaaggaattagtccaaacctacagcagattattaatgatagctgtgctgaagttaatcccaattcatcagatttttgggttttggtggcagctcttaaggcaactcttaacatcttcacctatcaaat

>comp23952_c0_seq4 nedd8-activating enzyme e1 regulatory subunit-like

gtacaaattttcttcttcatggtggatgagtcaagtgttgggcaatccaaggcgaaaacggtttgcgcctttctagaggagttgaatgatgcagtcaaggcaaaatttatcgaggagtatcccgaaaagttaatcgaatccaacccatcgttcttttcgcaatttactttggttgttgccacgcagcttgttgaatcttcaatggtgaaactggatcgaatctgtcgtgctgccaatgtcatgttgatatttgcacgctcttatggccttactggttttgttcgaatcagcgtaaaggaacatgcagtaatagaatcaaagcctgatcattttctagatgatcttcggctaaataacccatggccagagctaaggagatttgcagaaacaattgatttggatacagctgatcctgtcactcataaacacactccatatgttatcattctcattaagatggcagagcaatgggcgaaaactcacaatgtaaaccttccatcaacaagggaagagaaaaaagtatttaaggatttaatcaaggccaggatgattgcatcagatgaggacaattacaaagaagccatagaagcttcattcaaagtcttttctcctcaaggaattagtccaaacctacagcagattattaatgatagctgtgctgaagttaatcccaattcatcagatttttgggttttggtggcagctcttaaggcaactcttaacatcttcacctatcaaat

>comp23952_c0_seq5 nedd8-activating enzyme e1 regulatory subunit-like

gtacaaattttcttcttcatggtggatgagtcaagtgttgggcaatccaaggcgaaaacggtttgcgcctttctagaggagttgaatgatgcagtcaaggcaaaatttatcgaggagtatcccgaaaagttaatcgaatccaacccatcgttcttttcgcaatttactttggttgttgccacgcagcttgttgaatcttcaatggtgaaactggatcgaatctgtcgtgctgccaatgtcatgttgatatttgcacgctcttatggccttactggttttgttcgaatcagcgtaaaggaacatgcagtaatagaatcaaagcctgatcattttctagatgatcttcggctaaataacccatggccagagctaaggagatttgcagaaacaattgatttggatacagctgatcctgtcactcataaacacactccatatgttatcattctcattaagatggcagagcaatgggcgaaaactcacaatgtaaaccttccatcaacaagggaagagaaaaaagtatttaaggatttaatcaaggccaggatgattgcatcagatgaggacaattacaaagaagccatagaagcttcattcaaagtcttttctcctcaaggaattagtccaaacctacagcagattattaatgatagctgtgctgaagttaatcccaattcatcagatttttgggttttggtggcagctcttaaggaatttatagctaatgaaggtggtggagaggcacctctggagggggcaatcccagatatgacatcttctaccgag

>comp23974_c2_seq1 dnaj heat shock n-terminal domain-containing family protein

gagagagagagagagagttgttgatggttgattcttgcaatccatccatggctgctgcgtctccgtctcttgccggcggagagaagaaacactggtggctcaccaataaaaagatggtcgagagatacgtgagggaagctaaaatgttgattgcgacgcaagaggaaagcgagattgcgacggcgttggggctattggaggcggcgctagcgttggcgccgcggatggaggtggcgctagagctaaaggcgcgctgtttgctgtatctccggcggtttaaggaggtggcggatatgctgcaggactatattccgagcttgaaaatggtcgcctccgatgat

>comp23974_c4_seq2 dnaj heat shock n-terminal domain-containing family protein

gagcttaaaaatggtcgcctccgatgatacgtcgtcgtgcggctcatcggataactcgtccactcagctatcgagggagcgagtcaagcttctctcctccggcggcagctcactgagtgggaacgagccggctttcaaatgtttttctgtgtccgatttgaaaaagaaggtcatggctggactctgtaaaaacgtcgagaaagaagggcaatggaggtattccgttttgggtcaagcatgttgccacctgggcttaatg

>comp23974_c5_seq1 heat shock protein with tetratricopeptide repeat isoform 1

atgtctacatgtaccaaaaatagaaaattaagaaggaaggaaaaattgcgaagggaaagagacgaagagaagaacaagatagtagtactagtaacaaaatttttatccatggcggtactactagaagttttctaatttttacattgccactagaatcagattctcccgccggtctattaacattttcaaaagtacaaagattattccacttttatcaccataatttacgactaatcaacagcttaatgcttcatattttactgggattggacgattgaatccagcctgagaaagcagattgccaacaactgcaagatccctgcaaaaaactccttgaaaa

>comp24179_c0_seq1 derlin- -like isoform 2

ctcaggagccatgtctacacccttgcaatactacaactctatccccccagtggcaaagacatatgctgtggtatgtctattgaccactggtgcttctcagctggagctttacaatctcgggaacattacacttatctattcagacgtttttaaaaggtttcaggtttggaggctcgtcacacccttcttcttcctcggaccattttcgcttaactttgcgctacgtctcttaacaatattactctatggagttcagttagagaggggaccttttgacaagaggacagcagatttagtatggatgtacatatttggagcaatgacactactgg

>comp24205_c2_seq1 abc transporter c family member 4-like

agttggaatcgcgttactgctgttatatgtttatttaggagtctctgtattggtgtcgttggctgcagtcgttggagttatgttcctaactcttacaattactcgcaagaacaattcgtttcaattcaatttgatgatgaaccgggatgcaaggatgaaggctaccactgaattgctcaataacatgcgcgtaataaaatttcaagcttgggaagaacatttctataacaaaattcagtctgctcgtcaaaaggagtatagctggctcagtaaatttatgtacaacatttctggaaacttaattttgctatggggcatgccatttgcaatagctgcactcacatttggagtcgcaactttgttgcgagtatctcttgacgctgcgacagtgttcacagcgacatcaattttcaagattttacaggagccaattcagaatttcccacagactctcatttcagtttcgcaagcaatcatatctttgggaaggttggatagctatttgacaagccgcgaattggaagataaaaccgttgagagggaggaaggttgtgatggtaaaattgcggtcgaagtgaaagaaggcactttcgtgtgggatgatgaaggcggtcagcatgttcttgaagatttaaattttgaggtaaagaagggagagcttgctgcaattgttggaacagttggatcaggaaaatcgtctctattggcttcagttttaggtgagcttcgtaaaatttcaggaaaggtcagagtttgcgggactactgcttatgtggcacaaacatcatggatacagaatgcaacgatccaagaaaatatcctgtttggttcatcaatggacaatgaaaagtacacgaatgtgataaaggtatgttctttagaaaaagatttggaaattatggaacatggtgaccagacagagattggagaacgtggaattaaccttagtggtggccagaagcagcgaatacagcttgctagagcagtatatcaagactgtgatatctatctgctcgatgatgtattcagtgcagttgatgctcacactggaaccgaaatatttaaggaatgtgtaaggggagctttgaaggacaaaacagttctactcgtcactcatcaacttgattttcttcataatgcagatctaatattggtcctgcgagatgggaagattgtgcaatccgggaaatttgaagaacttcgtcgatcagaactggactttagtgcacttgtagcagctcatgagacctccatggaattagtagaagctaacctgaccggctctggtataaatgacaaacaagcaccagaatcaccccgcgaagaagtaccaatatcacctcgcgtagtttcaagccatagtgagctcaatagcgaacaagagcaaaccaagtccaatgagggtagctcgaagctcattgaagatgaagagagagaaactggccgagtcagtctaaaggtctataagcaatattgcactgaggcatatggatggtggggagtaacagctgtgatgcttacctctatattatggcagttgtcccagatgtcaagtgattattggctggcttatgagacttcagacaagcgtacttttgtagcctctctgtttgttggcatctactcgggcatagttgttgtttcttgcgttttcctaggagccagatcaatccttgttgcatttttgggtctcaaaacggctcaaagttttttcaatcagatattgaatagcattctgcatgctccaatgtcattctttgatacaactccttccggtagagtgttaagtcgtgtttcatctgatcaagtcaacgtcgacatcttgattccattgtttttgagtgtaaccattgttatgtacatttcattgctcggcacattggttattacatgtcaatacgcttggcctacaatattcatcataatcccactgatctggcttaacgtttggtatcagcgatattatattgcatcatctcgtgaactgacaagactcgaccagatcactaaagctccaatcatccatgatttctccgaaacaatatcaggtgctatgacaatacgttgcttcagaaagcaggatagattcttccaggggaatattgacagggtgaacttaaatctgaggatgagttttcacaacaatgcatcaaatgagtggttaggcttccgcttggagatgatcggaagttttctcctatgcgtgtccactgtttttctgatcttgttgccaagtagcatcattcgaccagaatatgttggactgtctctttcatacggccttccacttaacgctgtactgtactacactgtctaccttggttccttccttgagaacagaatggtttctgtggagagaataaagcaattcataaacattccatcagaagcagcatggaggaaagcagattcttcagcctctccagattggccaaatcgcggtgagattgagataaaagatctgcaggtcaggtatcgatataatactcctttggtattgaaaggattgtctctaagcatcaatggaggtgaaaaaattggcgttgttggacgaactggaagtgggaaatcaactcttatccaagtgttctttaggctagtcgaaccttatgctgggacgatagtaattgatgggattgacatatgcaagctcggtcttcatgatcttaggtctcgttttggaatcattcctcaggagccagtcttgtttgagggaactgttagaagcaatatcgaccctcttggattgtactcagacgacgaaatatggaagagtctcgaacggtgccaactgaaagaagtcctggcagcaaagcctgaaaaactcgactctctggttgtcgactctggggacaactggagcgtggggcaacggcagctactatgtttgggacgagtcatgctgaaacgcagcaagatcttgttcatggatgaagcaacagcatcagttg

>comp24205_c3_seq1 abc transporter c family member 4-like

ggatgaagcaacagcatcagttgattctcaaacagatgctgtcattcaaagaatcatccgccaagacttctccgcctgcacgataatcaccatcgctcataggatcccaactgtgatagactgtgaccgcgttttggttatagatgatggactagctaaggaacttgacagtccatccaaccttctagagaggccctcattgtttggagcattggtt

>comp24205_c4_seq1 multidrug resistance-associated protein 4 isoform 3

gctggcaatcaacggccatataattcacaatctgtcctactccatgagcctgtctagacgaggaggacagtctgagccccttcttatacagagcagtaatcaaagtcgaacgaataagcattcccagcttttgagataaaaaattgaactgatgggagctaagaacttcgatgacctttgaaataaatagtgtcaatatcagaagaaaaccttgatacaaattgcttctatcaccagaagtgaaactaacgaaaccttggattaagactggcccgatgtacatgacagccaaacgtacaacagcaagaaaacctgtgaaagccagatccttccagaaacatcggagcagcatcgttcttacaggattctttgagttctcactaggctcaggccaatgaagttcaaaaagttcagccattgcttgagcctgatgatcaggcggaagcgaggggacttcatccatctgcagaggagacttgtacccttttctcagaatcggattcatccaatgccacactgctctagaaaacaaggaagctgtagcatagccactcaaattatccactgaagtggattcatctaattctcgtcttgaattctcatcaacatcttcctcaacatttatactaatccctgttgatcccttgatggcaacaataaacagaaaaccatagagggggaagctcgccaaggagaatatgtcatccactttcatatttggatcaagactttcaccaccagacactaaatgagtgagagccgtagcagaaaacaaacacacaagaacaaagctcacgatccaatacactcgaaggggcaatggatgaaagacagctccaaattttttctcatgtgtaataaggaccaaaagtaccacattagtcattgcttgaaacaatttgaacaaaacctctaataagttccattcaga

>comp24222_c0_seq1 kda class i heat shock

gctgcgttcgccgcttatctgaagcacattcccttcttcaacctccaccttcacctcctccttcttcagccccggtaaatccgccttgaacacgtgtgcttgcggcgtctccttccagtcgatgcgggtggctgcgaactgcgacgtcttgttggaggagctgagctgccagtcgcggaaagggtcccatacgtcgacggagaatggatcgaagatgctgcttcgtcgcccgaaaacacttgggatcaacgacattttcgcctttgcaattgaaggtgctgagttctgaaattgagaaattgaaggcgagctttctatcgaaatcgtatgaa

>comp24223_c3_seq1 double clp-n motif-containing p-loop nucleoside triphosphate hydrolases superfamily

ggagagaagagaaaagggttttggtcggaagcataagagagtaaatgggaatgccagaaattgctagggtttataacgcctctctgagtttcgcttggtggagaactcggagtggagtaaatcccaccggcagtgttataacactggaaaacagcagaaactgagttacaatcctccaaattgttcttgacagaagtgctggaaaaaccagcttctttcataaccctactgacactagggtcatctaagatggatagtatgagctgttccaactcaactttaatggcgatgagaggttgttgctgctgttgttgttgctctatacagcctctcctctgatgagcctgagctcgtttgagggcggcgatgagagcattggataacgagggctgagcgtggaggagagggccgggtgtggccgggaggcggttgagggcgacgttgaagcaaagttcaagggctctgcactgaagaggatgattggattgatgaggctgagatttgaggcaagccctacggagaagactggctcttgagctcagcaaagtggcggcgacatgtagcggagtgacctgagcgtggccgcgccgccgggccaggctgagagagtgcttcagcacggaagcagcctccgcggagagggtctg

>comp24250_c0_seq1 defective in meristem silencing 3

gttgaaacatgtcctcctgcctggtactgagttgcatacgatgagctttcagcaagaaaacggacaaattctctcttctttgtctcatatttgaaccttgcatggtctaacagttcctgttctctctgtatatcttccaacgttcggtccttcttccattttgtctccttcaaccgattttcaatatcaaagtagttcacagggaggctgaaccttttcgcgtcacaggggaacttgacatctatatctccctgatgatgacccaaagagaatacaccaggacttcttagcactcctccatctagagatatggctccagttgtcatacaagaccgtgcatttagcatgtcttcccttgatttgtacacttgcaaatttgagaagaggttatagaacagtgtctctctaagactgtgcccagttttcgagaaacaatataagttattgttgtcaatagtgaccatgtttacagcaaaaccaaggaatccaggaggagtgtccccgttgattaatcttggcttcagaagattaagcctccgttgatgatcatcagctatcaactcaccaacatatggtcttagattttcaagacaaataacaagaaatcggtcatccagaggcctcccaacag

>comp24250_c4_seq1 defective in meristem silencing 3

tagcaacaacaccttcataggatttgcagaccactgctagcatggtttcaagacccaagtactctgaaagaagcctgctaaggttagcatcatcaacttttccaagtgtagcaacaacaccgaccacatccttcgtcaataaatgatcagaaagttgagcttcacttttcatcttacacaaaacggcagctgcagacttctcgtacttcagaatatgctgaattgtttcttcctcactccctgaatatgcatgattttcattttcctctttggacaaacttgtcatatgatgcttcccaatagcaacttgcatatcaaggattgagtcctccaatttgttcctcaaagtttttaagtagttgacattgtcctcatgatgcttaatcttttctcccagttcctgcagatcatcctgaagtttcttagaattgtttataactgactcaacatgctgaccatgcactccattttgcatgtcgtccctagg

>comp24287_c0_seq1 heat shock protein with tetratricopeptide isoform 1

taatttgtcttgttgcttgtcaatttaaaacaatggatcaaacatatacatccataaagaagccaaaagaataccaaaaccttatcagggtgatattttaatgcagctttgcgataagccttcttaatttcggatgcagaagcagatggatcaactcccagaatgagatacatattgagaggaatctcatttctcgctgattcctccatttctgaaagtctcaatctagcttgccgaagctcattcacgcaatccactttatcaggtcttccagattgatttgtcttcctatccacttcttcactaagcagggatacaagtttttgaagatctgctactgcctgcccataatctctaatcatctcgtatagcgtcgctcgcctggaaatggccttataataagttccatcaagggcaatggaaaggcaacaatctgcaatggcatctaaaagttggcccatagaccgatatgcagcggcacggttacaaaaacatatagctgcaaaaggacgtgattcaacagaacatgatatagcagcagtgtagtgctcaacagcttctgcatggtttcca

>comp24287_c0_seq2 heat shock protein with tetratricopeptide isoform 1

atccagaaaagtcatcatgtgtaccaattatactggtttctctgtgaccctttgtttgagtttccatgtgatctccaagcttcttgccaatgtctcctaccactcctctcaaatggatagttatgaaaatccgaaaaaatatttgaagtattactggcattggctctgtttggagcatttcgcatctcttcttccagatcatactgcgagcgcttggtagggtctgaaagtactgcatatgcctctccaattattttgaagagtctgtctgcatctttatggacctcctctgctatttccttccatatggcgtcatccggattttcatttcttgccaaggattgaccagccttatcagggtgatattttaatgcagctttgcgataagccttcttaatttcggatgcagaagcagatggatcaactcccagaatgagatacatattgagaggaatctcatttctcgctgattcctccatttctgaaagtctcaatctagcttgccgaagctcattcacgcaatccactttatcaggtcttccagattgatttgtcttcctatccacttcttcactaagcagggatacaagtttttgaagatctgctactgcctgcccataatctctaatcatctcgtatagcgtcgctcgcctggaaatggccttataataagttccatcaagggcaatggaaaggcaacaatctgcaatggcatctaaaagttggcccatagaccgatatgcagcggcacggttacaaaaacatatagctgcaaaaggacgtgattcaacagaacatgatatagcagcagtgtagtgctcaacagcttctgcatggtttcca

>comp24291_c2_seq1 molybdenum cofactor sulfurase-like

gatcaggacaagatactttctcaaatcactcactgtctatccaataaaatcttgtgctggcttcagtgtggagagctggcctttaagtaatactggattgttgcatgatagagagtggcttctcaagagcttgagtggggaaattttgacccaaaagaaggttccagaaatgggcttcattactacactcattgatctcaaattgggactacttattgtcgagtcacctcattgcaaagataaactgcagattgagcttacatcaggtcaatctattggtggaagagaggtgatggaa

>comp24291_c3_seq1 molybdenum cofactor sulfurase

gtacccttttacggagttatgcttcccaacacgatatgtgttcaaatagggatcagagtatactagggatgtgcagagatgtagaaaccagattgaattttgtcaatgaagctcagtttttgcttatatccgaggaaagtgttgctgatctgaacaacagattaagatcaaagttgcataatggctcacataaccagcctgctgaagttaatcctatgagattccgtccaaatttggttttctctggtggtaaaccctatgaagaagatggatggagaagtctgaagattggaaagagaaactttacgtcattgggtggttgcaaccgttgccagatgatcaatatgacttccatagatgggacggtgcagagatccaacgaaccattaaccactttagcatcttaccgaagaataaaggggaagatatattttggaatattgttaagattgtgtgatagtatcaaggaagatgcacagctttctgtgggacaagaaatagttgtgaacacagattagctttgagagttaaatcagtctagacct

>comp24291_c4_seq1 molybdenum cofactor sulfurase

gctatcgagaggtggaaaaactggcatccttgtccaatattcagctaaggacagggtgcttttgtaatcctggcgcatgtgcaaaacatcttggtttgtctcatttggatcttctttctaacattgaggcaggacatatttgctgggatgatcgcgatatattgcagggaaaaccaactggagcagttagggtatcatttggttacatgtcaacatttgaagatgccatgaagtttttgaagttcatcaagagttcatttgtacctcaacagacacatctggatttatttcgagaaaagccatttcatcctgcaa

>comp24523_c0_seq1 heat shock transcription factor a2 isoform 1

gaagaagcatttgctgatgcacatcaagaggcggaagcaaaattctcaaacaaatcagcagcaatcgtggctgggttcaactaagcatggagtggaggcagagcttgaacaattgaaagctgatcaaaatacattgcagacagaagtgttcaagctaaggcagcaacaagagatcacacagagttatctggcaactgttgaaaagcggcttcatgttaccgaagtgaagcaaaaacatatggctttgttcatgaataaattcctgaagaatcctctgctgttgcagcatatcattgagaaattgaagaagataagggcacttagcagtggagaaatcttgaaaaagagaaggttggcagctgctgatatgggtgatggcagcttaatggaagcaatgaaagccgttgacattgatgagattactaatgcgagaattgatgataagaagatccaacttcaagagaagctgacaacaattcaatcagatattcaaacattgttttcttctgactcaccaggcagtcctgtgcaggaacagaatgctgaaaattcctctgagacaaatagttctgatgcttgctctgagaattttgttctatgggagaaactcatggaagatgacatgatttatgaggatgaagcagcagcatccaagcaacaatctgacattgtttcagaattggagaatttagttgcaaagccatctgattgcggtatgcaaatgaggagcctagtagagctggtcggttgtctggcatctatagcttaaatttgtggtatatgtgtactaagtgcaactctcgatgcaaggaggatgaaattggagatgctgggcagactagagcgttgtttttgtcgttaagatgaaaaggagggttcaacagaaacttaggattgacaacagaaggggcgctgccattcattaactagaagctgagaatgccgttgcagctcctgtagtttgtcattccccttgtgtattcactctctctggcgaacttcagctcattttaaatcttcttaatttacgtcttgatgagaattcttattctctgtaaatatatccttggctagacagaatggcagaccttgttatcaagttggaaaggctataatactgcttagtgtagagtattagaagtgctattcttgacgcttgtgactctgttcttgtgcaactatagttttaacaattttcagatgatatgtatcagtagtgtatgcacagttctaatgcacatgttgccgttctcgcttattgaagcata

>comp24536_c3_seq1 leucine aminopeptidase

gaggctgttgctgctgcagcaaagtctgcgcaggccagtgatgttgccatcacacttgcttcttcagatggagttgctacagaattgaagccaagtactgcttcagcaatagttactggagctatattgggaacatttgacgataacaggttcaaatcagaatcaaagaaacctgctctgaaatcaattgacattattggtcttggtagtggacctgagatagagaaaaaactcaaa

>comp24640_c0_seq1 protein proton gradient regulation chloroplastic-like

atctgtagtattggaaaaagattctgtatcagctcactcatcacttgcattttgcagaaggaattgacatttgaaactgaagaaatgcagataaaatatgaacagcagataaaatggaagtatccatcttaaatttagagggaaattgggatagaaaggggtatacttgggatgttatgaatcaatctaacacatctgtatatcagagcaaatgaaaacagtaaatcgacataggcaggaaggatcctctgttcaggccagaaaaccaagtttctctccatttttcttggccagtcgaatcagcccttgccgttgcttcgcgtctgcccctattgacttgcagaactctgttatcacctgtgagtgtagagcaatggctttgcccctgagctggttgaacctcttcttgccaattatgtttcttgtgatgacaacaattggagcaaaaagaccctttccttcattaacattcctcatcatcggctgcggtcggactggccgcgctgccaccctcacttgggccggcgccgccttggcctgcagcacggcatggtcttcgccagctatggaagtgccccagctaccaagaaaagcagaagcagaaactgaggtaacagccattgaaggataatgaa

>comp24640_c0_seq2 protein proton gradient regulation chloroplastic-like

atcaggccagaaaaccaagttgttcaggccagaaaaccaagtttctctccatttttcttggccagtcgaatcagcccttgccgttgcttcgcgtctgcccctattgacttgcagaactctgttatcacctgtgagtgtagagcaatggctttgcccctgagctggttgaacctcttcttgccaattatgtttcttgtgatgacaacaattggagcaaaaagaccctttccttcattaacattcctcatcatcggctgcggtcggactggccgcgctgccaccctcacttgggccggcgccgccttggcctgcagcacggcatggtcttcgccagctatggaagtgccccagctaccaagaaaagcagaagcagaaactgaggtaacagccattgaaggataatgaa

>comp24644_c1_seq1 hydroxyproline-rich glycoprotein family protein

tctggagtaactgtaccagatccttgacatgactcccgttcatgaagcacaatcttgtcaaggtccaaaagtttgggagggttaccacttctgaactcaagaaaaaatgggtggatagaagcaaactcacggtcaggaaaaggtgatgatgtgcctgaaccagagatgcctgagctaggtgagatcaaatggctgattggactgccaggttgaagctgataggactgaaattcgtactgagacagagggtatctctgaccagcttcaccagcctggaggttgggttcaagaagtcgcgcaaatggcacctcaggagaggaaggtgtagtcatgtggacagactctgggggaggagtatagggggcagtagatggttcagtggtgaaggtggaaaagaccggaggagaaactaactggggctcatgagcataaggaccaatagcaaacattgaggcaggaccaccaggagaatacatgcttgcagatacagaagtcagtgataatatgccagttggtgactgggtagcagaaggaggttcagatggaacgaaggatacaggagaggaaggaggtgcgacaaaaggcagtactatcgatggtggttgggatgaatgttcagctgcggaagcatcagctcctgtggcagtagtttcagaaacaagaacagcatgcccaattcgcttattcttgcttgatccaaaacaccagtaaaggctccagaagcttccccaccttcttttctgcacgaccataagcaatggcaatcaagtaccaagtaacataattggatatcgtacttctgagataacaaaattctgacacaggatactaagtaccgaaaaaaaagtacaagcataaccatgagtt

>comp24644_c1_seq4 hydroxyproline-rich glycoprotein family protein

tctggagtaactgtaccagatccttgacatgactcccgttcatgaagcacaatcttgtcaaggtccaaaagtttgggagggttaccacttctgaactcaagaaaaaatgggtggatagaagcaaactcacggtcaggaaaaggtgatgatgtgcctgaaccagagatgcctgagctaggtgagatcaaatggctgattggactgccaggttgaagctgataggactgaaattcgtactgagacagagggtatctctgaccagcttcaccagcctggaggttgggttcaagaagtcgcgcaaatggcacctcaggagaggaaggtgtagtcatgtggacagactctgggggaggagtatagggggcagtagatggttcagtggtgaaggtggaaaagaccggaggagaaactaactggggctcatgagcataaggaccaatagcaaacattgaggcaggaccaccaggagaatacatgcttgcagatacagaagtcagtgataatatgccagttggtgactgggtagcagaaggaggttcagatggaacgaaggatacaggagaggaaggaggtgcgacaaaaggcagtactatcgatggtggttgggatgaatgttcagctgcggaagcatcagctcctgtggcagtagtttcagaaacaagaacagcatgcccaattcgcttattcttgcttgatccaaaacaccagtaaaggctccagaagcttccccaccttcttttctgaaccgaagcatgagaactacgagtctctccagatgcgatcacagtggcggcggcgtttatcgtctccaacgcgtcgcttccattcactcctcttctcatcgttcacgcaatcaatcaaacaaaatcaaactcaaaaacatactcatcaaaggaaaaatcatggaaaaacaaatgaaaattcaatttttatgcgtcaatttttttatgagaaaaggaacgga

>comp24652_c2_seq1 calcineurin b-like protein 4-like

gtttatatataaaaagaaattcatatacaatcaaccgaaagccacgattttagatatatcaacatgcctatcttgatgttcacaaggaactattagcatctcccagaacagcaaaacatgtacagcttgagagacaagggccacttgtttatagttatgtagcattgaagtaaccctatattttgcaaaaactgcctatgaacaccagcttccacagaggagttttagttgctgataaatgcatctaccgtgccaatttggagtcttctgcttctgtgtgcatcacaaagctaggaaatgctagtgttatatccattagatatggaagagtcatatttctcaacaacaatggattctttgcagcatattctttccactcttcttgatcaatcttgccatcaccatttttgtcagcttcattgaatgtcttatcaacaattgtttctaccatactatcggatagactcagctcagattcgttgagaagggccaataccatctcctttagctcttcacgctctatgtagccagtatgcctcaagtcatagagcttgaatgcacatgccactttctcagcttcaggagttctggggtgaaagatgctcaatgacctcacaaattctccaaaatcaatgtgcccattccgcttgacatcaaagagatcaaatatcctgtctgcaaaaaggttctgcttcttggtgctcctgaatagcgcaaatgcaagttcttccctctgaataaggccatcattgacaactgaactgctcaatttcttgtataattcatgcaacgc

>comp24693_c0_seq1 potassium channel akt1-like

cactcgaccgaggaacaacggattgacagctctccatgttgctgtttgtgaaggcaacattgaaatagtcaattttctcctggaccaaggtgctgatattgacaaaggcgatgaaaatggttggacagcaagggatcttgctgagcagcaagggcatgatgatatcaaggaactctttaattcttacaaagggaccaaaaccgaatctgctgtcacaatccctgaggagcgtcatggagttcgttttttgggaagatttaaaagtgaaccatctatcttacctgttaaccaggattcctcatttcaagtacctgatggattgtggggaagatctcgtcctaggcgtaggactaataatttctttaactctctgtttgggataatatcagcagcacaaactggagaaaacggctcgctgttatcagcagacaatgctacaggtgcagcagccactagaacttatgctgccagagtgactgtgagctgcccggagaagggagatcttgcaggaaagcttgtgttacttccgcataccttcgaa

>comp24693_c1_seq1 potassium channel akt1-like

gcattttaatttacatcgctagtcaaaagaaacgctcgtggagaacctaaaaaaactaaggaacataaattgagcatgggaatgttataaactgcattttaatttacattgccagtcaaaagaaatgctcgtggagaacctcaaaaaactaaggaacataaaagattaaggtaggaaataagtgaccactttgcagtcactggaagcaatcattaatcggtttcatagttccaattattgctttcaaaaactaaatgatcaccatctctgatcagctctatatcgtcaattgcagcaccatctttgctcaaaattttcgcaggaaagaatccatatttgtttgcaccaatttcaagtagttcttggaagctatgtggaagta

>comp24738_c0_seq1 probable protein phosphatase 2c 27-like

aacgatttatagactatgaataagatctatacatgaggaggaggagagaaatcatccttagaagatgaaaagctcaacatccaattgatacaacaacagttcactcatatacaacaggtgaaaatatccagaaatgacagagccagacgccaaccgtgggaaaaaggaaggtgcagcaatctttctcacaagttaccttccaggacacctttcagaagatttaacccttcagctgatatactcctccggactcgactcggagcaatttcaattcgggggggagggtcttgtgagaaacagataactatgactgttaagttatcgcatgtattgcgtttcagtgcttccctaaccagctctttcgagcatctttcaggatcattatgaagcatcaactctttcctagccattgtgacagcacattggctgctcatgacatcccacagcccatcgcatcccataattaagaattcatcttcttccgtcagcaaagtctcctgcaactctggctctgcgcttaaagggcaagcagagccttttgagcccttcatgtgccagtccccaatggcgcgtgatacagatagttggccattaaggtagccatcatatatg

>comp24863_c0_seq1 endoplasmin homolog

gtgactccaagctagaggcttctcatcactgaagtccttggccagagagtgatagaattttgtgtattcttcatcagtcacctcctttggattccgtagccatatggctttcacgtcattcaaaagttcccactcatgtgaagttttcttaactgtcttggtcttgggctttttctcctcgtctttctcagcatcttcttcctcttcttcctctgaagatttactttcagatgtttcctcatcatcactggactcatcctcatcagcaggaacctcctcatccacttccttgcttgcccaaaggtgtatggggaagttgatgaattcagaatacttcttcaccaactccttcaatttgtactcatccaaatactcctgcgcttcatctctaaggtgcaatctgatttcagtcccacgaccaagtggttcattccatgtatcttctgaaatcgcaaaagcgccatctgcctttgactcccatatatgctgtttatcatcattatgtttgctaatcacttcaacatagtctgccacaagatagaccgagtagaatccaacaccaaattgcccaataaggttaagatcaccacttgtctgcattttttccacaaatgctgatgttccagatttagcaatggtacccaagtgcttaatcaaatcctcctttgtcattcctatacccctatcacggatggagagtattttcttttctttgtccaatttaatctgaatctccagcttggcattgtcaccttcacccagaatttctttgtcagtgagtgaaaggaacctaatcttgtccagagcatctgaagcattcgagatcagctctctcaagaaaatatccttattgctgtaaagagagttgatgataatgtccataagccgcgacacctcagcctggaactcaaatttctccgcatttgctcggagagttttcctcgatatcgactcagcttctctcttaacgacatcagaatcagtagacaatccattcggaacggcaccgatcctctcctccaccttcggcggatctaccggcgcatccgaatcgacttctgcattcgcctgtattttcctaccttgatctgggagaagaaacagaaggcagagtaggaaaagaacggaagggatcgtccacttcctcattgttcgattagcttcaacaatttagctcagaatgtgttgcttcgaattgtgagtgagtg

>comp24863_c1_seq1 endoplasmin homolog

gcgtaaagaggcatccttccttagtaaagctctcaccaaagatttcaacttccaactgaacaaggaaaaaaatcacacataagatagattacaattctacactgcatgggcaaaaggattacgaggcaaaattcttggtagtttcttacaccaatccgacacatttctaaatcctaacgagtgggtctaatcagcatgcagcatctgaaagattttttttgagaggaagggtaaaataagaactaattcctccaacatcatcctgtctttccagatacatggacctacaactcatctttcaaatcctcttcaggaactctgtcagtcttggaagattctgtttcttgggcactgggttcagtctctggttcttccacatcatcttcctcctcgactgttgcatcaggactgatgttcaggctgtttttcactgagccatatattcgagaagcgaaatcctttgggtcggatagtgtaaagccactctccataagagctgtttgatacataagttgtgctgtttgctttacactttcatcctcagggtctttcactactctctcacgaagctccttgatgatagggtgccttgggttaacttcaagcaccctcttcccacgcatataagcctgcttgctagcatctgatagagtttgagactgcataatcctttccatgtttgcgctccaaccatattttgatgtcactactacacagggggagtcagccaaacggttgctaatcttcacatcatcaacattttcactggcaagagcacccttccaccatttagtcaactccttgaatgactccttgagttctttgtcctttgaatctttcccaattttcagcccctcttttgatacattctggaatttcttgtcctcataatccatcaggtattgcatcagatactcatctactggatcagtgaagaaaatgacctcgtaattcttctttgttagtctctcaaggaatggagatttctccaactgttccttgcttgttccagtaatataaaagatatctttctgtccagacttcatccgggagatgtattgatccagtgaggttagcttaccatctgatttagtggtttcaaaccggagaagttttgccaggcgattcctgttagttgcatcctcgataataccaagcttaattgacttgccaaattcattccagaatttagcatattgacctttcttctcattgtcatcactagattcctcaatatctttcttatctttgtcattagattcatcagggtcttcatcagcaagcttccggatcatatcaagggccttgcgaataagtttcttctttatcgttttcaagctgctatgctgttgtaacatttctcttgatacattaagtggtaaggtgtcagagtcaacaagacccttcaagaagtttaaatacttgggcagaagttcatcaaattcatctgagataaagacccgtctgacatatagtttcaaattggatttttttgagttgtagtaactctcatataaatcatgaggagccttgggaggcacaaacaatacagccttgaattcaacatcaccttcagcagtaaagtgactccaagcta

>comp24863_c1_seq2 heat shock protein 90

gctggtttctgtctcgccttcattcacatcctcatcttgaggagcttcttcagtcttggaaggagattctgtgtctttcgtgctggtttctgtctcgccttcatctacatcatcttcttcctcaactgttgcatcagggctgattttgaggctgtttttcacagagctgtagatttgcgaagcaaaattctttggatcgttgagtataaaaccactctccatgagcgccgtctgatacatgagctgtgctgtttgtttaatgctttcatcctcagggtctttcactactctctcacgaagctccttgatgatagggtgccttgggttaacttcaagcaccctcttcccacgcatataagcctgcttgctagcatctgatagagtttgagactgcataatcctttccatgtttgcgctccaaccatattttgatgtcactactacacagggggagtcagccaaacggttgctaatcttcacatcatcaacattttcactggcaagagcacccttccaccatttagtcaactccttgaatgactccttgagttctttgtcctttgaatctttcccaattttcagcccctcttttgatacattctggaatttcttgtcctcataatccatcaggtattgcatcagatactcatctactggatcagtgaagaaaatgacctcgtaattcttctttgttagtctctcaaggaatggagatttctccaactgttccttgcttgttccagtaatataaaagatatctttctgtccagacttcatccgggagatgtattgatccagtgaggttagcttaccatctgatttagtggtttcaaaccggagaagttttgccaggcgattcctgttagttgcatcctcgataataccaagcttaattgacttgccaaattcattccagaatttagcatattgacctttcttctcattgtcatcactagattcctcaatatctttcttatctttgtcattagattcatcagggtcttcatcagcaagcttccggatcatatcaagggccttgcgaataagtttcttctttatcgttttcaagctgctatgttgttgtagcatttctctcgacacattcagtggcaaggtgtcagaatcaacaagccccttcaagaagttcaaatacttgggcagaagctcatcaaattcatcagagataaagacccgtctaacatataatttcaagttggatttgtttgaattgtagtagctttcatacaaatcttgaggagccttgggaggcacaaacaatacagccttgaattcaacatcaccttcagcagtaaagtgactccaagcta

>comp24863_c1_seq3 endoplasmin homolog

gctggtttctgtctcgccttcattcacatcctcatcttgaggagcttcttcagtcttggaaggagattctgtgtctttcgtgctggtttctgtctcgccttcatctacatcatcttcttcctcaactgttgcatcagggctgattttgaggctgtttttcacagagctgtagatttgcgaagcaaaattctttggatcgttgagtataaaaccactctccatgagcgccgtctgatacatgagctgtgctgtttgtttaatgctttcatcctcagggtctttcactactctctcttggagttccttgataatagggtgtcttgggttaatttcaagcaccctcttgccacgcatgtaagcttgcttgctggcatctgacagagcttgagattgcatgatcctttccatgtttccagtccaaccatactttgatgtcactaccacacatggggagtctgccagacgattgcttatcttcacgtcatcaacgttttcactggcaagagcacctttccaccattttgtcaactccttaaatgactccttgagctccttgtcttttgaatccttctcaattttgagtccctctttcgatacattttggaatttcttgtcttcataatccatcagatattgcatcagatattcatccactgggtcagtgaagaaaataacctcataattcttctttgttagtctctcaaggaatggagatttctccaactgttccttgcttgttccagtaatgtaaaagatatctttctgtcccgacttcattctcgagatgtattgatccaatgaagttaatttaccgtttgacttggtggtctcgaatctaagaagttttgccaggcggtttctgttagttgcatcctcgacaattccaagcttaattgacttgccaaattcattccagaatttagcatattgacctttcttctcattgtcatcactagattcctcaatatctttcttatctttgtcattagattcatcagggtcttcatcagcaagcttccggatcatatcaagggccttgcgaataagtttcttctttatcgttttcaagctgctatgctgttgtaacatttctcttgatacattaagtggtaaggtgtcagagtcaacaagacccttcaagaagtttaaatacttgggcagaagttcatcaaattcatctgagataaagacccgtctgacatatagtttcaaattggatttttttgagttgtagtaactctcatataaatcatgaggagccttgggaggcacaaacaatacagccttgaattcaacatcaccttcagcagtaaagtgactccaagcta

>comp24863_c1_seq4 hsp90-like protein grp94

gcgtaaagaggcatccttccttagtaaagctctcaccaaagatttcaacttccaactgaacaaggaaaaaaatcacacataagatagattacaattctacactgcatgggcaaaaggattacgaggcaaaattcttggtagtttcttacaccaatccgacacatttctaaatcctaacgagtgggtctaatcagcatgcagcatctgaaagattttttttgagaggaagggtaaaataagaactaattcctccaacatcatcctgtctttccagatacatggacctacaactcatctttcaaatcctcttcaggaactctgtcagtcttggaagattctgtttcttgggcactgggttcagtctctggttcttccacatcatcttcctcctcgactgttgcatcaggactgatgttcaggctgtttttcactgagccatatattcgagaagcgaaatcctttgggtcggatagtgtaaagccactctccataagagctgtttgatacataagttgtgctgtttgctttacactttcatcctcagggtctttcactactctctcttggagttccttgataatagggtgtcttgggttaatttcaagcaccctcttgccacgcatgtaagcttgcttgctggcatctgacagagcttgagattgcatgatcctttccatgtttccagtccaaccatactttgatgtcactaccacacatggggagtctgccagacgattgcttatcttcacgtcatcaacgttttcactggcaagagcacctttccaccattttgtcaactccttaaatgactccttgagctccttgtcttttgaatccttctcaattttgagtccctctttcgatacattttggaatttcttgtcttcataatccatcagatattgcatcagatattcatccactgggtcagtgaagaaaataacctcataattcttctttgttagtctctcaaggaatggagatttctccaactgttccttgcttgttccagtaatgtaaaagatatctttctgtcccgacttcattctcgagatgtattgatccaatgaagttaatttaccgtttgacttggtggtctcgaatctaagaagttttgccaggcggtttctgttagttgcatcctcgacaattccaagcttaattgacttgccaaattcattccagaatttagcatattgacctttcttctcattgtcatcactagattcctcaatatctttcttatctttgtcattagattcatcagggtcttcatcagcaagcttccggatcatatcaagggccttgcgaataagtttcttctttatcgttttcaagctgctatgttgttgtagcatttctctcgacacattcagtggcaaggtgtcagaatcaacaagccccttcaagaagttcaaatacttgggcagaagctcatcaaattcatcagagataaagacccgtctaacatataatttcaagttggatttgtttgaattgtagtagctttcatacaaatcttgaggagccttgggaggcacaaacaatacagccttgaattcaacatcaccttcagcagtaaagtgactccaagcta

>comp24903_c0_seq1 elmo ced-12 family protein isoform 1

aatctgttgaaggatcagatccctgccaacccatgtctttccaaacctctgatttaagtgatgggagttgtctatcaggataagataacctccaaagttgtctaagcgcatcttgatgctctgaaaaggtgccatcaaaaggaactgaaagcctctgctttagatacttcagccttgcttcctattgtcataccaactcaagaatcaaa

>comp24903_c1_seq1 elmo ced-12 family protein isoform 1

gtataaatataagaagcagtatcacaaattgggtctttgacttgatacaaaaattttcatttcacaatcacaatgaaaactatgatatatcatcaataaactatctccgagaggatatcttaattgactgtatggtttaacttccctctcaccaattcatcttcttggaaagggatattgaagttattggatgatacctgcaagggactaagtaaaacaggtcgaaggttgctgtttgatccattttgagggctcgatataaatggtacaatccgcgcaagcaaagatccagatccaagaacaatattagctaaccactgcgcccactgcgcaattacatttgaaaatagcagtgtccaatgcagacgctctttactccgttgttcatccaaaatatccaccagcgattgcccctcagagtgcttattatcatcgtaatctctatctccaagtaatggctcattcaacgaatcgaattgcgaagtctcgtaacgatcatatcttcctccatcaacatctccatggtgaagcctccttctcaatgtccgggaactcatcaagagccagattcaccaaacagcgtcgtcaagtagaatcagacaaacggagaaaaagattgctcggcgaatttggggaaagaacctcgaataaatactaattcgcgaaccctaattaaatcaattttcttctatttttttatgaaggggtttgctctccacgacgt

>comp24903_c1_seq2 elmo ced-12 family protein isoform 1

cttcagccttgcttcctgcaagggactaagtaaaacaggtcgaaggttgctgtttgatccattttgagggctcgatataaatggtacaatccgcgcaagcaaagatccagatccaagaacaatattagctaaccactgcgcccactgcgcaattacatttgaaaatagcagtgtccaatgcagacgctctttactccgttgttcatccaaaatatccaccagcgattgcccctcagagtgcttattatcatcgtaatctctatctccaagtaatggctcattcaacgaatcgaattgcgaagtctcgtaacgatcatatcttcctccatcaacatctccatggtgaagcctccttctcaatgtccgggaactcatcaagagccagattcaccaaacagcgtcgtcaagtagaatcagacaaacggagaaaaagattgctcggcgaatttggggaaagaacctcgaataaatactaattcgcgaaccctaattaaatcaattttcttctatttttttatgaaggggtttgctctccacgacgt

>comp24903_c3_seq1 elmo ced-12 family protein isoform 1

aatctatgaataatatgattaggggtggaggcttcatatcgttggagaatttaatctactttgccaagacatatccggaagcatttcaaaacttgttgcacaagagagatggagacaggtccgaatgggagtatccattcgctgtagctggcatcaatatttcctttatgctggttcaaatgttagatcttcagtcaggtaatcctagcactctggctggacaacgctttctggaaatgctcagccaggatgatgtggctttcgacaacctcttctgtgtcgcattta

>comp24903_c4_seq1 elmo ced-12 family protein isoform 1

tgtcgcatttaagttgttggatgcgcattggcttgcaaagcgtgcttcatatatggaatttaacgatgttctcaagtctacaaggtcccaactagagcgcgagatggccctggaagatgtatcatgcgtgaaggacttgccagcttataatttactgagaagataatactgaaaacacaaccatttcttgtgagttagttcacataatgtatcacttcgcatacttgcaagaatcagcgaaaggggtgaatttctttgtatagatctaaagtattatcatctccgatcctcattgatgagtcccgtgttaaggaataattttcattagccttttttaaggtttctatagtaaatttgtaaacgatggctattggattaacactcattgtcggaaaaaaatttcataatcctcagcagatttaa

>comp24922_c0_seq1 f-box lrr-repeat max2 homolog a-like

gtgtgagtttccatcaaaggaatcacttcttccctttttctacttctacatcaagaggaatggcaatcgtttcttatgcactcaaagtgtgaatttctttactcatgtgctttccccgacttaagtagctaaatttggcaaaatataagatacaagttaaaactgtaacttaaccagcatttcctagtgaactttgtaatagcagcaacggcaaatgagaaaagaaagaaaaatggaacaggattatgaaactttaaccaactgatgccccaaaaaggataaaaagaaaagacaaaaatccaaaaccttagcattctgaagctcctgttatttcttgtgctactcagcttgtgttaatcaggaatctggcgcccattaagagcagcctcaaagcggctacatgagtcggccctcatctcagtgctcatatcattctcaggcgctgggtaatagtcttctctcagctggacatctcgtaaatgatgaattctcagaaggaacatcatgaaatgctcgtgagccgtcccatgtatgaaaagtttcctaagcctagaacattcttgcagcaatcctgctgctggaagggatagactcctctgattaacatccctgtcttgtggaggccagtaatcaagctctttcag

>comp24922_c1_seq1 f-box protein ore9-like

actccgccgccctcgtctgccgcaagtggtacatgttggagcgcgccacccgctccgccctcgccctccgcggcaaccttcgcgacctcttcatggtccccacctgcttcacctccatatcccacctcgacctctctctcctctctccatggggccaccctctcagctccgcctccgacccagccctcatcgcccacctcctccgcatcgccttcccttctctcacctctctcacgctctatgcgcgaaacccttccaccattcagctcctagcccctcagtggcctgaaatcgaacgggtaaagctcatcagatggcaccagcggccgcagatggccgccgccggcgatgagctcaagatgttgctttcggaatgcgggaagctgaagtctctcgatctctccgcgttttactgctggacggatgatgtgccgccggcccttgagtcgtatcctgcggttgcttcgaatctcacttgtcttgatatattgaacccatcattttcggagggttttaagtccgaggagattaaggtgatcaccaaagcttgtccgaatctgagggaatttcgggctgcttgtatgttcgatccgaggtatatcgggtgtgttggagatgaggcgttggtttctgtatcggggaattgtccaaaattggtgattctgcatttggctgatacatccgctctgtcgaatgcgagaggcgatccggagcacgaggggttcacgcaggaggatgccaggatcaatgtggccactttgattgaggtgttttcggggcttccattgctcgaagagttggctttagatgtttgtaataatgtcagagagagtggtccagcactagaggttctcaattcgaaatgccccaagttgagatctcttaaactggggcagtttcatgggatctcgatgcctgttgagtcgacgctggatggcgtcgcgctgtgccaagggctgaagtcgttgtcgattaggaatgtaggcgatctgacggatatgggattgattgcaatagccagaggttgttgcagattgaccaagtttgaggttcatggttgcaagaagataacggtgagggggatgaggactttggcttctttgcttcgtcggacattggttgatgtgaggatctcctgctgcaaaaatctcggtgcagcgcagtcattgagagcattggaaccgatacaggatcggattgagaggctgcatattgattgcatctgggattggacggaagagctcgaggatgatgatggaatcagatgtggcttcgacctaaatagtttggatcaaggtgaggtgtcgtaccagcccgttgagtttgctgcaaaacacttcagaagcattgagtatgattatgattatgatggcatgagtaatgcaagcaagaagtgtaaatactcttatgatctgaatgcttcttatgtgggattagatgtcaatagcaatgggtatgaaactggcaatggacatggtgtgaagacatgggacagactgcattatctttctctatggattgccgtagggcagcttttgacaccgctagcatctgcaggacttgagaactgtccgaatctggaggagatccggatcaagattgaaggagattgtagggaattgtcgaagccctctgagcgcgagtttgggttgaggatcctgctgaactatcctaagctgtcgaagatgcatttggactgtggcgacaccattggatatgcccacacagcaccttctggacagatggacttgagcctctggg

>comp24947_c1_seq1 ubiquitin-like modifier-activating enzyme atg7-like

ttgacaaacaaagcctactcaaagcagaagcaaaaaagatctggcaggacatttgtttgggcaaagttgaggaggactgcagtgttcttttaagattccttgtcatttcatttgccgacttgaaaaaatggagctttcattactggtttgctttccctgctctgattcttgaacctccagcaactgtaactaatcttaagccggctgctcagtggtatagtgtggaagaggcggagtcggtaacagctgcttgtaatgattggcgtaacactcgctcaacaacagacgttcctttcttcctggtatccatttcttcaaattctgctgtgactattaggcatctggctgagtttgaagcctgtcaacgggatggccataaggtcctgtttggattttatgacccttgtcatcttccaaataatcctggttggcctcttcgaaatttgttatggtttatttgcagaagatggagtcttcagaaggttcagtttttctgctatcgtgaaagtcgtggttttgctgatctggaattatctttggtgggtgaagcatcaataacaaattcacaagagttaaaaaatcatcagaatatgccgaatgctgttggttgggaacataataacagaggaagaaagacatcaaggtgtatcagtcttgccaagacaatggatccaactaggttagccattgaagctgcagatttgaacttgaagctaatgagatggcgtcaactgccgtcattaaatctgaatgtcttgtcatctacaagatgtcttcttctaggtgcaggtacacttggatgccaagtcgctcggatgcttatggcatggggtgttcggaaaattacacttctcgatagtggcagagtctccatgtctaatccactgcggcagtccctgtataccttggatgattgcgtgaatggtggtgaatttaaggccatagcagctgtcaaaagtctaagtagaatattcccagcagtggaagcacaatctgttataatggctatcccaatgcccgggcatcctataccgagccaagaagagaatagggtgcttgaggactgcaaacgcttgcatgatttggtagattctcatgatgcaatttttttgctaactgatacacgagaaagcagatggcttccaactcttctttgtgccaacaccaacaaga

>comp24947_c2_seq1 ubiquitin-like modifier-activating enzyme atg7-like

gctgctctagggtttgatagctttcttgttatgcgccatggagctggtcctttaagctcttctcatgaaataaaagcagaagctgtgaatgatttatctgccgacacaggaactgtctctctcatgaacaacactggggggcagagagtgggctgttacttttgcaatgatgttgttgctccagttgactcaaccaccaatcgtacattagaccagcaatgcacagttacgcgtccaggtctcgctcctattgcatcatctcttgcagttgagcttcttgtaggaattatgcatcatcctttggggatttcagctaaagctgaatttgctagctcgattgatactggcggtgagcaacctcttggcatcctaccccatcaaattcgaggctccctctcacagttttcgcagatgagcctcatgggtcatgcctcaacaagttgcacagcttgtagctcaactgtcgtctcagaatatcagaagagggggttggattttatacttcaagccatcaatcatcctacttatctggaggatcta

>comp24947_c3_seq1 ubiquitin-like modifier-activating enzyme atg7-like

agcctggaatccctccaacgtgtttgtattgtagagaataccagggacaggacatctattcctgtttccacgactcatttccggcataaatgattgttcattagattcagaaggtaaggattccgcaagaagagttaaatgatttgatacttgattatgtgagcaaggggcatagaaacctgtgataggaataggagactcatcaatccccagcttgttgagcttcagtgacgataacctgtgccaaaaaccctcgtccactgcgctttgaaaaggcgcaaattgaagaatcgattctatcccttgatccaccattatagacaacacaaaaaccctctacttctcgttcaattgatgcgttgatagatccaatgcaagaaaattacagagttcagagagattagcaccccgtcgctaaacagatcggaattgtcatgacgaactgagattgaaattgggaaaaccggtcttaaatgctgattatggatgatcggcaaaaaattaacggagcaccggcgatacagttgaagaaatttagggcacaaaatttgggattgaggtaag

>comp24960_c5_seq1 e3 ubiquitin-protein ligase bre1-like 1-like

gataaacctccagctcaggaaatcaaatccaacatatcatctttggctagatggcattcaaagcctcgtgcatgttttgtaatatccagtgtgcaattagaacgtgattccaagtgaccaaccagctcttcccatgaattattgaccacagccaaggttttctcgtatgactgttgtttagcattcaactcatacaatctgtcctcaagagtgttaatctcaaccttctgagtttccaacttttgtaagagtttttgattttgaaattgaagtactgctgcatcaagctttttctcctcagacaagggcgcaagaggctgtttcttggccgtagcggccgtgggcgatatggagctaacatgacgccgtttcttgtcagcttcacctgtactacccattagatacccaccccctcaaaaccct

>comp25058_c0_seq1 serine threonine-protein phosphatase 5-like

ggccttgacgaagagttcaaagcatattctataccatctctacagacaataagcacccacattgtgccatggacttgacttgctaaccgtaaactcttatcttaaaagatggacagcaattgatgactgtcggtattacagatgccagctggtattacatcaggagtaggtcatttcgagattgatgaaccattacaccaaatcttcaactaatattctatcagatattggctcataaagcaagcaatccaaaagtagcaacaagaaaaataatctaggagacaaaccagaaatggcatttcagcctatgatatcaaacaaaacaaattcaaattgcaaaagcagaagtaacacaagactagaatctgtttcctccacgactaaagccttcatgaagtgcagccactcccaatggatccagaaatcttacctagtccaaggataagcacctgaatttggatgcttcttggacatgattttgtaaacccaagctagatccttgcaacagagtcaacactacttctcaaaatgtctattccacttccaactgcaagctccgcaaataagacacctgtgtcctctgatgaagcttcagccacaattaagaacatgcattgtccatatatctattcctgcttgaatagacgagggagaatttcatgcactccaatgtctaatgcaactggatccctgcacaaggttttgcccttctcactagaactaggaataaa

>comp25058_c1_seq1 tetratricopeptide repeat -like superfamily protein isoform 2

aacatgtcctcagcaacaggacctagcaaatacgcatcgtggaaagcttacaaatgagaatttggagtttgaatcctaattaaaaactacagactataccccaagaaaaagttaagaaaagataaattataaagggatcagaatctcagaaaccctaataaaaaacccagaacaaattaccgaaattggaaaaataatagtgttaatcgctgttgaatcttgagactgttaatcgctgttgaatctcgagaccaaagctaacaaaatgggagaatcagagggaaaagcagcagagatttcactgaaagatcagggcaatgagtttttcaaagcagggaattatctcaaagcggcagctctctacactcaggccattaagcaagactcctccaatcccactctttacagcaatcgtgctgctgcatttctgcatttggtaaaacttaacaaagctctagctgatgcagaaacaactatcagtttgaaaccagattgggaaaagggttatttcaggaagggatgtgtattagaagctatggaacgatatgaagatgccttagctgctttccaggttgccttgaagtacaacacacaaagttctgaggtgtccaaaaaaatcaagaggcttacgcaattggcgaaagataaaaagcgagcacaagaagtggaaactatgagatcaaatgttgatatggcaaaacatttggattccctgagaactgaactatctggggagtatggagctgaagctgaagactgttggaaggagattttctctttccttgttcaaacaatggagaatgctgtaaaatcatggcatgaaacttcaactgtggatcctagagtttatttcctgcttcaccaggataaaactgacactgaaaaatatgctccagttgttaatattgataaggcattcgaatcaccgcacacgcacagcagttgtttttcattcctcaggcagtatgctgaagattctttctctcgagctgcttgcttagtggcacccaaaagtataatatcctatcctcaggtttggaaaggccaaggatcaagaaaatggaagcatgggcagaatgatggtttctttgtccagtttgaatcaccttcactgcgcaaaatctggtttattcctagttcta

>comp25181_c0_seq1 dnaj homolog subfamily c grv2-like isoform x2

acttgatctggaacgagcgtactaggcaggagttaatggaggctttgcaggctgaggttcacaaattagaccttgagaaggaacgtactgaagatattgttcctgggggtacatctaaagaaactatgagtgggcaagaaaccatgcctcaaatatcatggaactatgcagaattttctgttcgttaccctagcttggctaaagaagtttgtgttggtcaatattatcttcggctgctgcttgatagtggaactggtggtagagcacaggactttccactgcgtgaccctgttgcattcttcagagcactttatcatcggttcctttgtgatgcagacactgggctgactgtagatggtgccattcctgacgaaatgggtccatcagatgattggtgtgatttgggaagattagatggttttggaggagggggaggttcctcagtcagggagctttgcgcaagggctatggcaattgtatatgagcaacattacaactcagtagggccatttgaaggcactgcacatattacagtgcttttggacagaacaaatgatagagctctaagacatcgccttcttttccttttgaaggttttgatgaaagttctgtcaaatgtggaggcttgtgtcttggttggagggtgcgtccttgccgttgatttactaacagttgttcatgaagcttcagaaagaacagctataccattgcagtctaacttgattgctgctactgctttcatggaacccttaaaagaatggaagttcatagacaagaacaattcagaagttgggccagtggagaaggatgcaattagaagattttggtccaaaaaagaaattgactggacaaccagatgctgggcatctgggatgccagattggaagagactaagagacatccgtgaactccgatgggcaatggctgttcgagttcctgttcttactccagtccagataggagaggtggcattgtccatactacacagcatggtagctgctcattcagatattgatgatgcaggagag

>comp25181_c6_seq1 dnaj homolog subfamily c grv2-like isoform x2

tgaaatttgtggagcagcagcatgcaagcctcagtcctgatggttcatatgatgttaaagattcacactcctttgtttatgaagcactgtctaaggaattgtatattggcaatgtgtacttgagagtctataatgatcaaccagattttgaaattactgaacctgagaagttctgccttgctcttgttgattttatatcccatctagtgcataatgctccatcctcgaaggttgatattcatgttaatggtgatgtaactactgaatcatctgtggagcagctctctgctgatgattcttcagcaactgttgatgggaaaatcatggacagagaggaatttgagttcattaagaatcttcagtatggtttgatatctcttcagcacttgttgacaaggaatcccaatttggcttctgtggtgtccacaaaggaaaagttactgcccctatttgagtgcttttctcttccggttgcttcagcaagcaatattcctcagctatgtttagctgtgttatcgcacttgaccacatatgccccttgcctggaggcaatggttgcggatacctctagtttgcttattttactacagatgcttcactcatctcccagctgtcgtgaaggagctctgcatgttctctatgccttagcaagcacaccagaacttgcatgggcagctgctaagcatggtggggttgttttcattcttgaagttctcttgccaatacaagaagagattcctttgcagcaaagagcagcagctgcttcactactagggaagcttgttgggcagacgatgcacggaccaagagtggcgataactctggcaagatttctgcctgatggccttgtctctataatcagggatggtcctggtgaagctgttgtcagtgctcttgaacaaacaactgaaactccagagcttgtatggactccagcaatggcagcatcactgtctgcacaaattgtcacaatggcttcagatctttatcgtgaacaggtaaaaggacatgttgttgattgggatgcccctgaacaggcctctggccaacaggaaatga

>comp25292_c0_seq1 acetylornithine mitochondrial-like

ggcaagagtcattatatctggatatacgccaaaagcttcatgagcccagagataaccagttcggcccaagccacattgtacctcatcaaatacaagaagacaaccagcattatcacaggcagcacgcagggattgcaaaaattcctttgttgcactgtatataccaccttccccttgaattggttccacaaatactgcagcaatctttccactttgaattaattctactgttgtttttgtattaccatattctaagaacgtgactccaggcataacaggttcaaatggtgatctgtaatgctctttgctcgttaatgcaagagcacccattgttctaccatgaaaacaatttgagaaggcaatgaactccacaggagggtccttttcatcgggatgtgaaaatctttggaacttccttgaaaactttattgcagcttcattagcctcagttccagaattgcaaaagaatacacgatcagcaaaagagctggcaaccagaagttttgcaagctccacctgtggaatagagtagtagatattgctgacatgagtaagcataccagcttgttgtgtaatggcacgcaaccaatctgaatccccatggccgagggcattcactgcgataccagaactcaagtcgagatactctcgtccttcaacatcgtacaatttacaccctttgccactcgacaacaccaccggcgccctcgcgtacgtccctactataaatttcttctcgtccgcgatgatttccgagcttctcttctcgtggagagacggattctgattggaatctggcgtacgcacgtccacgttgaggcacgctctgggcgcaacggagaacttggtgggtttgagggtttgagagcagtggagggcgcatgagtgagaattgttgagaggaagaagcgctgtccagctcatttttgccggcagaaattcgtgaatttgtccttcgatttgatgaacggaattgaggaggggtggcggcgccgccggggagtggtggtaggagga

>comp25292_c1_seq1 acetylornithine mitochondrial-like

atattactcaggctaaggactcaagtatgcagaactgagctaagacagattagtgcaacataaacatgcaaacacttaattagtcagggcaaagttcaacaaatcctctttgaagaggcaatgacagctaattgttggtagcccgatcaagtacaggaaaacaattcttcaagatctcagcagcttgatcaagttcctgctttgagatgatcaacggagggacaatccgaacgacatttcctttcccggcagttaaaaggagaaggccggattgttggcacgcatccactaaggtggaggccgatacatccagctctataccgatgatgaggccaaatcctcgtatttctttcacgtgagagtttcctgctaactttgctgctaatacatctttgaaatactgacctttctcccttacattggccaaaaagcttggattcgatattttatcaagtacagcaattccagcactgcaaacgagagggttaccggcaaatgtactcccatgatctccgaaattaatggcagcagcaaccctttcagtcaccaatacagcaccaatggggaggcctccag

>comp25297_c0_seq1 arm-repeat tetratricopeptide repeat-like protein

caataaaagattacctaaaagagataccttttcttgatgatgaagagcttattctggtcgttagcgagctgtggaacgttgcaatgactcgaccggaaggtgaagaactcccgtctctagggatctttgaatgcatgacaagtttgatcaacaaagctatccatgatagagcttggctgctcaaacatcaaaacatttacattccctattatgcagctcatattgtgggctcgtatacgatgcacaaagttgatttcgcagtgagggctgtcgattctggtgtcatatcaccactattggagctcttgagagggaagatgagttgggtggagcaaagggttgccattagagcacttggccatcttgcaagctatgaaaagacctttaaggccatcgcagtttatgaagatgaagtggtgaaattagccatgcatttggcctcatcctgtttggaagaggtgtatagagcgtttgtgggagtggacaggagctcaaagagattgaagtaccattgcgacttgcttacaagaggcgtcggaggggtagagatggag

>comp25347_c1_seq1 peptidyl-prolyl cis-trans isomerase fkbp16- chloroplastic-like

gttgctcaattttctgacagatatgatattccaggtattatgatctgaaggttggcgggggacctgaagctgtaaagggatctcgggttgcagtccattatgttgccaagtggaaaggcatcacatttatgaccagtagacaaggccttggtgttggtggtggaacgccttatggatttgacgttggtcagtctgatagaggaacagttcttaaaggattagacttaggagttcaaggcatgcgggttggaggccagcggttgctgatagtgcctcctgaactagcttatggaaataaaggtgtacaggaaattcctcctaatgcaacaattgagctggatgttgaattactatccatcaaggaaagcccatttgggtcccctgttaaaattgttgaaggataattacttaagatttcactatgagtggatttggttcatggagggcaaatgtacaaggtcctgttaatacttggtgacagcttcatgaaggacatagttgcattgaatttgttaatgaactggacaagatgtctccatatgaacatgacatgctattcattcaatataattttctgcagcattttgataat

>comp25347_c1_seq2 peptidyl-prolyl cis-trans isomerase fkbp16- chloroplastic-like

gttgctcaattttctgacagatatgatattccaggtattatgatctgaaggttggcgggggacctgaagctgtaaagggatctcgggttgcagtatggctgtggcacagttgaaatttttaatatctaagtacatggattttaatacttttaaaatgtggattcatgcaaattcttctaacctgcaagagtcctgtggagttatttattacagagctgtgcaatctacacttttttcgaggatttgcatttgatgagtccacacctctgatgttagttattagcatgttcaaaacttaagtaatagaatcagcatcttctgacattattccctcaggtttttagctttcatttccacatgaaaggtttagtaacagaaataagaatgtttgtttcaaaaattttagttaatcatgtttatcaggaagatgatccgatcagtagcatatgccgaagtaaattctgaagctggattgatgtcaatgttcttgaggttttacttgaatgcataactgcgcaatcggatttctttaataaggtgaatataggggtttccagaaccatatagaatatctccatttagaagtcttttcaagagttgagatctcaattcaatcacaaaagaagggttgaccaaaaatcaacatgacattccccataattaagtggtcatgatgcagtttcattatgttctccaaaaatatatttgtttttcataatctgttgcttttctaatagaaattgtatcataccattgcttgcttgcaatttgtcaggtccattatgttgccaagtggaaaggcatcacatttatgaccagtagacaaggccttggtgttggtggtggaacgccttatggatttgacgttggtcagtctgatagaggaacagttcttaaaggattagacttaggagttcaaggcatgcgggttggaggccagcggttgctgatagtgcctcctgaactagcttatggaaataaaggtgtacaggaaattcctcctaatgcaacaattgagctggatgttgaattactatccatcaaggaaagcccatttgggtcccctgttaaaattgttgaaggataattacttaagatttcactatgagtggatttggttcatggagggcaaatgtacaaggtcctgttaatacttggtgacagcttcatgaaggacatagttgcattgaatttgttaatgaactggacaagatgtctccatatgaacatgacatgctattcattcaatataattttctgcagcattttgataat

>comp25347_c1_seq3 peptidyl-prolyl cis-trans isomerase fkbp16- chloroplastic-like

gttgctcaattttctgacagatatgatattccaggtattatgatctgaaggttggcgggggacctgaagctgtaaagggatctcgggttgcagtatggctgtggcacagttgaaatttttaatatctaagtacatggattttaatacttttaaaatgtggattcatgcaaattcttctaacctgcaagagtcctgtggagttatttattacagagctgtgcaatctacacttttttcgaggatttgcatttgatgagtccacacctctgatgttagttattagcatgttcaaaacttaagtaatagaatcagcatcttctgacattattccctcaggtttttagctttcatttccacatgaaaggtttagtaacagaaataagaatgtttgtttcaaaaattttagttaatcatgtttatcaggaagatgatccgatcagtagcatatgccgaagtaaattctgaagctggattgatgtcaatgttcttgaggttttacttgaatgcataactgcgcaatcggatttctttaataaggtccattatgttgccaagtggaaaggcatcacatttatgaccagtagacaaggccttggtgttggtggtggaacgccttatggatttgacgttggtcagtctgatagaggaacagttcttaaaggattagacttaggagttcaaggcatgcgggttggaggccagcggttgctgatagtgcctcctgaactagcttatggaaataaaggtgtacaggaaattcctcctaatgcaacaattgagctggatgttgaattactatccatcaaggaaagcccatttgggtcccctgttaaaattgttgaaggataattacttaagatttcactatgagtggatttggttcatggagggcaaatgtacaaggtcctgttaatacttggtgacagcttcatgaaggacatagttgcattgaatttgttaatgaactggacaagatgtctccatatgaacatgacatgctattcattcaatataattttctgcagcattttgataat

>comp25440_c0_seq1 integrin-linked protein kinase family protein isoform 1

catgaacataaacctgaagctattgtccaccgtgatcttgaaccttcgtaagtttttagtcttccaacttctttgtgtatttcttttatgatctataagtttctcaaagcatttggtcttatgtctgagtaagataacagaaatatcttgctggacgactctggacacctgaaagttgcagactttggagttagcaagcttcttcaagtcgccaacagaatcaaagaagacacacctctttcttgtcaagatacttcttgtcgttatgtggctcctgaggttttcagaaatgaagagtatgacactaaagtggatgttttctcatttgcattaattttacaagagatgattgaaggatgcccaccattcaattcaaggcaagactatgaagtgccgaaatctcttgctggaaaagagcgccctcctttcagagcccctttgaagttttatgcccatggattaaaacagttgatcgaggagtgttggagtgacaagccagctaatagaccaatatttaagcaaattatcccacggctggaggtgatctacaacagatttggccacagaaggcgttggaaggtgatgaggccattgaagtgcttccagaatttggactggacgaaagatggttctagtgtaggtagccgaacacattcaaatcgctccacctgaaactgcagaaaggtgctcttttcagctgtcattttccggaaattgttatttgagataaatcatcttgttaggacgtgtgcggtagtgatattgataaggcagatatttttgtgctgttttatgaagttgtcaatgttatataaattattgcaacagtacaccagttacaaagtatgctcatggaccatgagaatattagtttgtaagacaatgtcagttaccagcagttgatttctctttttcttgtttctttttctgttgaggaagaaggttggaggggttagaaatttttttttgattaaccaatgtaataacatgaatttggatattctattgggataagatactttatttcgtgtttcattgtatta

>comp25440_c0_seq2 probable serine threonine-protein kinase drkd-like

ggggtttaatgtgccatggtgtttccccattagatggctccaatgcatgttaaaaatgctcgtgaggtgcctgaatatgagattgatcccagagaacttgattttaccaacagtgttgaaataacaaagggaacctatagaatagcttcatggcgtggaacacaagttgctgttaaaacattttgggaaggaattgctgttgaggataaagtgaagtcatttagagatgagctttccttgcttcagaaagtaagacatccaaatgttgtgcaattccttggtgctgtaacacaaagcagtcctatgatgattgtgacagaatatttaccaaagggtgaccttcatgaatatttgaaaagaaaaggtgcacttaaacctgcaacagctctcagatttgcaatggatattgccaggggaatgaactatctacacgaacataaacctgaagcaattattcaccgtgatcttgaaccttcaaatatcttgctggacgactctggacacctgaaagttgcagactttggagttagcaagcttcttcaagtcgccaacagaatcaaagaagacacacctctttcttgtcaagatacttcttgtcgttatgtggctcctgaggttttcagaaatgaagagtatgacactaaagtggatgttttctcatttgcattaattttacaagagatgattgaaggatgcccaccattcaattcaaggcaagactatgaagtgccgaaatctcttgctggaaaagagcgccctcctttcagagcccctttgaagttttatgcccatggattaaaacagttgatcgaggagtgttggagtgacaagccagctaatagaccaatatttaagcaaattatcccacggctggaggtgatctacaacagatttggccacagaaggcgttggaaggtgatgaggccattgaagtgcttccagaatttggactggacgaaagatggttctagtgtaggtagccgaacacattcaaatcgctccacctgaaactgcagaaaggtgctcttttcagctgtcattttccggaaattgttatttgagataaatcatcttgttaggacgtgtgcggtagtgatattgataaggcagatatttttgtgctgttttatgaagttgtcaatgttatataaattattgcaacagtacaccagttacaaagtatgctcatggaccatgagaatattagtttgtaagacaatgtcagttaccagcagttgatttctctttttcttgtttctttttctgttgaggaagaaggttggaggggttagaaatttttttttgattaaccaatgtaataacatgaatttggatattctattgggataagatactttatttcgtgtttcattgtatta

>comp25440_c0_seq3 integrin-linked protein kinase family protein isoform 1

gtgtgtgtgtgtgtgtgtgtatatctgttcatatatagaaacgcgcactagaatttcttttcaaaaccaataaaaaatttccatgttaagttcttgcctgcttttttcgccatccgcaatctagctcaggatttggtcgtggatttcccgtgattttcatcttatagcttttctggaaatatgatggtttttaattcgttctagttgttgatttgctttgtaatttttagggtttagactctattgtttgcttagattaggattgagttttcggattcttgattatttctgggttttgattttttttcgggtcagttaagcttttgatttttccttagccgcagttgactttgtttccattttgacattttatagtcttccttgttaaaccctctattttcttggagaaaaaaggaattatcaagaatatgagttgttgatttgtgaaagttgcttcaagaactcgtgatttcttgattaaaaaattaatcttggtttagaaaatctcttcaaatcttgtcgaagttcttgataatattttcaatggaatccgaaaccccggtgaaatttactctctggaagcaatcttccatggcgccggagagggactatgatgaccttgatgatctggagggagaacatgaagatgacgatgtgattgacattgatccggggttgaagttgctgtatttggtgaatgggggtgacttggatggcatcaaggagcttttagcctccggtactgatgttaatttcggcgatatcgatcaccggactgcgcttcatattgccgcatgccaggggtacgaggatgttgcacggttgctggttgaaaatggggcaaagctagacgccaaagatcggtggggaagcacgcctcttgcagatgccatacattataaaaatcatggtgtaatcaaactattggagaaacatggcgcaaaggattttatggctccaatgcatgttaaaaatgctcgtgaggtgcctgaatatgagattgatcccagagaacttgattttaccaacagtgttgaaataacaaagggaacctatagaatagcttcatggcgtggaacacaagttgctgttaaaacattttgggaaggaattgctgttgaggataaagtgaagtcatttagagatgagctttccttgcttcagaaagtaagacatccaaatgttgtgcaattccttggtgctgtaacacaaagcagtcctatgatgattgtgacagaatatttaccaaagggtgaccttcatgaatatttgaaaagaaaaggtgcacttaaacctgcaacagctctcagatttgcaatggatattgccaggggaatgaactatctacacgaacataaacctgaagcaattattcaccgtgatcttgaaccttcaaatatcttgctggacgactctggacacctgaaagttgcagactttggagttagcaagcttcttcaagtcgccaacagaatcaaagaagacacacctctttcttgtcaagatacttcttgtcgttatgtggctcctgaggttttcagaaatgaagagtatgacactaaagtggatgttttctcatttgcattaattttacaagagatgattgaaggatgcccaccattcaattcaaggcaagactatgaagtgccgaaatctcttgctggaaaagagcgccctcctttcagagcccctttgaagttttatgcccatggattaaaacagttgatcgaggagtgttggagtgacaagccagctaatagaccaatatttaagcaaattatcccacggctggaggtgatctacaacagatttggccacagaaggcgttggaaggtgatgaggccattgaagtgcttccagaatttggactggacgaaagatggttctagtgtaggtagccgaacacattcaaatcgctccacctgaaactgcagaaaggtgctcttttcagctgtcattttccggaaattgttatttgagataaatcatcttgttaggacgtgtgcggtagtgatattgataaggcagatatttttgtgctgttttatgaagttgtcaatgttatataaattattgcaacagtacaccagttacaaagtatgctcatggaccatgagaatattagtttgtaagacaatgtcagttaccagcagttgatttctctttttcttgtttctttttctgttgaggaagaaggttggaggggttagaaatttttttttgattaaccaatgtaataacatgaatttggatattctattgggataagatactttatttcgtgtttcattgtatta

>comp25461_c2_seq1 pyrophosphate-energized vacuolar membrane proton

accagtgttggatgcggagatagcaatctgaacaccagatacaagagctccagcaaggacaccagaaagagtttccacaccgaagaaaatgcccacaatgaggggtgtgagcataacaagtgcacctggaggaatcatctccttgatagatgcatctgtagaaatcttaacacatgttgcatagtccggctttgtagtgccttccatgagaccagggatggtgttgaactgccgacgcacttcttcaaccatcttcaga

>comp25461_c5_seq1 pyrophosphate-energized vacuolar membrane proton pump-like

tacatattattactgagagagcgagagacagttaaatatttctcccttccaccaaccattcaatttttgccacccaaatattcctccaaacccccaaacacacacagcacacactcacagacacacgtaaaagaagacagaacacaaacccctctcccccatacagtttctttccttagccaaatttctcctgatatggccgagacgactctgcttccagatcttggcaccgaaattctcatccctatttgcgccgtcgtcggcatagcttttgcccttgtgcaatggctgttggtgtcgaaggtgaagctgtcggaatcagtcgtcgccgacgccaaaaatgatttcagggaagccttcctcgaggaagaagaaggcatcaatagtcatgacgtagttcggaagtgcgccgacatccagtccgccatttccgaaggtgcaacatcatttcttttcacggagtaccgctatgttggtatcttcatgcttgcttttgccatattaatatttctgttcctcgggtccgtggaaggatttagcacaaagaatcaaccttgtacgtatgacagcgccaaattgtgcaagcctgctcttg

>comp25497_c1_seq1 poly(adp-ribose) glycohydrolase 1-like

atggaaacaggccttagattattggaatcacaacagtcaggcattgtatttctcagtcaggaacataaaataaggtgcattgttcactattttgagaggatatgcttggatatgcctatggggaatgtctcctttgagcgaaaagttcttcctttgaagaaaagtctatcttacattgtttatccagagcctgatttctggagcaagtctaacatttctctctgtcagtttgaggtccgcacttctggtttaattgaagatcagttatccgaagctcttgaagtagattttgcaaataaatatattggaggtggtgctctcagccggggttgtgtacaagaagaaatccgtttcatgatcaatccagaattgattatcagcatgcttttcttgccttcaatggcagat

>comp25524_c1_seq1 probable dolichyl pyrophosphate glc1man9 c2 alpha- -glucosyltransferase-like

gtcctgttgaccttgaaagtcgaaacaacgtcctcggatagacctgtcaagacatctgttactgataaagaaagaactcattgttgttgagatgaagttactgcaaagaataggcttcaagcatccctcatacttcgggtaaagatcaaagaaagtacaaaacaaatacaacaacaaaatcttctcatttgttagttatgtgtgcttagatgatcccagctgcaagccaagcaattaatctataaatcagtgagtcagagcaatctgtcttagttgccagatccacgagtataccattccaagagcacaatatatagaaatcatcaaaaggggtagaaaaggaagccgatcaccaaaaatgataggatgcagaaactgtccccataactcaactacaaacaagccgaacaggtaggactttccaaaccaaccaataccaaaggcgggagtttccaacttattatctcctcccttaaccgcagtttctgttgtagcagttgtcccaggaaaacgcgaggaaaacccaaaaaacatcagaacagcatgcagaagcagaaatacaactttaattggatattcttgggcttcaaacagaagagggaaaagtgagtagcaagacactatagacaagaagaagtaatgcttagcatcctctaagcttttcagggatgtcagtgcaaggggaataacaaagtgaagtgatgctttttcatggacatgccatccaaacagaaagccacatgtataagcataggatacccatctattgaccatccttggttggggattcctccaagcctttactagacaaggagacagtgccaagaggacggtgatgaatgttatcaaaggggtgatcgtgggtaatacagcaaaaggtgaggaatcccccactagaccaccagtgaatgaagcttttggtgattggatattgaaccctagtttcacaagcaaaaatcctagaattttatccaacatta

>comp25545_c0_seq1 serine acetyltransferase

ggcttagactgaatacttctccatcttcacttctatataaatgaagcaatacacatgaatccacaacagctaaaaagctaatctttttttgtaatttttttagtcgttttttttctgattttgcatttgaaagcaatggcagcttgtgtcactgattcaacaacaaatgagatacatgtaagagctaactcttgctgctgccagagtgctaattctattagcaggttaattagactctgcaaaccgaactctttggttctatccactaaaagtttcaagaattcttgcaaagttagtcctcaagaatacagtgatgagatatggctgaaaataaggcaggaagcagagtctggaataatcgaagagcctatcttatcaaagttctatcattctgcaatactgtctcatacttcactagagagcgcgttgtcgaatcacctagctataaagttgtgcaacgcgaatctatcaagggacacccttaatgctgttttcttgaaggccttagttgaagatggtgaaatccagcacgccgttagagatgatctcaaggctgtgaaggagcgtgatcctgcctgcgtaagttatatccattgctttctgaatttcaagggcttcctagcatgccaagcccatagaattgcacatagcttctggctccagggcagaactgcgctggcgctgcttattcaaaatcgagtttcagaagtttttgcagtcgatatccatccaggagcaaggatagggagtggaatagtgtttgatcatgcgactggaattgtcatcggagagacagcagttgtcggaaacgacgtaacaattttgcataacgtgacgttaggaggaacagggaaggttcagggggacaggcatcccaagattggaaatggagttatgataggtgctggggctaaagttctgggaaatattagagtcggagaaaatgcgaaaatcggtgcaggatcggttgttttgaagcaaattccggcaggggccacagccgtcggaaatccttccagattagtgggatcaagatgagctcgagaacaagacagggagtcttaggcctttgttattgactcattcttgaaattcttattttatatttcggcctgggaaatttctagttatgctccctgccttggcttctttgttttgctatgctcagcttctgtatctacattttaccttgtattatggattagaagttggagccttatttctgtgtgaatctgttccactaccattttgtgttcttaatgtagaaccg

>comp25605_c3_seq1 lrr receptor-like serine threonine-protein kinase rpk2-like

ttccagtaagcttgtttccaccggcaagaaatgcataatctgtttgcttgcctaatcttgcggatgcaacaggcatcgactgcaggggaccacttaggttgttagaaccaaaattatgcattaccaagaaacttccaccttctccataaagaagcaaagaggtttcaatctgtgttctatatccaaaatatgctaaataagcagatgaa

>comp25652_c3_seq1 chaperone -domain superfamily protein isoform 2

aatctttctccaaattccataattcgcagcttatggacgccgatcacagcaccacttataaggattactacaagatcttagaagtggattatgatgcaactgatgagaagatacgattgaattatcgaaagctcgcattgaagtggcatcctgacaaacataagggtgatactgcggttactgcaaaatttcaagagattaatgaagcttactctgtgttgggtgatccagataagagacttgagta

>comp25674_c2_seq1 thioredoxin-like protein chloroplastic-like

atcttccaaactggataataattttatcattccctctgttttctctctgcatctgggatctcaacactcattcctcagagccaatcagacaactcattgttttgcatcatttttttgacttctttcatggctacattcacaaatttcttagccaaaaccccatatctcactcccgccaaaaaaatcgctcctcttgctcctcaattttcctctattcgcctcccaatttcatccaaacccaaacaaaatgacttctacaaattagatgcaaatcgtcggagcttcgtcgcaaaagcatcggctgctgcaggcgtcgaaaaagtaaacagggatgaaagagtacagaaaatccacagcaccgaagaattcgacgaagcgctacgcgccgccaaaaaccgcctcgtggtggttgaatacgccgcccgcgacagcataaacagcagccagatctaccccttcatggtggacctcagccgcagctgcagcgacgtcgactttttactcgtcatgggcgacgaatcagaagaaactcgcaaactatgcgaacgcgagaaaatagaacaagtgcctcacttcagcttctacaagggcatggagaaaatccacgaggaggaaggaattggccccgatcagctcgtcggtgacgttttgtactacggcgacagccactccgccgtcgtgcagctgcactccagagaagacgtcgagaagttgatcgaggaccacaaagctgatcacaaattgatcgttttggacgt

>comp25687_c1_seq2 heat shock factor

aacaacaaaactatggcctgtatgactccaggagacaatagaattagtcaatggatcatctaccatctcatatgtcttcaggagaaacggcgacggcgaattagaaccaccattcgatccatccatcactaatgatcaatctataacaaggcacatataaaatgacttcagcagaccaaaagtcctttatgaatcttcccttgatatcaactatatgaatgttctgtttccttcaaaaatccttcatttttccaacatttaaccttcaaattccagacccttagcaagaacaagaatcatcaatcagaaaaaaccgaaaacccacatataattcagaaggaaaaacacgatccttacaatccaaccccaaaaaccgatcttccgtcattaacaaaacagagaaaaacaaatgaactatcgcagtacaacccaacaaagctccaaatacacataatcaaccgactctaatctgcaacaacattacaaagaaaaaagaactaaaaacccaatatatagatcttcacaaacgcttttcattttcccacatgtttct

>comp25730_c0_seq1 cation h+ exchanger 20 isoform 1

cgccgtatggagttcaagtcaagctcgaggccaactaggaaaaggaagaagagcagtccaatgcttgctacagattcaagaattggagtactccattttggaaaaatcctatgcatgtattcttggttccgaccaaaagccgacggcccgagtaaaatcccgccaaccatctctgcaatgactttgggttggcgtagaggtttgaagagaaaagcgagcaatcggcagagtacgaggattagagttgtctgaataatcagcaatggaaaagcgtaatctaagggattgtcaccttgccaaattccatttgatgatgtcttaattgaagttatattcatcgccatatttggatgaaacaagaaa

>comp25730_c0_seq2 cation h+ exchanger 20 isoform 1

tttcggtctaaaatgaagcaaaaagttgattaataaagaacgtacaaccatctctgcaatgactttgggttggcgtagaggtttgaagagaaaagcgagcaatcggcagagtacgaggattagagttgtctgaataatcagcaatggaaaagcgtaatctaagggattgtcaccttgccaaattccatttgatgatgtcttaattgaagttatattcatcgccatatttggatgaaacaagaaa

>comp25730_c1_seq1 cation h+ exchanger 20 isoform 1

aaatccaagctgcgacatcgttgagggccgccgcagccatggcggtttcaccgataggcgtagttaggagcttaagctcggctaatacacgagccagtacagggaaagctgtgatggaaagtgctactcccatgaaaactaagtattgaggataactaaccatatcagctccatcaacagctttgtggaagataaaggcaacgccgacaccaagtgtaaaggtaagcgagatcccggccaccgctatggcgaaggcgcgccacccactccgccgtatggagttcaagtcaa

>comp25730_c2_seq1 cation h+ exchanger 20 isoform 1

atggttgccaacggcgatatggcggttgccggacggacgctgatgcagcccagttggctgtacgcctgacaagcgagggcgactcggtcgggcaattgcgttctgcggcggaggcgattgatgagagggaagccatttttcctcaaacgctggaccatcataattgatgaggatcgctcggtgagttcgaccagttgcataacatataatttcagacgggaggacttgttggtggagcgagttgactcaatgaggctgaggagtgagggaatgttgctagggccgtggatgcaggcaagaaatcggagttcgtcagaggag

>comp25730_c4_seq1 cation h(+) antiporter 20-like

ggggtagcacagccgcgggcgggtttgtagatcgccatgactgtcggagttgtaaggaaggtagtgaagagtgccattaggactagtattgcaaaagcctcgtcatttagaaccttcttctcctttccaatattcagaacaatcaactccaccaaccctttggtattcatcagcacgcccagcgttatcgactccctcgccggaatcatacacagcatcgccaccgcaaaagttccgatgatttttccggcgcacgccgcgaatatcaccagcgccagcagcccccaagcctcgccgccttttatcttcgtgacgtccgttttcagcccgctcgacgcgaaatacagcggcagcaacaatcccgaaacgaagtcttcaattctctccattaatcttccggcgaagtttcctcctttcggaattgttaaaccgaaaacaaaagcgccgaaaatcgcgtgaattccgatcaagtccgtcacaaaacccgccaccaaaacgccgccgagggttaagcagatgtaggcatcatccgccgtgttgtgctgccgcgaacaccgccgttccacccatttcatcgccggcttgatcgccaccatcatgaatgcaacaaaaacagtaccggataagagaacccatatggaaatcaacgggcttttccccggcccatcgccgccattgcctggaagggcgacagctagagcaagtga

>comp25733_c0_seq1 zeaxanthin epoxidase

ggatgtgggaggaggaaagatgcagtggtatgcatttcacaatgaagcaccaggtggaacagatgttcccaaaggtaaaaagacaaggttgcttaagttatttgaaggttggtgcgataatgttacagatctgctgcttgccactgatgaagatgcaattcttcggcgtgacatatatgatcgcactccaatcttttcatggggaaagggccgcgtgacattgcttggtgattcagtccatgcgatgcagccaaacttgggtcaaggaggatgcatggccattgaggatggatatcaacttgcacttgagctcgataaagcaaggaggcaaagcattgaatcaggaagcccagttgatgtagtctcttctttaaagagatatgagaatgctcgaaaacttcgtgttgcaattattcatggccttgcaagaatggctgcaataatggcatcaacttacaaggcatatctcggagtgggccttggaccattgtcgttcctgacaaaatttagaataccacatcctggaagagttggagggaggttatttatcgacattgctatgcctttaatgctaagttgggttcttggtggtaacggctcaaagcttgaagggagaatgctgcactgcagactttctgacaaagccagtgaccagttacagaaatggttcgtggatgatgatgctctagatcgagctcttgatgcagattggtttctatttccaattggagattcaactgcagcatctgagactatattcctaagccgagatgagaagaacccttacgtaattgggagcgtatcacatgccaactttcctggagtgtcagtagctataacttcacctcaggtttccaaaatgcatgctcggataagttacaaagatggagccttttttgtcacagatttaaggagtgatcatggcacctggatcacggataatgaaggcaggcgatatcgtgtgacccccaactcgc

>comp25733_c1_seq1 zeaxanthin epoxidase

ggattgtcggttcaaaacaggaaaatgagttcaactgtgctctacaatccaattaatccctcaataggagtatcatcaagaacgcattttccgttttcgaaagatttcccagcagaaatctgccgttctctgcaccagaaatccctgttcagaaaccaagaaaatgggctttccaagaaactgcgtagggtaaaggcaagtctaactgaagagccaaaatcggaaattgacggaaattccaagccacaagaaaagaatctgagaattctggtggcgggaggtgggattggagggctggttgtcgcattggcggcgaaaaggaagggatttgatgtgatggtgtttgagaaggatttgagtgctataaggggggagggccaatataggggtccaattcaaatacagagtaatgcattggcagctttggaggctatagatatggatgttgctgaagagattatgaatgctggctgcatcactggtgatcggattaatggcttggttgatggaatttctggcaattggtatgtcaagtttgatacattcactcctgcagcagagcgtggccttccggtcactagagtcattagccgcatgactttacaacaaatccttgctcgtgcagttgggtctgatattattatgaatgaaagcaatgtagtggactttgatgatgatggtgaaaaggttactgtgaaacttgaaaatggacagtgttatgaaggtgatcttcttgttggtgctgacgggatatggtcaaaggtgaggaaaaatttgtttgggccaactgaagctatatattctggctacacctgttacactggaattgcagattttgttcctgctgacatcgagacagtagggtaccgag

>comp25733_c2_seq1 zeaxanthin epoxidase

ttcttcacttctttgacgcttaatatcagtaaatggaaaccattggtaaattttcatcgcaattacagtggttaaaaaaagaatcacctatataatcgtacttgtacttccacattgtatatatacatgtgtttctattgctgctcataaatccgaaaacgatgctctcccagtaattcatactacgaatcagccttttcaatttcacgtataaatttgctgtggtgttatcctgcgccaaacaaaatgtcagcagctttacatggctcagctgccatcaataaattctgtacaggtttgctctgaatatctctgcatgcacttctatactgcctgcagaacttcatttttgtcctcgttcgtcgtcttaggtggaaacttcatagcctttacacgaaacgctgccttcttatcagacccaaactcaatcacatcggtagggcggaaacgagcaggcgagttggggg

>comp25743_c0_seq1 alpha- glucan phosphorylase l chloroplastic amyloplastic isoform 2

gccttccaaggatcccatcagttcatcatagttataaggcccaaaagcaccacttatgacaaattttttaacttcttcaaagcgttcatcgggcacaaacttgcctgcagctctttcttttctaagttctgctatctcatgagcttgagcaccaaaaaggaaaaagttgtcttctcccacttcctgcctaatctccacattggcaccatccagggtcccaatcaagacgcaaccattcattgcgaacttcatgttgctggttccactggcttccattccagcagtactg

>comp25743_c2_seq1 alpha- glucan phosphorylase l chloroplastic amyloplastic-like

atacaaccttcaacaagtcaccaatatctggatcatggtttatggtagccccaacatctgtgataaattttacaattctcttagcttgtacatatgtggcaaatgcttttccaccgaatatacaaactcgaggaacaaactttgcttccctttcagctgcagtcatttctttcattttcttatagcggtaaacaattcccaagatatttaatagctgccgtttatattcatgaatacgttttacctgaatgtcaaacattgcatcagggttaacggaataccctgttttttgtttgatgaatgacgcagccttagttttgttgcttagttttgcagccttccactcaatttggagatcctcgttatctgcaaacttcctcagttctactaatttttcagttttcagaacccaatccttagcgcctatccaccttgttattacatcacttagatctggattgcaaaaatggatccatcttctaggtgttaccccatttgtcttattctgaaacttctcaggccacaactggaaaaagtcattgaagacttctttcttaactatttcactgtggatctcagcgaccccattcacagcatgaccagccacaatgcaaaggttagccatccggaccatttttggaggtataggaactggttcctgatctatggctttactcttcttctgtgtttcctttccttcaggttcatctttatcagtacttgtggcttcatccttatcagtaattgtggcttcatctttatcagtaattgtggcttcatcactagcttcaacttcctcaccagtcttatccactgggctttcttctggtttggcaaataactcggcaatagaagtaggcagctcaaaattttctaaaattctcattgctgccaatttcttttccaaaatttctggatttgatgtgccgtattctgatactatgttttcaattagctgttcgtcaatcatctctatgatctctacatgtcttggaagtagtctttgcatgagatcataactccatttctccagggcctcaggcaaaacagtgtgatttgtatatgccacagttctctgagtaattttccaagcttcttcccaactcatacccttcaagtccatcaatactcttattagctccgggatacagagtgttgggtgggtgtcattcatctgaacagcaactttatcaggaagctcttcccatctcacatttccaccagatctcctctcaaaccgtgcaataatgtcttgaagtgaagccgagcacagtgtatactgttgtttcagtcgaagaatttttccctcctctgattcgtccccagggtaaagtatataacaaatcttctctgcattaatttgggcctcacacgctttagtatgttctccagcattgaaggcacataaatcaaattgctctgaa

>comp25743_c2_seq2 alpha- glucan phosphorylase l chloroplastic amyloplastic isoform 2

atacaaccttcaacaagtcaccaatatctggatcatggtttatggtagccccaacatctgtgataaattttacaattctcttagcttgtacatatgtggcaaatgcttttccaccgaatatacaaactcgaggaacaaactttgcttccctttcagctgcagtcatttctttcattttcttatagcggtaaacaattcccaagatatttaatagctgccgtttatattcatgaatacgttttacctgaatgtcaaacattgcatcagggttaacggaataccctgttttttgtttgatgaatgacgcagccttagttttgttgcttagttttgcagccttccactcaatttggagatcctcgttatctgcaaacttcctcagttctactaatttttcagttttcagaacccaatccttagcgcctatccaccttgttattacatcacttagatctggattgcaaaaatggatccatcttctaggtgttaccccatttgtcttattctgaaacttctcaggccacaactggaaaaagtcattgaagacttctttcttaactatttcactgtggatctcagcgaccccattcacagcatgaccagccacaatgcaaaggttagccatccggaccatttttggaggtataggaactggttcctgatctatggctttactcttcttctgtgtttcctttccttcaggttcatctttatcagtacttgtggcttcatccttatcagtaattgtggcttcatctttatcagtgattgtggcttcatctctatcagtaattgtggcttcatctttatcagt

>comp25743_c3_seq1 alpha- glucan phosphorylase l-1 chloroplastic amyloplastic-like

tgggatccttggaaggcaatgaaggcttcgggcgtgcagacttctttctcgtgggcaaggacttccctagttacattgaatgccaagagaaggttgatgatgcttatcgagatcaaaggagatggacaaagatgtcaatattgaatacagcaggttcctacaagttcagcagcgacagaacaattcacgaatacgccaaggacatatggaacattgagcctttggaaataatatagagatcaacagttgacaacttcaaccaaggcacacaaccagccactcctggatttccttcagaactcttgtatagagtattacttaagatcaataaattgtgtacaacaagaacaatggtaaactgcagtagatatgttgtatcgcttcaaaaccttttttccagcctgatatgtcccgattcatgaatcggtgtaaattttgtgaaaaaataagtttcctggtttg

>comp25743_c4_seq1 alpha- glucan phosphorylase l-1 chloroplastic amyloplastic-like

gagagagagagagagatagaggagagtggtggagcagtaatggcgacttcatccaccggaggagcggcggtggcgtactcgcgttgttcttccaacgccaggcttattgatttcacctccagatggcggagctccaatcgcttgttgttgagaagggtaaagccgtcatcctgcatcaggtgcgtcaccagtgagccgaagccgagtgtgagcgatccgatcactgaagaaggagttctcagcaatatgagttcttttcctcctgatgctgcatctattgcctcaagcatcaagtaccacgcacaattcacaccattgttttcttgtgagaactttgagccacccagtgcttactttgccactgcacaaagcgtgcgtgacacacttattattaactggattgcgacatatgatctttatgagaaaatgaatgtaaagcaggcctactacttgtctatggaatttttacagggcagagctttgctaaatgcgattggtaatttggagctcactggtgaatatgctgaggctctgaaaaagcttggtcattcccttgaaaatgtagcttctcaggagccagatgctgctcttggaaatgggggtctgggccggcttgcctcctgttttctggattctctggcaaccttaaattatccagcatggggttatgggcttaggtacaagtatggcttatttaagcagcaaattacaaaggatggccaagaggaggttgccgaaaattggcttgaaattggcaatccctgggaaattgttagaaatgatgtctcttatcctgtgaaattttttggaaaagttgtgaggggttcagatgggaagaggagctggattggtggtgaagatataatagctgtcgcatatgatgtcccaataccaggatatgagaccaaaacaacaatcaatctgagattgtggtc

>comp25773_c0_seq1 u6 snrna-associated sm-like protein lsm5-like

tagagaacagagaatcccacgtgagaaaactccgaacaaaattagttcacatggctcttcagcaagaaaattacaaccactaaacaagtttccaatgggccttcttgaagctgcaaatcaagatcttggacacgagaatgaggttgatcaatagacggtacatattcagtaacatcgatagaagcttcaagagaacacatgaaagtagtgcaagatatggccttgaaaattaaaatggcgttaccaacaggcaaccacaactaatacaatcagcaaccacaagtagaagaggttaataagtgtgctcgatcaacatgatcactacattcaactactacactgtttcatcatgaattttgcacatgaaatactacaaaattcaatccgatttatgagaagttaatgccaaattgccaatcatcaaacacaccacagtaggactggcaaagaacatgaacacctcattccgggtcaggtgatccaccaggaaccaatatggcaatgttgttgccattaagcaagatttgatcaagttttgtaatacggcgtccttcagaagtgatctcatattcagtgacatcttccaacaccatattaacatacacatcgaagccacggagggtacccaccaactccttgtcacccttcattataacccatattttcgacccgatacaccggtcaatcaactctgatgggaggagctgcgacggattgttagacgacatgattatgaacaaagaatttgcaaaacttgggagggtttttcaatctgggttttggttcaaaagcacgacag

>comp25801_c1_seq1 heat shock protein 70 -interacting

tagagcttcctcgtacctcatacctgccttcgcatattccttttgtgcccaatcataggcagctttaacctgcatcagtgtggattcagcagaactgtcttcagtagtaatcaccctcttccttgccttagacaagtgaacatttccccagttaaacaaggctaaagcagtcatctcttggaacttggctgcagcaatatcaaacagttcttgtgcatcgtcacttgtaacagtgtcttccatcgcttctgaatataatttcattcccatttcgtgaaggtccaggtaagaatcacaatcaaaccctacctgattcttaaacagccttgcaaactgg

>comp25801_c4_seq1 octicosapeptide phox bem1p domain-containing protein tetratricopeptide repeat -containing protein

gtttctggaacaatttgttcccttcttccttcaaatcttgggacatgttgataaagatggcagtgtcttcatcaaatgccttagagttacgctccgaagctttggtgtgctttgtgtttccatcattggattttgaccctatctggattttcttcttcgcagtaggctttcccatatttttactccaataatatgcgcaaaatcaaatgtttaaaatgaaagaaaaaaactccgattatgcacccttgaatgaaaacccaccttaaaagtaaattaagcacattaaatcaattaaaatactgtgaatattacattcatgaataataaaacacaaaaagtatcagccagtcaccaaacaaatgacggaatgcctctgaaaacaatctttaaaaataaaaactaaataaaataaaattttcaaaattaattcccgaaatcaaaacattgatggctcaaatccaccttcggagtcagttttaagagaagaataccagaaactacagattaaatgataatattaaagaaaatcattaagcaaacataaaactaccaaatcataccttggagcgagcgtttcgcggatcaagaataatgactgatgattacgagcgagctcagaaactcg

>comp25801_c4_seq2 octicosapeptide phox bem1p domain-containing protein tetratricopeptide repeat -containing protein

gtttctggaacaatttgttcccttcttccttcaaatcttgggacatgttgataaagatggcagtgtcttcatcaaatgccttagagttacgctccgaagctttggtgtgctttgtgtttccatcattggattttgaccctatctggattttcttcttcgcagtaggctttcccatatttttactccaataatatgcgcaaaatcaaatgtttaaaatgaaagaaaaaaactccgattatgcacccttgaatgaaaacccaccttggagcgagcgtttcgcggatcaagaataatgactgatgattacgagcgagctcagaaactcg

>comp25801_c6_seq1 carboxylate clamp-tetratricopeptide repeat protein

acaggtgctcgagctatataacaaggccgaggatagcatggataaagggatgcaaatgtgggaagagatggaagaacagcggcttaatgggctctcaaaatatgaaaaagataaagctctgttgcaaaaatttgggttggatgggctgtttaaagatatatcagcagatgaagcttccgagcaagcagcaactatgaggtctcagatatattccttctggggtacaatgctgtatgagcgctcggtcgtggaatataaattaagtctgccaacttgggaagaatgtctggaggttgcagtcgaaaagtttgaacttgctggagcatcagcaacagatattgcagttatgatcaagaaccattgctcgaatgaaactgctttagaaggattcaaagttgatgagatagtacaggcatggaatgagatgtatgatgctgataggtggcgcacaggtgtggcatcctcgcatctggaaccactgttcagaaggcgctcttcaaaacttcattcagttttggagcatctttgaatgatctttgcttgataatggtgatttaatgttcccatg

>comp25801_c7_seq1 octicosapeptide phox bem1p domain-containing protein tetratricopeptide repeat-containing protein

gcaaactggatgatccaatcctcgacacatgtcggccctttttcaacactaatattctctgtgaccaccagtggtttctcacagcttttgtgcaattcttcttcaatgccagttccctcataaattggttctttgtctggactaacttcagcaatgtacagtcttaaaaagccttgacgatcagccagtccctcagctatcctcagctcatcagttgtagtgatggtaaccaaatctccttcttgatctttatacttgataagcacacccttcaaacttggaaacctctccaacactatgtcccttaccagacgcatgccacaatttactggtaactgtgcccatcttatgtcctcaccatgaaccaatttcaccgatttcatgactaccttttcttccttgacacttcttttctcttctacaaccaccttatcctcagccttcttttcctcaacttcactgacatttttctcctcaggttcctcaatattcttccggtcatacttggtattcttcttctttttacctttctctttggcaattttcccagaaacaggtgcataagtaggctcaacataatttgggggcgaaacaatttccttgtcttcaatctgcacaccagttttttcaattgcctttttcaaattatcagcaatctccaaggcagtcaagttattgggttccatccccaacacattattgacatctcttaaagccaggtcaagcctgttcaatgcctcataacatcttgccctcctcaacagagccttgctgtacttgggagctacttgaagtgccaaattgcattcattaactgctctagggtactcaccaatgcccatttgcatgtaacaagcagccatgttggtccgcaaagacgcaacatcaatatggtttggcggcagcaatttgagggccttttcatacttcaacatagcgccctcatgatcacgtttctggaacaat

>comp25836_c1_seq1 probable polyamine oxidase 2-like

cccaccggccaccatcgccagtttcccctgcatcagcattccactttttctctttctctctctagaatcgtaaagccctctggaatttccatcccaatttcagattttcctgatagcccttttccctttttgtagaggatcaacggaatttattgccaataggcgtagtaattttggtgatttgttgggcttttatggttcaaactcaaatctcaatctcgcttaattttggagattttgttgggtttgattgatcgatcgggaggtgggtagatttcggaagacgcatgagcgggttggtcaagctgggtatttcttcgaattccaaccattttatcacttccattctccatttctcgaaaaccaatacttttttattattcctcttgttattgagattatccaagcttttattgggattaatatatttatcgagtaattttcgtagtaataattgtcaaatccaattttcgttgaattatggattcgagggacagcaacagtaatcgccaattgcgagccggtctttgctattcaaccttggagaggaaatctaccacatctccatctgttatcgtcataggtgctggttttgctggcatcgcagctgcacgagctcttcatgatgcctcatttcaggttaccttgttggaatcacgggatagaattggtggtcgtgtacatactgattattcatttggtttccctgttgacttgggtgcatcatggttgcatggtgtttgcaaggagaacccattggctccgcttattggaagattggggctaccgctttatcgcaccagtggtgacaactcagttctgtatgaccatgatttggaaa

>comp25836_c3_seq1 probable polyamine oxidase 2-like

tctgtatgaccatgatttggaaagctatgccctttatgatatggatggcaatcaagttcctcaagatttagtctcgaaggttggcgaaacatttgagagaattttgaaagagacagatcttgtaagacaggaattcagtgaggacatgtctgttcagcatgctatctcaattgttttcgagaggagatcagatttaaggttggaggggcttgaacacaaggtgttacaatggtacctctgtagaatggagggctggtttgctgcagatgctgataccatatcacttaagagctgggaccaggaagagttgcttcccggtgggcatgggctcatggtcaggggatatcttcctgttattaatactcttgcaaaaggtctcgacatccgcttgggccacagagtgacaaaaatagttagacgttataatggagtaaaggtaacagtagaagatggaagatcattcttagcagatgctgctgttattgccgttcctcttggtgttctgaaatcaaataccattaaatttgagccaagattacctgaatggaaagaggaagcaattaatgaccttggagttgggatcgagaacaagatagttttgcactttggcgaggtcttttggccaaacgtagagttcttgggagttgtttcggaaacttcttatggatgcagttattttcttaatttgcacaaggccacaggtcattccgtccttgt

>comp25836_c3_seq2 probable polyamine oxidase 2-like

atctagttaccttcatggattaaccctctaatatacaattcaactctgaacttgccttttgttttctgtatttacttttagagtgacaaaaatagttagacgttataatggagtaaaggtaacagtagaagatggaagatcattcttagcagatgctgctgttattgccgttcctcttggtgttctgaaatcaaataccattaaatttgagccaagattacctgaatggaaagaggaagcaattaatgaccttggagttgggatcgagaacaagatagttttgcactttggcgaggtcttttggccaaacgtagagttcttgggagttgtttcggaaacttcttatggatgcagttattttcttaatttgcacaaggccacaggtcattccgtccttgt

>comp25865_c1_seq1 early-responsive to dehydration 7 family protein

tgtcgccctgtttcaactgaactacggtaaactctccatttgcaagctgaacactctgttctttatcaatcaaatgaacgatcgagcccggaattctgattataacttcctcggatgattcaaacgatgcacttcctccacgattttttggatcttcttcttcagtttctgggaacaaattttctgctaaatcattcatttcaacagaagggtataaagaagaagaagaagggtttttgctggggcttgagatgaa

>comp25865_c2_seq1 early-responsive to dehydration 7 family protein

ggtccagtacgccgccgcgctcttctcaacctcatctcttttcttctccatctcctccggagtaacctccttcgccaccgctccccaccacccctgctccaccaccgccgccgccttctccaccctaaacgcactaaacttctccagcaccgaatccaattccctcagcagcccctcctgccccttagcagctatagtcaatccataactcaaaataccatcaccatcaatttcgccattttcacccggaacccggagactgaaaaaataatgcgaatcatcgagcttcacggcggcctcatctttggctaacggccattgaatttgctccccaactcg

>comp25865_c3_seq1 senescence dehydration-associated

tggaggattatagtggcagcgttgcgaggatgatagctgcggggtcggggcagttagtgagagggatattgtggtgtggagatgtgactgtagataggctgaaatggggggatgagttcttcaagaagagaatgacgaagcgatcaagctcggaaattagtcctcaggcattaaagaggatgaaaagggttaagcagatgacgaagatgtcacagaaagttgcaacgggattgctatctggggtcgtgaaggtctctggattctttacaagttcagttgcgaactctaaagtgggccagaaattctttagccttcttcccggagaaattgttcttgcttccttggatggatttaataaggtttttgatgctgttgaagttgctggaaagaatgtgatgtcaaccacttcagttgtgacaactgggcttgtctcacaaagatatggagaacaagcggcacaggtgacacatgatgggctaggtgctgcagggcatgctattgggaccgcttgggctgtgttcaagataagaaaggctctgaatccgaaaagtgtctttaaacccacaactctagcgaaggctgctgcagaagccaatgcagctaaattgaaggctaaacagaacaagtgaccgtgggcttcgcctctcgttttttgccaattaacttacagattgaaacttaggacctgtactagtttgtggttagaatgtcttactttggcttgagatgtgactgatagttcctttactccttgtcgttatcctttctggtttctgatatctatattccatcagaggtgtaaggattcagaaaattgtatggtcatgtatatatactatctctttttacgtttcaagggagtcatttttggtttatgaattctggttttgtgttagatg

>comp25865_c3_seq2 early-responsive to dehydration 7 family protein

tggaggattatagtggcagcgttgcgaggatgatagctgcggggtcggggcagttagtgagagggatattgtggtgtggagatgtgactgtagataggctgaaatggggggatgagttcttcaagaagagaatgacgaagcgatcaagctcggaaattagtcctcaggcattaaagaggatgaaaaggtaagtcttttttcgaatgcttgaatttgcaaatattggatttacattgctaatgttattggaagatttaggtatgttggtttctagtaaatcgtacattcggttcttgtgatttagctctgatggtcttgtgatttagctctgaattacttccccgatcatttttatgctgatcattagatgatatttaagttgctacatgttcgactatgtcttagctcctgttcttaaacacgttgatcaattaatcgtggtgtaaacagtctcaactgcttatgttaagtttcacttaattcttgtacataggaaatcagattgaattcttttaatggcaatttttaagctattttacttcactcatataactatctggtttaattcatcaacccactaatgaatttggatagatatttgattttgactgtttggatttcccagggttaagcagatgacgaagatgtcacagaaagttgcaacgggattgctatctggggtcgtgaaggtctctggattctttacaagttcagttgcgaactctaaagtgggccagaaattctttagccttcttcccggagaaattgttcttgcttccttggatggatttagtatgttactctgacttttgattgccatattcacattgtctttgatcggccaacaatatgataagtccatatatatatacatatatgcgtgtgtgtgttaaacacatatgtatatgtataacgcatgaatataattgtgatggcaaaaaataacgttgcttttccaaatgcgatatatgtgatactttttgcagtagtggaaacccaatcactttatcttttcactaacatcgttgtttacgtcattacagataaggtttttgatgctgttgaagttgctggaaagaatgtgatgtcaaccacttcagttgtgacaactgggcttgtctcacaaaggtaattgtttgctgcctacaaagttttgagcttccaatgaacttggctttggactttttcatttttatttttttatttttttctcttttatcactcttgcaaaacaatctcagcattgcacactttagaagttgagatcaggctccttacagtgcgattgaaaacccccccttcccgcgcgtttgtatttgctttcatagaactctggttatcctttatcattcggattacctaaatagaagatattactatgttactttttacagcacttgttcttacattggtttggtcttgttccagatatggagaacaagcggcacaggtgacacatgatgggctaggtgctgcagggcatgctattgggaccgcttgggctgtgttcaagataagaaaggctctgaatccgaaaagtgtctttaaacccacaactctagcgaaggctgctgcagaagccaatgcagctaaattgaaggctaaacagaacaagtgaccgtgggcttcgcctctcgttttttgccaattaacttacagattgaaacttaggacctgtactagtttgtggttagaatgtcttactttggcttgagatgtgactgatagttcctttactccttgtcgttatcctttctggtttctgatatctatattccatcagaggtgtaaggattcagaaaattgtatggtcatgtatatatactatctctttttacgtttcaagggagtcatttttggtttatgaattctggttttgtgttagatg

>comp25865_c3_seq3 erd7 protein

tggaggattatagtggcagcgttgcgaggatgatagctgcggggtcggggcagttagtgagagggatattgtggtgtggagatgtgactgtagataggctgaaatggggggatgagttcttcaagaagagaatgacgaagcgatcaagctcggaaattagtcctcaggcattaaagaggatgaaaaggtaagtcttttttcgaatgcttgaatttgcaaatattggatttacattgctaatgttattggaagatttaggtatgttggtttctagtaaatcgtacattcggttcttgtgatttagctctgatggtcttgtgatttagctctgaattacttccccgatcatttttatgctgatcattagatgatatttaagttgctacatgttcgactatgtcttagctcctgttcttaaacacgttgatcaattaatcgtggtgtaaacagtctcaactgcttatgttaagtttcacttaattcttgtacataggaaatcagattgaattcttttaatggcaatttttaagctattttacttcactcatataactatctggtttaattcatcaacccactaatgaatttggatagatatttgattttgactgtttggatttcccagggttaagcagatgacgaagatgtcacagaaagttgcaacgggattgctatctggggtcgtgaaggtctctggattctttacaagttcagttgcgaactctaaagtgggccagaaattctttagccttcttcccggagaaattgttcttgcttccttggatggatttaataaggtttttgatgctgttgaagttgctggaaagaatgtgatgtcaaccacttcagttgtgacaactgggcttgtctcacaaaggtaattgtttgctgcctacaaagttttgagcttccaatgaacttggctttggactttttcatttttatttttttatttttttctcttttatcactcttgcaaaacaatctcagcattgcacactttagaagttgagatcaggctccttacagtgcgattgaaaacccccccttcccgcgcgtttgtatttgctttcatagaactctggttatcctttatcattcggattacctaaatagaagatattactatgttactttttacagcacttgttcttacattggtttggtcttgttccagatatggagaacaagcggcacaggtgacacatgatgggctaggtgctgcagggcatgctattgggaccgcttgggctgtgttcaagataagaaaggctctgaatccgaaaagtgtctttaaacccacaactctagcgaaggctgctgcagaagccaatgcagctaaattgaaggctaaacagaacaagtgaccgtgggcttcgcctctcgttttttgccaattaacttacagattgaaacttaggacctgtactagtttgtggttagaatgtcttactttggcttgagatgtgactgatagttcctttactccttgtcgttatcctttctggtttctgatatctatattccatcagaggtgtaaggattcagaaaattgtatggtcatgtatatatactatctctttttacgtttcaagggagtcatttttggtttatgaattctggttttgtgttagatg

>comp25865_c3_seq4 senescence dehydration-associated

tggaggattatagtggcagcgttgcgaggatgatagctgcggggtcggggcagttagtgagagggatattgtggtgtggagatgtgactgtagataggctgaaatggggggatgagttcttcaagaagagaatgacgaagcgatcaagctcggaaattagtcctcaggcattaaagaggatgaaaaggtaagtcttttttcgaatgcttgaatttgcaaatattggatttacattgctaatgttattggaagatttaggtatgttggtttctagtaaatcgtacattcggttcttgtgatttagctctgatggtcttgtgatttagctctgaattacttccccgatcatttttatgctgatcattagatgatatttaagttgctacatgttcgactatgtcttagctcctgttcttaaacacgttgatcaattaatcgtggtgtaaacagtctcaactgcttatgttaagtttcacttaattcttgtacataggaaatcagattgaattcttttaatggcaatttttaagctattttacttcactcatataactatctggtttaattcatcaacccactaatgaatttggatagatatttgattttgactgtttggatttcccagggttaagcagatgacgaagatgtcacagaaagttgcaacgggattgctatctggggtcgtgaaggtctctggattctttacaagttcagttgcgaactctaaagtgggccagaaattctttagccttcttcccggagaaattgttcttgcttccttggatggatttaataaggtttttgatgctgttgaagttgctggaaagaatgtgatgtcaaccacttcagttgtgacaactgggcttgtctcacaaagatatggagaacaagcggcacaggtgacacatgatgggctaggtgctgcagggcatgctattgggaccgcttgggctgtgttcaagataagaaaggctctgaatccgaaaagtgtctttaaacccacaactctagcgaaggctgctgcagaagccaatgcagctaaattgaaggctaaacagaacaagtgaccgtgggcttcgcctctcgttttttgccaattaacttacagattgaaacttaggacctgtactagtttgtggttagaatgtcttactttggcttgagatgtgactgatagttcctttactccttgtcgttatcctttctggtttctgatatctatattccatcagaggtgtaaggattcagaaaattgtatggtcatgtatatatactatctctttttacgtttcaagggagtcatttttggtttatgaattctggttttgtgttagatg

>comp25865_c3_seq6 senescence dehydration-associated

tggaggattatagtggcagcgttgcgaggatgatagctgcggggtcggggcagttagtgagagggatattgtggtgtggagatgtgactgtagataggctgaaatggggggatgagttcttcaagaagagaatgacgaagcgatcaagctcggaaattagtcctcaggcattaaagaggatgaaaagggttaagcagatgacgaagatgtcacagaaagttgcaacgggattgctatctggggtcgtgaaggtctctggattctttacaagttcagttgcgaactctaaagtgggccagaaattctttagccttcttcccggagaaattgttcttgcttccttggatggatttaataaggtttttgatgctgttgaagttgctggaaagaatgtgatgtcaaccacttcagttgtgacaactgggcttgtctcacaaaggtaattgtttgctgcctacaaagttttgagcttccaatgaacttggctttggactttttcatttttatttttttatttttttctcttttatcactcttgcaaaacaatctcagcattgcacactttagaagttgagatcaggctccttacagtgcgattgaaaacccccccttcccgcgcgtttgtatttgctttcatagaactctggttatcctttatcattcggattacctaaatagaagatattactatgttactttttacagcacttgttcttacattggtttggtcttgttccagatatggagaacaagcggcacaggtgacacatgatgggctaggtgctgcagggcatgctattgggaccgcttgggctgtgttcaagataagaaaggctctgaatccgaaaagtgtctttaaacccacaactctagcgaaggctgctgcagaagccaatgcagctaaattgaaggctaaacagaacaagtgaccgtgggcttcgcctctcgttttttgccaattaacttacagattgaaacttaggacctgtactagtttgtggttagaatgtcttactttggcttgagatgtgactgatagttcctttactccttgtcgttatcctttctggtttctgatatctatattccatcagaggtgtaaggattcagaaaattgtatggtcatgtatatatactatctctttttacgtttcaagggagtcatttttggtttatgaattctggttttgtgttagatg

>comp25865_c3_seq7 early-responsive to dehydration 7 family protein

tggaggattatagtggcagcgttgcgaggatgatagctgcggggtcggggcagttagtgagagggatattgtggtgtggagatgtgactgtagataggctgaaatggggggatgagttcttcaagaagagaatgacgaagcgatcaagctcggaaattagtcctcaggcattaaagaggatgaaaaggtaagtcttttttcgaatgcttgaatttgcaaatattggatttacattgctaatgttattggaagatttaggtatgttggtttctagtaaatcgtacattcggttcttgtgatttagctctgatggtcttgtgatttagctctgaattacttccccgatcatttttatgctgatcattagatgatatttaagttgctacatgttcgactatgtcttagctcctgttcttaaacacgttgatcaattaatcgtggtgtaaacagtctcaactgcttatgttaagtttcacttaattcttgtacataggaaatcagattgaattcttttaatggcaatttttaagctattttacttcactcatataactatctggtttaattcatcaacccactaatgaatttggatagatatttgattttgactgtttggatttcccagggttaagcagatgacgaagatgtcacagaaagttgcaacgggattgctatctggggtcgtgaaggtctctggattctttacaagttcagttgcgaactctaaagtgggccagaaattctttagccttcttcccggagaaattgttcttgcttccttggatggatttagtatgttactctgacttttgattgccatattcacattgtctttgatcggccaacaatatgataagtccatatatatatacatatatgcgtgtgtgtgttaaacacatatgtatatgtataacgcatgaatataattgtgatggcaaaaaataacgttgcttttccaaatgcgatatatgtgatactttttgcagtagtggaaacccaatcactttatcttttcactaacatcgttgtttacgtcattacagataaggtttttgatgctgttgaagttgctggaaagaatgtgatgtcaaccacttcagttgtgacaactgggcttgtctcacaaagatatggagaacaagcggcacaggtgacacatgatgggctaggtgctgcagggcatgctattgggaccgcttgggctgtgttcaagataagaaaggctctgaatccgaaaagtgtctttaaacccacaactctagcgaaggctgctgcagaagccaatgcagctaaattgaaggctaaacagaacaagtgaccgtgggcttcgcctctcgttttttgccaattaacttacagattgaaacttaggacctgtactagtttgtggttagaatgtcttactttggcttgagatgtgactgatagttcctttactccttgtcgttatcctttctggtttctgatatctatattccatcagaggtgtaaggattcagaaaattgtatggtcatgtatatatactatctctttttacgtttcaagggagtcatttttggtttatgaattctggttttgtgttagatg

>comp25879_c0_seq1 zinc-finger dna binding protein

actctctctcctctctctctctctaaatatatatacacacacgtatgtaatcctacacaactcctccaccgccactccccacgcaactgccaacctccaaaaccttctcctatacttatatataaatccccttctccaaactctccttcactctctgacttcaactcttcaacaaaaagaaccctttcgtgaattctcccgatttttttctcaacttctgttccttcacgccttcaaacaaatggcccttgacgccttgaattcttctccggcggtggcgccgcccaatctttcccggcggttcgacgatgtcgagccgcgtagtgtggactcatggcagaaatctaaacgctcgaagcggccgcggcgggaccaatcagaagaagagtatcttgccgcttgtcttgtcatgctcgctcgtagcggcgacggtcgctacccctcctcctccgccaccgctagtcacgccaccggcgcaagcgaggacaaggatgagactatcgccgtccccaccagttctgccaccgcttcgcccaaagctgaagtcaagaaagaccaagaaacctcccccgctacgccaccgccttcggtggcggccaccgccaattcttacaagtgtagcgtgtgtgataaggttttcccttcataccaagcactcggcggccacaaagccagccaccgtgtcaagcccccaaccgcaaccgcggcggcgtccgacgacagcaatcactccgccagcgtctcaacggcggcgaattacatctctgctctcactccaagtggccggcttcacgagtgctccatctgccacaagacttttccgaccggtcaggctcttggcggacataaacgcagacactacgacggcgtaatcggcggtggtgctgctaagagtcgcaccacttcctccaacggcggcgccgccagtgatcagatgaccgtagccgtcccccggaatatcgacttaaacctgccgccatctccagaattgcagtgtgatgaagaagtgcaaagcgctctgccgcctttattcccttaaataaattttagtagaaaaagtagcttttattacttactttttttaaacctctttttaggacaagtttatggtgctattatttgttatggtgatgt

>comp25959_c1_seq1 transcription regulatory protein snf2-like isoform x1

gcactgcttgaacagagatcaagttgcttgcctggttctaaattatcaggcttaaaggaaaacaggtttcagagccgaattcaacatcggttaaccgaacttgaggaattgcccacaagcagaggcgaggacctgcaatccagatgcttgcttgaactctatggacttaagttagccgagctccagagtaaagtccgctctgaagtgagttctgaatattggcttcgtctgcattgtgcaaattctgacaagcagctatttgattggggcatgatgcggttgcgtcgtcctttatatggtattggagatgcttttgctacagaaactgatgatccactgaagaagaaacgagaggctgagaggttgtcaagatttgaggaggaagagaggaaccgtatagagactaggaaaaggaaattctttgctgacttacttaatgctgcacgtgagctccaattgcaagtacaggctgcccagaaacggcggaaacagagaaatgatggtgttcaggcttggcatggaagactcaggcaacgagccacacgagctgaaaaattgaggttccaagctctgaaagctgacgatca

>comp25959_c3_seq1 transcription regulatory protein snf2-like

gagagagagggagagggagagagagaaacctcactctaattttttcaaaaggccctcgcacgacgccgtattcggccctcccttcccgccatggtcgcacaggtggcggagccacagccctctgcttcccaccaattgggcctccgtcaggccgagcccggcccggggcgcgaaaacccgctcggcagtgccaagacgctaatttgcgccctcaatttcctctcccgcaatttgcccttgccccggcatgtttacgacgccgtttcctccatttaccaagactccgcaactgatagcggccaggaggtccccgccgacgaagctgacggtgacgccgagggtgatgtcaggaacggagcaacttcgccgtcgcggacggatgattttggtgtttcaacttatgatgagttgatgttggacttcgaggatgcactgcttgaacagagatca

>comp26002_c1_seq1 heat shock protein binding

gaatgcaacaaaagtaatttactgaaaaacaagagccagtctgacccttttcatggctacatctatcataaatcataccccaaatccttacaatataaaaggctaaaaatgattcgactttgctctcacgtgcgtcttttctggcttttctgtttcctcccttaccattcattattcacccgctccattcaattataacgccattgtttcctttaatgacaatgaattacatcccccacaaagacaaggttacattaaaaggaagaaaatcggtggacaattagcaggaaattaagaaaagccagtgtgcagaaccatggtttcgtgctgtatgacatgaactgcgatgtgcgaaagaagcttatatgttaataatataagtagaatccataaaaatgaatggaaaaataaaaagaagccaagccaaacaaaaatgatggagagggtcatatataagttggtataacgtgaagaggacaagaaaagaaagaaatgctagggggtagagagagtactgcacagtgatttgtatgcattgacacaatgcttgaatttctcttcagcagctacttgtgaagaaccttgatgcctgtcaggatgccacttcaaagcagccaagtgaaagctgtgtgaaaaacaattgtcataaattcttacagtgaagtt

>comp26013_c2_seq1 dna-binding protein with miz sp-ring zinc finge isoform 1

gatagtcatgcaggtcttagactaggaatgagaaagaaccaaaatagtagttgggagattaacaacgaaaatgatgtgcagggaatcttatctgcaaacagatttgaagaaaattttgggaacaatggccaaactataattcccatgagcagcagtggtactggcagtggcagagattgtgaggatgttagtgttaaccaggatggtgggggaaacctcgattttaacagttttgagtatgagtccatttccctgaacattgatccagtacatggatttaatgatagaataacttctgcaccaactgtggatactgaagtgattgtcctcagcgattctgaggaagaaaatgcgccactaatgtcctctggcgccaatcacaagaatgctgctccagataatggtggagtcccatttccatctgcacagcatggaattcctgattcttattatgacaatcctgctcttggtctttacagttcgaatgacgatgactttgggattaatatgtggtcattgccttctggtagtcagggaggtccggggtttcagttatttcgttcagatttggatgtttctgaagccttagttgaaatgcagcatggttctcttaattgtacatcgcctgttaatggctatacgatgactgcagagactgccatgggatctgctgcacttgtccctgagtctactgctcaatgtactaataataatgatggcttagtggataatcctttagcatttagcagtaatgacccttctcttcaaatatttctgcctacaagaccatctgatgcatcagtggggcagtctgatttgagagaccacccagatgtttcaaatggtattcctactgaggactggatctctctcaggcttggggatggttttggaatgggtgaaggtgaatctgctgctggaaatggtttgaattcaggacaagtacaaccaaaggatgctgccttggattcattagcagataatgcatctttgttgcttggaatgaatgacaatgggtctgataagacaagtagggagagatcagatagtccttttacatttcctcgccaaaggcgttctgtaagaccaagattat

>comp26013_c3_seq1 dna-binding protein with miz sp-ring zinc finge isoform 1

gggctgccacaggaaatgctgataatgacagtgatattgaagttgttgctgattccattcctgtcaatcttcgctgtcctatgagtggtttaagaatgaagatggctggaagatttaagccctgtgcgcatatgggctgttttgatcttgaagtgtttgtggaaatgaaccaacgctcgaggaagtggcaatgtcctatttgtttgaagaactattctttggagaaaatcatcatagatccctatttcaatagaatcacatctaagatgcaaaattgtggagaagatgtggcagagattga

>comp26013_c5_seq1 dna-binding protein with miz sp-ring zinc finge isoform 1

atcagcatttcctgtggcagccccaccaccaacacatctacgaacacgagccaatgcatcctcaaaatgctcgccctcagcctctgatggaatcaagttgagaacctggtgcaacgtgcgcctcttggcaattctaactcccacacaaaatatgcgagcatcacctcctcctatgaagatcttgttaattccatctctggtccatggtgtaataacaggtccatcatcacggccatttgctcccagtaactgagagccaggtctgtttatcgccctcactggtacaccattgatctgtagatctgcatattgcggccagtgcatcctaaatgtcaccttgtcattaagaagcatgcaccatgcctgaacatcatattcctgtttcgacaacaaatccctatctggccttgtgagttgaaatgttttctcaatgctctggctggaatttgagccatctgtagaaacatttgtgacattcaacttcaccggcagcagaggatgtgcaaccgtcacccagaagggatcagcccgagcgagtctgcaaatttcacagtaaaaagtatctggaggatttg

>comp26013_c5_seq2 dna-binding protein with miz sp-ring zinc finge isoform 1

atcagcatttcctgtggcagccccaccaccaacacatctacgaacacgagccaatgcatcctcaaaatgctcgccctcagcctctgatggaatcaagttgagaacctggtgcaacgtgcgcctcttggcaattctaactcccacacaaaatatgcgagcatcacctcctcctatgaagatcttgttaattccatctctggtccatggtgtaatctgacaaagaaacaagacacttactgaa

>comp26062_c1_seq1 u-box domain-containing protein

ttttactcacataattacggtcaagcaatatatttgctggctttagatcacgatgtatgatagcttttggctttgaattgtgtaggaagacaagtgctgaagctatttcccaggcaatccggaatcgatcataccataaaagtggaggagtattatttttcctcatcagtctatcttcaaggctgccattctccatgtactcatatactaggcaatttcggtcaggacatgcacccaaaagaatcagcaagtgttgatgacgaattcggctcaatatttccagctcttgctgaaattgttttgttctgcttccttccttagtatggagaactttcacagcagcagttgtatgctggaacctgcatttgtatacaattccatatgctcccattccaattttaagactatcagaaaatgatgaagttgcggacatgatttcttcccatgtaaattgacggtactgatagaaacagccattcaagacactttcaagattttccttttctttggtttcacgagacaatcttatctctgcttcttttctttctgctgcttctctttcagcacattccttgacaagttcagcttctcttttagcagcttcatatctctttttctcctgtatgaccaactctttggcttcttcctccttaaaacttatctgtttgagattaatttcctcctccagatgatgtttctgaagttcatttaactttctagtagcatcaatcgcctcgccttgggccattgcatacattcctcgaatatgtctgagttcaattctcaatttttctagctccaaattgatattatcctggttcttggtagaggttttagaagttgacgcttg

>comp26062_c2_seq1 u-box domain-containing protein 35-like

tttgcaattgcttacagcaaaaccggcaattgccctagctcataaagtggaaattgcagtggacaataattgtttgatggaagtactagatccagaggctgatgcatggccaatcaaagaagcaaaggatttagctctcactgcattaaagtgcacagaacttaggggaagagataggcctgatttacgagatgaagttcttcctgctttggagaaattgaaagattttgctgagaaagctcgagattcggcattagtttcagcaccttcacctcctaagcacttcacatgccccattctcaatgaggtaatgactgacccctgtgttgctgctgatgggtatacatatgaccgcagggcgatagaggcatggcttcagttgaaagatacgtctcccatgactgaattaccattgccgcataaatacctcatacccaattacgcccttctttctgcaattatggaatggaagtcttgaagtcaaacataaatatctatcttctgttgtgattttctcatttttttctgtgttgtaaatgcaattcatagtattagatgcttcaaaatgtatgaagtagagagagtagtgatagaacaacctcttttactcttaagcagaaggaaaaagagaaactcaggcttgtagttcttgaatagagtgttcgagttattacttttacgaggaagaatgtaataccataaacttcaagtgtaaatatggaattccttaaaaa

>comp26080_c0_seq1 probable pyridoxal biosynthesis protein pdx1-like

ctctgtctctttctctctctaaatttccccccctctctctagtccgttcactttgcgatttctgcttttccgaaagaaataatggccggaagtggagttgtcaccgtctatggcaacggcgctatcaccgagaccgccaaacaatcccccttctccgtcaaggtcggccttgctcagatgctccgcggcggcgttataatggacgtcgtcactcctgagcaagcccgcgtggcggaggaggccggcgcctgcgccgtcatggccttgg

>comp26195_c4_seq1 heat shock 70 kda protein 17-like

attcatttatatgtaagaagccaagaaaccatctcatctccaagaaaaatcaatgggtaaaaatatccttcaaggaactttgtattcaaaccaaatcaggaaacaaacttactttttccgatccttcactttatcaaaaaatacatcttctatcatgactagccaactgtaagtaaaaacttacctttgaaactatcaattatttacctgttgtagctctgtcaagtatctctctgcatgttcagatgtagccaactatgcagtgaggtccatatgcctaagataaatgactaggactaacttcaacatgcccagacgttcttgaaattcagtggcagaaggatcttcaccatcaacatataaccattattccaaaaacattagtgccttagtggataagtttaccaagtatttaaacacaatgcttaactaaaatccataaggcagcaagtacacaaaattgatattcagaaaaataatcccaaaatacaagataatgatccagccaccttagcaccgg

>comp26225_c4_seq1 xanthine dehydrogenase 1-like

agagatgcgggcctgactgggacgaaacttggttgtggtgagggaggttgtggcgcatgcactgtgatgatatcctattttgaccacaacttgaagaaatgtgtgcatcttgcaattaatgcatgcttggctccactgtattctgtagaaggaatgcatgtaattacggttgagggtattggtaatcgcagatatgggttgcatccaatacaggaatcattagcaaactcacatggctcacaatgtggcttttgcacccctgg

>comp26225_c6_seq1 xanthine dehydrogenase 1-like

aaattccttccataaatgatgtacccttcagatttagtgtctccctcctgaaggatgctccaaatcctaaggcaatccactcatccaaagcagttggagagccgcccttttttcttgcctctgctgtattcttcgccatcaaggatgccatcatagctgcgagggctgaagtaggccttagtgattggtttcgtctggataatccagcaacccctgaacgtatacggatggcttgcatagatgaattcacgaaacccttcattcactctgatttccatccaaaacttagtgtctagtgtcaatcctggatccatactcaataccggaaaataaaagaggggagagagggggaaaaaaaatcactctcagtacttttcttgaattatagattgaatccttagatgcaataatttgttccccacttgttacaggacaacgaactccatgcataagattgtatggtcattgtaatccaacatgctttgtaat

>comp26244_c0_seq1 type i inositol -trisphosphate 5-phosphatase 2-like

agaaatggctgaatatcaagcccatggtttatgactttagtgaagatgaagtcgacacggagagtgaggatgacgcttgctcgcttaaagacgaaggtttgcacgatgaccatgttgatgcaacgcagggatttcaatccatatgctcaagtgcagacagaaaaccttcaaaagaatgctcgggaaaacaccggaggggaaaatcagaaactctgcgacttcagtacataaataccaaagatgtgagagtaacaataggcacttggaatgttgctggaagacttccagatgataatcttgagattgatgaatggcttgatatgcaagagccagcggatatgtacattctcggttttcaggaggtggttcctctgaatgctggcaatgtacttggagcagaaaatagaagacccattcccaaatgggaggctatcatccgcaagactttaaataagtcgcttgaacctgaaactaaacataagagctacagtgcacctccctctcctgttcttaggacttcctctgcttctgatatattggctgatgtggctgatgctcctgtactagatatatgtggagggaattctgttggcaccaccatcggtggtgataccaatatgaccaactgctggaagaacatccagttaaagagaatatatgatagtgattgggacaacagattggactggccggaacaatcattggatgctacacctcaagctctattttccaggtccaatttacgaagagtactgagcagttcagcaagaattggttctggctggatgggtaatggtcttaccgccagccctaaaaattttccactaactggttctggcttaaaaagaatgcactgtagctctggaaatctaggattgctttatatggaccaggaagatgagcatgaaggagttcaagattctctctctgataagttggatcaagtctctggagaggaggaagattcatttgtagaaatgacagagttcagacacgaaaatgaactttcagctaatgcaataaagtcccatccagcatatgtacggattgtgagtaagcaaatggttggaatatatgtgtcagtgtgggtacgcagaaggttaaggcgacatataaaccacttgaaggtctctccagttg

>comp26322_c0_seq1 universal stress protein a-like protein

gagagagagagagagagaatggcagaaaaagggcggcggattttggttgctgttgacggaggcgaggaaagcggctacgctctttcatggtgcatcaagaatttgatatctgaacgtaattccaaagatacccttattctcctctttgccaagacgcctctacccatctactcagccacggattcaacagggtacgtattttcgagtagtgtagttgcgacgatggagaggtatgcaaacgaggtggctgagggtgttatggacaaggcgtgcagactttgcaaagagttgaatttgaatgaccaggttaaggtggagacgatggtggagcatggtgatccacgtgatgtcatctgtgaggtggcccaca

>comp26322_c1_seq1 universal stress protein a-like protein

tagtcatgggaagccatggctatggtctcattaagagggcatttctaggcagtgtgagtaaccattgtgcacagaaggtgaagtgcccagttctgattgtgaagaagccaacagattaaagcaaatgatgacatcattaattaattcaaatgcatgcttcttcaatcaatttcaaatattgatacaaattaaatcgttgtatggtgttgtactataatttcatgttgtatgaagggtgttgtatcataatttcatgttctatgtcatttttacctcttctgctttgggccatcggccgacgtgtaatgta

>comp26504_c0_seq1 probable polyamine oxidase 2-like

tataacagcaatatatcgtattgtccatcaccatatgatcattacaaccttctacatttcttccctagaaacacacattaaggaagactacaaacatgaaatggtgctttgatcattcaattgggaaatcttttgatttccacaattgctcacaaagcaaccaccgtggattactagctagcgttttcttacaggttactgattacatacgagagatcaatagcgggacagaaaccggaaccccctcgcccatgactggctggaaaagatccagctctccatacctctccaagacatgcattctacagtcctcagcagccattagaccagtcgagtatgcaccatggaccgatcctggataatctatgcttgttgcttccccagcaaaaaataggttatccaccgggatccttagctgttcatatagttcgtggggtttcccaactttgtcgtagctgtaagagcctaacgagtttttgtcagccccccagtgcgaaacaagggactggatcggtgcagaaacatttggtaggattttttga

>comp26504_c1_seq1 probable polyamine oxidase 2-like

accgggtgaccagtggccttgtgcagattaagaaaataactgcactcataagtagtctccgcaacaactcccaagaactctacattcggccaaaagacctcctcaaagtgcaaaattatcttgttctcaattccaactccaaggtcattaatcgcttcttgtttccactcggggagtctaggctcaaatgtgatgcaatttgattttagaacaccaaggggaacagcaactatagcagcatctgccacgaatgttgtcccatcttcaacagttacctttactccattgtaacgcctaataatttttgtcactctgtggcccaatcggatgtcaagacctttagcaagggtattaataacaggacgatatccccggaccattagcccatgcccaccaggaatcaactcctcctggtcccagcatttaagtgatatggtatcagaatctgcagagaaccagccttccattctgcataggtaccactgcaacaccttatgatcaagtccctccagccttaaatctggtctcctcttgaaaacaatggagatggcacgtcttatggacatgtcctcgctaatttcctgtcttacgagctccgtctctttcaagatgctctcaaatgtttcaccaactttcgagactagatcttgaggaacttgatttccatccatatcaaaaagtgcgtaactttccaaatcatgatcatacaaaactgagttctcctcactagtgcgataaagtggcaatcccagtcttccaataagaggggccaaaggattttccttgcaaacaccatgtaacctgaaatgcagcatcatgaagagctcgtgcagccgcaatgccagcaaaaccagcacctatg

>comp26504_c1_seq2 probable polyamine oxidase 2-like

accgggtgaccagtggccttgtgcagattaagaaaataactgcactcataagtagtctccgcaacaactcccaagaactctacattcggccaaaagacctcctcaaagtgcaaaattatcttgttctcaattccaactccaaggtcattaatcgcttcttgtttccactcggggagtctaggctcaaatgtgatgcaatttgattttagaacaccaaggggaacagcaactatagcagcatctgccacgaatgttgtcccatcttcaacagttacctttactccattgtaacgcctaataatttttgtcactctgtggcccaatcggatgtcaagacctttagcaagggtattaataacaggacgatatccccggaccattagcccatgcccaccaggaatcaactcctcctggtcccagcatttaagtgatatggtatcagaatctgcagagaaccagccttccattctgcataggtaccactgcaacaccttatgatcaagtccctccagccttaaatctggtctcctcttgaaaacaatggagatggcacgtcttatggacatgtcctcgctaatttcctgtcttacgagctccgtctctttcaagatgctctcaaatgtttcaccaactttcgagactagatcttgaggaacttgatttccatccatatcaaaaagtgcgtaactttccaaatcatgatcatacaaaactgagttctcctcactagtgcgataaagtggcaatcccagtcttccaataagaggggccaaaggattttccttgcaaacaccatgtaaccatgaagcacctaggtcaactgggaaaccaaatgaataatcagtgtgcactcttccaccaattctatcacgcgattccaacaagataacctgaaatgcagcatcatgaagagctcgtgcagccgcaatgccagcaaaaccagcacctatg

>comp26632_c0_seq1 glutathione peroxidase

gaaatttttgggtaccttgaactgtctcttaatggcggccgtccaaaattaatttggtggttcatctacctaagactggcgcatcaagaaatcgacagagaatttttacaactggaaatcttaggtactcccccttttccatcgactaagcttcaacgagatttcagcaaactgctgaagaaatactacattctgtatttttctgaacacaaaacatgtttttcttgagacctatcaactgggcatctcttttctttcttggacttgccttctggttatattacaggaaccctggtgccaaagatgtagctgcagattccccaaaatctatttacgatttcactgttaaggatattcatgggattgatgtgcctttgagcaattaccgagggaaggttcttctgattgtcaatgttgcttcaaaatgtgctttgacccagtcaaattacaaggagttgaacattttgtatgagaaatataaagatcagggttttgaaattcttgcatttccttgcaatcaatttgcttatcaagagccaggaactaatgaggaaattcaggaagctgtatgcactaggtttgaagctgaattcccaatctttgagaagaaaattgaccaggtaacatgactggttaaaacctaatccagattgaggtcaatggaaagaatacggcgcccctttataagttcttgaagtcagagaaaggtggtctatttgtcaatgctatcaagtggaattttacgaagtttctggtaaacaaagaaggaaaagtagttgaaagatacgctcccacaacaccaccacttgcaattgagaaagatgtgcaaaat

>comp26632_c0_seq2 glutathione peroxidase

gaaatttttgggtaccttgaactgtctcttaatggcggccgtccaaaattaatttggtggttcatctacctaagactggcgcatcaagaaatcgacagagaatttttacaactggaaatcttaggtactcccccttttccatcgactaagcttcaacgagatttcagcaaactgctgaagaaatactacattctgtatttttctgaacacaaaacatgtttttcttgagacctatcaactgggcatctcttttctttcttggacttgccttctggttatattacaggaaccctggtgccaaagatgtagctgcagattccccaaaatctatttacgatttcactgttaaggatattcatgggattgatgtgcctttgagcaattaccgagggaaggttcttctgattgtcaatgttgcttcaaaatgtgctttgacccagtcaaattacaaggagttgaacattttgtatgagaaatataaagatcagggttttgaaattcttgcatttccttgcaatcaatttgcttatcaagagccaggaactaatgaggaaattcaggaagctgtatgcactaggtttgaagctgaattcccaatctttgagaagattgaggtcaatggaaagaatacggcgcccctttataagttcttgaagtcagagaaaggtggtctatttgtcaatgctatcaagtggaattttacgaagtttctggtaaacaaagaaggaaaagtagttgaaagatacgctcccacaacaccaccacttgcaattgagaaagatgtgcaaaat

>comp26634_c0_seq1 mitochondrial heat shock 22 kd

acatttcagtcttaatccaagaaagaagcctgatataagacatcgagtactactgcaagaaaaaggatcgtcaacaaggctacatttaattcacttgttgataatctctaagtataatcatctaaactaccacttcctcacaaagaaaatttgcccactgcaaaccataccaaagaaattcactataagtcttcagcataacacagaagaaactagatcaatacagaaaaaacactgtgcttcttatttcaaaacttgcccttcaacaataactacaactagtaccactcctaagatgatacaattcattccacattgacatggaaaacatcagccctctcttcctctttgatcttgggcacaaaaactttcaagacaccatttttcatctccgcctttatttcgttagtcttgtagagtttgtccgggagatcaatcctgcttgtgtaccttcgcccgccctcatcttcatcatcaaactctttcttgccttcccctttaattatcaaagtgttttgctccaccgaaatcttcacatcctccttgcctaatcccggcatatccacgcggagatgaagtccgtcatccatctcccgggcatcccagtttcgccgaatgctagaactgactggcgattccatgaactgatccatcatgtttagaatttggcttaggctgctccttgttggaaacggaccccatatgtctgagaagggagagaagatgcggcgatcgggacggcgatcgacgtccacatcgcgatcatcactgtcgtactggcgaacggcgttggtagtgaagaggcgagaggaggcaaagttagccaccggacggagcgacctcgacaggagattggaagagaccagcctctttagagcgagcgaggaagccattcttacggttgagtagatatcaagaggga

>comp26681_c3_seq1 aux iaa family protein

aatttaattataacaagccagacaacagatgagtgcattttgcttcttgtgcaatacatatgtctttagatttttttttttaataactatataatataacaaaatacagacacagtcacaaaaaccaagaaagaaaaattgaaaaaaattacaggaaaaaacaaatactgggcacatagaaacaagttcctgctggatcaaaccaagttgcacaagtaggtttcattacatagtttcatcagcacctgctcttgcatttctccatggcccttggtgccagtccaattgcttcagatcctttcataatgcgaaggcgcttgcatgaatcaacaaacatctcccatggtacatcccccacgagcatccagtccccatccttatcttcataagtgggcacatattcagaactgttcaacagatccatcaatttgctctcattcatgaagtctatcattccttgggtcccataattacccatggtgaaggagctgaacattttggctaaagcatcggagagttgttgatagctcttgtacattttcaagtccactttacggagatagggcgccccgtccatcgacaccttgacgaaggcggccgcgccgctgcatgccgccttctcagcctcctcattgccgctcttctgattggccattatgttcttgcggaaagaccgcactggtggccatcccaccacttgtgccttggctggtggcttgactggatccttgggaggaacagcagccttctccatagctgagttttttatgctttccttcagatctaaagcagctg

>comp26681_c3_seq2 auxin-responsive protein iaa14-like

aatttaattataacaagccagacaacagatgagtgcatttgcttcttgtctaatacaaatttgtccagaaatatttcatatctatataactacaataacatacagacacttcgtcgcaaaacccggaaaaaaataaaattaaaaaaatcaccaaaaaaaaaagaaaaaaaaaatactggcacataggaagagcttcttgctccatcaaaccaagttgcacaagtagttcattacataatttcattaacatctgctcttgcatttctccatggctcttggtgctagtccaattgcttcagatcctttcataatgcgaaggcgcttgcatgaatcaacaaacatctcccatggtacatcccccacgagcatccagtccccatccttatcttcataagtgggcacatattcagaactgttcaacagatccatcaatttgctctcattcatgaagtctatcattccttgggtcccataattacccatggtgaaggagctgaacattttggctaaagcatcggagagttgttgatagctcttgtacattttcaagtccactttacggagatagggcgccccgtccatcgacaccttgacgaaggcggccgcgccgctgcatgccgccttctcagcctcctcattgccgctcttctgattggccattatgttcttgcggaaagaccgcactggtggccatcccaccacttgtgccttggcaggtggcttgattgaatccttgggaggaagcatagctttctccgtacaagaattttgcatgttttccttcagatccaa

>comp26704_c1_seq1 autophagy-related protein

ggaataaaaccaaactactgttggcatataactaccattaactgaaccattggattgttggatggatccattacaagggatagatgacccctcctgaacattaatatatccagaaaaggacccatcagcgcaaataatcagcaatgggcgactgtctgcaaatttatccccagattggtttgacaacactggttttggtaacatttgcataaatgatactggaccatcatgcctggagactatattccgcacattgtctgctgcttcaacatcccagacctgaaatccataactgaaccccaacaagagaacctgtcgcgtgatacctctttcaagttctagtttgtcaaacccggcccacgaaacctgatcatggttggggtcaccgtccctctccacaatagccgaagctgcagaggcagcagacctcacagtggaggccacggtcgatgcaccagaggaaacatacttcag

>comp26704_c4_seq1 autophagy-related protein

agtaggtactggatttgagaggattgtatactctctctctaacgtagcagcattgagacaatgtatctgactggactgtaagacggcaacaactcgagaactgcatcgcactaaatggacaactgatctaaatcttaaaagatgtacatatgactgagttctcaaggaataaaaccaaactactgttagcatataactaccattaact

>comp26792_c0_seq1 regulator of g-protein signaling 1-like

taccaaataaaatatcattttgtaatagatgatttgcactgcttgtaccccctttgcttccatactaaacgatttacataaggttacaaaagaacttccaaacctacgaaactaccaactctaacggacattccttctcatacaacattcacggagaacacatgcgacaacattaattgcctacgagaagttgccaaaattgcagtggtccatatagcatgttacattagaaattggcaaaacaactcaggaagcagaagaacatagaatttcaatatggctgcaaacagcaagctgctgaagtacgatttctctaagcctcagcacatcgggaacttccatttttgtagtgtcgtctaagcaaatcttgtttcagaccaagaaagtctcgtggttcgtgtgaggactagctaggaatgaagaagcatattattatctgctcatgtcattgcagctctagttcatggctgctagagcccaactttctagggctatgttcttggtgaaaagggtcatcagcacaatgcacggaactcagcctatgtgagaaattccagccaccctgttctagctcatggtcaactgttttcattctggcctcttctttcagcttcatgaagaatgtcgatgaccaataatcattcaccaaattcattgtcatcaattgaatcaactcatttagtgcatttttaaagagattggggtgtgtcaaatcaggagtagatagaatttcttgtcgacaacgatgagaaatgttcacctccattgttgcaccaggagtaatatacatttcaataatatgccgtgccatgtaaattcttctaacatgatcaccaactggaattttgtcaagctgttgcacctcctcatagaaatgaacgctctccccagctagacagctatctgcaaattccataaacgactgacggaatcttctgttcaaaagaagcttatccaaaggtccattaggatctaccaactccattgattccctttgtactaaaagcccactatcaggaatacccaacgcccggcccatcctactatactcctggggatcctttttcctcaagctcataagtgcgatgaggggttgtgatattgacatggagaagaatgccagcagaagggcacttgtcataactagtaataaagatcttgtgatgatctgaaccaatgaactaccttcgtaagtctcatttagaatgtaggcaaccacccatatcccaatgcaagaagtggagactaaaattccccaccagagctccttgagttcatggaatctaaactctatatgtcgaacagctccagtgaaaccaaccaaagcagca

>comp26792_c0_seq2 regulator of g-protein signaling 1

gtgtctgaaaatggaggtcaggaaacagactaacctttttcatcaactgaatcaactcatttagtgcatttttaaagagattggggtgtgtcaaatcaggagtagatagaatttcttgtcgacaacgatgagaaatgttcacctccattgttgcaccaggagtaatatacatttcaataatatgccgtgccatgtaaattcttctaacatgatcaccaactggaattttgtcaagctgttgcacctcctcatagaaatgaacgctctccccagctagacagctatctgcaaattccataaacgactgacggaatcttctgttcaaaagaagcttatccaaaggtccattaggatctaccaactccattgattccctttgtactaaaagcccactatcaggaatacccaacgcccggcccatcctactatactcctggggatcctttttcctcaagctcataagtgcgatgaggggttgtgatattgacatggagaagaatgccagcagaagggcacttgtcataactagtaataaagatcttgtgatgatctgaaccaatgaactaccttcgtaagtctcatttagaatgtaggcaaccacccatatcccaatgcaagaagtggagactaaaattccccaccagagctccttgagttcatggaatctaaactctatatgtcgaacagctccagtgaaaccaaccaaagcagca

>comp26792_c0_seq3 regulator of g-protein signaling 1-like

taccaaataaaatatcattttgtaatagatgatttgcactgcttgtaccccctttgcttccatactaaacgatttacataaggttacaaaagaacttccaaacctacgaaactaccaactctaacggacattccttctcatacaacattcacggagaacacatgcgacaacattaattgcctacgagaagttgccaaaattgcagtggtccatatagcatgttacattagaaattggcaaaacaactcaggaagcagaagaacatagaatttcaatatggctgcaaacagcaagctgctgaagtacgatttctctaagcctcagcacatcgggaacttccatttttgtagtgtcgtctaagcaaatcttgtttcagaccaagaaagtctcgtggttcgtgtgaggactagctaggaatgaagaagcatattattatctgctcatgtcattgcagctctagttcatggctgctagagcccaactttctagggctatgttcttggtgaaaagggtcatcagcacaatgcacggaactcagcctatgtgagaaattccagccaccctgttctagctcatggtcaactgttttcattctggcctcttctttcagcttcatgaagaatgtcgatgaccaataatcattcaccaaattctacaaaaataagttggatcaggaaaagatgtacatttcaggaaaaggcgttcaatgaattcatatcgtgtctgaaaat

>comp26792_c1_seq1 regulator of g-protein signaling 1-like

gcaaagcagctgaaatctgaaaatggcgaattgtgcagtcagaggaggctgccccagcgactatgtggccatctcaatcgctctgctctctgttatactgcttcttgccagggttggtacgccctatctaattcataagattccaagacctaacggcagtggcttttggcttgtagcaatacaagtttttgccagcttcaaccttttactgtcacctgtgatggcccttggttttttgaggttgagaaggaggcactggtggtgctacttatgggctgtttgggtcgaaggtccactaggatttggcttgctgttgagctgtcgtattgcacaggcgtttcaactatataacatatttgttaagaggcgtttgccaccagtccgatcatttatatttcttccattggttctcttgccctggattatagctgcagcatttattcacattgaaaagcctctaaacaaccgttgccacatgggaactcagtggat

>comp26814_c2_seq1 rubber elongation factor family protein

gatttgaggaatggatgatatataaaagttgggggtaatcaaaatgggggaatcaacttatttttattctcatgaacttatatcaagcactattaatacgaatgattcccccatcacacacatcctcacctctaataataccccaaccaacactaaatattcagattgacgcaagtattcctcaataacacgcttgtgtaaacagagctctctcttgataaagtcacaataatgacagaacctaagcttcaaacgaaaagacttaacgcgacaaaaaaataataccaatatgatactattacacacattaagtattccatataatcaaagaaaataaacatcatccatcaccaaggcgataatcataaatactacccaaagagagcaagattaatctgttttctatccgaaattaatgagtttgtgatgaaaatatcactattgcggaaatgaagcatcaacagcttagcccgatcgcttggcgaacaagcaatcaatttttatgcgtcaaaaatcttagcaatcctttcagtaggtactaatggaagatgtgaggcaaccttgtaccctttctcagcagcttgttgaacggtttggttatacttctctgtacaatagctggctgttggagtaacagcttgagccacttgaggaaagagcgggagctgattcagggagcgccaggcagatgctgcgtattgctcggccactggttcatacttactgcaaagatccttagcagcaggctcatacttggcatacatggtttttgcaagccct

>comp26838_c1_seq3 protein dehydration-induced 19 homolog 4-like

cctaatcaagtcctgaacactgaatattttaatcaatatgataataccaaagttccactatttcatgaaaaatcttttgagtcacaatacctaagctctaactgcttccgcgtatctaaaacattatcatagggcaattaatacaacagtacacccccccaccctcaaactaagcgccacatacacaaaaacaaacacgaaaaactagacataaaggaggtcagtgaagtatgtaccccattcttggcttcaacagcatgctcttcatcaatatgacaacaaagccccaccatatcaaaatcctcagtacaaaatgggcataaatactcaggcttctgctcctcttccccctcatactcttctccgttatatacatctgatcgagatcgataacgcctagaataactagaaaatcgggtccatgaatcggagtccatttttcctgaaatttccaattctgaatcaagaaaaatatctcaaaatagatttacactccgcaaaacccagttgcttacacgcaccgagtgaaaaatgaagacatatgtgaatatacttcggggcgtgcgtgtgtgtgtgtatatatatatatgtaaaagaaaaacaaatgtgaaggaaaaaaagaaagctggagaagagaagcttcggagaaattagtgactaaagagagccagagaggaagctctccaccctgt

>comp26858_c0_seq1 nadp-dependent glyceraldehyde-3-phosphate dehydrogenase

ggaagcgtgcagagctccttcacaaagcagctgctatccttaaagagcacaaagcccctatagcagaggctttaataaaggaaattgcaaagccagcaaaagatgctgtcactgaggttgtgaggtctggggatttggtttcttattgtgctgaagaaggtgttagaatcttgggagagggcacattcctcgtatctgatagttttccaggaaatgaaagaaccaagtattgtctcacatccaagattcctcttggtgttgttctagcaatcccacctttcaactatcctgtcaaccttgctgtctccaaaatcgcaccggctctcattgctgggaactcccttgtcctcaagcctccaactcagggtgcagtggcttgccttcacatggtacattgcttccacttagctggtttccccaggggacttatcagctgtatcacagggaaaggctctgagatcggtgattttctgacaatgcatcccggagtgaattgcataagcttcaccggcggtgacactggaatttccatttcaaagaaagcaggcatg

>comp26858_c2_seq1 nadp-dependent glyceraldehyde-3-phosphate dehydrogenase

tttcgcccacaacttttgtgcggcttttgctgtttccatggccttattcacctcttcttgtgtacaagcttgaaccttgtactgagtctttcttgtagtgggatttacaatggaaacagacttcccagaagaagacttcttccactctccatcagcatagtacttaaacacatctccatcaataatctctgcaaaaactccactgcccgccattgttactccctcaaaaaccttcttcacgccactttttcctgtatcagagatatataaataaag

>comp26907_c0_seq1 lactoylglutathione lyase glyoxalase i family protein

ttatgggtattttgtttgtcttgttaattagttataatgccacccacttctttaattacagctatgaaacatacagcaaggaatgcaagtaaatgcatttagtgcaaattcacgatagataccgcatttgatgatagtaatattttatcggggtagcatggaaaaacccttattttattcactaatattaaactaaattttaaagaaaaacacataaacggtgcaaattgacgataaatactgcatttagtggttgtattttatcagcacagcatcgcaaaaaattcactaatattaaacaaaattctaaagaaaaaagcatgatcaacaccattaaccttaatttaatcatgtttatgaggaatgtttttggttgtttatgaatgcaaatctttttatggcagtgtgaaaacatgggcttggtggagaagaagctggcagaaatggggattgattcggtgcggcagagagtagacgaaggtggagtctacgttgaccagttgttcttccacgacccagatggattcatgattgaaatctgcaactgtgataacatcccggtgataccattggctggggaagttgtacgttcttgctcgagactgaatttgcagatggtgcagcaacagccgcagcagcagcagatccatgtccccgtggtacaaccttagataatatgtggattgggatgtaatcggatactctctggcctatctttgtttgcatgctcccttgtaatcttcgctgtcgcgtttgtttggtacttctgttgtaataagacgcttttgcctttgggtttaaataggaattcaaagtttctcttcttcaatgtttgatcgatttttctttgttttgattttatttgtacaattttgtgttcctcttttctcaactggcatcttatcatcattgtcatggttttatagatattat

>comp26909_c2_seq2 aldehyde dehydrogenase family 3 member h1-like

tatgctacttttctgtgaaaacaaagaccaaagaggaagatgtaccgaattcttgttttagctatgggaaccttttattgtattaattttgttctatttctttgtattttctatatgaaggtcaacaaaattgaggatggcattggcctgattaattctaaagaaaaacctcttgctgcatatatattttcaaatgacaaaaagctccaggaggaattcaaaagaagcgtctcagcaggtggcatgtgcattaatgagacaactctacatcttgcagtgcctggtttgccatttggaggcgtgggtgaaagtggaatgggagcataccatgggaaattctcctttgatgctttcagccataaaaaggcagttttacaacgtgggtttaatggcgatgtggctgcaagatatccaccatacaccccctggaagttaaaatttctgaaagcattgttaagtggcaatataatcagtattattcgtgctttggttggttggtaacatttatcaattgt

>comp26909_c2_seq5 aldehyde dehydrogenase family 3 member h1-like

aaaaaagctccacttgatgagatcttgccagtttagtgactcaaggggcttcacatgtttcagttaggggcataatacatggttaaattgtctctttcttgactcttccttgatattttgctaaatctgtctccagtctatcgttggatgcaatcaggtttatgcttgagtgtcctttaatggtgaagtgtgtgtgtgtgtgttatatttctcaaaattttctaatgctgttatctaatgtcatatagaaaataatgcacttatcaaaagaaaaaaggatgaaaatacaacttgatggtcctgtatccacaaacttcttggtgcagcttgcagtgcctggtttgccatttggaggcgtgggtgaaagtggaatgggagcataccatgggaaattctcctttgatgctttcagccataaaaaggcagttttacaacgtgggtttaatggcgatgtggctgcaagatatccaccatacaccccctggaagttaaaatttctgaaagcattgttaagtggcaatataatcagtattattcgtgctttggttggttggtaacatttatcaattgt

>comp26911_c0_seq3 transmembrane proteins isoform 2

ttctttaatttctgctgctgccgctaaaattcgcaaaaactgacccaaaaaaataaaaaacaaaagaaaattccttcacgaatctggcattagcattagcaaaatagcaaatatgtcttcggcgattactcagctgtcatgtttctcttccattgttaataaccgtcgattgcagctccagggcggatcgtgctcgtgccccgcccgttttcgtcccgtaaaggttttaaatattagcagcgacggaagtggcatagatgtatccaacttggagaataagacagccccaagttacccagaagatgcatccaaatcacataatggaagcactgtcaaatcacatcctcttacagaacgttctgttgcagcatcaaatgggcacatacaggaacaagtcattagccaaccaaaaagggcagcaaagattcatgatttttgttttggaattccctttggtgggcttgtattaagtgggggacttcttggctttattttctcaagaaatcctgctgctttaggcactggtatgctcttcggaggtgctttattggcccttagcactattagtttgaaggtctggagacaaggaaaatccagcttaccgttcatactgggtcaagcagtactctctctggccctcctttggaagaactttcaggcatactcatcgacacaaaaagtcttcccaaccggttttaacg

>comp26931_c0_seq2 heat shock transcription factor a2 isoform 1

gacaagaaacgcattgaaataggccaaaagagaagattaacaatgagtccaagtgttgagaacctccaggaagttgtagccatttccacgggcagtagccagccttcgaattatacaggagaagaacaggaagagcttgcagatattcaattgaaggtggagagtatgttttctgctgcattaggtgatgaaccaagtagtgatctaaagggtctttcagcagagacagtgccctcgacaagtgaaaccagtttgaataatatgatgaatctatgggaggagttccttaatgaggatcttatagccggtgatgaggcagaagaagtcttggcagctgaggacctgtctgatgttgaagtggaagaattggtagctaaaacacctgagtgggatggactcaagtaaaaagagcagcagtgaagatctcaagtagaaggtttgatttcttggacaacagttgaaaacgttctcatatttgtcctggatgtggtgcaatacaactcctttttgtcttgtatctataagtatgggttattgtatgttagacgtcacctcatcgaggcccgtattagtttttttggccaatttagcatatgtccattatactttttatgggagagaggtgaatattatttcttctgccaatctaggttttgtcaagagttctatgtttgaattgactgtctcagctgaggtaatgcgacataaatcttgaggaagcaattatcctgttgcatttcagcagctccaactgtttgagttaataacattctgtactattagatgatatctaaataacttcttcatgaaatttggaattcaagtcacaagtcatcatgagaatacaatttttcacattatagaaacaggttatggattcatgcaatttgctctgtcacttagctccaggatttgtgtttcttattggcagggaatgaggccaagacacagagacttacaaaggataagaatgggtgctgcttttgggataattattacagaaagccactgatttcttaagaatctgattcaatcactttttgcctttgaggtctgtcgtttctctggttagctgggtagcctctcttatgttcttataatttgccctgctgctgttgctttctgctgttgcacctgcagtttttccagaaattagaatcgtggttgccaaaattgaagatctatccgggctggaagaggacatggtagagtggaggctattctgatgtccctgtcatgggattgttcttcctgacttttgccagactaggttctgaaaacctagaaagattgttcttttgcaggg

>comp26931_c0_seq3 heat shock transcription factor a2 isoform 1

gacaagaaacgcattgaaataggccaaaagagaagattaacaatgagtccaagtgttgagaacctccaggaagttgtagccatttccacgggcagtagccagccttcgaattatacaggagaagaacaggaagagcttgcagatattcaattgaaggtggagagtatgttttctgctgcattaggtgatgaaccaagtagtgatctaaagggtctttcagcagagacagtgccctcgacaagtgaaaccagtttgaataatatgatgaatctatgggaggagttccttaatgaggatcttatagccggtgatgaggcagaagaagtcttggcagctgaggacctgtctgatgttgaagtggaagaattggtagctaaaacacctgagtgggatggactcaagtaaaaagagcagcagtgaagatctcaagtagaagggaatgaggccaagacacagagacttacaaaggataagaatgggtgctgcttttgggataattattacagaaagccactgatttcttaagaatctgattcaatcactttttgcctttgaggtctgtcgtttctctggttagctgggtagcctctcttatgttcttataatttgccctgctgctgttgctttctgctgttgcacctgcagtttttccagaaattagaatcgtggttgccaaaattgaagatctatccgggctggaagaggacatggtagagtggaggctattctgatgtccctgtcatgggattgttcttcctgacttttgccagactaggttctgaaaacctagaaagattgttcttttgcaggg

>comp27017_c1_seq1 heat stress transcription factor b-3-like

gcagccggcggagttcgccagggacctgcttccgaccctcttcaagcacagcaatttctctagctttgtcaggcagctcaacacttacggatttcgcaaagttgcgacaaaccggtgggagttcggcaacgacaagttccgcaggggcgaaagagataaactctgcgagattcgccgacggaaagcaatgtctaacaagccgcagcctaatgtacaagtcgcagcagcagacgacaacgacgagcaaagatcattatcaacatcatcatcgtcggatttcaccacgctcatcgacgaaaacagaaggctcaagcgagaaaacggcctcctgaattccgagctcatcatcatgaagaagaaatgtgaagaacttctcaagttggtgaccatttatggagggaatacagagaaagaagaacagtatgtcgaaatggaagaagaaaataatggttgtttaaagctttttggagtgagattggaagttggggatgagagagagaggaagaggaagagggcagaggtcagtgagactgcgagggttcttctgtctcaatcatgcaaatattcttgtggattagggctttccatt

>comp27064_c2_seq2 crt dre binding factor

gaacacttcggcaggaggaataatttttcagatgaagaagtgattctagcttcaaacaacccgaaaaagcgggcggggaggaagaagtttcgcgagacgcggcacccggtgtaccgcggcgtgcggcggcggaactcggggaagtgggtgtgtgaagtgagagagcccaacaagaaatcaagaatctggctggggaccttccccactgtggagatggcggcgcgtgcgcatgatgtggcggcgatggcattgcgagggaggtcagcttgcctgaactttgctgattcggcgtggcggctgccgttcccagcgtcatcggatcctaaggacattcagaaggcggcagcagaggcagcggaggctttccggcctcagtcattggagtcaggggatgaaacaaaggatgagacggccaccgcagcggtggcatcgccggaaaatgtgtgctttatggatgaggaggaggtgtttggaatgcatgggttgctggc

>comp27069_c1_seq1 peptide methionine sulfoxide reductase chloroplastic-like

acagtgacgcacatatagaaggaaaaagaggagaaaaagaaattaattattagcaaatgggctctcaaatactgaaaatctcgccatttgcagcttctagaacgttcattgtcagcgccccagctttctcaagatttctacctaacccaattaaccatatccctaaatcccgccgttgtgaatttaccaaattccttagatatagcagcagtttatcaagtgggctttctgggtggagtcttaatcagggaaagaagagtttcagaagtggggttgtggccatggctgcagctggctcagtgcagaagtcggaggaggagtggcgtgccattttgacccctgaacagtttcatattctaaggcagaagggcactgagtatcccggtactggggaatatgacaagttctatgaggagggcatatacaaatgtgcagcatgcggaactcccctgtacaggtccactaccaaatttaattcaggttgtggttggccagctttctatgagggtctccctggtgccataaatcgaacacctgatcctgatggaaggaggacagaaatcacttgtgcggcttgtgggggacacctcggtcatgtctttaaaggggaa

>comp27082_c2_seq1 arm-repeat tetratricopeptide repeat-like protein

tgctctatagcaatagagcacaatgtaatttgctgctaagggatccagattctgccattagtgatacgacacgagctctttccttgtctacccctccaaattctcatgctaatagcctctggacaagatcacaggcctatgacatgaaagggatggctaaagagagtttgatggactgcataatattcatcaatgcttgtatcaaatcgaagacagctaatcgtgtaagagttccgtattacgcagtgcgtatgatcagcaagcagatggactcaacatggcttttcagagctgctcagctgaaggcattgaactatcatcctgaaaaggcga

>comp27082_c3_seq1 arm-repeat tetratricopeptide repeat-like protein

attagaattctatgttacagcaaggttggaagagacaacatctcaagatccagacgtgttattgagaatctatgtaacctttcaagatcttcggacgattggcagtacatgggaattgattgtcttctattgcttctcaaggatccagatacaaggtacaaagttatcgaagttgctacctcgtaccttgttgatttgattgagcttaggaaccttggcaacaggacaaatgttggtgaagcaatcacaagggcacttgtctatgactacaaataccaaaactcaaaaatcaagaatgctgatgtccaaagatcactagaagaaatctggttttccaagatagaaagaagaaagagagagcaatttatgtcaactgagaagcttgaagaaagaagagtcatggttaaactgataaaacaacagggaaatcataaatttttggtgggagacatagaagaagcacttttaaattatactgaggcattagaaatatgcccattgaggcatagaaatgaaagaattgtgctctatagcaatagagca

>comp27100_c1_seq1 abscisic acid receptor pyl8-like

agcgagatgcattgatgatgatgatgatgaggcgacacacacattagttctcactgcttccaaaacgcgaaaatggcagttctgagtctgtacggatctcgttgagtcaactcgccaacggctactcagctgtatatattacgttgatcggaggtggaattacaccggcgaaggagaagaatatgatgaacgtcaaaggattgagcggtgtggagagagagtatataaggaggcaccacaggcacgagattaaggataatcaatgcagttctttcctcatcaagcatattaaagcgcctgttcatcttgtttggtctctggtcaggagctttgatcaaccacagaagtacaagccttttgtcagccgttgtgttgtgcagggaaaccttgaaattggcagtctgagagaagttgatgtcaagtcgggtcttcctgccactaccagtaccgagagattggagcttcttgatgacaatgaacacatacttagtgtcaggattgttggtggagaccacagactcagaaactactcttccattgtctctgtccatcctgaggtcattgatgggagacctgggaccatggtaattgaatcttttgtagtggatgtgcctgaggggaacacaacggatgaaacatgctactttgttgaagcactgataaagtgcaatctgaaatcccttgctgatgtttcagagagacttgctgtgcaagacaggactgatcccatcgatcatgcctagaactgtg

>comp27159_c0_seq1 nitrate transporter -like

gtcttgtccatcttcgccatggtggctgcagcgcttaccgaaatcaaaagattaagggtagcacaatcacatggtctgacacgcgcgcaggttacaactataccattaagtgtcttctggttggtcccgcagtttctcttggtggggtcaggcgaggcctttacgtacatggggcaactcgatttcttcctaagagagtgccccaagggaatgaagacaatgagcacaggattgtttttgagtacactctcactgggattctttgtgagctctattttggtgagcatagtgcacaaggtgactggccacaacaagccatggttggctgacaatctcaatcaagggagactatacaacttctattggctcttgacaattttgagcattttgaatttgatagtgtttttggtttgtggaagatggtatgtgtacaaggagaagaggccctctgaggagggaattgagttggaagagacagagccaagttgccactgatgcaattaatttttgtattcaaatcataggtttggttataacttatttcaactattaaaagaaaaaaatatgggagaagagtcatggatgtgaaaaagactctttttcttttcttttttttttctt

>comp27159_c2_seq1 nitrate transporter

tttcggatccgatcaattcgatgaatcagacaagaaggagagaaaacaaatgatcaaattcttcaactggttcttctttttcatcaacactggatcacttggagccgtgacgattctggtttacgtccaagataatattgggagggagtgggggtatgggatctgtgcctgcgccattgttgtgggcttggtcgtgtttttgtcgggcaccagaagataccgtttcaagaagcttgtgggcagcccactgacgcagattgccaccgttttcgtggcggcgtggaggaagagacgccttgagctgccgtcggattcttctcttcttttcaatgttgatgacaacgctactgctgatggggcgctgagcaagaagaagaagcaaaaattaccacacagcaaggagttccgtttcctggacaaggcagctatcaaggaccctcaagtaccattcacggtagtcaataaatggtacctttcaactttaacagatgtggaagaagtgaaattggtgataaga

>comp27159_c2_seq2 nitrate transporter -like

acacctacttgggaaggtatttaaccattggtatctttgcaactgttcaagcaacgggtgtgacagtcttgacaatatcaactgtgatccctggcctccgtccaccaaaatgcatccccggcagggagtcctgcattccggcaagcggcaaacagcttatggtcctctacacagctctctatctaaccgccctgggcaccggtggtctgaaatccagcgtctccggcttcggttccgatcagttcgacgaatccgataagaaggagaagaaacagatgatcaagttctttaactggttcttcttcttcatcagcattggagcacttgcagctgtcactgttcttgtgtatattcaggacaatcttgggagagaatggggatacggtatttgtgcctgcgccattcttacaggtttggtggttttcttgtccggcaccagacggtaccgtttcaagaagcttgttgggagccccctgacgcagatcgcctctgtcgtcgtggcggcgtggaggaagaggcaccttgagccgccgtcggattcttcacttctctttgacgttgacgatattgttgctgatgaagggcggagcaagaagaagaagcagaagttaccacacagcaaggagttccgtttcctggacaaggcagctattaaggatgcagccatgccaatggagaataaatggtacctttctaccttaacagatgtggaagaagtaaagctggtcatcagaatgctacccacatgggccacaacaataatgttctggactgtatatgcccaaatgaccacattctcggtgtcacaagcaaccacactcgaccgtcacatcggcaaatcattcgaaatcccagcagcatccctcaccgtcttcttcgtcgctagcatcctcttgaccgtcccactttatgatcgtatcattactcctatttgccggaggcttttaaagaacccgcacggcctctcgccgctacaacgaattggggtcggactagtcctatctatagtggccatggtggcagcagcgctaaccgaagtcaaaagacttcgagtagcgcattcacacggtctcacacatgaccccgcagccacagtgccattaagcgtcttctggttggtcccccaattcctgttggtggggtcgggcgaggcgttcacgtacattggccagcttgatttcttcctgagagagtgccccaaggggatgaaaacaatgagcacagggttgtttctgagtacgctatcactagggtttttcttgagctctattttggtgaccatagtgcacaaggtgactggtaagaagccatggctggctgataatcttaatgaaggcaaactttatgacttctattgggtattgatgattttgagcattttcaatttggcgatctttttgtcttgtgcaagaaagtatgtgtacaaggagaagaggcttgctgacgagggtattgagttggaagaaactgaacctgtttgccactaagtcgcactttgaaaattaatttgttgccagaatatagggtttatgacttgttttatgtaaaacatggaaacggaaaagagtcatacaaatgaaaaacaagcggctcttttcttttttctttttttcgggtcttttctcatgtaatatatgtaggcagccaaaataataacaaaagttgtttggctaataaacatttacagaagccgactatggactggttaaacggccatgtttcgcgcatgctgtgcggtccatatctttgtttaatagtgaaagtattgagaatcaatgtg

>comp27159_c4_seq1 nitrate transporter

aaactttttccatatagccaaaagctctcatttatcgcatcccaactgcatctcacgggcccaaaactatatccaaacaaatggctgcactacctgaaacaaaacaagaggctgaaactctcccggatgcctgggactacaagggcagccccgccgttagatcctcctctggcggatggacgagcgccgccatgattctaggggttgaggcatgtgagcggctaaccacactagggatagcagtgaatcttctcacatacttgacaggaaccatgcatttgggcaatgcaacttctgccaatactgtcaccaactttcttggcacttctttcatgctgtgtctgcttgg

>comp27246_c0_seq1 ornithine cyclodeaminase

gcacaaaattccaatctctctggctagtaagtctcctgtctccctcccatggcggctaccaaacaagagcaagtcaaccccccggcctccgccacaccgatcttcatctccaccgccgcactccactcgctcatcacccacaaatccctcatcagccacctccaatccaccctcccgccactctccgccgccgtccaatcccccctccgccacgcctaccaagttagccccaattcatccttcctccttatgccttcttggtccacctccccactcctcccttacattggtaccaaactagtcacccacaattccaacaactccactctaaacttacccggggtgcacgcaatcttcgtgctctttaactccctcaccggccagcccttggcctccatcgacgccaccgagctcacactctaccgcaccg

>comp27246_c1_seq1 ornithine cyclodeaminase

gcccctcccttcaaatccagccccttgagcaatgctacttgtgaatttgtggcacaacttataacatcaccctctctcacagcctcctccaaacattgattaatctcaaaagatatcccttttagcgctgtctcctccctcaattgatcaaccaaaattcttgccttttcaattgttcgattccacactacaacccttttaatgcttggtctaaccgtcaagtgagccttgatcaaatgtggagctaatgaaccagcgccgatcatcacaagagtcgcagcgtcttcccgcgataaataagacgacgcgagagccgaaacggaggcggtgcggtagagtgtga

>comp27347_c0_seq1 histone h1

aaattcgccggcaacacagccttatgcttctcttccatgtactttgcgattgcgtacggacttgacccactcttctcattcaatgccaagagagcctccttaatcatctcaaaatacggcggatgagcagcagttttggcagccttcggcttcttctccttcggcgctgccctggttttcttctccttaaccggcttctccggcgcaggaggctgctccgccgcgggtttctggacttcctcagccgctgacatcacgatttcacacgatcaacacagaaatgaaaatgagacacagtgtgatttgagaaagtaaaatgctttgatcacaatgaacgaagcagcggtagttttcactttata

>comp27347_c0_seq2 histone h1

aaattcgccggcaacacagccttatgcttctcttccatgtactttgcgattgcgtacggacttgacccactcttctcattcaatgccaagagagcctccttaatcatctcaaaatacggcggatgagcagcagttttggcagccttcggcttctgttccttcggcttcttctccttcggcgctgccctggttttcttctccttaaccggcttctccggcgcaggaggctgctccgccgcgggtttctggacttcctcagccgctgacatcacgatttcacacgatcaacacagaaatgaaaatgagacacagtgtgatttgagaaagtaaaatgctttgatcacaatgaacgaagcagcggtagttttcactttata

>comp27356_c0_seq1 low temprature induced-like protein

atgatcactggcctcaaaaaagaaataataattaataatatgatcacatcaccgagattgccaagaatcccatctcaagacacactcataattaaactttacattaaatatgccaaatcactaaagaaaaacatagatacaataaaataattacatcaataacattgtattaacatagcaacatatgcaatgagacaaaccaagaccatagaataaatcactatacgacacatcataatacgaagaaagggacttgcagcctaactccaaacaccaaatcaatcaggaccgtccatctagaaatagtaaaaacatcgatggccgtttagatctccaccatgagaaaggatcaagtcctggtgatgatgtagacagcatatataattccaggtatataacccaatatcgttagaagcaaacatatccagaattgatgcttgcagccatacttgaggaaaacacccaggggaggcaaaagaattgccacaagaatatcaatacaagttgctgcgccttctgccattttcccttgatttctatttaattttccaaacaaataggattaagcaatctgtttttggggaattaacttaacacaccacagatatcaaggttagaagaaaattttgcaactgcttcttaagtgaaagggaagaggggttacggatttataacagaggagaaacaagtggttatcagtggggat

>comp27400_c10_seq1 dolichol kinase

taacagtgagtttcaagttaagattacatgctataccttcaattccaatttcctgcaatgactccgctctcagactctgtatcaacttagagaccaagagaccaggaagtgtgactgcacccagcagaactccagatgaagctccaggcctggttctgaagaagtgaacaagaggattggaggcatcgtcggcggatatctcgacggagagagcaaaaaaggagagcagagagagggagaaagcttcggggaggagagagagaggtatggagaagagaattcgaacgacatagagaaacaccaccgctctctcgccgttgagctgctgcaacagccgcggcgccattgctcttccgtcggcgatctctttcactcgccggagagacaacgaattccaagggggagagtaatcccaaagattttgtcttgtcaaatggttatggggtttgtgggga

>comp27414_c0_seq1 heat shock 70 kda mitochondrial-like

cttgctattctcccagcatctttggtggcttgccgctgggcatcattaaaataagctgggactgtgataacagccttgttgacagacttcccaaggtaggcctctgctgtttctttcattttagtcaaaacaaaagcaccaatttgacttggggagtactgctgtccattggcttcaacccatgcatctccgttaggagccctcacaattttgaaaggaaccatcttcatttccttctgcgtctgagggtcatcaaagcgtctaccaatcagacgcttggttccaaagagggtgttggttggatttgtgacagcttgtcgcttagcaggtgtaccgactaatagctctcctttctggctgaaagcaactactgacggagtcgtccgagcaccctcagagttctcaataactttgggattctttccctccatgacggcaacacaagaattcgtagtacccaaatcaataccaatgacatcatttccagcaggttttgtgctgaatggccttgcaagacctgcccacttgctgcctaaaacccatgatgatttggtgtttccagcaaaggttctaaaagcagaaacaggggcagaggaaaattcacggcgacgaagagatctgagaagaacggcggtggcggccattgataagagagagaaacggagagagagagacctgcttcgtaaatctgccggctcctttattgtgcagcagttcctgctagggtttagggtttacgaggaggagctttgaggaggcggattgaag

>comp27414_c0_seq2 heat shock 70 kda mitochondrial-like

cttgctattctcccagcatctttggtggcttgccgctgggcatcattaaaataagctgggactgtgataacagccttgttgacagacttcccaaggtaggcctctgctgtttctttcattttagtcaaaacaaaagcaccaatttgacttggggagtactgctgtccattggcttcaacccatgcatctccgttaggagccctcacaattttgaaaggaaccatcttcatttccttctgcgtctgagggtcatcaaagcgtctaccaatcagacgcttggttccaaagagggtgttggttggatttgtgacagcttgtcgcttagcaggtgtaccgactaatagctctcctttctggctgaaagcaactactgacggagtcgtccgagcaccctcagagttctcaataactttgggattctttccctccatgacggcaacacaagaattcgtagtacccaaatcaataccaatgacatcatttccagcaggttttgtgctgaatggccttgcaagacctgcccacttgctgcctaaaacccatgatgatttggtgtttccagcaaaggttctaaaagcagaaacaggggcagaggaaaattcacggcgacgaagagatctgagaagaacggcggtggcggccattgataagagagagagctgcttcgtaaacctgccggctcctttattgtgcagcagttcctgctagggtttagggtttacgaggaggagctttgaggaggcggattgaag

>comp27414_c1_seq1 heat shock 70 kda mitochondrial-like

caccaaaattagttttaaaaaaaatctaatgttgagttccgcctgggaaaatttctaatccaggaactttcagtacaatttggtaatttaaatacaagactgctaaaaaaaaaaaacacctttatagctcaactggaacaagttcaaatcaacttgccatgtctccaccatctacttctttacctcctcatactctgcctcaggggcctggtcgcctccctgagaacctccagaggaatcaccaccaccaccagaactgccagacatgtgctgtccaatttttgacactgctttgtttgctgcatcaagcttcgccttgatatcatcaatgttctcagtgcccattgcatttctcaaatctgaaacagcagactcaatttctgaaaccacttcacttggaatcttgtccttgtactcatttaagctcttctcaatgctgtagatggtggtgtctgcattgtttctgagatcaatcaacgccttcctttcttgatccttctgggcatgggcctcagcttccttgaccatcttttcaatctcatcctctgataggccgcctgatgaccgtattgtaatctgctgctctttaccggttgtcttgtcttttgcagacacggtaacgattccattggcatcaatgtcaaatgtgacttctatctgaggcatgcccctag

>comp27414_c2_seq1 heat shock 70 kda mitochondrial-like

tacccacaagctcaaactcaccaaggagtttgttatctgctgccatttcacgctccccttggagtactttgatacccacctgggtctgattatcagcagctgttgagaatgactgacttttctttgttggaattgtggtatttctgttgatcaatctggtaaagataccaccaagcgtctcaattccaagggaaagtggtgtgacatcaagtagaagcaactctttgacatcaccacggagaatgccaccctgtatagcagcccccatagcaactgcctcgtctggattcacccctttgcttgggctcttgctgaagatttcagaaactacttcctgaacctttggaacacgcgtcattcctccaacaaggagcacttcatcaacttccttggttgatatgccagcatccttcaaacaactcttgcaaggggccttagtcctctcaatcaagtggttcaccagagcctcaaacttggacctagttaacgttatattaagatgctttgcaccagatgcatcagcagtgatgaagggcaagttgatctcagtctgagatgttgatgaaagctctatctttgccttctcagcagcctctcgaagtctctgcaaagcaagcctgtcttttgacagatcaataccatctgtcctcttaaattcactcaccaaaaattccaataaagcattatcgaaatcctcccctcccaaaaatgtatcgccatttgtcgctttaacctcaaaaacaccattggatatctccaaaatagatatatcaaaagttccacctccaagatcgaaaacagcaacaagaccttctttgttgttcaaaccataggagagtgcagcagcagtaggctcattaataattctttg

>comp27417_c1_seq2 dcd domain protein isoform 1

tctgtcttcttccttggcatacagggtaagcatacatacaacttttccacactccctttgacaaatctacacgctatccctctctttccttgaaatttccccttgtttttctttctcaaatcaatatatattcacacccctcttctctatcttttctccacatttcagttcatacactttttttccccttctctctgcagtgttttactgaaaattttctgatcaagattggatttttctttcatttttcaagaaatggagaacaatcaatcttctttctggcaattcagcgacaatctccgtcttcagaccagcagtctagccaatttgtcgctgaatgattccatctggagcactaaccacacggccaagcgcaccgctgaggagcgtcgtaacttcgacttccggaacggcggcgacgccttcggcggcgccccggttaatgatttgaagtctgatttcaatctcgggttcagcaacgacgtttggaaggctccgattaacgacagttttagtgggaatgttagttactctgggttcgggtcggcctcaaccgggctgaatggcgggtttaacaaggggatttactccaccccttctttgaattttaacagttacagtaaaggtaagggttttgctaataataataatgttatggtgaatgggaaggtgaataagggcggtaaaaatgatgaggaaaattacggcggtaaaggtgggaagaagaacagagggaatagggagagtaataaggataataataacggcggaaatggtgacaacaagacggcgctggataagaggttcaagactctgccgccggcagaggctttgccgaggaacgaaacagttggtggatatatttttgtttgcaacaatgataccatggcagagaatctcaagcggcaactctttggcttgcctccccgctaccgggactctgtccgatcgataacaccaggattgccgctctttctttacaactattcaacccatcagctccatgggatctttgaggctgcaagctttggtggcactaacattgatccctctgcttgggaggacaagaaaaacccgggcgaatcacgctttcctgctcaggttcgtgtgataacaaggaagatctgtgagcctttggaggaagattccttcaggccaattcttcaccactacgatggtcctaagttccgtctcgaactgaacatccctgaggtaaaatctgttctgtttcaggaacatattacaaaagatagcttttcaagaccctcgaaacacagcacttcattaaccttagctaattcaatttttcattttatcatggcaggctctatccctcttggacatttttgctgaaaacaatccttgagcattcagtcccggataaccatattcgcacgaaataacgcgtgaaaggaacaacagatgaactagagattggcgaagtagttatatactggatgtcaaggaaagattgaagaatggaaagcgtagattaatagtcttttgcttgtatataaacacagttatgcaatctagaggagttttgggtggtggagccatactccccttgtaatatggcagaggcaagcatatacgtatacatatataaatatatataagtagataaattatgtttttctgtacaagtaataattggtgctgagaataattgaaactattgtgctctgaaaatcctaagaatttttgtgctggttgtagagacaatgatgcaataattgaaactattgtgctctgaaaatcctaagaatttttgtgctggttgtagagacaatgatgc

>comp27425_c1_seq1 harpin binding protein 1

cactcagcaatggcgtctgtgcttcactcaacgcttcctctctgtcgccatcactcttcatcatcatcaaaaactacttcaacttctgctctctgggcttcccatttttcaagaactcagaaaattcaccgtttttcaacgcttcatcaaaatagaaactttaatatccagaaggggctcgtctgccagtcatcatctagtgaagtcacattcactgagcctcctgagtctgcgccggccaaagatgatcttattgggtccctcaagatcaaattattgagtgctgtttctggtttgaatagaggtcttgctgcaagtgaagatgacctgcggaatgctgatgctgccgcaaaagagcttgaatcagctggaggtccagtcaatctggcagaggaccttgataagctgcaaggcaggtggaaactgatttacagcagtgctttctcatctcggactcttggaggtag

>comp27486_c2_seq1 feronia receptor-like kinase

catgtggcctctctagcaagataatgtggcctctctagtagggtattgcgagacagaatcagacatgattcttgtatatgagcacatggcaaatgggacactctatgaccatcttcatgaagcttccaaggatccccttccgtggaaacaaagacttcaaatctgcattggtgcagcacgcggtgtaagctatattcattccaatgttcagcagacaatgcttcatcgtgacttgaagtcaaccaatatttggttagatgagaactggattcccaaggtttcagaatggggtttgatgaaaaagaaaggaaacaatcaggttccatcaattgtcaagagtaactgggggtgtttggattcagattacatacgtggaggacaattaacagaaaagtcttatgtctactcatttggtctaattttgtttgaggtgctgtttgctgacaaagaatcagattgttggttcgatgaggatcaagtcagtctagcccagtggatcaagtcatgcatgaagagtaacttttctgggtgtattgacccattcctggttgggagaacatcaccagatagcctgaaaatattcatagagacagctggaagatgtttagttgattatggaattgaccgtccatcaataaccgatatagtgacaagacttgaggctgcattaaagcaggaagatgccacagaaggcactaagggtactcaaccccgaaggagctgacctggagcatgtaggtttcctgatcaggcagtaaaataacttgtatggggtaaagcaaagaatgtcagatcaagaatggaacccaaagaaatgaaaacaaatgtcacctatccttatcctatgtaccatatatatatctattgtatatctattggc

>comp27486_c2_seq2 kinase family protein

ctggacaatgtatacaaaggatatatagatggtggccaaaaagcagtagcaatcagacggtcaagagcaagagaatccagactaatcatggcacatgagttacaatcaaagaaagagattcaaatgaaatcttctccaactcaagataatgtggcctctctagtagggtattgcgagacagaatcagacatgattcttgtatatgagcacatggcaaatgggacactctatgaccatcttcatgaagcttccaaggatccccttccgtggaaacaaagacttcaaatctgcattggtgcagcacgcggtgtaagctatattcattccaatgttcagcagacaatgcttcatcgtgacttgaagtcaaccaatatttggttagatgagaactggattcccaaggtttcagaatggggtttgatgaaaaagaaaggaaacaatcaggttccatcaattgtcaagagtaactgggggtgtttggattcagattacatacgtggaggacaattaacagaaaagtcttatgtctactcatttggtctaattttgtttgaggtgctgtttgctgacaaagaatcagattgttggttcgatgaggatcaagtcagtctagcccagtggatcaagtcatgcatgaagagtaacttttctgggtgtattgacccattcctggttgggagaacatcaccagatagcctgaaaatattcatagagacagctggaagatgtttagttgattatggaattgaccgtccatcaataaccgatatagtgacaagacttgaggctgcattaaagcaggaagatgccacagaaggcactaagggtactcaaccccgaaggagctgacctggagcatgtaggtttcctgatcaggcagtaaaataacttgtatggggtaaagcaaagaatgtcagatcaagaatggaacccaaagaaatgaaaacaaatgtcacctatccttatcctatgtaccatatatatatctattgtatatctattggc

>comp27511_c0_seq2 zinc finger a20 and an1 domain-containing stress-associated protein 8 isoform 1

acctttttgggccttctttggcttcctcacttctctcacttgctggtgattcagaagatgtttgtgctgaagccaccatgacctccaatggaacagcctttgattcaggagagagagtaacatctgcacccttccctgcagtgctggagctcccattcacaatactttggatagatgatgcagcaagcttggcttgctcttgcttcagaacaaggtccttgtaacacttggagcacatgttcatggttgctgctgctccaaagaatccacaattgttgacacataaaatgggagcttgaggagtttggcatcctgtctcattctgttccattttggatttttcctgttcgcggaggagagaaaagagaatacctcgtcagaatttccgtctctaatcccctagaagcgaacaggaaatgaagcgagagaaatgagaaagatgagagagagagatttgaaagagtcgaagga

>comp27590_c4_seq1 protein oberon 3-like

agtcaagctcttttatcagggtttctagaccccaatccttcgcacaagacatgaagacctccttaacgaacccaaacatctcagaagcgtgaccgcaaccgaggcagtgaaattgcatctcagttgtacctgaagaccctttcaagcagggaccaggctttatgagatttctttgaatgccacatgctgcatgacaccagtgcgaacaggcatcacagccaacccaactgcaagtgttgcttgcacagtcaaagttcaaacacacaggacacatgcattcattgcaaaatcccttttttgttgagcaaatcttgcaatcacaatcatcaactggtagcaaccttctgcaatttatgtttcggcacctttccagcaagaaaatatccactaattctgttggtggaagtcggtttttgctatataaaaagcttccgagccccattttaattgcaactaaaatttcaagttggctcttttggcactttgagagagtctcctttgtaagatcggatctcctttcgagtctattccggagactcaccaattcatctttcctttctggattatcaatgatgttctttaaatatgctttaacagattcaactgtttcatcagtgagctcctgaattatctgggccatggcaggaattgactcagaaacaatttctgtaagtatcctctctggtcttgaaagcttacgcactttaaccgcatctatatcttctgaatctctcaagttttccaaattcatcctccttgaatctcccaactgagcatctatttttggtcttgctggcaactcagacgggaaaaacgaaatattgtccgagcttgtagtcctataaagactgttattacatgaatccttatcgaacccactgcgatgacccatcccaaatcctcctccgccgccatgattggagaatgcaacattcccatctccaa

>comp27597_c1_seq1 protein early responsive to dehydration 15-like

caggtcattgctctttacgtgtaaactccaaattggcgttgaagacaaggagaaattttgcctgcaagcaatctagtcatggctctagtatccggtggaaggtcaacgctcaatccgaacgcgccgcttttcattccagcggctctgaggcaggtggaggatttctcaccggagtggtgggatcttgtgaccacttccacctggttcagagactactggctcagccagcaccagggagaagatattttcggtgaggtggaaggtggtcttgatgccaacaatgtcgtcgggttgttgcctgatagtattgatcttgatgtggatgaggatatcttgaacatggaggcacagtttgaggaatttcttcaatcatccgaagctgattatggtattaaggcaggcagagttatggaaaatggttttgataagtattccaatacactagtcatgaacgtaaacgtgccaacgcagggagggttcaagtcgccaagggaaccgatgatgaagtactgggagaagccagccaaagcagggagg

>comp27643_c0_seq1 zinc finger an1 domain-containing stress-associated protein 12-like

aagcttttccaaatttaggaagacattgccaacactccgattgccatcaactcgatttcctccccttcacctgtaaggcctgtctaaaggtactgtttccctaatttgatcgacattgtttaatgtttatttcaccgattttaatcggatttctttatcgaatcaaaattgaatattaatttacctgatgttgtgtgtttgcaggtgttctgtttagaacaccgatcgtataaatcgcacgattgtccgaagtccgacgtcggcagccgtaaagtcctggtttgtgaaatttgctccaccgcgatcgagaccacaggatacgacggagaggatgagaagaaaattctggagaggcacgaaaagtcgggagattgtgatccgaagaagaagaaaaaaccgacatgccctgtccggcggtgcaaggaggttctaacgttctcgaacacggcgacgtgcaagaattgtcaaattaagatttgcttgaagcataggtttccggcggatcacgcctgcgagcagctaagatcgtcgtcgtctttgtcttcggctgcttctaataacaagttcttggttgctttggctgcaaggagcgggaaagattgtggaaacaagattgctgctcctagatcgcagcctagtgttaaagcctgttgattatttccatgttatacacaactattttttccgggattaattaataaaatatgaagttcttacattcaaaaagcattatgctatgtgtttctctatatcgcatctaactggatttcacaatcaatgtacaaaaaccataacacattttgcgttggtaaactacga

>comp27643_c0_seq2 zinc finger an1 domain-containing stress-associated protein 12-like

aagcttttccaaatttaggaagacattgccaacactccgattgccatcaactcgatttcctccccttcacctgtaaggcctgtctaaaggtgttttgtttagaacaccgatcgtataaatcgcacgattgtccgaagtccgacgtcggcagccgtaaagtcctggtttgtgaaatttgctccaccgcgatcgagaccacaggatacgacggagaggatgagaagaaaattctggagaggcacgaaaagtcgggagattgtgatccgaagaagaagaaaaaaaccgacatgccctgtccggcggtgcaaggaggttctaacgttctcgaacacggcgacgtgcaagaattgtcaaattaagatttgcttgaagcataggtttccggcggatcacgcctgcgagcagctaagatcgtcgtcgtctttgtcttcggctgcttctaataacaagttcttggttgctttggctgcaaggagcgggaaagattgtggaaacaagattgctgctcctagatcgcagcctagtgttaaagcctgttgattatttccatgttatacacaactattttttccgggattaattaataaaatatgaagttcttacattcaaaaagcattatgctatgtgtttctctatatcgcatctaactggatttcacaatcaatgtacaaaaaccataacacattttgcgttggtaaactacga

>comp27669_c6_seq1 dna repair protein uvh3-like isoform x1

aatcgcatctgaagccaatagagaatttattttctcctcatcatttaccgggaacaaaggagcccttacatctgttggacaagaaagaaatggggctgaagaaagtcagccaccaccagtaaattcttccaccaatgccgtgaataaagctcagcagaccaaaagatctgatgctgcagctggatcagctgtgactgaaactggacgggcatttcatgatgaagttgagacatatctggatgagaggggtcgtgttcgagtcagtagggtaagagcacttgggattcgtatgactcgagatctgcagaggaatctagatttgatgaaggagattgatctggagaaagctgaaagaaatcaggaagaaaataatgaatctgccaatgctaggaaactggttgatgttccagacaattcatctgccagaatccagcatcgagaagctactgataagaatgatggaatgaatactgaagttgataaaactgaggaacctgcagtagcaaatggagcctctattgagatttccttcgaagatacatcagagcatcgtgaatatgatgacaacgatgatgagttatttgctcatctcgtggctggagaaccagtaatggacttctcttttgataagtctgccacatcgaaacaatctttccattcttcttcagataacgagtgggaggaaggagtcattgaaggcaagagtacagactatctctctgaaggaggcacgaatgatgaggga

>comp27673_c4_seq2 hva22-like protein a-like

tgtttgaaattaaatatttcaattaagctaagtaaaccctacgaaagtttcatttgcaacccataaaattacattaagaaatacaacagcacatctacctccgaaaaaaaaaaaaaaaagagagagaaaatattgatcttgtgtggcaagtttgaagatgggtggtggatctggagccgcgagcttcctcaaagtgctggctaacaatttcgacgttcttgctgggcctgtggttagtctggtttatcctctatacgcatctattagggcaatcgagacgaagtcgcctgttgatgatcagcaatggcttacttattgggttctttactctatgattactctttttgagcttacttttgctaaacttattgaatggtaagtttcttttagtttcgtacagttttcgtttcagacaccatggctgtttgatttttcggttccgatgctattaatagctggacttgatcgaactgttgtatctgtatcccttgcatattgtgttgttgctggttgggctcgacttagcttgacccttttttttttctttggaaactatacgtattttttaaatcctggtgtatgtttacattttcttgaggaacttaagtaagttttgctttggatgtcttttgcttgcctgaatttagtttgtaaaaagtgcttggggattatactttgagaactttcggaaaatagtttgaggaacgagacgttccttttaattctctgaagctagtttgaatttgactggggtgcattgtcgatttggttaaatgtgagctattggtggggctgagtaagattttctgttgaatattggcttggggtacctctgtggagagccaaaggtgacacatgttctgtactcataatgacaataaagcagatggtaagaatgaaatttgttaact

>comp27703_c4_seq1 zinc finger protein constans-like 5-like

cgcagacctcgcacatccacacacgctcgtgtttcagacaggctaaattgtcgctagcattatgcacctttgcgtcacaagctatgcacatgaaagcagactccgcacggcagaagagaagcgcggcagcggacttgcagtagtcgcaaggcttcgccgtcacattcaagcccactgggaaaaacttggctctagcagcgccaccaccgtcttgtgccattctcattttctctttctaaactttggtttctctctgcactccaagtagtcaattgtataagcctggcttctctccagtaatattggtgactaggcttaactagtg

>comp27709_c2_seq1 3-ketoacyl- synthase 11-like

acccaacccggacctctccaagatcttcttctgaaaattcaaattctcctgagtgaaaatccctgcctgctttgatctctccataaacagttccttactacacataacctccggcgtaggcttgtaacacgcgaaatcgaccaagtaaactttcctcggccttgtcatgaaatacagcgtgcctaaaaacaccataagggcagaacaaacaacaaccgagacaagattgaacttcagctggtcccataacaggactagatcctctgtcgtaagcatcgaaagttgaaccgagataatccctaacaaaggaactaacaataaatacatggcatgtgagatcaagtaatggtaaccaagcttaacatatttgagcctgacagactggtgaaagttgggaagattgttactccctcgcctctcgggtgtggcctccaccg

>comp27736_c1_seq1 zinc induced facilitator-like 1 protein

attataggctctgccacaattgttgttttcaacactctatttggccttagtataaacttttggatggccatctcaatgaggttccttcttggaagtttgaatggcgtacttggaccaataaaggcatatgcatgtgaaacagttcgtgaggagtaccagtctttaggattgtcagcggttagtacagcatggggtacaggattaattgttggaccagctttgggaggctttcttgct

>comp27744_c0_seq1 calmodulin-binding heat-shock

tggccgttccagatgcttttccaccttctcattatggaaccttcgatgaaaaagacaatgatgactcccaccactcaattagcgattcttccacaggatcatcgacaaaaagcaagactaaagaaagctgggatgaactgatcgaacgcctttttgagagggatgactctggtctcatggtactgaagaaacctctcgttgctgatcgaaattgatgcatcagatcaagtcattgttcatttgttcagttgtggtgcagtattctttccctggagttaccaatgtttcttgtatctatctgtgagtgacttgatatacgaatagaacataattgtcaagagttgccattatcaagtattacccttttaagtttaagatggattttgctaaaatagcagtgaatagtcatagaattagtgttgctataatacatggatgaatttgtgattttagtgtacttcgtattaaatcaaaatggtg

>comp27744_c1_seq1 calmodulin-binding heat shock protein

agacatgggcccttgccacagctgaagagttcgaacctgtccctaggctatgccggtatattctggctgtctatgaggatgacctcagacagccactttgggagcctcatcgagggtatggaattgatccagattgtttaattatcaaacgaagttataaggatacacaagggaaagtgcccccatatttattgtaccttgatcatgaccatgccgatattgttcttgctataaggggccttaatttggccaaggagagtgactatgcagttttgctggataataagcttgggaaaaggaaatttgatgggggttatgttcacaatggcctgttgaaagcagctgggtgtattttggatgcagaatgtgagattctgaaggaagtggttgaaaagtatccaaattacacattgactttcgcaggacattccctggggtcaggtgtggcagcattattgacaatggttgtggtgcaaaatcgtgataggctggggaatattgacaggaagaggctaagatgctatgctattgcacctgcaagatgcatgtcccttaatttggctgtgagatatgccgatgtcatcaattcagttgttctgcaggatgatttcttgcctcgaacagccacccctttggaagacatcttcaagtcactattctgtttgccatgcctattatgcctaagatgcatgagagatacatgtatatcagaggagaggatgctcaaagatccaaggaggctgtatgtaccaggtcgcctttatcacattgttgagagaaaacctttcagatgcggaagattccctccagttgtgaggaatgcagtgcctgtggatggaagatttgagcatatagttctttcttgtaatgccacttctgatcatgccattatttggatagagagagaagccgaaatggctttggaattgatgcgagagaaagatgatgccatggagattcctgcaaaacagaggatggagaggcagaagacattagataaagagcacagag

>comp27744_c1_seq2 calmodulin-binding heat shock protein

agacatgggcccttgccacagctgaagagttcgaacctgtccctaggctatgccggtatattctggctgtctatgaggatgacctcagacagccactttgggagcctcatcgagggtatggaattgatccagattgtttaattatcaaacgaagttataaggatacacaagggaaagtgcccccatatttattgtaccttgatcatgaccatgccgatattgttcttgctataaggggccttaatttggccaaggagagtgactatgcagttttgctggataataagttggggaaaaggaaatttgatggagggtatgttcacaatgggctgttgaaagcggctgcgtgtgttttaaatgccgagtgtaacattttgaaggaattggttgaaaaatacccaaattatagactgacatttacagggcattccctaggctctggtgtggcagcattgttgactatggtggtggtgcaaaatcgcaatagattggggaatattgaaaggaagaggatcaggtgctatgccattgcacctgccaggtgcatgtcactaaatctggctttgagatatgctgatgtaatcaactcggttgttcttcaggatgattttctgcctcgaacagccaccccattggaagatatattcaagtctcttttctgtttgccatgcttattatgcctaaggtgcatgagagatacatgcataccagaggacaaaatgctcaaagatccgagaaggttatatgcacctggtcgcctttatcatattgttgagcgaaaacctttcagatgtggaagatttcctccagttgtgaggacagcagtcccggtggatggaagatttgagcatatagtgctgtcttgtaacgccacctctgaccacggcattatctggatagagagagaagctgaaagggctttgcaattgatgcgagagagagatcatgcgatggagattccagcaaagcaaaagatggagcgacaggagtcgttagccaaagaacacagtgaggagtaccaggctgctctacgtagggctgtttctttagatgttccccatgccttttcaccttctcagtacggaacctttgatgaaaaagatgatgaagattcccagcaatcagtaggagattgttcgactggatcatcggccagaagcaagggtcgagaaagctgggacgagctgatcgagcgtcttttcgagagggacgagtctggtcacatgatgcttaaaaaacccacccattgttgacttatgtgctgtggtaacgcatcggatccaagcatagcatcattcttttattgaattctgcttattctttataagattatgtagt

>comp27744_c1_seq3 calmodulin-binding heat shock protein

agacatgggcccttgccacagctgaagagttcgaacctgtccctaggctatgccggtatattctggctgtctatgaggatgacctcagacagccactttgggagcctcatcgagggtatggaattgatccagattgtttaattatcaaacgaagttataaggatacacaagggaaagtgcccccatatttattgtaccttgatcatgaccatgccgatattgttcttgctataaggggccttaatttggccaaggagagtgactatgcagttttgctggataataagttggggaaaaggaaatttgatggagggtatgttcacaatgggctgttgaaagcggctgcgtgtgttttaaatgccgagtgtaacattttgaaggaattggttgaaaaatacccaaattatagactgacatttacagggcattccctaggctctggtgtggcagcattgttgactatggtggtggtgcaaaatcgcaatagattggggaatattgaaaggaagaggatcaggtgctatgccattgcacctgccaggtgcatgtcactaaatctggctttgagatatgctgatgtaatcaactcggttgttcttcaggcaagttgctacatagtttctgtgtgcttcttatgacttatgaattaca

>comp27744_c1_seq4 lipase class 3 family protein

agacatgggcccttgccacagctgaagagttcgaacctgtccctaggctatgccggtatattctggctgtctatgaggatgacctcagacagccactttgggagcctcatcgagggtatggaattgatccagattgcatagttatgaaaaagaattacgaggatacacaggggcgagcaccaccatacttgttatatcttgatcatgatcatgcagatatagttcttgccataaggggccttaatt

>comp27748_c0_seq1 nogo-b receptor-like

tgcaatctactcaatttttttctgtccttcttttgacaattttagagggagagagaatgacattggttagtggtagaaaactcatgggttttgtcttcattttatcttaggctttttcgcaactcagatatgctggagctcgacttcgttcgtttggtcatgttccttcgcagggtggaatcaacgattttggatgtaggggatgaagtgcataaggtctacgcaaagatctggtcgagtggcaatctcgggcttcaattgctctggcacattatgcatctgatggtgagcatgtggtactttgtcctgggattgataaatgcccttgagagcttccttatctccagtggtttcttcaagcagtataaagatcttgatattagtaaggttaagtacctggctgttgttatagacagtgaagaagctcttcacactttgaatgttcttcaactattgaggtgccttgcagctcttggattgaaaaatgtatgcctctacgatgcagaaggtgtgctgaagaaatccaaagaagctctcactctatggttgaaaagtgaaagactgacaaatgaaactaccaaggatccgcttcttgagcagaaatatatgagcttggaagtgatatcttttactgatgggaaacatgcagttgcaaaagctgctaatattcttctaaagaagcattatttgagtgccaatcctgagaagccagagttgacagagtctgatatgatagatgctcttgcagcattaggatatggggcaccagaacctgatctgatgttgatttatggacctgcaagatgccacctgggttttccagcatggagacttcgttacacggagattgtgcacatgggaccgctaaagtccatgaaatttggtaaccttataaaagccatccgtaggtacactatggtgaatcagaactatggttcatgaactggtatacctgaattagcattttcttatatgccgtgtgcaagaataaatacttgttgccccgatgacatctgcagcaaggctctaaattatgtattgtagttttggagagtgtggttttgtcaaactttaaatacaaatgctgtaactattcaatagtgtgtggttttgtcaaactttaaatacaaatgctgtaactattcaat

>comp27748_c0_seq2 nogo-b receptor-like

agagagagagagagagaggagagaaaaaaggctccttcctctcattcgcgctcacagtggaagggagggtaaaattcatgcagtgcaagtgttgttgaagcaggcgcaaactacgtgctttttcgcaactcagatatgctggagctcgacttcgttcgtttggtcatgttccttcgcagggtggaatcaacgattttggatgtaggggatgaagtgcataaggtctacgcaaagatctggtcgagtggcaatctcgggcttcaattgctctggcacattatgcatctgatggtgagcatgtggtactttgtcctgggattgataaatgcccttgagagcttccttatctccagtggtttcttcaagcagtataaagatcttgatattagtaaggttaagtacctggctgttgttatagacagtgaagaagctcttcacactttgaatgttcttcaactattgaggtgccttgcagctcttggattgaaaaatgtatgcctctacgatgcagaaggtgtgctgaagaaatccaaagaagctctcactctatggttgaaaagtgaaagactgacaaatgaaactaccaaggatccgcttcttgagcagaaatatatgagcttggaagtgatatcttttactgatgggaaacatgcagttgcaaaagctgctaatattcttctaaagaagcattatttgagtgccaatcctgagaagccagagttgacagagtctgatatgatagatgctcttgcagcattaggatatggggcaccagaacctgatctgatgttgatttatggacctgcaagatgccacctgggttttccagcatggagacttcgttacacggagattgtgcacatgggaccgctaaagtccatgaaatttggtaaccttataaaagccatccgtaggtacactatggtgaatcagaactatggttcatgaactggtatacctgaattagcattttcttatatgccgtgtgcaagaataaatacttgttgccccgatgacatctgcagcaaggctctaaattatgtattgtagttttggagtgtgtggttttgtcaaactttaaatacaaatgctgtaactattcaatagtgtgtggttttgtcaaactttaaatacaaatgctgtaactattcaat

>comp27748_c0_seq3 nogo-b receptor-like

agagagagagagagagaggagagaaaaaaggctccttcctctcattcgcgctcacagtggaagggagggtaaaattcatgcagtgcaagtgttgttgaagcaggcgcaaactacgtgctttttcgcaactcagatatgctggagctcgacttcgttcgtttggtcatgttccttcgcagggtggaatcaacgattttggatgtaggggatgaagtgcataaggtctacgcaaagatctggtcgagtggcaatctcgggcttcaattgctctggcacattatgcatctgatggtgagcatgtggtactttgtcctgggattgataaatgcccttgagagcttccttatctccagtggtttcttcaagcagtataaagatcttgatattagtaaggttaagtacctggctgttgttatagacagtgaagaagctcttcacactttgaatgttcttcaactattgaggtgccttgcagctcttggattgaaaaatgtatgcctctacgatgcagaaggtgtgctgaagaaatccaaagaagctctcactctatggttgaaaagtgaaagactgacaaatgaaactaccaaggatccgcttcttgagcagaaatatatgagcttggaagtgatatcttttactgatgggaaacatgcagttgcaaaagctgctaatattcttctaaagaagcattatttgagtgccaatcctgagaagccagagttgacagagtctgatatgatagatgctcttgcagcattaggatatggggcaccagaacctgatctgatgttgatttatggacctgcaagatgccacctgggttttccagcatggagacttcgttacacggagattgtgcacatgggaccgctaaagtccatgaaatttggtaaccttataaaagccatccgtaggtacactatggtgaatcagaactatggttcatgaactggtatacctgaattagcattttcttatatgccgtgtgcaagaataaatacttgttgccccgatgacatctgcagcaaggctctaaattatgtattgtagttttggagagtgtggttttgtcaaactttaaatacaaatgctgtaactattcaatagtgtgtggttttgtcaaactttaaatacaaatgctgtaactattcaat

>comp27748_c0_seq4 nogo-b receptor-like

tgcaatctactcaatttttttctgtccttcttttgacaattttagagggagagagaatgacattggttagtggtagaaaactcatgggttttgtcttcattttatcttaggctttttcgcaactcagatatgctggagctcgacttcgttcgtttggtcatgttccttcgcagggtggaatcaacgattttggatgtaggggatgaagtgcataaggtctacgcaaagatctggtcgagtggcaatctcgggcttcaattgctctggcacattatgcatctgatggtgagcatgtggtactttgtcctgggattgataaatgcccttgagagcttccttatctccagtggtttcttcaagcagtataaagatcttgatattagtaaggttaagtacctggctgttgttatagacagtgaagaagctcttcacactttgaatgttcttcaactattgaggtgccttgcagctcttggattgaaaaatgtatgcctctacgatgcagaaggtgtgctgaagaaatccaaagaagctctcactctatggttgaaaagtgaaagactgacaaatgaaactaccaaggatccgcttcttgagcagaaatatatgagcttggaagtgatatcttttactgatgggaaacatgcagttgcaaaagctgctaatattcttctaaagaagcattatttgagtgccaatcctgagaagccagagttgacagagtctgatatgatagatgctcttgcagcattaggatatggggcaccagaacctgatctgatgttgatttatggacctgcaagatgccacctgggttttccagcatggagacttcgttacacggagattgtgcacatgggaccgctaaagtccatgaaatttggtaaccttataaaagccatccgtaggtacactatggtgaatcagaactatggttcatgaactggtatacctgaattagcattttcttatatgccgtgtgcaagaataaatacttgttgccccgatgacatctgcagcaaggctctaaattatgtattgtagttttggagtgtgtggttttgtcaaactttaaatacaaatgctgtaactattcaatagtgtgtggttttgtcaaactttaaatacaaatgctgtaactattcaat

>comp27847_c0_seq1 alpha-glucan h isozyme

tgaggaggaagttgcagaagattggttggagaagttcagtccctgggaagttgttagacacgatgttgtcttccctataagattttttggtcaagttgaggtccatccctctggctcccggaaatgggtcagtggagaggtcatacaagctgtagcatatgacgtaccaattcctggatacaaaacaaagaacactaacagtcttcgtctgtgggaggcaaaagctagtgctgaggactttaacttatttcagttcaatgatggacaatatgaatctgcggcttcacttcattcaagggctcagcagatttgtgccattctctatcctggggatgctactgagaatgggaaacttttacgactgaagcaacagtttttcctatgtagtgcatcacttcaggacatcattgccagatttaaggaaaggaaaggtgaaaaggaaaaaatccagtggtctgaattcccctctaaggttgctgtacaactgaatgacacacatcctactcttgcaataccagagttg

>comp27847_c1_seq1 alpha-glucan phosphorylase 2

tcaacgttagccccatctagggttccaacaatgaggcatccattcagagcaaatttcatgttacttgtgccactagcctccattccagctgtactaatatgttgtgataactcacttccaggaataagcacttctgccacagacacattgtaattggggacaaaaacaacctttaaaaagctattgacttctggatcagtgttaacgacagcaccaacatcatttacaagcttaactattcttttagcatttgtatatgttgcaaatgcttttccaccaatcatgatagtacgaggtgttgtcttttttcggtcttcagggctcatctcctttaatttcttgtacctataaacagcagccaaaatgtttaacagctgcctcttgtattcatggatgcgtttaacttgaatatcaaaaagtgagctagggtcaatgctaacaccagtaacctgaagtatgtagtttgccaaacgttgtttgcttgccaacttagctaattcccactcgctttggagttctggattgtcagcaaattgtcgtagatttgctagtaggtcaagattagtcacccattgatctgtttttagccatttggtgataatatgactaagctcggggttgcaaaacctgagccatcggcggggagttacaccattggttttgttttgaaatttggcaggccatatagagacataatcagcaaacaactcagactttaagatgtcactatgcagctgtgcaacaccattcactgcatgtgaggacaccacacacaagttggccatacgaacaactggtttttgtgggttgttatccaaaatacgtatatcagcaagttttccctcgaggtcaggtctggttgactgtataatttggataaacctcttgtcgatttcttcaatgatttccatatggcgtggaagaagcttccacatgacagcttgtgaccatttctctagggcctcaggaaggactgtatggtttgtataggctattgtcctgttggtgatatcccaagcttcatcccatccaaggaaatcattgg

>comp27847_c2_seq1 alpha-glucan h isozyme

gttctaggatttgcatctatttctgatcgttctactgaaagagcagcagaaaatatacaagtttacggactttattacataaaaagtatctgtccacgtgttcgacattatttgtgttacataaatgcctgaaagaaagcaccaaatgtaaaaggataaacatttatcactttggattgatttcttgggtatacctgtcttcttgctagcagattatgatgtatatgctcggaacacttccatgatgtgtccagaatgtgatgcatatggtcaaaacacttacatgattaaggcacacggcactcgctcattttccaaatttcacttgcatactgggctatggttcgatcactgctaaatttcccactgccagcagtgctaagtatcgacatctttatccatctctttctgtccttgtaagcttgatctaccctttcttgggcatccatgtagcttgggaagtcatagccaacaagaaaataatcaccacggccatagccagaattgccctcaagtgactcaagcagaggattatagtcatagctgccaaatgctccagatcttataaactgtttggcctcttcaaatcgagggtctgccttaaactacagagtaaaggaaaaaagagaggccattttggaataagctttgaagaccaaaggtaggcagaaaggcacttgttaatgaaacagatgtgtagttttatcaaggaacaatgtttttggtgttgcataattgataagcacccacgtgatatgaacatcctgatttcctgtttaagctttagtttctattttgatacttcaaaccatttgaaacaaatttagtggtcagcaatggttagttcatctctcctttcacctctgtttagtagtttaccaaattttttccttttaataaagtaagctgtagggttttagacaagttaatcagactgtacagcaataatctttagtaggtttatggcagagcttaatcttgtaacatgctatatagtgttgcaatgtgtaaggtacacatagatagtaagatctggtataacggacatataaagatttac

>comp27847_c2_seq2 alpha-glucan h isozyme-like

gttctaggatttgcatctatttctgatcgttctactgaaagagcagcagaaaatatacaagtttacggactttattacataaaaagtatctgtccacgtgttcgacattatttgtgttacataaatgcctgaaagaaagcaccaaatgtaaaaggataaacatttatcactttggattgatttcttgggtatacctgtcttcttgctagcagattatgatgtatatgctcggaacacttccatgatgtgtccagaatgtgatgcatatggtcaaaacacttacatgattaaggcacacggcactcgctcattttccaaatttcacttgcatactgggctatggttcgatcactgctaaatttcccactgccagcagtgctaagtatcgacatctttatccatctctttctgtccttgtaagcttgatctaccctttcttgggcatccatgtagcttgggaagtcatagccaacaagaaaataatcaccacggccatagccagaattgccctcaagtgactcaagcagaggattatagtcatagctgccaaatgctccagatcttataaactgtttggcctcttcaaatcgagggtctgccttaaacagcccttgctctctttcgttgcgcaaccgaggaacttcatcagctgtggcaccaaataaaaagaaatttgtttctccaatttcttctctgatttcaacgttagcccca

>comp27847_c3_seq1 alpha-glucan h isozyme-like

tgatttgttgcttgaatagaccatatctgtacctcagaccatatccccatgcaggtaagttcaaggtagccattgagtcaaggaaacaagaagcaagcctcccgagaccaccatttccaagtgcagcatctttctcctgctccactatgtcctcaagttcatgacccaattgctttaaagcatcagcataagcatcttggacatctaggtttccaactgcattggtcagagctcgaccttggagatattccatggatagatagtatgtttgcttcggattgactttgtgatagtgactataagtctcattccattgcttgatcagctgatcacgaacgctttctgcagtagcatagaacgcttgctccggctcaaacttgaaaggggaaaagtgggggctgtactgagcatggtaattgatattggacgcaatttcagtcggctctttggccagcggatgtgcaactggtggaattttttctgaaattttagccttagcagttgcagagccgttggccttcgctgtagctgccatgcctacaagtttcaccagcctctacagatgcaccacctgatacacgctccgaatttgaacggaaaacaataatgcaatttagtttgtgctctaaaccatacacaaatttgagatatttaaggagtggaatgagagaattataccggtaagatatttttcggaagagaaaaatgacaaaaaaggcgaaaagcaaaaaggctttctctacggctgttagtgttttgtggccatgagtgattgattgaaagatgatgatggcgagagtag

>comp27847_c3_seq2 alpha-glucan h isozyme-like

tgatttgttgcttgaatagaccatatctgtacctcagaccatatccccatgcaggtaagttcaaggtagccattgagtcaaggaaacaagaagcaagcctcccgagaccaccatttccaagtgcagcatctttctcctgctccactatgtcctcaagttcatgacccaattgctttaaagcatcagcataagcatcttggacatctaggtttccaactgcattggtcagagctcgaccttggagatattccatggatagatagtatgtttgcttcggattgactttgtgatagtgactataagtctcattccattgcttgatcagctgatcacgaacgctttctgcagtagcatagaacgcttgctccggctcaaacttgaaaggggaaaagtgggggctgtactgagcatggtaattgatattggacgcaatttcagtcggctctttggccagcggatgtgcaactggtggaattttttctgaaattttagccttagcagttgcagagccgttggccttcgctgtagctgccatgcctacaagtttcaccagcctctacagatgcaccaccggtaagatatttttcggaagagaaaaatgacaaaaaaggcgaaaagcaaaaaggctttctctacggctgttagtgttttgtggccatgagtgattgattgaaagatgatgatggcgagagtag

>comp27856_c0_seq1 extra-large g-protein

tctagtaccatatttaaacaggccaagatagtatataatgtcccattctccaaagatgagaaacaaaatatcaaatttgtgatccaaagaaatgtctacagctatatctgtattctgcttgaggggcgtgcacgttttgaagaagattatttgattgaaatgaggagagaacttgctgataaacctggtccttcagggatttttgagcagctcaacgaaagcaatatttatttacttagcccaaagctgaaggcattctcagattggctactccaaattatgatgtccggcaacttggaggtcatattcccggctgctacgcagatgtattcacctttggttgaggagttgtggaaggataaagcttttcaagccacttacaaacggagaaatgaactgcgtatgctgcctagagtagccaactatttcttagaccgcgtaagttttgctgtatgttcatttctataaactgcttcttcatatgttggatacttataagcgcctccatctttcaaaaggctgttgaaattacacaagtagactacgagccttctcaaatggacatcttgtatgcggagggaataacgtcttccaatggggttgcgtcaatggatttttctttccccaagttgtctcaagattgctacatggaatcttcggatcacaaggatcccccagtaaggttggttctctaatcttgcaccactcgtctcatttactggacgcgcattcctttatgttatcgacttgtttataaacacttgatatgaccatagatgcattatgatacatcttcctgcagctatcaactgattagagttcattcgagcagcctcggagaaaactgcaagtggctagagatgtttgaggacataaacctagtcatatactgtgtttcgttgacagattatgatgaattctatgaagacataaatggagttcggacaaacaagatgctggcaaccaggagactcttcgaaagcattgtcactcatccaactctggccgacaaagattttctcctcatcctta

>comp27856_c0_seq10 extra-large g-protein

tctagtaccatatttaaacaggccaagatagtatataatgtcccattctccaaagatgagaaacaaaatatcaaatttgtgatccaaagaaatgtctacagctatatctgtattctgcttgaggggcgtgcacgttttgaagaagattatttgattgaaatgaggagagaacttgctgataaacctggtccttcagggatttttgagcagctcaacgaaagcaatatttatttacttagcccaaagctgaaggcattctcagattggctactccaaattatgatgtccggcaacttggaggtcatattcccggctgctacgcagatgtattcacctttggttgaggagttgtggaaggataaagcttttcaagccacttacaaacggagaaatgaactgcgtatgctgcctagagtagccaactatttcttagaccgcgctgttgaaattacacaagtagactacgagccttctcaaatggacatcttgtatgcggagggaataacgtcttccaatggggttgcgtcaatggatttttctttccccaagttgtctcaagattgctacatggaatcttcggatcacaaggatcccccagtaagctatcaactgattagagttcattcgagcagcctcggagaaaactgcaagtggctagagatgtttgaggacataaacctagtcatatactgtgtttcgttgacagattatgatgaattctatgaagacataaatggagttcggacaaacaagatgctggcaaccaggagactcttcgaaagcattgtcactcatccaactctggccgacaaagattttctcctcatcctta

>comp27856_c0_seq12 extra-large g-protein

tctagtaccatatttaaacaggccaagatagtatataatgtcccattctccaaagatgagaaacaaaatatcaaatttgtgatccaaagaaatgtctacagctatatctgtattctgcttgaggggcgtgcacgttttgaagaagattatttgattgaaatgaggagagaacttgctgataaacctggtccttcagggatttttgagcagctcaacgaaagcaatatttatttacttagcccaaagctgaaggcattctcagattggctactccaaattatgatgtccggcaacttggaggtcatattcccggctgctacgcagatgtattcacctttggttgaggagttgtggaaggataaagcttttcaagccacttacaaacggagaaatgaactgcgtatgctgcctagagtagccaactatttcttagaccgcgtaagttttgctgtatgttcatttctataaactgcttcttcatatgttggatacttataagcgcctccatctttcaaaaggctgttgaaattacacaagtagactacgagccttctcaaatggacatcttgtatgcggagggaataacgtcttccaatggggttgcgtcaatggatttttctttccccaagttgtctcaagattgctacatggaatcttcggatcacaaggatcccccagtaagctatcaactgattagagttcattcgagcagcctcggagaaaactgcaagtggctagagatgtttgaggacataaacctagtcatatactgtgtttcgttgacagattatgatgaattctatgaagacataaatggagttcggacaaacaagatgctggcaaccaggagactcttcgaaagcattgtcactcatccaactctggccgacaaagattttctcctcatcctta

>comp27872_c0_seq1 carnitinyl- dehydratase-like

atattgatccaaaaatcacattctttccaacaaatacaaataattaacttttatttatttttcaattcttattatagttttttcatgtaatttctcagagcttagaaggcaagacggtctcctcctccaaccctagcaaccggcaaacctccggatacaaagccttcctaatcccggcatacgcttccccgctccacctcctctccgccagctcctccgccgtccgcttcgccgcagccaccacctcttccacgctatcatacgcggagtcaacaatccccattcccaccgccgcctccgccccaaccttcgccgcgcgcagcaccagctgtcgacgagccgcgctcgaaccgattttccccttaatcaaagccgtgaagtaatgaggcaaagtcatacctatatccagctcgctcatgtacatcactcccctcgacgacgtcatcacgacgtaatcgtggctcatggctaggattagcccggctgcggcggcgtgcccggtgacggcggctatagtcggcatggggagggaaagaagatcggcgacaactcctttgaagagatccaccatccggaggagctccgccttcgcggcgtcggtcgagccggctctcgcgccgacggcttgcgcgtgtctcaagtcgaagccgttggagaagaatctgccctcggattgcgtaatgagagccgatccattgacggcttgggctttggcctccgccaaagcggcgcggattgatgaaatgagcggcggatttaagcggtgctcctgatcttttttggtgtcgcctttcaacgtgaggatgaaaagattaccgcgcttctctaacgtgcacatggtgtatagtgggtgatcgataaacagttgcgttgggaggaaagaagaagatgaagacgaaggcggggggggagccaaaaaaaaaaaaaaaaaaaa

>comp27879_c1_seq2 transcription factor bee 3-like

ttcttgttttgaaatgatcaaaacttgataaaattctgatctgtttcccaattcagtttctttctatgaagctcacagcagcaagcacgttttacgacttcaactctgagacggatgccatggaggcaatacagagagcaaaggcatttgaagcgttaaagattcaaaatggagccagccctggacttccttctgctcattttgctccatcaggcctcaattctgaatactatcctcaatttccacaaaacagatgaaaagattccaaaactttcccacttttttttctcagttcaatttgtaccactcaagagcaaagtaaaggcaaatgtcaaaaagaaataaatatactcaattgtatcagttcacacagatgttggaatttaa

>comp27879_c1_seq4 transcription factor bee 3-like

gtcggtaatccgtcaagtagagtttctttctatgaagctcacagcagcaagcacgttttacgacttcaactctgagacggatgccatggaggcaatacagagagcaaaggcatttgaagcgttaaagattcaaaatggagccagccctggacttccttctgctcattttgctccatcaggcctcaattctgaatactatcctcaatttccacaaaacagatgaaaagattccaaaactttcccacttttttttctcagttcaatttgtaccactcaagagcaaagtaaaggcaaatgtcaaaaagaaataaatatactcaattgtatcagttcacacagatgttggaatttaa

>comp27902_c1_seq1 mediator of rna polymerase ii transcription subunit 8-like isoform x2

gtctggtttctgcaagtataggaatgttaacatggttttatatattctgaaagaaatgattgatacaaatattgcagtagactctaaagcattcaaatcgatgcaaaaacttctgggtgactctcccaagtctcttttagctaatctcttttttgagttgagaaattcggggctgttgcctgaagtggagatgttgatggtcgatggacatgatactagtgcctatgccagtttcagttcatcgaatgattactggaatgttcttgaccttggcacatccggcattgacgagctatccaatgctgttgtttcagtgggctgactgttgagtaaagctagccgttaacatgcgatcattgttgaagctctctttgatgtttcatctcgaagaaaccaacactaaattgcatcttcacgtcttttggtcgtcgaagattcacatttcccttgattattgggctacagctttctgcaaactgaagtgaaacaatagttgcaatcaagtgaatgttcatatattagattatccagattccatttttcgtgtcggcactatgtaatacaggtgtgagtctgagaacatatcgaaacaacttatcaaaaagctaattgtattccttaaatactagaagcagtgaagcacaaacagtggtctctcaatgtaaattacacaaagcaaatgctacaatgtttgaagtagaaaccatttaataaagaagaaaaggcattcaaccccatttcctggcaatatttagtttataagcacattgtcagtctggtcatcatttttcactgctggttttgtggtctctgctgctggaagttaggatgattttggggtgtgttttgcatagctaccatgcctgattgtagattctgagcactttgcgacatgtttccaaatgcaccctgtgtattatgttgctgctgttgctgaatgggcatcataccaccagcattggctgctcccatgttaaacatctgatcgctcagattttggggaggcagagttcgatttccaccagacattccaaactgcactcttgggagaattgattgtgatggcatcatagctgacatgttagggatcatttgagatgttggtgtcgtctgggcagcattaaacagtgcactatttgctgggcttgagaactggtttaattgacgattcagctggttcccctggtttagttggttttgacccatatgcattgcagctgatgtaagctgtctaccctgtagttgctgggctgccaatggctgagagaactgtacatgatgctgaccatgcatttgcgattgaaatttttgttgcgactggctaggcatgtcatgcagctgcgatagttggttctgtcccaggacaggcattgacgatggtcttagctgctgttgactcagaagttgttgctgatgtggaggtaattgcatcattttctgcctctgctggtgctggtgttgctgtagttgttgttgcggctgttgttgctgaggagatggtgtgttcatgatgtttgcaccagatctgggtgaattagcatattgtagaggagatgctgtcgtattatcaaaggaagtgctcccagtagcaccagaaggggaagctgctggtcttccaataagctgtgcaccagaagcctgtaacaaagaaccctgactgttattactggcggttgacaaaagtggaggtgtacccttctggtacattccagatgaatcggcaaaagtttgcatactgtcattagcagtgagtacatctaccaaatgcattggaagtgaagatgtcgcttgcctttggtcaccaggtatttttaacccttcaccatggttgacagcacttcgaagcaaattctcttgttcttgtatttttgcagcttgaaccttgtcaatggttgggagaagtgttggtccctgtcgagtaccaaagtaggttttacgtgcatcacctatgatcttctcagcactttcacaggctgctccaatcatatcaattctagtctttagcttttcaatttgggcagccactggcaggccctccataccatgtagcagctgtgctctcttggtgttgtcctccatctccatttcaggcaaaagcttagacgacagcattacaggcagaatggtagcattctccgcatttacattcttaggatgcacaacaaaggccttagaaacgttcttaatatcttcaacaatattgtaaagctccagatttaccatcgagaactgccccagtatatcttgccatttgggaacggcatttgctcgggctatggcgtcgaattcctcgaggatgcgagagatggctttggaaaggctgatagcgcgaatcttcaccgagtcgaggttgagttgctgctgaaccgccgtgttcagccgctccaccacccttgctggcggcggtt

>comp27902_c1_seq2 mediator of rna polymerase ii transcription subunit 8-like isoform x2

gtctggtttctgcaagtataggaatgttaacatggttttatatattctgaaagaaatgattgatacaaatattgcagtagactctaaagcattcaaatcgatgcaaaaacttctgggtgactctcccaagtctcttttagctaatctcttttttgagttgagaaattcggggctgttgcctgaagtggagatgttgatggtcgatggacatgatactagtgcctatgccagtttcagttcatcgaatgattactggaatgttcttgaccttggcacatccggcattgacgagctatccaatgctgttgtttcagtgggctgactgttgagtaaagctagccgttaacatgcgatcattgttgaagctctctttgatgtttcatctcgaagaaaccaacactaaattgcatcttcacgtcttttggtcgtcgaagattcacatttcccttgattattgggctacagctttctgcaaactgaagtgaaacaatagttgcaatcaagtgaatgttcatatattagattatccagattccatttttcgtgtcggcactatgtaatacaggtgtgagtctgagaacatatcgaaacaacttatcaaaaagctaattgtattccttaaatactagaagcagtgaagcacaaacagtggtctctcaatgtaaattacacaaagcaaatgctacaatgtttgaagtagaaaccatttaataaagaagaaaaggcattcaaccccatttcctggcaatatttagtttataagcacattgtcagtctggtcatcatttttcactgctggttttgtggtctctgctgctggaagttaggatgattttggggtgtgttttgcatagctaccatgcctgattgtagattctgagcactttgcgacatgtttccaaatgcaccctgtgtattatgttgctgctgttgctgaatgggcatcataccaccagcattggctgctcccatgttaaacatctgatcgctcagattttggggaggcagagttcgatttccaccagacattccaaactgcactcttgggagaattgattgtgatggcatcatagctgacatgttagggatcatttgagatgttggtgtcgtctgggcagcattaaacagtgcactatttgctgggcttgagaactggtttaattgacgattcagctggttcccctggtttagttggttttgacccatatgcattgcagctgatgtaagctgtctaccctgtagttgctgggctgccaatggctgagagaactgtacatgatgctgaccatgcatttgctgtcccaggacaggcattgacgatggtcttagctgctgttgactcagaagttgttgctgatgtggaggtaattgcatcattttctgcctctgctggtgctggtgttgctgtagttgttgttgcggctgttgttgctgaggagatggtgtgttcatgatgtttgcaccagatctgggtgaattagcatattgtagaggagatgctgtcgtattatcaaaggaagtgctcccagtagcaccagaaggggaagctgctggtcttccaataagctgtgcaccagaagcctgtaacaaagaaccctgactgttattactggcggttgacaaaagtggaggtgtacccttctggtacattccagatgaatcggcaaaagtttgcatactgtcattagcagtgagtacatctaccaaatgcattggaagtgaagatgtcgcttgcctttggtcaccaggtatttttaacccttcaccatggttgacagcacttcgaagcaaattctcttgttcttgtatttttgcagcttgaaccttgtcaatggttgggagaagtgttggtccctgtcgagtaccaaagtaggttttacgtgcatcacctatgatcttctcagcactttcacaggctgctccaatcatatcaattctagtctttagcttttcaatttgggcagccactggcaggccctccataccatgtagcagctgtgctctcttggtgttgtcctccatctccatttcaggcaaaagcttagacgacagcattacaggcagaatggtagcattctccgcatttacattcttaggatgcacaacaaaggccttagaaacgttcttaatatcttcaacaatattgtaaagctccagatttaccatcgagaactgccccagtatatcttgccatttgggaacggcatttgctcgggctatggcgtcgaattcctcgaggatgcgagagatggctttggaaaggctgatagcgcgaatcttcaccgagtcgaggttgagttgctgctgaaccgccgtgttcagccgctccaccacccttgctggcggcggtt

>comp27916_c4_seq1 serine threonine-protein kinase srk2e-like

attcagtgaagatgaggcaaggttcttctttcagcagctaatatctggagtcagttactgccattccatgcaaatttgccaccgagatttaaagcttgaaaacacattgttggatggaagcccagcaccgcgtctcaaaatatgtgatttcggctactctaagtcatcggtattccattctcaaccaaaatcgactgttg

>comp27936_c0_seq1 xanthoxin dehydrogenase-like

atgggagaaaagtgccttaaattgaaaaagcaaattctttggagttctgtggcatggcaacaaacagctccaacgaagccgctcttcctcgtcaaagattattaggaagggttgctctgatcacaggaggtgctactggcattggagagagcattgtgcgtctgtttcacagacatggtgcaaaagtttgtatagctgatattcaagatggcctgggccagcgtctctgtgaaagccttggtggcagtccggatatttgttttcgccactgtgatgtgacaattgaagatgatgtcaagaatgcagttgacttcaccgtcgacaggtttggtacccttgacataatggtgaacaatgctggaatgggaggcccatgctgtgcggatatccgtgactttgaactgtcagtatttgagcaggtctttgatctcaatgtgaaaggtgttttcattggaatgaagcatgcggctcgcataatgattccagccaagaaaggctcgataatatctatctgcagcgtgacgagcaccgtcg

>comp27936_c1_seq1 short chain alcohol

gaatgagcagctgcaatccaaagttcagagttgttggaaacagttcaatcacttgttctaatttacaagactacattgacaatataatgtgtcttcatctgaatacccggagggagtggtttgtagatgtgaagccaccatcgatcatgagattggtgccgcttatgtactttgcctcgtcgctggccaagaagaccacagcattggcgacatcgtctacagtcaattccacgccctgcaagttagcattatctgctacaaaattacggaaaccagtccatgcatcctcagtcctctcatcatcgggcaagtgtgccagtgccaaccctgttgcaacggcgtaaggtgagacacagttcacgcgtattccatgtttccctagctcagctgcaacattcctggtgagccccaaaacagcatgcttggaccctgtgtat

>comp27973_c0_seq1 poly

gaagaagcatctcctccagttttagcaatttttgatttctgatcactttcaagggctcttttcctcttggcaccacctttggctgttgattgttgcaagagttcgtccttcccttcatgctcggcttttgtgcctttggatgtggaaggattcttcttaacgagtgacagaagtgcggcacagtctgaggctgaaagactgtcccatccagaaaatttctctggttgggaagttgatgatgtttccatgtaacacttcgcatggttccaggacaaggatctaggaccttgaccttcaggcttggttgatatgcgaagctctcccttcacaatcttttggttgcagatgcgacaagtagcacgagatgtttgtgaaacttcaacaccacattcaacaacagcaggagcaggggaacttgaaaccacagcaccatcgacatacttcctgatcttctgctgatcttcccagcgaagcaattctaacccttcaacatcatcaaccgattttatctgattcgctttccttaagatgcaagaagcatggttccacataggcatgaaaccatcaaactgagtggcctggaccatcttgccaagcctgagattctccttg

>comp27981_c1_seq1 probable protein phosphatase 2c 24-like

gccctccgattctcaagccactactgtctgtttcgacagctctcggcgatagcagcagtgatttgctaccgaaatttgggatcgcttccgtctgcggacggcggcgggacatggaggacgctgtagccattcacccttcgttttgccggagagattacgacgccgccgacgcaggtctccactactttggcgtgtacgacggccacggctgctctcacgtggctaccaaatgcaaggagagattgcacgagatggtgaaggaagagctgcttctcgacgacgtcggagagagccgaaacagca

>comp27981_c3_seq1 protein phosphatase 2c

agccgaaacagcagtaactggtggaagtgcgtgatggagcgaagcttcactcgcatggacaaggaagttgttgcatggaacgaaaacgttgtcgataatatgagcgccacctgccgctgtgaactccagacgccggagtgtgacgccgtcggctccacggcggtcgttgccgtcgtcactgcacacaaaattgttgtagccaattgcggcgattcaagggcggtgctctgccgcaacggcaaggctattcctctctccaccgatcataaacctgaccggccggacgagctgagtcggattcaagcggcgggcggccgagtaatttactgggagggtgctcgggttcttggagttcttgccatgtccagagccattggtgataattatttgaagccgtacgtgataccggagccggaggtgacgaccacggagcgaacggcggaggacgagtgtttaatactggccagcgatggattatgggacgttgtctctaaccaa

>comp27981_c3_seq2 protein phosphatase 2c

atcatcgtgtcaaattgtggagattctcgcgccgttctttgccggaacggcgtggcgattcccctttctgtagatcataagccggacagaccggacgagctcaaccgcatagaagaatccggcggccgtgtcatattctgggatgggccaagagttcttggcgtcttagccatgtctcgcgccattggtgataattatttgaagccgtatgtaagctgcgaaccggaggtgacgataacggagcggagcgacgaggatgagtgcttgattttagcaa

>comp27981_c3_seq4 protein phosphatase 2c

atcatcgtgtcaaattgtggagattctcgcgccgttctttgccggaacggcgtggcgattcccctttctgtagatcataagccggacagaccggacgagctcaaccgcatagaagaatccggcggccgtgtcatattctgggatgggccaagagttcttggcgtcttagccatgtctcgcgccattggtgataattatttgaagccgtacgtgataccggagccggaggtgacgaccacggagcgaacggcggaggacgagtgtttaatactggccagcgatggattatgggacgttgtctctaaccaa

>comp28037_c1_seq1 autophagy 18 f isoform 2

gatctcagccaaatgaggcccctgaggctgaaactgaccattgttttgggtacttatcgcagaaatccttcccttttgaaacttggtattttgaagataatcaaaaactggaaccaaatcttttgaccttgcttcaagcatacgaacggggactctttcaatctcgatctctccaccataatcaccttcatcgcttatgtcatcagtctgcattgactgaaaatatatctcagatcttgcccacagtgggttcctgttttgatgcatttgaagttctgcttcggatatattcatatgatgcttttcttcagtcgtgatcttctcttttttggttataacaaaaacatcagaagatgcatcattttgatatttcattctttcgggatatactttactgctatctgaatttccattttccccatacatatcaatattatctcctcgatctttgcgattttgcttttgacatatattccatttctggattgcctcaaccattaatcttacatcagaatcaagattggattcaccagcaagacttactccaggcaaagccgtcataacatcagaagcaggagaaaaacgcagagcatactgtatgacacagccagaaggagagaaaacaagcaggtaataatttttcttcagtgaactcagatcggcatgcataccattgcctttgcagttgtggaaagccgacgcaattgccccagagagagaagtaaaccgcccagttgcagccgcagcagcaccacttactgtgtttctccaaccattacttccattccttattcggctaacagccgagagggtaactgggggaccagatgcacacacacactgttgagtcagcacccgtaatcctgagctaggtgatccatgaactgctggcttcaccgtcaagctggatccactgtttcttgcactagaataagaatcagtggtttgcaaactaaccaatcccccagaaggagatatagcaaaaagatggcttgtccctcttgaggaactgatcataatccaatggctatcactgctaaaacttacgtcctgtataacagcattagtgagaccacgttgcagcctgtaaagatggacataagatggccccgaagcagctcctgaggagccctcagataaaccaggcattattcgaaagacatttatgttgtgaccctggatcgaagccgtcactaaaagggtaccgctgggatcaaagcacaatgataatatgggacttttatgtgccctaaattgtgcaatcacagttttgctgacaatatctctgacgataaccatcccaacactgtctgcgtcagtcgcatgtccattggcaacaccatttaccttcccacaagctgtccctgactgacaattgttgccttcaggtaacagctcagaatagtaccttgttaacttcttataccccatgtctcctaaattcacaataccagcagcaagttgtttgctggactctcttgcataatgtgcaacgaggcttccatttgaagcatgag

>comp28051_c1_seq5 armadillo repeat-containing

gtctcataccatctaactccaagtcaaatccttgatttcttggtttcctttattttggcagaaatttcttgcttttccattgtacataaagagaatgttctgggagatgttgaatcctgacagacctggtctttatctccaggaaatgcgagttgttgcattgttccttggaatcctttattcattcatcccctttgatttcctccgaataggtcaccagaacatcatagatgtgtttgactactctgccatggcgctctcatttatcttgtatctggttggtctgtatcttcgtcgaaggcgtgaaagaaatattagagagttggttgacatggtggcggggcatgattgagagtttctgcacttatgttttccttcatggagctcacctatgactattcgatcttgtatacaagattataaatgagtgtgtccctccaattgtcgagttgtcctcctggagttcagatcactttgtacagatgtctgtctgtcgatttgttctggttggtataatacaacctgtatcacagtcggtgcattttcagaaaactagctaatgttcgcccaggtgtggatgtttgagcgcgatgagtttctgtacaaaagtatgctggttgctcttctttgatgtatgtggatgtttgagcgcgatgagtttctgtacaaaagtatgctggttgctcttctttgatgtatgttgatgtttgtctttggcaaccgcgtgaacttgataacgtacactcctttctttgactaacgtgaaatgtgtgaatagcttaaataagtt

>comp28068_c2_seq2 lactation elevated protein 1-like

aacttaccactcttattcaaaaatgatgttattgcaacaatttttttattcaattggaagctgtacacagattttattacaatccaactatgaataatcagctataatgtcttctcatctaagctttatttgctgcaaaaaatctactccagaaattctggtatataatagcggaattctacagacaagtagtagttcagccggtacaattcggcgcctataattcttctaaaactacgactgtaaggctttgacgatgcgatgatttcctgccaaagtttcagcatgttgccccaagtactctctgctattcatttctgttaacctactaatcgtgcgatctttcgcaaaacctaattcgttgtccacacaaatgtcagaatcatcagattttctcgatcttgaagaggtcctaggagccaattgctgagcatcagctattgttataatccggtcaaaaagttccattggagtgccctcagcagtgcataataatctagctctgttctcatacataacatcaactaaagtgacaaaccgatatgcagcagtcctattggagagaccaaaaattggcacaccatccaaggccagcgtatgaaat

>comp28068_c3_seq1 lactation elevated protein 1-like

tagaggaaacttaatttatgatggctcttcgagaaggcatgatgtttggttcaatacgaatcaccgctctctgtacagcctatccagcagtacagacatttgtcaatgtagacatccgtcttacatgatacatagagcgatttccactgatgcagctaaagttatcaatgcagaactaaatggagggggacctctcttagaatatgagcgaagggtggctgctggtgaacttttggatggagatgcctgtcagctaggtaccttgggggagcttcaaagactatatgatgagcttgttgaaaatgcagaagcttgccgattggatcgttactctacttctgagaaagctgggaggagtaggtggttgtggtcacgtttcattccccaatcttcctgtgcacctgtgaaaggactttatttatatgggggagtcggaactgggaaaactatgttgatggacttgttttttgatcagctacctgataattggaggaagaagaggatccatttccatgactttatgctgaatgttcacagccgtttacaaaagcacaagggagtggcagatcctttagaagttgttgctggagagctatcagatgaagcaatattgctatgccttgacgagttcatggtgactgatgttgctgatgcactaattctgaatcgtttgtttggacatttatttagcaatggagctattcttgttgctacttcaaaccgagctccagataacctttacgaaggaggactgcaaagggatctatttcttccatttattgctactttgaaggaaagatgtgtagttcatgaaattggttcctctgtggactaccggaaaatgacatcggcagaacaaggtttctattttgttggtagacatttgactgtccttctta

>comp28068_c3_seq2 lactation elevated protein 1-like

ttctgtctactcagtccattttataattctgattagaacatatttatcatcaagatgctattgcattagccatagaagttcctgggatgaattaggggcaatcactgtcaatgttctatcttcttgccaatggtttactcctttttttcccttcttcttgttcctcctcctccttaggtgactgatgttgctgatgcactaattctgaatcgtttgtttggacatttatttagcaatggagctattcttgttgctacttcaaaccgagctccagataacctttacgaaggaggactgcaaagggatctatttcttccatttattgctactttgaaggaaagatgtgtagttcatgaaattggttcctctgtggactaccggaaaatgacatcggcagaacaaggtttctattttgttggtagacatttgactgtccttctta

>comp28100_c0_seq1 2-aminoethanethiol dioxygenase-like

acatattatcaacgggttcgagctaaaaaagggactattttgcatttttttctcaatgcacttagaaattactctcgtacgtgcacatcgaaaactcgaatgtattttcttctttagttctccgctaagtatttacatcaaactttccagacaagggggaagaaacctcaaccacccctttcctgcaaagaataaatacaaaagggaaaaagaaatgtagaaagaaaataaatggaggagatacgtaagaaacaaaagttcttatgtcacactgcaacttctgttgacatcctaacggaaactttgcttccttattcgatcattttttcactatctttggaccattgtagggctctccaactacgatgtggtcttctggcacgtccctctccttaagccatgcatacccctccttctcctcctcaggcaccgaaacgttttctactgagaaatggtcaaaggggaactcatggtagtactgacaatggcgaccttcgggatcacagtatggagggccaagcacatctagtacagcacatgcagttttggcagtaaaacggtgcatgtttccaccatcagctggatagagaatggaggttctggaagaggcagtgaactgggaattgaccttaaccttcgccagacgtaatccatcaggatgactttttggatcaatagcatttgtcgtcgtccaatcagtgttcttatccacctcattcgtccagtcaaaagacttaatatgcattgtcccaaatagaagcttgctgaaaactgtcattccaggatggttatgtagtggaatgacacatgttgggggcaagcagaagattccaattgagaatttg

>comp28125_c1_seq2 protein yls7-like isoform x1

gtccctccaggcaggaaaagcttgaacaagactggctccttatctgttttcaaaattgaggattacaatgccacagtggaattttactgggctccattcttggtggagtcaaattccgatgatcccaatatgcacagcattttgaaccgcatcatcatgcctggatcaatcaaaaaacatggcaagaactggaaaaatgtggactatctcattttcaacacatacatttggtggatgaacactttctccatgaaagtcctccgaggatcgttcgacaaaggcgcgaccgagtacgatgaagtcgacaggcctgtagcttatgggagagtgttgaggacttgggctaagtgggttgacaaaaatgtggatcccgatcggactaaagtcttctttatgagcatgtctcctcttcacatcaagagcttggattgggacaaccccgacggcatcaaatgtgctaaagagacagtcccaatactcaacacatcaatgccactcaacgtgggcacggaccgaaggctgcttgttactgcaaacaatgtaacaaagtccatgaaagtgcctgtccatttcctcaacatcaccacactctccgagtaccgaaaagatgcacacacctcagtttacaccatccggcagggcaaaatgctcacggcggagcagcaggccgacccg

>comp28125_c1_seq3 protein yls7-like isoform x1

gagcctgaaatcagagacaaaaaaagaggaggaagaagaagagaagaagaataaaatccaaatgcctgtcgacaacgaagacgacgaggaagaggaggtcgaaatcccacctgaagactgcgacctgttcacaggacaatgggttttcgacaattcttcgtatccaatttacaaagaagatcaatgtgaattccttacagcgcaggtgacatgtttgaggaatggaagacaagattcttcgtaccagaactggagatggcagcccagagattgttctttgccaaaatttaaagcaagattactgcttgagaaactgaggaacaaaagactgatgtttgttggagactctttgaatcggaatcaatgggaatccatggtttgtttggtgcagtcggttgtccctcctggaaggaagagcttgaacaagactggctctttatctgttttcagaatccaggattacaatgccaccgtggaattttactgggctccgttcttggtggaatcaaattcagatgatcctactatgcatagcattttgaaccgcatcatcatgcccgagtcaatcaacaagcatggcgagaactggaagaatgtggactacctcattttcaacacttacatttggtggatgaacacttttgccatgaaaattctgcgaggatcgttcgacgaaggttcgacagaatatgatgaaattccgaggcccgtagcttatgggagagtcttaaatacttggtccaagtgggtcgacaaaaatgtggatccaaatcgcaccacagttttcttcataagcatgtctcctcttcacatcaagagcttggattgggacaaccccgacggcatcaaatgtgctaaagagacagtcccaatactcaacacatcaatgccactcaacgtgggcacggaccgaaggctgcttgttactgcaaacaatgtaacaaagtccatgaaagtgcctgtccatttcctcaacatcaccacactctccgagtaccgaaaagatgcacacacctcagtttacaccatccggcagggcaaaatgctcacggcggagcagcaggccgacccg

>comp28125_c2_seq1 low quality protein: uncharacterized loc101203137

ggaggaggaggaggaggaggaagaggaagagattgaaatccctcccgcagattgtgatttgtttacagggcaatgggtttttgataatgttacacatccattatacaaggaagacgaatgcgaatttttgacggcgcaggtgacctgtttgaggaatggaaggaaagattctttgtatcagagttggaggtggcagccgagagactgttctttgcctaagttcaaagcaagattactacttgaaaaactgaggaacaagaggctaatgtttgtcggcgactcgt

>comp28156_c2_seq1 abscisic acid receptor pyl8-like

gaatgactactctgcctccaaagcctcttgaggttcaaaaaagccttcaaagaaagcttcatccctggtgaacatagccctctattagaagtgcttatgacagttctagacacgatcaatgggctctgtcctgtcctgcacggccagcctctctgaaacatcagcaagcgacttcagattacacttaatcagtgcttcgacaaagtagcatgtttcatcctttgtgtttccttcaggcacatccaccacgaaagattcaattaccatggtccaaggtctcccttcaatgacctcaggatggacagacacaatggaagagtagttcctgagcctgtggtctccaccaataatcctgacactgagtatgtgttcgttatcatcaagcatctccagtctctcagtgctggtagtggccggaagaccagacttgacatccacttccctcagactgccaatttcaagatttccctgcacaacacaccgactgacaaaaggcttgtaattctgtggttgatcaaatctcctcacgaaagaccaaacgagatgaacaggcgctttaacatgcttgatgagaaaagaagtgcattgattatcctttatctcgtgcttgtgatgtttctttatatactctttctcaactccgctcaatcctttgacatccatcatcttcttcttcttcttcttct

>comp28185_c0_seq1 low temprature induced-like protein

tttccagtggaaaatatttttcataatttttttatttgtagaaaaaaacaagagacgatatttttgttgttggaaattgaatagaaggaaaaatggcagatgggacagcaaattgcattgacattcttgtcgccgttcttctgcctcctcttggtgtttttctcaagtttggctgcggagttgagttctggatttgcttgatattgacgcttttcggttacatccctggaataatctatgcggtctatgccatcaccaagtagagtttctctccactggtgaagtgtcgtggttgaattggtgttggtgttggtgttggatgctagctattactcctttcctttttatttttttgtttttaattttttctcctttggtattggtgtctttctggatgatctgaatttattttgtcatattattgtcatggctgtgttatgtggagcaaggaagtgatttatgttagtgtcagggaattgttcattgttctaattaatactttggtcactattacttttccgaccttgattttattaccatacttgtgacaaatttcatg

>comp28229_c0_seq1 geranylgeranyl transferase type-1 subunit beta-like

gcacagtatgtgcttgctaggtgactgccattaggaattgttgccccattatcatttgattgaaattgggagcttctggagccatggaatccataaaactgtcccttttccatgatgcccttgttcctgggttgagcttgtagtgataaaacccaattaattactgcatccttgtcaattcgatcgagagcagagaggacgtcgaggccggagatgacgaagtaagcgagagtgaggcggttgatctcttggtcttgatacggcgtcggcagcagctgatacatcatcaacagatagtttatgtgcggatcccgatcgaacaccgatgattcagaatcggaatcccaaaatgcttg

>comp28229_c2_seq1 geranylgeranyl transferase type-1 subunit beta

tggcttcctccaacttctgccatctcctgtgcctctaatgcaactcaaattgctgcagcctgccaataacaagcctaattggcccaaatatgtgtgaatatatctctctctgtattcttatccagtgaaaataccaaattaaaactgatatgtagcatagatgactgctaatttgcctcttaacagtttcatgcatgaatatttctgtggaaacatagaagaagcacttcctctgtatgatattttgatatgcgaatttaacaacttagtcttcatcttagtcagccttcagctcttcatttttctatatctgctatgcttcaatcttactctttatcttttatctgtaatgcatatctggttccattcttcttcgagtgtgcctgatggattggaaaagagatcatttttacattcctggccatctgctttccccagttctgataaagctaaaaagttccacatagttgttaagctattttggcatttattagcaaaactatgatgaggggttatttccactttgctcatttctaatcattgcctaaagttgctgtagtttattagtttcacccattcatgtggctgaattgaggatctaacttatcatatcttatatgtctcagttttattcccattcatactgggggagaaacagatctccgatttgtgttttgtgcagcggccatttgttcaatgttgaagaactggagtggcatggaccgggagaaagctaaagagtatattagaagttgtcagtcatatgatggtggttttggattgattcctggctcagaatctcatggtgagtggatatgaaagtatccattcttttgatttatattgtgtggttgctttcagaaactatgaacccaatgtgcctgccaatccttccttctcaattttctttatgggacttcatgagcaagtggtgaaagttggttgcttgttttgatgtatgagcgggagatctgatttcatttgctaggctgctggaacttcctttatctgcttctggttcatggttggtcacttcttgcttgttttttgttacgcttatggatgcaaattgttttagtgtcacaggtggtgccacttactgtgctgttgcatctcttaaactgatgggattcatagaagaagattcactgtctaaaaatgttacctgtcatattatcaatgtgccattgcttctggattggagcttgcaggtttattttcttatctatttcagagaaaagcaactaactctttaatatcaagattgtagaactgatattcagcataatgaattatttaatatacttgccgatatggttcagtattgaggggtcaaactcaaa

>comp28229_c2_seq2 geranylgeranyl transferase type-1 subunit beta-like

tggcttcctccaacttctgccatctcctgtgcctctaatgcaactcaaattgctgcagcctgccaataacaagcctaattggcccaaatatgtgtgaatatatctctctctgtattcttatccagtgaaaataccaaattaaaactgatatgtagcatagatgactgctaatttgcctcttaacagtttcatgcatgaatatttctgtggaaacatagaagaagcacttcctctgtatgatattttgatatgcgaatttaacaacttagtcttcatcttagtcagccttcagctcttcatttttctatatctgctatgcttcaatcttactctttatcttttatctgtaatgcatatctggttccattcttcttcgagtgtgcctgatggattggaaaagagatcatttttacattcctggccatctgctttccccagttctgataaagctaaaaagttccacatagttgttaagctattttggcatttattagcaaaactatgatgaggggttatttccactttgctcatttctaatcattgcctaaagttgctgtagtttattagtttcacccattcatgtggctgaattgaggatctaacttatcatatcttatatgtctcagttttattcccattcatactgggggagaaacagatctccgatttgtgttttgtgcagcggccatttgttcaatgttgaagaactggagtggcatggaccgggagaaagctaaagagtatattagaagttgtcagtcatatgatggtggttttggattgattcctggctcagaatctcatgtgtcacaggtggtgccacttactgtgctgttgcatctcttaaactgatgggattcatagaagaagattcactgtctaaaaatgttacctgtcatattatcaatgtgccattgcttctggattggagcttgcagaagcaggcgcgagatggtggctttcaaggtagagcaaacaagccaactgatacttgctatgccttttgggttggaggagttttaaggatcttacatgcagataacttcattgacaagagagcattgcatggatttttgttaacttgtcaatctaagtatgctggtttcagtaagttcccaaggttgctgccagatctttaccactcctattacggattttgtgcgtttagtcttttgaagaaacccggtctcaactctttatgcgttgaactgggtataacagatcatgctgcgaccggactctgatgtccccgtttgcagctcctgaatctttgggtctggctttactgtcattgtgcaagattagacctagtcaaagcaaatacccaccctgttcaacaatatcaatagcattatagaaacatatttctgcaagaaaattactgcggtttcaagtttctttttcgttcatttgatctatgtatactggaagggcattgtgatatttcttgacttcatctctgtcattcagacatatatcatttgtgagaatctgttaatgtagagttggagaaccatcttcca

>comp28229_c2_seq3 geranylgeranyl transferase type-1 subunit beta-like

gactctgaattattattgaaatcaatgaaaaaccttcagcagcctaatggatgttttattcccattcatactgggggagaaacagatctccgatttgtgttttgtgcagcggccatttgttcaatgttgaagaactggagtggcatggaccgggagaaagctaaagagtatattagaagttgtcagtcatatgatggtggttttggattgattcctggctcagaatctcatgtgtcacaggtggtgccacttactgtgctgttgcatctcttaaactgatgggattcatagaagaagattcactgtctaaaaatgttacctgtcatattatcaatgtgccattgcttctggattggagcttgcaggtttattttcttatctatttcagagaaaagcaactaactctttaatatcaagattgtagaactgatattcagcataatgaattatttaatatacttgccgatatggttcagtattgaggggtcaaactcaaa

>comp28229_c2_seq4 geranylgeranyl transferase type-1 subunit beta-like

gactctgaattattattgaaatcaatgaaaaaccttcagcagcctaatggatgttttattcccattcatactgggggagaaacagatctccgatttgtgttttgtgcagcggccatttgttcaatgttgaagaactggagtggcatggaccgggagaaagctaaagagtatattagaagttgtcagtcatatgatggtggttttggattgattcctggctcagaatctcatggtgagtggatatgaaagtatccattcttttgatttatattgtgtggttgctttcagaaactatgaacccaatgtgcctgccaatccttccttctcaattttctttatgggacttcatgagcaagtggtgaaagttggttgcttgttttgatgtatgagcgggagatctgatttcatttgctaggctgctggaacttcctttatctgcttctggttcatggttggtcacttcttgcttgttttttgttacgcttatggatgcaaattgttttagtgtcacaggtggtgccacttactgtgctgttgcatctcttaaactgatgggattcatagaagaagattcactgtctaaaaatgttacctgtcatattatcaatgtgccattgcttctggattggagcttgcaggtttattttcttatctatttcagagaaaagcaactaactctttaatatcaagattgtagaactgatattcagcataatgaattatttaatatacttgccgatatggttcagtattgaggggtcaaactcaaa

>comp28229_c2_seq5 geranylgeranyl transferase type-1 subunit beta-like

tggcttcctccaacttctgccatctcctgtgcctctaatgcaactcaaattgctgcagcctgccaataacaagcctaattggcccaaatatgtgtgaatatatctctctctgtattcttatccagtgaaaataccaaattaaaactgatatgtagcatagatgactgctaatttgcctcttaacagtttcatgcatgaatatttctgtggaaacatagaagaagcacttcctctgtatgatattttgatatgcgaatttaacaacttagtcttcatcttagtcagccttcagctcttcatttttctatatctgctatgcttcaatcttactctttatcttttatctgtaatgcatatctggttccattcttcttcgagtgtgcctgatggattggaaaagagatcatttttacattcctggccatctgctttccccagttctgataaagctaaaaagttccacatagttgttaagctattttggcatttattagcaaaactatgatgaggggttatttccactttgctcatttctaatcattgcctaaagttgctgtagtttattagtttcacccattcatgtggctgaattgaggatctaacttatcatatcttatatgtctcagttttattcccattcatactgggggagaaacagatctccgatttgtgttttgtgcagcggccatttgttcaatgttgaagaactggagtggcatggaccgggagaaagctaaagagtatattagaagttgtcagtcatatgatggtggttttggattgattcctggctcagaatctcatggtgagtggatatgaaagtatccattcttttgatttatattgtgtggttgctttcagaaactatgaacccaatgtgcctgccaatccttccttctcaattttctttatgggacttcatgagcaagtggtgaaagttggttgcttgttttgatgtatgagcgggagatctgatttcatttgctaggctgctggaacttcctttatctgcttctggttcatggttggtcacttcttgcttgttttttgttacgcttatggatgcaaattgttttagtgtcacaggtggtgccacttactgtgctgttgcatctcttaaactgatgggattcatagaagaagattcactgtctaaaaatgttacctgtcatattatcaatgtgccattgcttctggattggagcttgcagaagcaggcgcgagatggtggctttcaaggtagagcaaacaagccaactgatacttgctatgccttttgggttggaggagttttaaggatcttacatgcagataacttcattgacaagagagcattgcatggatttttgttaacttgtcaatctaagtatgctggtttcagtaagttcccaaggttgctgccagatctttaccactcctattacggattttgtgcgtttagtcttttgaagaaacccggtctcaactctttatgcgttgaactgggtataacagatcatgctgcgaccggactctgatgtccccgtttgcagctcctgaatctttgggtctggctttactgtcattgtgcaagattagacctagtcaaagcaaatacccaccctgttcaacaatatcaatagcattatagaaacatatttctgcaagaaaattactgcggtttcaagtttctttttcgttcatttgatctatgtatactggaagggcattgtgatatttcttgacttcatctctgtcattcagacatatatcatttgtgagaatctgttaatgtagagttggagaaccatcttcca

>comp28229_c2_seq6 geranylgeranyl transferase type-1 subunit beta-like

tggcttcctccaacttctgccatctcctgtgcctctaatgcaactcaaattgctgcagcctgccaataacaagcctaattggcccaaatatgtgtgaatatatctctctctgtattcttatccagtgaaaataccaaattaaaactgatatgtagcatagatgactgctaatttgcctcttaacagtttcatgcatgaatatttctgtggaaacatagaagaagcacttcctctgtatgatattttgatatgcgaatttaacaacttagtcttcatcttagtcagccttcagctcttcatttttctatatctgctatgcttcaatcttactctttatcttttatctgtaatgcatatctggttccattcttcttcgagtgtgcctgatggattggaaaagagatcatttttacattcctggccatctgctttccccagttctgataaagctaaaaagttccacatagttgttaagctattttggcatttattagcaaaactatgatgaggggttatttccactttgctcatttctaatcattgcctaaagttgctgtagtttattagtttcacccattcatgtggctgaattgaggatctaacttatcatatcttatatgtctcagttttattcccattcatactgggggagaaacagatctccgatttgtgttttgtgcagcggccatttgttcaatgttgaagaactggagtggcatggaccgggagaaagctaaagagtatattagaagttgtcagtcatatgatggtggttttggattgattcctggctcagaatctcatgtgtcacaggtggtgccacttactgtgctgttgcatctcttaaactgatgggattcatagaagaagattcactgtctaaaaatgttacctgtcatattatcaatgtgccattgcttctggattggagcttgcaggtttattttcttatctatttcagagaaaagcaactaactctttaatatcaagattgtagaactgatattcagcataatgaattatttaatatacttgccgatatggttcagtattgaggggtcaaactcaaa

>comp28229_c2_seq7 geranylgeranyl transferase type-1 subunit beta-like

gactctgaattattattgaaatcaatgaaaaaccttcagcagcctaatggatgttttattcccattcatactgggggagaaacagatctccgatttgtgttttgtgcagcggccatttgttcaatgttgaagaactggagtggcatggaccgggagaaagctaaagagtatattagaagttgtcagtcatatgatggtggttttggattgattcctggctcagaatctcatgtgtcacaggtggtgccacttactgtgctgttgcatctcttaaactgatgggattcatagaagaagattcactgtctaaaaatgttacctgtcatattatcaatgtgccattgcttctggattggagcttgcagaagcaggcgcgagatggtggctttcaaggtagagcaaacaagccaactgatacttgctatgccttttgggttggaggagttttaaggatcttacatgcagataacttcattgacaagagagcattgcatggatttttgttaacttgtcaatctaagtatgctggtttcagtaagttcccaaggttgctgccagatctttaccactcctattacggattttgtgcgtttagtcttttgaagaaacccggtctcaactctttatgcgttgaactgggtataacagatcatgctgcgaccggactctgatgtccccgtttgcagctcctgaatctttgggtctggctttactgtcattgtgcaagattagacctagtcaaagcaaatacccaccctgttcaacaatatcaatagcattatagaaacatatttctgcaagaaaattactgcggtttcaagtttctttttcgttcatttgatctatgtatactggaagggcattgtgatatttcttgacttcatctctgtcattcagacatatatcatttgtgagaatctgttaatgtagagttggagaaccatcttcca

>comp28229_c2_seq8 geranylgeranyl transferase type-1 subunit beta-like

gactctgaattattattgaaatcaatgaaaaaccttcagcagcctaatggatgttttattcccattcatactgggggagaaacagatctccgatttgtgttttgtgcagcggccatttgttcaatgttgaagaactggagtggcatggaccgggagaaagctaaagagtatattagaagttgtcagtcatatgatggtggttttggattgattcctggctcagaatctcatggtgagtggatatgaaagtatccattcttttgatttatattgtgtggttgctttcagaaactatgaacccaatgtgcctgccaatccttccttctcaattttctttatgggacttcatgagcaagtggtgaaagttggttgcttgttttgatgtatgagcgggagatctgatttcatttgctaggctgctggaacttcctttatctgcttctggttcatggttggtcacttcttgcttgttttttgttacgcttatggatgcaaattgttttagtgtcacaggtggtgccacttactgtgctgttgcatctcttaaactgatgggattcatagaagaagattcactgtctaaaaatgttacctgtcatattatcaatgtgccattgcttctggattggagcttgcagaagcaggcgcgagatggtggctttcaaggtagagcaaacaagccaactgatacttgctatgccttttgggttggaggagttttaaggatcttacatgcagataacttcattgacaagagagcattgcatggatttttgttaacttgtcaatctaagtatgctggtttcagtaagttcccaaggttgctgccagatctttaccactcctattacggattttgtgcgtttagtcttttgaagaaacccggtctcaactctttatgcgttgaactgggtataacagatcatgctgcgaccggactctgatgtccccgtttgcagctcctgaatctttgggtctggctttactgtcattgtgcaagattagacctagtcaaagcaaatacccaccctgttcaacaatatcaatagcattatagaaacatatttctgcaagaaaattactgcggtttcaagtttctttttcgttcatttgatctatgtatactggaagggcattgtgatatttcttgacttcatctctgtcattcagacatatatcatttgtgagaatctgttaatgtagagttggagaaccatcttcca

>comp28268_c0_seq1 u-box domain-containing protein 34-like

ttttcaccatgtaattttttcacatacgctttgctattgattcatcaccattttatttcttaggaagcccaaagattctgaggtgccatcaatagttctcaaacatgctcctgacacctgtgacatctatgtggtttctgcaaataaacttgtatcaaattctttgaaccctatgttaacttctggtatgttaataatgaggttccctgattgtttgaatatctttggctaccataactggtctaatttgcacttgagcagagggggaacatgatcctcataaatttggtaaacaagaatccggactttcttcgtcatccgcagactgcaaatatcaaagctgcctctcagatgttagtcatccacattctcaggcacaaactccaacattctcttctatgtattctggtgctgtcaaggaaatatctcaccaagcctcagaagagagcgtctcagatacgtcgagaagtagaagatttacatccatgtcttcgacatactcagaacagtcagacattcaggctgaaatagaaagactgcacctagaattggagaataccctgaccatgtacaatcaagcttgcgaagacctgatccatgtgcaaagcaaggtccacatgctttcttctgaatgtattcaagacgcaaagcgagttaaagatgcagaggaaagagaacagaatctaaggaaaattgctgctcaagagaaagagaaatatctggaagctgagaaggaggttgagatggccaaaaaacttctagctaaagagacatatgaaaggcaaatggcagaactgaatgtccagaaggaatcattagaaaaaaagaaaattgttggtgaactattgactagtgacctacgttacagaagatacacaagagatgacatatggatggcaactggtttctttaatgagaataaattgattggtgaaggagcatatggtaaagtttataagtgcagccttgatcatgccttagtcgctgttaaaactcttcgacctgatgcttccgacagaaaggaggagtttctgagagaggtagaagttcttagtcaattgcgccatcctcacattgttttgttggttggagcctgtccagaaattggctgtcttgtttatgaatatatggagaatgggagcctggaagatcatatcctgcgcagaagaggcagaccttctcttccttggcccgttagattccgaatagcatttgaagtagcttgtggacttgcctttctgcaccactcaatgccagaggctatagttcaccgggacctgaaacctggaaacattctgttagacaaatattatgtgagcaaaattgcagatgtgggcctggcaaaaatcatttacgatattgtgcctgacaatataacagaatatagggagtctattatagcaggtaccctgttctacatggaccctgagtatcaaagaactggaactcttcgaccaaaatccgatctgtattctcttggagtaataattctccagttgttggccgcacgtcgtcctaatgggctcatagtgaagtttgaaaatgctataagcagtggcactttcactgatgttcttgataagtcaattgcagactggccactagctgaagcagcagaactagcacagatagcattgagatgctgcaaacttagatgtagggatcggccagaccttgaaactgaagtattacctatcctgaaaagactttctgaatttgctgattcgactagcttgactgaccgagaccttagacaagcacctaaacactattactgtccaatcctacaggaaataatggacaatccctacatagcagctgatggttttacatacgagcattatgcaataaaagcatggcttgacagacacgatgtatccccagtgacaaaacaaaagctacagcacaagatgctcattccgaatcatatgctgcattcagccattcaagaatgtaaaaaacgcataagaccagcctgaatgtgaaagaactcttgcattgtagaggtttcatggatgcactattcttctacggcatttatgttcttggagcagcgtgaatgtatattttgttcacgcagctgccagattatggtcacagcattgttctacagttcaattgcagctgctcgctggaagcgtacataattggtattgggtttaattgtacatatcaaatagtattaggcaacgtgctaactcaagagtaaggccttcgctatgttatgaaatgtacaaagtggatgaaattgtatggctgctttactattttggtgagtcaagtcttgtcttatgttgcatttagtgattaggccaagaatga

>comp28268_c0_seq2 u-box domain-containing protein 34-like

aggacacaaaagattgagaccttgttattggaaggagacaatcctgccttcgctcttctaaggtatatatctgattcaggaccaacaagcttggtgctgggctcttgctcctccaattactttgcaaggaagcccaaagattctgaggtgccatcaatagttctcaaacatgctcctgacacctgtgacatctatgtggtttctgcaaataaacttgtatcaaattctttgaaccctatgttaacttctggtatgttaataatgaggttccctgattgtttgaatatctttggctaccataactggtctaatttgcacttgagcagagggggaacatgatcctcataaatttggtaaacaagaatccggactttcttcgtcatccgcagactgcaaatatcaaagctgcctctcagatgttagtcatccacattctcaggcacaaactccaacattctcttctatgtattctggtgctgtcaaggaaatatctcaccaagcctcagaagagagcgtctcagatacgtcgagaagtagaagatttacatccatgtcttcgacatactcagaacagtcagacattcaggctgaaatagaaagactgcacctagaattggagaataccctgaccatgtacaatcaagcttgcgaagacctgatccatgtgcaaagcaaggtccacatgctttcttctgaatgtattcaagacgcaaagcgagttaaagatgcagaggaaagagaacagaatctaaggaaaattgctgctcaagagaaagagaaatatctggaagctgagaaggaggttgagatggccaaaaaacttctagctaaagagacatatgaaaggcaaatggcagaactgaatgtccagaaggaatcattagaaaaaaagaaaattgttggtgaactattgactagtgacctacgttacagaagatacacaagagatgacatatggatggcaactggtttctttaatgagaataaattgattggtgaaggagcatatggtaaagtttataagtgcagccttgatcatgccttagtcgctgttaaaactcttcgacctgatgcttccgacagaaaggaggagtttctgagagaggtagaagttcttagtcaattgcgccatcctcacattgttttgttggttggagcctgtccagaaattggctgtcttgtttatgaatatatggagaatgggagcctggaagatcatatcctgcgcagaagaggcagaccttctcttccttggcccgttagattccgaatagcatttgaagtagcttgtggacttgcctttctgcaccactcaatgccagaggctatagttcaccgggacctgaaacctggaaacattctgttagacaaatattatgtgagcaaaattgcagatgtgggcctggcaaaaatcatttacgatattgtgcctgacaatataacagaatatagggagtctattatagcaggtaccctgttctacatggaccctgagtatcaaagaactggaactcttcgaccaaaatccgatctgtattctcttggagtaataattctccagttgttggccgcacgtcgtcctaatgggctcatagtgaagtttgaaaatgctataagcagtggcactttcactgatgttcttgataagtcaattgcagactggccactagctgaagcagcagaactagcacagatagcattgagatgctgcaaacttagatgtagggatcggccagaccttgaaactgaagtattacctatcctgaaaagactttctgaatttgctgattcgactagcttgactgaccgagaccttagacaagcacctaaacactattactgtccaatcctacaggaaataatggacaatccctacatagcagctgatggttttacatacgagcattatgcaataaaagcatggcttgacagacacgatgtatccccagtgacaaaacaaaagctacagcacaagatgctcattccgaatcatatgctgcattcagccattcaagaatgtaaaaaacgcataagaccagcctgaatgtgaaagaactcttgcattgtagaggtttcatggatgcactattcttctacggcatttatgttcttggagcagcgtgaatgtatattttgttcacgcagctgccagattatggtcacagcattgttctacagttcaattgcagctgctcgctggaagcgtacataattggtattgggtttaattgtacatatcaaatagtattaggcaacgtgctaactcaagagtaaggccttcgctatgttatgaaatgtacaaagtggatgaaattgtatggctgctttactattttggtgagtcaagtcttgtcttatgttgcatttagtgattaggccaagaatga

>comp28268_c0_seq5 u-box domain-containing protein 34-like

ttttcaccatgtaattttttcacatacgctttgctattgattcatcaccattttatttcttaggaagcccaaagattctgaggtgccatcaatagttctcaaacatgctcctgacacctgtgacatctatgtggtttctgcaaataaacttagggggaacatgatcctcataaatttggtaaacaagaatccggactttcttcgtcatccgcagactgcaaatatcaaagctgcctctcagatgttagtcatccacattctcaggcacaaactccaacattctcttctatgtattctggtgctgtcaaggaaatatctcaccaagcctcagaagagagcgtctcagatacgtcgagaagtagaagatttacatccatgtcttcgacatactcagaacagtcagacattcaggctgaaatagaaagactgcacctagaattggagaataccctgaccatgtacaatcaagcttgcgaagacctgatccatgtgcaaagcaaggtccacatgctttcttctgaatgtattcaagacgcaaagcgagttaaagatgcagaggaaagagaacagaatctaaggaaaattgctgctcaagagaaagagaaatatctggaagctgagaaggaggttgagatggccaaaaaacttctagctaaagagacatatgaaaggcaaatggcagaactgaatgtccagaaggaatcattagaaaaaaagaaaattgttggtgaactattgactagtgacctacgttacagaagatacacaagagatgacatatggatggcaactggtttctttaatgagaataaattgattggtgaaggagcatatggtaaagtttataagtgcagccttgatcatgccttagtcgctgttaaaactcttcgacctgatgcttccgacagaaaggaggagtttctgagagaggtagaagttcttagtcaattgcgccatcctcacattgttttgttggttggagcctgtccagaaattggctgtcttgtttatgaatatatggagaatgggagcctggaagatcatatcctgcgcagaagaggcagaccttctcttccttggcccgttagattccgaatagcatttgaagtagcttgtggacttgcctttctgcaccactcaatgccagaggctatagttcaccgggacctgaaacctggaaacattctgttagacaaatattatgtgagcaaaattgcagatgtgggcctggcaaaaatcatttacgatattgtgcctgacaatataacagaatatagggagtctattatagcaggtaccctgttctacatggaccctgagtatcaaagaactggaactcttcgaccaaaatccgatctgtattctcttggagtaataattctccagttgttggccgcacgtcgtcctaatgggctcatagtgaagtttgaaaatgctataagcagtggcactttcactgatgttcttgataagtcaattgcagactggccactagctgaagcagcagaactagcacagatagcattgagatgctgcaaacttagatgtagggatcggccagaccttgaaactgaagtattacctatcctgaaaagactttctgaatttgctgattcgactagcttgactgaccgagaccttagacaagcacctaaacactattactgtccaatcctacaggaaataatggacaatccctacatagcagctgatggttttacatacgagcattatgcaataaaagcatggcttgacagacacgatgtatccccagtgacaaaacaaaagctacagcacaagatgctcattccgaatcatatgctgcattcagccattcaagaatgtaaaaaacgcataagaccagcctgaatgtgaaagaactcttgcattgtagaggtttcatggatgcactattcttctacggcatttatgttcttggagcagcgtgaatgtatattttgttcacgcagctgccagattatggtcacagcattgttctacagttcaattgcagctgctcgctggaagcgtacataattggtattgggtttaattgtacatatcaaatagtattaggcaacgtgctaactcaagagtaaggccttcgctatgttatgaaatgtacaaagtggatgaaattgtatggctgctttactattttggtgagtcaagtcttgtcttatgttgcatttagtgattaggccaagaatga

>comp28268_c0_seq6 u-box domain-containing protein 34-like

aggacacaaaagattgagaccttgttattggaaggagacaatcctgccttcgctcttctaaggtatatatctgattcaggaccaacaagcttggtgctgggctcttgctcctccaattactttgcaaggaagcccaaagattctgaggtgccatcaatagttctcaaacatgctcctgacacctgtgacatctatgtggtttctgcaaataaacttagggggaacatgatcctcataaatttggtaaacaagaatccggactttcttcgtcatccgcagactgcaaatatcaaagctgcctctcagatgttagtcatccacattctcaggcacaaactccaacattctcttctatgtattctggtgctgtcaaggaaatatctcaccaagcctcagaagagagcgtctcagatacgtcgagaagtagaagatttacatccatgtcttcgacatactcagaacagtcagacattcaggctgaaatagaaagactgcacctagaattggagaataccctgaccatgtacaatcaagcttgcgaagacctgatccatgtgcaaagcaaggtccacatgctttcttctgaatgtattcaagacgcaaagcgagttaaagatgcagaggaaagagaacagaatctaaggaaaattgctgctcaagagaaagagaaatatctggaagctgagaaggaggttgagatggccaaaaaacttctagctaaagagacatatgaaaggcaaatggcagaactgaatgtccagaaggaatcattagaaaaaaagaaaattgttggtgaactattgactagtgacctacgttacagaagatacacaagagatgacatatggatggcaactggtttctttaatgagaataaattgattggtgaaggagcatatggtaaagtttataagtgcagccttgatcatgccttagtcgctgttaaaactcttcgacctgatgcttccgacagaaaggaggagtttctgagagaggtagaagttcttagtcaattgcgccatcctcacattgttttgttggttggagcctgtccagaaattggctgtcttgtttatgaatatatggagaatgggagcctggaagatcatatcctgcgcagaagaggcagaccttctcttccttggcccgttagattccgaatagcatttgaagtagcttgtggacttgcctttctgcaccactcaatgccagaggctatagttcaccgggacctgaaacctggaaacattctgttagacaaatattatgtgagcaaaattgcagatgtgggcctggcaaaaatcatttacgatattgtgcctgacaatataacagaatatagggagtctattatagcaggtaccctgttctacatggaccctgagtatcaaagaactggaactcttcgaccaaaatccgatctgtattctcttggagtaataattctccagttgttggccgcacgtcgtcctaatgggctcatagtgaagtttgaaaatgctataagcagtggcactttcactgatgttcttgataagtcaattgcagactggccactagctgaagcagcagaactagcacagatagcattgagatgctgcaaacttagatgtagggatcggccagaccttgaaactgaagtattacctatcctgaaaagactttctgaatttgctgattcgactagcttgactgaccgagaccttagacaagcacctaaacactattactgtccaatcctacaggaaataatggacaatccctacatagcagctgatggttttacatacgagcattatgcaataaaagcatggcttgacagacacgatgtatccccagtgacaaaacaaaagctacagcacaagatgctcattccgaatcatatgctgcattcagccattcaagaatgtaaaaaacgcataagaccagcctgaatgtgaaagaactcttgcattgtagaggtttcatggatgcactattcttctacggcatttatgttcttggagcagcgtgaatgtatattttgttcacgcagctgccagattatggtcacagcattgttctacagttcaattgcagctgctcgctggaagcgtacataattggtattgggtttaattgtacatatcaaatagtattaggcaacgtgctaactcaagagtaaggccttcgctatgttatgaaatgtacaaagtggatgaaattgtatggctgctttactattttggtgagtcaagtcttgtcttatgttgcatttagtgattaggccaagaatga

>comp28274_c1_seq1 respiratory burst oxidase homolog protein a-like

gacaccgacgtcggagtctcgatgccggtgattccggaaatgacgtcattaccgtcgatgctgatgaccgtggccggttcgacgctgcgtaggatgatagtgtcgtcgtcctgaagatcgagcgtcacttcgacgaactccgccgcggcgttcgtcgacaaatcagtggaatactccgttgccggtgaagatccttcgctggttaaagtcccggcaggtccgctgtcggagccccaccggcgctcatgttttcctgtccccctcattgaattaacaaaaatgtgatactttttctttatgattaatttcttagtttcgtgttagcatagaaaaatgatgattattaggaggacttgcacaatggggggagttgagaaacggagaataaactactcct

>comp28274_c3_seq1 respiratory burst oxidase homolog protein a-like

ggaaatgttctaacattacaaatgtcaaagcctccacagtttaaatataagagcggacaatatatgtttgtccagtgtccagccgtttctccgtttgaatggcatccattttcaattacctcagcacctgatgacaattaccttagcattcacatacggcagttaggagactggacacatgaacttaagagggtattctctgaggcatgtgagcctcctgctggt

>comp28274_c4_seq1 respiratory burst oxidase homolog protein a-like

ggtgggaagagtgggctactcagagctgatgaaaacaccaagaaaagtttgccaaagctattaatagatggaccttatggagctccagcgcaagactataggaagtacgacgtcctcttacttgttggtcttggcattggtgcaacacctttcatcagcatcctgaaagatttactcaacaatattgtaaaaatggaggaacaggctgattcaatctcagatttcagcaggtattcagaccaaagtgcgggttcttttgagtcatctctgaacaaagtttctccaaaacggaagaaaactcttaggactacaaatgcttacttttattgggtcacaagagagcaaggctcatttgattggttcaaaggggtcatgaatgaagttgccgaacttgatcaaagggtaatgcagcttctcaacatctcatttcatctcaatgctagtatattctactgact

>comp28274_c4_seq2 respiratory burst oxidase homolog protein a-like

ggtgggaagagtgggctactcagagctgatgaaaacaccaagaaaagtttgccaaagctattaatagatggaccttatggagctccagcgcaagactataggaagtacgacgtcctcttacttgttggtcttggcattggtgcaacacctttcatcagcatcctgaaagatttactcaacaatattgtaaaaatggaggaacaggctgattcaatctcagatttcagcaggtattcagaccaaagtgcgggttcttttgagtcatctctgaacaaagtttctccaaaacggaagaaaactcttaggactacaaatgcttacttttattgggtcacaagagagcaaggctcatttgattggttcaaaggggtcatgaatgaagttgccgaacttgatcaaaggggtgttattgagatgcacaattatctaactagtgtatatgaggaaggagatgctcgctcagctctcatcactatggttcaggcacttaaccacgccaaaaatggggtcgatattgtatctggcacaagggttagaacacattttgcaaggcctaactggaagaaagttctctctaaaattgggaccaaacatgccaatgcaagaataggtcggtatacgattcaaaaacttttgactatatccagttttcattcatgtcttaacattcaggggattggttctttt

>comp28274_c4_seq3 respiratory burst oxidase homolog protein a-like

ggtgggaagagtgggctactcagagctgatgaaaacaccaagaaaagtttgccaaagctattaatagatggaccttatggagctccagcgcaagactataggaagtacgacgtcctcttacttgttggtcttggcattggtgcaacacctttcatcagcatcctgaaagatttactcaacaatattgtaaaaatggaggaacaggctgattcaatctcagatttcagcaggtattcagaccaaagtgcgggttcttttgagtcatctctgaacaaagtttctccaaaacggaagaaaactcttaggactacaaatgcttacttttattgggtcacaagagagcaaggctcatttgattggttcaaaggggtcatgaatgaagttgccgaacttgatcaaaggggtgttattgagatgcacaattatctaactagtgtatatgaggaaggagatgctcgctcagctctcatcactatggttcaggcacttaaccacgccaaaaatggggtcgatattgtatctggcacaagggttagaacacattttgcaaggcctaactggaagaaagttctctctaaaattgggaccaaacatgccaatgcaagaataggagttttctactgtggggctcctgtattggcaaaagaattgaacaagctctgccacgaatataatcagaagggcccgaccaaattcgaattccacaaagaacacttctaagagacagtatgaggaatacatttaatatggaacaaccacatgtttaatactactatattgtgtcacattcttggcaagcccctgaatccatttgaagtctccatttcacagagtaagctgaaagggagctctagagtatggagaacacgcaaggaggaaataatatttgtatagaacttggtgggaagccaaagtaggattacaagcaaatgtatacatacaagtatatatatatatatatctataca

>comp28274_c5_seq1 respiratory burst oxidase homolog protein a-like

ggctctcatattattgcctgtgtgtaggaacaccattacttggctgaggtcctctaggttgggctattttgtgccgttcgatgacaatatcaactttcacaagacaattgctgcggccattgtgattggtatcatactccatgctggcaaccatcttgcttgtgactttcctaggcttataaatgaatctgataccacatacaaaaagtatttgattgatgactttggcgatcataagcccaagtacatggaccttgttagagggatggagggtgtgacaggaatcctgatggtgatcctcatgataattgcttttacattggcaacacggtggtttagacggagtctcattaagctgcctaagccattcgacaggctcactggctacaatgctttctggtattcgcaccacctgttcgcctttgtctacatcttgcttatcatccatggcacgttcctttatctcgttcaccagtggtacaaaaagacgacgtggatatatcttgcagtccctgtacttctctacgcaggagaaaggacccttaggttcttccgatcaggcttttatagcgtccggcttttgaaggttgccatatatcccggaaatgttctaacattaca

>comp28274_c6_seq1 respiratory burst oxidase homolog protein a-like

gccatgttgagtttcagcgtctcggccgcgcccttagccgtgagaaggcaataacccattacccgaaacgcattcttttgtttgtactgatagaatttccaggagaaaaggccaatcattatcatgatccaaagtgttaagacccaaattctcctccaattttcttgcaaaaaatagaggaattttgtgctcattctcctaatccggcctctgtatctgagcccatgaagattttggctcagagcctggctcgtgtagctgagggcttggctgtaatttaggtatgtgtccttctgtagcaatagtgtttccagctgccacagctcaatgtagccaagcctttcagggtctaattcttccatgatcagagctgcgtactcttctgcctgctcttttaatcttgataatttatttgcagaggcacttaccataataatctccttcacttcctcttcggtgatccgaccatcctcatttttgtccaccatgtcgaagaaaatctggagtctggaatcgaaactttgatcggtaatttgcgaccaaaattcgtagagctcatctctgctgatcttatcaactttcaatcttctccttctgcttaatgcgtcgaacaattccaatgcaaattcctttgaatctttcatccctatgcattgtgcaaaatcagcgcgatgaagataaccgtctttggcgagcttctcgaaattgatctgaacttcattccacgcatcaacggggttgttattactgttactgataaatttaagcccccggagagctttctgagcgccggagcgcgtccgatcaagctgcgcccgctggcggcgcatcgccctcgccgccagcgccaagtccaagccgctgctgcctccgccgacgccagagagtatccgcgacgcgcggccgtggctccacgaaaacctccttagctcctgcgataactgcttcgctttcgccagcgcctccgctttcaactcctgagaaaattgaagcagcttattggaagagctccggcgcatggtcggagatctcggggccga

>comp28324_c1_seq1 heat shock 70 kda mitochondrial-like

tcttggaggtggaacttttgatatttctattctggagatatcaaatggtgtctttgaggtcaaagccaccaacggtgacacctttttgggaggagaggactttgataatgctctgctggaatttttggtgagtgagtttaagaggactgatgcaattgatctgtcaaaggacaggcttgctcttcagagactccgtgaggcagctgagaaagcaaagattgagctttcatcaacatctcaaactgaaatcaacttgccctttatcactgctgatgcatccggggcaaagcacttgaatataacattaactagatccaagtttgaggctttggtgaaccacttaattgaaaggactagggacccttgtaagagttgtttaaaggacgctggaatatcaactaaggaggttaatgaggtgcttcttgttggagggatgacccgtgttccaaaggttcaggaagttgtttctgaaatctttggcaagtctccaagcaaaggagttaatccagatgaggcagttgctatgggagcggctattcagggaggtattctccgtggtgatgtcaaagag

>comp28324_c2_seq1 heat shock 70 kda mitochondrial-like

aggagcttgttgtcggaagccatttcacgctccccttgcaatacttttatacccacttgagtctgattatctgcagctgttgagaacacctgacttttcttggtaggaatggttgtattcctgttgatcaatctagtaaatataccccccaatgtctcaataccgagtgagagtggagtgacatcgaggagaagcaactctttgacatcacca

>comp28324_c3_seq1 heat shock 70 kda mitochondrial-like

atttaagctcttctcaatgctgtaaatagtggtatctgcattatttctgagatctatcaatgctttcctctcttgatctctttgcatgtgcatctcagcctccttaaccatcctttcaatctcatcttctgagagaccacctgatgaccgaatggtgatctgctgctctttacctgtcgtcttatctcttgcagacacagtgacaataccgttggcatcgatatcaaatgtcacttctatctgaggcatgcccctgggagctggggggatacccacaagctcaa

>comp28324_c5_seq1 heat shock 70 kda mitochondrial-like

aaaaatcctctctctcttcttgtagctattcgttaaaccctaaaccctagcagaaccgatcgaagaaaattcaaatacagttctgctgtgcgcactaatcgatccatggcggccgccgttctcctccgatccttccgtcgccgtgaattttcatccgcgtcggtttcggcttttaaaactttgactggaaattccaagccatcgtgggcgttgagtaacaaatgggcaggacttgtgaggccatttagtacgaaaccagctggaaatgatgttatcggcattgacttgggtaccactaactcttgtgttgctgtcatggagggaaagaatccaaaagttattgagaatgctgagggtgctcgcaccactccttctgttgttgctttcaaccaaaaaggggaattattggttggcacgccagcaaagcgacaggctgtcaccaatccaactaacacagtttttggaaccaaacgtttaattggtagacggtttgatgatcctcagacgcaaaaggaaatgaagatggttccctataaaattgtcagggctccaaatggggacgcatgggttgaagccaatgggcagcaatactcccctagccaaatcggagctttcattctgacaaagatgaaggaa

>comp28358_c0_seq1 heat shock 70 kda protein 16-like

tttattttttttaacgcatagtcatagccgttgtatatttatacgtattgctttaccatgtgaggatgttacgctaatgttgtaaattaatcacgatttagtcagttgctttctacagttttagattttcttgaaatccatgtgttaatcatgatgatatttttgtgtaaataattggaattttcttttgtgttcatgagatagatatttcgtgttcctggcagggaggtttttttgtcaactatttgattgcaggcgttgaatctgtggtttggtgatgaattccatgtagttagatacaacttgagattcctgtgattgggcttttatctggagatgagtgtggtggggtttgacgtcggaaatgagaactgtgttatagcagtggccaagcaacgaggcattgatgtcttgttgaatgatgagtcaaaacgagaaaacccagcggtggtttcgtttggggaaaaacagaggtttatgggttctgctggggctgcgtcagcaactatgcacccaaagtcgactatatctcaggtcaagagattgattggtcggagttttaatgaatccgctgtggaggatgacctaagattgctcccatttgagacatctgagggacctgatggtggaatcctgattcacctgcattacttgaatgaagagcaaatatttaccccagtacagattctggcaatgttgcttggacatttaaagcagatcacagaaaataatcttcagatgcagattgccaattgtgtaattggtgtaccatcttactttacagctctacagagacgtgcatatttgcatgctgcggagattgcaggtttgaagccggtaaggttgatgcacgactgtacggctattgcactgggttatggaatatacaaatcagacttcccaaacgggggtccagcaaatgttgtctttgtagatgttggtcacagtgatactcaagtggctgttgtatcatttaagcctggacgtatgaaggtattatcccatgcttttgacagcaacttaggaggaagagactttgatgaagttctatttagacattttgctgctcaatttagggaacagtacaacattgacgtctattcaagtgctcgcgcttctgtaaggctcagagcatcatgtgagaaactgaagaaagttttgagtgctaacccagaggcaccgcttaatattgagtgcttgatggaagagaaagatgtcaaaggatacatcaagagagatgagtttgagaaactggcatctgcattgctggaaagaattagcattccatgtcgcaaggctttgctcgactctggtctgactgttgagaagatccatactgttgaacttgttggatcaggttctcggatccctgctattacaaagatgttgaattcactttttaggaaagagcccagccggacactaaatgcaagtgaatgtgtggctctcggctgtgctgttcagtgtgcaatgcttagccccatatttcgagtgagagagtatgaggtggtggactgcttcccattctcaattgcatttgcatcagatgaagggccagtgcgctcattagcagatggagtgctgttccctaaaggcaattcttttccaagtacaaagattcttacattgcacagaaatgatatcttt

>comp28358_c0_seq2 heat shock 70 kda protein 16-like

ttctctttttatttctctatttatcggagcagagggaggttttttttgtcaactatttgattgcaggcgttgaatctgtggtttggtgatgaattccatgtagttagatacaacttgagattcctgtgattgggcttttatctggagatgagtgtggtggggtttgacgtcggaaatgagaactgtgttatagcagtggccaagcaacgaggcattgatgtcttgttgaatgatgagtcaaaacgagaaaacccagcggtggtttcgtttggggaaaaacagaggtttatgggttctgctggggctgcgtcagcaactatgcacccaaagtcgactatatctcaggtcaagagattgattggtcggagttttaatgaatccgctgtggaggatgacctaagattgctcccatttgagacatctgagggacctgatggtggaatcctgattcacctgcattacttgaatgaagagcaaatatttaccccagtacagattctggcaatgttgcttggacatttaaagcagatcacagaaaataatcttcagatgcagattgccaattgtgtaattggtgtaccatcttactttacagctctacagagacgtgcatatttgcatgctgcggagattgcaggtttgaagccggtaaggttgatgcacgactgtacggctattgcactgggttatggaatatacaaatcagacttcccaaacgggggtccagcaaatgttgtctttgtagatgttggtcacagtgatactcaagtggctgttgtatcatttaagcctggacgtatgaaggtattatcccatgcttttgacagcaacttaggaggaagagactttgatgaagttctatttagacattttgctgctcaatttagggaacagtacaacattgacgtctattcaagtgctcgcgcttctgtaaggctcagagcatcatgtgagaaactgaagaaagttttgagtgctaacccagaggcaccgcttaatattgagtgcttgatggaagagaaagatgtcaaaggatacatcaagagagatgagtttgagaaactggcatctgcattgctggaaagaattagcattccatgtcgcaaggctttgctcgactctggtctgactgttgagaagatccatactgttgaacttgttggatcaggttctcggatccctgctattacaaagatgttgaattcactttttaggaaagagcccagccggacactaaatgcaagtgaatgtgtggctctcggctgtgctgttcagtgtgcaatgcttagccccatatttcgagtgagagagtatgaggtggtggactgcttcccattctcaattgcatttgcatcagatgaagggccagtgcgctcattagcagatggagtgctgttccctaaaggcaattcttttccaagtacaaagattcttacattgcacagaaatgatatcttt

>comp28367_c1_seq1 at1g70420 f17o7_4

ggtctgcaagaagagcaactcgaccgggttttccaagatttggaggttcaaagatttgctggggaggagtaatagtgatgggagggacgcgtttgttttcttgaacaacagccacgcgccgccgcccacgacggaggatccggcggtgaaaggagaggagaagtgtgtgaggaaggaatcgacggtgaaagtcagccggaaggtgaagaaggacggcaagggtaaaaaggcggcgccgtcggctcacgaagtgtacttgaagagtaaggcgaaggaggaagagcggcggcggtcgtacctgccgtaccggccggagttgatggggttttttactaacgttaatggtggattaagtaaaaatgtacatcccttttaagatgaaaagggtttttcagcgttcaattgtacattttctatagtatctatttcggtagattttgttttattttttgaattttggccatagtggtgttcatgatctaacaagaaga

>comp28367_c4_seq1 at1g70420 f17o7_4

gtaacgccccggaggagctaatacatcgcctatagcggcggaggacgcattcataaacggccagatcaggccggtttttccattgttcagccgagatttacttttctccggcgaggattccgacggtctgcacgaaaacttgcccatgaggccgccggtgaagaaggtgttcgtcgaaacgagtgaagagaatggccaggtaacgtcatccacgccggagagcacgccggtcgccgggcagtattgcgagtggtcgaacagaaaggccgtggaggcgtcgccgggcagtattgcgagtg

>comp28429_c0_seq1 aldo-keto reductase family 4 member c9-like

ggttgacagcaggaggaacacgggctatatccaacaggtcacccagctttttcactgagaaattgctaaccccaatagctcgggccttgctagaatcatagagtgcttccatggccttccatgtgctaggtatatctgaagcaacaaggttttcaggatcaaagccaacagacccctttgccatccggaccggccaatgaataaggaacaa

>comp28429_c2_seq1 aldo-keto reductase family 4 member c9-like

cggccaatgaataaggaacaaatcaacatattcaagttgcaggtcttccagtgttttgtctaacgccacaggtacatctttggtggcatggtcagtacaccagagtttggaggtaataaacaaatcccctctcttcacaactccatcatcaaacaacttcttcagagctaaaccaatctccttctcattgccataagctggagcacagtcaatgtggcgatatccactcttgatcgccgcttcaacggcttgaccgacgagaccaggctccgacagccacgtaccaagtccaaccgaaggaattttagccccggtgttcaactcaaagtacaatatctcgttcgccattttctagagagagaaagagaaggattgagggcgaaaggcgtgaaaattcaacaaacgtatgagaactgcagagtgcagagagag

>comp28429_c8_seq1 aldo-keto reductase family 4 member c9-like

gaatttgtacgcaatgtcttagcataacaagcacaaggtgacctttattaaaaggaaaaagttgagtagcatcaagggacagtaaacggagtataaattccattaaaacatcaatactgaaggtgatgatttgaaagaaatataagatcatattattagaaagcgcgctcagagctctccgtcccaaagttcttcaacggttttgtactcgccgagtgtctcgtggacaaacgcagaacccttaaccagtctttcctgctcaatttcagagaacttggcaaacaggtcatctggtatggaccaaccaaaaacatcaaagttttctttgatccttgactcattcgtgctctttggaagtacgctatgacctatttctaatccccaccggagtgcaacctgcgcaggactcttgcccaatttctctgctgtagcagctaaaacaggatgctggaggacttcactcttgagccatgttttgccaggagagcccaatggtgaataaccggataggtgaattcccttggatttacaaaattcccgcagtttagcctgctgccatgaaggatgacattcaacctggttgacagctggaggaatgc

>comp28429_c8_seq3 aldo-keto reductase family 4 member c9-like

aaataaaagaccatatatgctggtatagactacaattttgaaccatagttacaacgtgtaaatatagcatgcattggtgaggttgcaatatcatatgaaatatcatctgcaataaaggggtttaactagcccaacaccaataggatttaagaattgttttgctctgcgggttctcactctgggacaagcccaatgcttatttcatcccccaaatatatagatgaataaaaacataaccgaatgaaaataagtaaaacaggttcaaagactttggcattgacaagtaatcaacttaacgatcaagagcgtgtataatgtgcatcaccagaaagcaagctcagatctctccatcccacagctcttcaactgttttgtactggctgagtgtctcgtggacagcaaaagtacctctaaccagtcttgcctgctggatttcagagaacttggcaaataagtcatccggtatggaccaactaaaaacatcaaagttttctttgatcctcgattccttcgtgctctttggaagaacactatgacccatttgcaatccccatcgaagtgcaacttgtgccggagtcttgcccaatttctctgcagtcgcaactaaa

>comp28458_c0_seq1 annexin d2-like

gaaaacgttgactttcattgcataattcagctaagtacctccacgggtatatcagaaggacaccattgccatcaaacaaaccagcaagtttcttgtgcctttattcatagccagagctcacacacacacaataaaaaaagatgcacgcacacgcacacacactcaaaaaccccgcgcgagcgcgcacacactgcatattctgcacctgcattcattcttatgcgacaccgtcttcctcgtttttgatcaatgcaaggaggatcttctcatagtctccagaagtgtccccagcaatcgcacgctccagagggatgctattccttctacggtattcttctttgatccgctgcatgttgacctcagcttgagtaacaacaacacgagtgagagagttctcgtctgtgccaagccctttgatagcttggcgaagtgttttctcataatatttctcaggacagatcaaacactttattgttgccctgaggaaggtaaggtactcattg

>comp28472_c1_seq2 kinase protein with adenine nucleotide alpha hydrolases-like isoform 1

attaccttcataaggaatgtccaaggcctgtaattcatagagacatcaagtcatcaaatattctcctaactgatgaattagaaccacagttgtgcgattttggattggcaatatggggacccacaacggcatcttttctgatggacagtgatgtagtgggaacatttggctatcttgctcctgagtattttatgtatggcaaaatcagtgacaagattgatgtatatgcttttggtgtagttctactagaactgctatcagggaggaaacctattggtatggagaccacaaagagtcaagaaagcttagtgatgtgggcaaaaccccgattagaaagcaaggacttgaagagtttactggatccagatttggaaggaaatatcgatgaggctcaaatgcaaaggatggctctagctgcatcactatgtctaactcaagcagcccgtcttcgtcccaacatgttccagatacttaatatacttagaggtgaggaccatatggttggtacaaaaccaaaatgtgggaaccgtgatgatcaagaaaatgaggatgacaacgacgacgaagtttatccagattcaagtgcagaatcccatctaagtcttgccttccttgatataaatgaaaacacatcatcattcagcagccttgatcaaagcagccctctttctgttgaagagtatttaaagaaaagatggagcagatcatcaagtatagaataggttctgaaaagattgtggcctttctttgtacatgagagccaaaaagaaagtctgcacccagaaatgaggtggtttgcagaagtatttaaagtgatgattttgtttatttgttatttcttttttgtaggagtttctggggaaaaaata

>comp28496_c1_seq3 zinc finger protein constans-like 4-like

atctccgaaacagaatttctgcagaagcaaaacttgattatattacacagtgaactggaaatatcacaacaaaatcgacaagtaataatacacggatcaaaaacacgaaaatcatcatcccctacaatatatacacacgacatattcatcatcagttcatcttcgtcatcatcatggtcgtcgtcttgcataccaacaacatcaaaattcataaccaacgatcgggcctcccaaaatccaggtgatactaagatcttactaaaaggtcgggacaacaccgtacgacgaagcatcagcagcaatgagggaatcaacttcaagctccgagcgcttggcaaacctccctttgatcctcggccttgtctctgcatacgcctttcttgaggcgtatcgtattgtttttacaaactttctattctttcttttctctctatacctcaacaccctcgcttctctgtcgatcccagagactcgggtgggaacgccttcgctcttggatacatcggccatggcattgtg

>comp28496_c1_seq4 zinc finger protein constans-like 4-like

ttgcatacaaaatagttatgctctagaaacagctaatcccatacacacttcagctagttgcaacaactgcagtactgaaagttcgcatgaccaaagtttgatcacattacgcagaaaatgcagcatccctgcaaaatcgatgacttaaaacaatgcatcgatcataaacaccgtagtcataatccacctacaatatatgtacacagacatgctgattattgtgttacaacgatgagttatcgttcgttgctcccaaaattacttggcaacggctgatcaaaaagtcgggactacgccataagatgcatcagcagcaatgagggaatcaacttcaagctccgagcgcttggcaaacctccctttgatcctcggccttgtctctgcatacgcctttcttgaggcgtatcgtattgtttttacaaactttctattctttcttttctctctatacctcaacaccctcgcttctctgtcgatcccagagactcgggtgggaacgccttcgctcttggatacatcggccatggcattgtg

>comp28510_c1_seq1 enhanced disease resistance 1

ttcactatgacaacctcataactggatgccccaatgtcgctttcaagatctgtaagagaaggcatgttccctcgagctgctggatctgtagatagattatatacatcatagaaaccatccaccactctctcctcaaagtcgaggacaccaaaattccaatagcggcgcgataacaagtcggcggctgcgtccgcgttatcagtggcgcggatcggggccttgtccgggtcaataatcgcagaatcctgacccgacgacgacacgcttagagctaaggccaattggacctggtactcctcctcggaggcgtaataatcctgctgctgatgatgctg

>comp28510_c3_seq1 ctr2 protein kinase

aaagcagtgcttctggcttccaagatgtagcacccaaaatctgaccaggctgtgttgaggaaactttggtatctgtagttgtctgaagattaggaagcctacttggttgtggactatatgacttcaaaggagtatcctttccgctcagagcatcagctgggattagtgccccaggagctcccatgagatcaaccaaacactcactttcatcctgtaactttataatgttaacagcgtcgtcttcaatgccagtgtaaagactacccttcactaatctgcaaggtattccaacactatcagctaatgccttaaaaagcaatgcacggtgacgtaagaggccaactcttagggatccaataggcagcacagtggtgtgaagagaagtcctcaactccatacttctttccatccatttagccaatatgatatttgcatcaggaacagggccacctagttgttttgtaacaagttcagctagtctctgtataagcagagtgatttcagatggagggcagtccaaaactatgcagtgtgctatctgcatcaactcctccaaagcagggtctatttttctgtt

>comp28510_c4_seq1 serine threonine-protein kinase edr1-like

cttccatgtgaaaacccaatcatcaaggtacgtgtggataacatacaatcagcacgatgacagctggaaaagagaacggaaatatgataaaactatagaagttcaaaaagacttatgaatatgccttgtgcacacttgatgatgtaaatacacatacgctgacatacacgaagtcaaacatggtgagttcccagaaacaagagatggctactgttaccaatatatacattatgacgtgaccgttggccgaaacttgtgttcaactatacaagaaaacggctgaaatacatggacagtgatcgtgacaaaatgatcttccaatttaggattctttgtcttctgtagaattttccatagaactaacacagcaaaatcccattcacttgcatcgggacatcactttgctatacaacaatggtattcacactagaacaaaggagaaatgtctaaggtgtggaatttactgggatctgttgttgcaaagatgcagttggttgctcaacatgggaaggaacgacaagccgctgtaatggcttaagcgccacactgagctgtgcaaaacagggtcgcaaatttggatcggtttgccaacattcccatataatccttgcgaccagaggatcaacctcctttgggatatcaagacggcggttttggaaacctactgcacccacaacttgcattgggttcattccgctccaaggcaatttgagtgttgcaagttcccatagaataacaccaaagctatatacatcacactttgtcgtcaggtgacctgttcatgaacaaggatttcaaacaaccaccagactagaaaaattatgctacctaaat

>comp28510_c4_seq2 serine threonine-protein kinase edr1-like

cttccatgtgaaaacccaatcatcaaggtacgtgtggataacatacaatcagcacgatgacagctggaaaagagaacggaaatatgataaaactatagaagttcaaaaagacttatgaatatgccttgtgcacacttgatgatgtaaatacacatacgctgacatacacgaagtcaaacatggtgagttcccagaaacaagagatggctactgttaccaatatatacattatgacgtgaccgttggccgaaacttgtgttcaactatacaagaaaacggctgaaatacatggacagtgatcgtgacaaaatgatcttccaatttaggattctttgtcttctgtagaattttccatagaactaacacagcaaaatcccattcacttgcatcgggacatcactttgctatacaacaatggtattcacactagaacaaaggagaaatgtctaaggtgtggaatttactgggatctgttgttgcaaagatgcagttggttgctcaacatgggaaggaacgacaagccgctgtaatggcttaagcgccacactgagctgtgcaaaacagggtcgcaaatttggatcggtttgccaacattcccatataatccttgcgaccagaggatcaacctcctttgggatatcaagacggcggttttggaaacctactgcacccacaacttgcattgggttcattccgctccaaggcaatttgagtgttgcaagttcccatagaataacaccaaagctatatacatcacacttttcatttgaaggctcatttcggagaacttctggagccatccactcaggcgttcctgcagtcgacttagaggacaagaatgtattatgcttcaagcgtgacaatccaaaatcacccaccttgacgttccagttgttatccaccaagagattgggtgatttcagatcacggtggacaattgtcggtgtgcttgtgtgtaagcagttcatcccttttgccacatcaagagccattttaattctcctcttttcatcaatttgacaatgagggcgatgaattatccgatataagcttccacgaggcagaaactcggtaataatagaaaggtttggcgggcgagttactgcacccataaagagaacaacatttggatggcgtaatctgcgcattatccgcacttctctcttgaactcatccaaagcagcacccgagaaatcttggtcaagaaacttcttcacagcaacttctgtgccattccaatctgcatggtagacctcgccataagaacctagtccaatcctttcacctaggacaagatcctcccagacaatttcacattcaccaacatcatcaatctctgtgtcaacacgattagcacctgaatcaagtgaacagccagcaccatcagtttccatcattttcatattggtcacagttatcctgtcttgcatattttggttatccccaggcagcctggctgtgacaatcccatggtcttttcgattaggttcttcatccttaataacgtccccatcctccctgagatatgtctcactaccattgaacctaaaatctttcccaaaaggcaatctacttggtctactttcaccagatgccaacttaaaattgttgctacccatactgtcacgtttctctgcagaattattaaacgatttttcaggaaatttgga

>comp28535_c0_seq1 uncharacterized loc101212152

gaccatacttagacaagcagaaaaataatcagacaacctctcaacaaacgcatcactatagagaaaatgtctcaattttgagtataacattagtaaaagaaccctcattctcacatttattttaccaatttctggaggtctgttggtggaaaaggaaacaagaaagaatagaagatcacaattgcagatgaacagacctgcgacagtctagcaacaacaaaatacaagatcagataccacgagcatcagcagaacatgtcagaaggatgatgacgcaacaggaacttgtagagagtttgagttccaatcagcattttccctgcaaccatctctaattccagaaactgctaactgatttggctcaatacttgaagtttcaccaaattctagtttccttggagcaactgtcttcagccgatgcctctctgccctagaaccaattacctgtcctagaatagatgctccaatggtgaatgccatggctgtctttggcatcaatacagatttcctaagcatggctatgaatggcacagcagcatggactgcagcaaaccatgatggtgagaatttttcagtgtgttccctccatattcctaaaggtatattagctgccattcccaacatgccaataacgagtatttttgcaggc

>comp28535_c1_seq1 uncharacterized loc101212152

gataaatattaaactcgatgtctaaatgaatggtgtaatcttgagctctccaaagtctgcctaagagcctcaaagatgacaaaacattgttttcttcgtattggatattcatgaacaattacaaagtgttaaatacaacatgagcacaagaaaactcatcccactgatcattccaatttggttcaataatgtgaaagagagaaaacaaaacaaagaatggaagaacatggcagcaggtcaacagacgaccaaatacaagattgacactgacaatcagcagaacacatcagctgatgatgagggaacttgcagagaatctggtttccaatcgacaatctcactgcaatgcccactacgaattccaccaacagctaactgagtcggttcagttgttaaggtttcagtcagaatcattttcttcatagcaactgtcttcagacgatgcctttctgcccttgaaccgacgacttgtcctaagatggaagctgcaatggtaaatgccatggctgtcttaggcattaatatggatttcctaagcatagctatgaatggcacagccgcgtggactgctgcaaaccatgaaggcgagaatttttctgtatgctct

>comp28535_c2_seq1 uncharacterized loc101212152

tttttgcaggaagaggttgtggtctaaggtttctagcaaaggcggtttttgctagagcactccgggcagcaaccactgctggaggacacctcagcttcactccgggtgggggctgaagtgcctttgctacaagaggaagaacatgactcacggctcgatatgactttgcgattggacagttcccattttgcaaccactcattgctcattgcttcatgttctgatttacctccctttgaagaagaatcctttttggatgaacttgatttcttctttgcattcttccacttctctgaaaatgcatcaaatccaaaaggccctccagagccaaatgcagagaggctgatggtagctgcctttgcagccaacggattgaattgagctggtgatggttcggaaggaggctcctcagtgcaaacaaatgaccttccagagagtggaacaacaccattctgcccatggaaaagcctgaatgccgcatcaaaatttgggccatcctcaaaaattggacctttcccgtcccttacaggcatgggaaaggccattgatgtcaagaaggagaagttagttggctcattgatgttccgcaaaaatggacacctaacaatgtcctggtcaaaaggcgagacctcattgttcaagttcccaaagacaaaatccattctaaacaaataaattgatcttcccaactttaaaaactcaatgctgaagaggggttattatcaaaaatcttgagcgagatagatctttagagggaggaggaagaatagagagtaggtggtaagaggaaataagaagggaagaaatcggacaaaaatttggaatctttagagagagagagagagaga

>comp28535_c2_seq2 zinc finger b-box domain containing protein 1 isoform 1

caataacaagtatttttgcaggcagaggttgtggcctgaggttttttgcaaaagcagtttttgctagagcactcctggcagccacaactgctggaggacacctatacttcatgccaggagggggctgaagggcctttgctacaagaggaagcacgtgactcacagctcgatatgactttgcaatggggcaattcccattttgcaaccactcattgctcattgcttcatgctctgatttccctccctttgacgaagattccttttttgaggattttgattccttgttttctttcctccacttctcaaagaaagcatcaaacccaaaaggcccacctggaccaaaggcagagaggctgatggtggctgcttttgctgccaagggattgaactgagctggtgatggttcagattgagacttctcagtgcgaataaatgattttccagatagtggaacaacaccattctgcccatggaaaagcctgaatgccgcatcaaaatttgggccatcctcaaaaattggacctttcccgtcccttacaggcatgggaaaggccattgatgtcaagaaggagaagttagttggctcattgatgttccgcaaaaatggacacctaacaatgtcctggtcaaaaggcgagacctcattgttcaagttcccaaagacaaaatccattctaaacaaataaattgatcttcccaactttaaaaactcaatgctgaagaggggttattatcaaaaatcttgagcgagatagatctttagagggaggaggaagaatagagagtaggtggtaagaggaaataagaagggaagaaatcggacaaaaatttggaatctttagagagagagagagagaga

>comp28535_c2_seq3 zinc finger b-box domain containing protein 1 isoform 1

caataacaagtatttttgcaggcagaggttgtggcctgaggttttttgcaaaagcagtttttgctagagcactcctggcagccacaactgctggaggacacctatacttcatgccaggagggggctgaagggcctttgctacaagaggaagcacgtgactcacagctcgatatgactttgcaatggggcaattcccattttgcaaccactcattgctcattgcttcatgctctgatttccctccctttgacgaagattccttttttgaggattttgattccttgttttctttcctccacttctcaaagaaagcatcaaacccaaaaggcccacctggaccaaaggcagagaggctgatggtggctgcttttgctgccaagggattgaactgagctggtgatggttcagattgagacttctcagtgcgaataaatgattttccagatagtggaacaacaccattctgcccatggaagagcctgaatgccatatcaaaattgggaccatcctcaaatataggacctttcccatcccggacaggaataggaaaagccattgatgttgagaaggagaagttagttggttcattgatgtttctcagaaatggacacctaagaatgtcctggtcaaaatgcgatgatcccccattcaagtttccaaagacaaaatccattctagaaatgtaaatgccctttaaaatacttaagagctaatgctcaagcgaggctatacaatcaaaaacctcgagcaaaatgggatcttttagagagagaaaacgaagattgagaaattttggagtaggtaagacgatggttttagag

>comp28535_c2_seq4 uncharacterized loc101212152

tttttgcaggaagaggttgtggtctaaggtttctagcaaaggcggtttttgctagagcactccgggcagcaaccactgctggaggacacctcagcttcactccgggtgggggctgaagtgcctttgctacaagaggaagaacatgactcacggctcgatatgactttgcgattggacagttcccattttgcaaccactcattgctcattgcttcatgttctgatttacctccctttgaagaagaatcctttttggatgaacttgatttcttctttgcattcttccacttctctgaaaatgcatcaaatccaaaaggccctccagagccaaatgcagagaggctgatggtagctgcctttgcagccaacggattgaattgagctggtgatggttcggaaggaggctcctcagtgcaaacaaatgaccttccagagagtggaacaacaccattctgcccatggaagagcctgaatgccatatcaaaattgggaccatcctcaaatataggacctttcccatcccggacaggaataggaaaagccattgatgttgagaaggagaagttagttggttcattgatgtttctcagaaatggacacctaagaatgtcctggtcaaaatgcgatgatcccccattcaagtttccaaagacaaaatccattctagaaatgtaaatgccctttaaaatacttaagagctaatgctcaagcgaggctatacaatcaaaaacctcgagcaaaatgggatcttttagagagagaaaacgaagattgagaaattttggagtaggtaagacgatggttttagag

>comp28636_c0_seq1 hypothetical protein M569_03811, partial

cctgaactatcatccatataataaccggaagtttatctagtccttgatattttgattcgcttaattttggcaatagattaataagtacatcaggtttccggcgtaataccattgctaaaactaaaaagatagcaacctgagactttgaggatgctgcttgccctcctttctttgaacccttaggccccaattgttgagcagccaagtcagcaagaatgcaatctaatgaccagatcacaaatgttcctaatgcctcataggaacggtgattgatccagtcaactgacgtcttgtaaacagcttcagagatataagaaacagggacatcggcaatttttgccacagcagactccctgaacaacttatgccacgggaattgcgacccactcatggcagaaaatgcacgcccaaaataatccgcaaacctcatcaactgaatatcttgctgcccttcatacgattccgaaacaccagacagaaaagcgacgagatcatcagcgtcgatcttcgcagcggcctccgccaccgtgaccttaggcttcttaaccttcttcggcttctccttcttatccacctccgccacattattctttacatcggcatcgctactgaggtcatcctcgtcctcttctcgtccgcgatttctcttcgatctctccggaatttcatcctcgtcgtcataaaccacggcggcgcgctgcgcctccaatttccggcgccgctcttccgcgtgcttctccaa

>comp28639_c0_seq2 udp-glucose 4-epimerase 1-like

ctacgaacaagaacaaccatattccttatgtcaacaagggagcgaacagctaaaattttgtcatagtcgtatagtcatacagctacatgcctagaaatatgtaaggataagagaaaataaaattggcattgtattgcatccaaagaatgaatagactatagttgatcatttcaccaagagagagaaatgtattgctgcctcaacaaatacatgagaaatgcttgtaaatcgtataaatttacaaggtttgagcacaaaaaacgtacctttttacagttatatttcaccattactttgtacaaattaattgatccaaccaaattattgtcgaaatatctcatgggataagcgacactttcaccaacagctttacgaccagcaaaatggatcactgcatcaaacttgttctcagaaaacaatttatccaaatcctccacatttctgatatcccccaaatagaattcaagattttggga

>comp28639_c0_seq4 udp-glucose 4-epimerase 1-like

ctacgaacaagaacaaccatattccttatgtcaacaagggagcgaacagctaaaattttgtcatagtcgtatagtcatacagctacatgcctttttacagttatatttcaccattactttgtacaaattaattgatccaaccaaattattgtcgaaatatctcatgggataagcgacactttcaccaacagctttacgaccagcaaaatggatcactgcatcaaacttgttctcagaaaacaatttatccaaatcctccacatttctgatatcccccaaatagaattcaagattttggga

>comp28639_c2_seq1 udp-glucose 4-epimerase 1-like

gaattcaaggttttgggagagttgggggccgaccaacactctgaccctgtgaacagcttcttcgacagcattgtcgaggttgtctatgatggaaacctcaaacccctgtttcagtagctgcaccacacagtgtgtgccgatgaacccagctccccctgtcaccagaatcctctgtttcaaagccattgccgcgaaatatcacaaaattggcgaagaaagctggtgattttattgatgattttgcaagggttcacagttgtgataagtgtgtgccgagtggatgatgagtcaatatcagtagatgatgagagagagaa

>comp28648_c1_seq1 aldehyde dehydrogenase family 3 member f1-like

aatcctgtaaatgttaaacaattttaggtgtcctaaggctttgcagagcagaagtagaggaaaaattgcagagaactaacaaattttctttgtccagtgaaccaccataaacaatggagtctgccacaccaggctcctcaagaagacggcatatcctatcaaaatgatgcttgttcacaattctacaaatactctgcaagttttttacgttttcaccataaaatcttttgatacacctcctcagtaactcaatcagaggtggagcaaacttttgttgtacaaggacataatcaattcctatgcatgcttgtccagcacagggtccccacttcccaccagctattctcttcacagccacatcatcctcttagagactttcaacaaggactgagctttgttccaaaaatgtcaaaaagcatagctctaaggaacctctaagtctgagccagagaaggagtcgagaatgacaggacattttcctccgagctctagagtcactggcgtcaaatgctttgcagctgcagacatgataatgcgtcctacacgtgggctgcctgcagcaacaattaagaagacgaaacaatgtctgaattggcatttgagaaatcaatacataacatggttgaatctcatacatttgaaataaggtcttctacaacaacaatacaaaggcaatccaaatgagagaatccaaattatttttatataccacaatttaaaaagaattgtgtaatctagtcacaactacca

>comp28648_c1_seq2 aldehyde dehydrogenase family 3 member f1-like

aaagtaagaaaagagcatttgaaggctttatatgtcacaactcacaagctcttagcatatttctctgatgaaccactaactaatgaaagcaggataaaaagaaaagaaggatcgaatcacatcatcctcttagagactttcaacaaggactgagctttgttccaaaaatgtcaaaaagcatagctctaaggaacctctaagtctgagccagagaaggagtcgagaatgacaggacattttcctccgagctctagagtcactggcgtcaaatgctttgcagctgcagacatgataatgcgtcctacacgtgggctgcctgtgaagaatattttatcccattggtgctgcaatagttgctcagcaacatcacttccaccttcaatgatcttaatggcttcagtgtccatataaagagggattgtacgataaagtaaagaagagcatgctggtgctagctcggaaggcttcaccaccactgtgtttccagcagacattgctccaatcaaaggatctagtgtaagactgatggggaaattccaggatccaaatatgagtacagtgccgagtggctctggcaatacttgtgctcttgatggaaacaaaagcagtggtagttggccctttttaggagccatccattccttgatgtgaagcaaagcgtgttctgctgatttcttcaccactccaatctcatcacgataagcttcaacagggtgcttcccaagatcttgctccagggcttcaaaaattcgggcttcattctcagcaataagtttcaaaagagcccgaagctgggcttttcgccaatcgatccctctcgttctcccgcttcgaaaagcctttttcagcccgaatacgctgtccccaaacccaaatccctccatatctactcgctccaaattctcttccaagcatagatttatacacaaatctattcagattttggcaatccctatccaagcattaaatacagtttttcattggtaacgcgaatttcaaccccccgaactgatgatggggacccacaaccaacaccgcaatatatgcgattcaattccaccttgccgaccctgtactccaccatcaagtcagcaaagccgctgattttcacgtgggctggattaaacatgtattgaagtggattatgcagtacttatcaaatctcaatttcatacgacagcaa

>comp28648_c1_seq3 aldehyde dehydrogenase family 3 member f1-like

aatcctgtaaatgttaaacaattttaggtgtcctaaggctttgcagagcagaagtagaggaaaaattgcagagaactaacaaattttctttgtccagtgaaccaccataaacaatggagtctgccacaccaggctcctcaagaagacggcatatcctatcaaaatgatgcttgttcacaattctacaaatactctgcaagttttttacgttttcaccataaaatcttttgatacacctcctcagtaactcaatcagaggtggagcaaacttttgttgtacaaggacataatcaattcctatgcatgcttgtccagcacagggtccccacttcccaccagctattctcttcacagccacctctaagtctgagccagagaaggagtcgagaatgacaggacattttcctccgagctctagagtcactggcgtcaaatgctttgcagctgcagacatgataatgcgtcctacacgtgggctgcctgcagcaacaattaagaagacgaaacaatgtctgaattggcatttgagaaatcaatacataacatggttgaatctcatacatttgaaataaggtcttctacaacaacaatacaaaggcaatccaaatgagagaatccaaattatttttatataccacaatttaaaaagaattgtgtaatctagtcacaactacca

>comp28648_c1_seq4 aldehyde dehydrogenase family 3 member f1-like

aatcctgtaaatgttaaacaattttaggtgtcctaaggctttgcagagcagaagtagaggaaaaattgcagagaactaacaaattttctttgtccagtgaaccaccataaacaatggagtctgccacaccaggctcctcaagaagacggcatatcctatcaaaatgatgcttgttcacaattctacaaatactctgcaagttttttacgttttcaccataaaatcttttgatacacctcctcagtaactcaatcagaggtggagcaaacttttgttgtacaaggacataatcaattcctatgcatgcttgtccagcacagggtccccacttcccaccagctattctcttcacagccacctctaagtctgagccagagaaggagtcgagaatgacaggacattttcctccgagctctagagtcactggcgtcaaatgctttgcagctgcagacatgataatgcgtcctacacgtgggctgcctgtgaagaatattttatcccattggtgctgcaatagttgctcagcaacatcacttccaccttcaatgatcttaatggcttcagtgtccatataaagagggattgtacgataaagtaaagaagagcatgctggtgctagctcggaaggcttcaccaccactgtgtttccagcagacattgctccaatcaaaggatctagtgtaagactgatggggaaattccaggatccaaatatgagtacagtgccgagtggctctggcaatacttgtgctcttgatggaaacaaaagcagtggtagttggccctttttaggagccatccattccttgatgtgaagcaaagcgtgttctgctgatttcttcaccactccaatctcatcacgataagcttcaacagggtgcttcccaagatcttgctccagggcttcaaaaattcgggcttcattctcagcaataagtttcaaaagagcccgaagctgggcttttcgccaatcgatccctctcgttctcccgcttcgaaaagcctttttcagcccgaatacgctgtccccaaacccaaatccctccatatctactcgctccaaattctcttccaagcatagatttatacacaaatctattcagattttggcaatccctatccaagcattaaatacagtttttcattggtaacgcgaatttcaaccccccgaactgatgatggggacccacaaccaacaccgcaatatatgcgattcaattccaccttgccgaccctgtactccaccatcaagtcagcaaagccgctgattttcacgtgggctggattaaacatgtattgaagtggattatgcagtacttatcaaatctcaatttcatacgacagcaa

>comp28648_c1_seq5 aldehyde dehydrogenase family 3 member f1-like

aatcctgtaaatgttaaacaattttaggtgtcctaaggctttgcagagcagaagtagaggaaaaattgcagagaactaacaaattttctttgtccagtgaaccaccataaacaatggagtctgccacaccaggctcctcaagaagacggcatatcctatcaaaatgatgcttgttcacaattctacaaatactctgcaagttttttacgttttcaccataaaatcttttgatacacctcctcagtaactcaatcagaggtggagcaaacttttgttgtacaaggacataatcaattcctatgcatgcttgtccagcacagggtccccacttcccaccagctattctcttcacagccacatcatcctcttagagactttcaacaaggactgagctttgttccaaaaatgtcaaaaagcatagctctaaggaacctctaagtctgagccagagaaggagtcgagaatgacaggacattttcctccgagctctagagtcactggcgtcaaatgctttgcagctgcagacatgataatgcgtcctacacgtgggctgcctgtgaagaatattttatcccattggtgctgcaatagttgctcagcaacatcacttccaccttcaatgatcttaatggcttcagtgtccatataaagagggattgtacgataaagtaaagaagagcatgctggtgctagctcggaaggcttcaccaccactgtgtttccagcagacattgctccaatcaaaggatctagtgtaagactgatggggaaattccaggatccaaatatgagtacagtgccgagtggctctggcaatacttgtgctcttgatggaaacaaaagcagtggtagttggccctttttaggagccatccattccttgatgtgaagcaaagcgtgttctgctgatttcttcaccactccaatctcatcacgataagcttcaacagggtgcttcccaagatcttgctccagggcttcaaaaattcgggcttcattctcagcaataagtttcaaaagagcccgaagctgggcttttcgccaatcgatccctctcgttctcccgcttcgaaaagcctttttcagcccgaatacgctgtccccaaacccaaatccctccatatctactcgctccaaattctcttccaagcatagatttatacacaaatctattcagattttggcaatccctatccaagcattaaatacagtttttcattggtaacgcgaatttcaaccccccgaactgatgatggggacccacaaccaacaccgcaatatatgcgattcaattccaccttgccgaccctgtactccaccatcaagtcagcaaagccgctgattttcacgtgggctggattaaacatgtattgaagtggattatgcagtacttatcaaatctcaatttcatacgacagcaa

>comp28648_c1_seq6 aldehyde dehydrogenase family 3 member f1-like

aaagtaagaaaagagcatttgaaggctttatatgtcacaactcacaagctcttagcatatttctctgatgaaccactaactaatgaaagcaggataaaaagaaaagaaggatcgaatcacatcatcctcttagagactttcaacaaggactgagctttgttccaaaaatgtcaaaaagcatagctctaaggaacctctaagtctgagccagagaaggagtcgagaatgacaggacattttcctccgagctctagagtcactggcgtcaaatgctttgcagctgcagacatgataatgcgtcctacacgtgggctgcctgcagcaacaattaagaagacgaaacaatgtctgaattggcatttgagaaatcaatacataacatggttgaatctcatacatttgaaataaggtcttctacaacaacaatacaaaggcaatccaaatgagagaatccaaattatttttatataccacaatttaaaaagaattgtgtaatctagtcacaactacca

>comp28648_c3_seq1 aldehyde dehydrogenase family 3 member f1-like

gccaatcttatgaactccagcttaaaatcattccaaggagggtaccttggttcgagttccaggtggaagcttctgtggagaactgctttctcatggctgaaagtatcaaatgaatacttcccatggtacctcccgaaaccactttgaccgactcctccaaaaggcagtgaatcacagataaactgaatcatggtatcattgaatgtcacacttccagatgatgtttcctgcagaacttggtccttcagtttttgcttctttgtaaatgcatatatggcaagaggttttggtcttgcattgataaagccgatgctttcttcgattttatcaagtgtaatgattggaagtaatggtccaaagatttcttctgtcataatctgagcatctagtgga

>comp28648_c3_seq2 aldehyde dehydrogenase family 3 member f1-like

gccaatcttatgaactccagcttaaaatcattccaaggagggtaccttggttcgagttccaggtggaagcttctgtggagaactgctttctcatggctgaaagtatcaaatgaatacttcccatggtacctcccgaaaccactttgaccgactcctccaaaaggcagtgaatcacagataaactgaatcatggtatcattgaatgtcacacttccagatgatgtttcctgcagaacttggtccttcagtttttgcttctttgtaaatgcatatatggcaagaggttttggtcttgcattgataaagccgatgctttcttcgattttatcaagctgcagtaagagagattcttatcagatatgaaaacttttccaatagaaagtaaaacattgctggaagttgctaaaacaagttggttttactgtaatgattggaagtaatggtccaaagatttcttctgtcataatctgagcatctagtgga

>comp28658_c0_seq1 calcineurin b-like protein 1-like

tgttttatccagtattatctcaatggtttcatcagccagcttcatctcagactcgcatagaagggcaatcaacatttgcttaacctcttgtcgctcgatatacccagttccatccaagtcatatagcttaaatgaaaaattgattttgtcctcttgtggtgcatttggatggaaaacattgagtgatctaacaaaatcaccaaaatcaattactcctttttgcttcacatcaaatagatcaaaaatctgttctcttgggcatcacagcaaaatatccccggtttgcgaaaagattctcttttttcctgttcttgaacaaagccaattgaagttcttccttattgataagcccatcatcaataacagaactgctaatgctcttaaacagctcaaataatgcttcaacctcgcttacactaaaagccgtttgggaagcaagtagaatggggtcttcatgtccaggaaactgctttcttgaagtagattgaaaacagcccatctgagggaacaaatccccactatacttgagtgtagtgcctcaggcctcacaacaaaacaaaacggaaggggcaaaaggatcaacgcctcaagatgtatggggctcttcaggggcgatatatacatacaattgtatgtatatcatgtaatatacacatatctaagatgtaaagtggctgtgctgataacacattaacacaatctgacttggttgggggcgagagagagagagagaagccaaattggaaacagccgacgaggaaaagagagccaaa

>comp28658_c0_seq2 calcineurin b-like protein 01

ttttcatatatcaccaattcaacaaattaatactcaaaatatcatgaaaatgcaaactcaattactcacccggtttgcgaaaagattctcttttttcctgttcttgaacaaagccaattgaagttcttccttattgataagcccatcatcaataacagaactgctaatgctcttaaacagctcaaataatgcttcaacctcgcttacactaaaagccgtttgggaagcaagtagaatggggtcttcatgtccaggaaactgctttcttgaagtagattgaaaacagcccatctgagggaacaaatccccactatacttgagtgtagtgcctcaggcctcacaacaaaacaaaacggaaggggcaaaaggatcaacgcctcaagatgtatggggctcttcaggggcgatatatacatacaattgtatgtatatcatgtaatatacacatatctaagatgtaaagtggctgtgctgataacacattaacacaatctgacttggttgggggcgagagagagagagagaagccaaattggaaacagccgacgaggaaaagagagccaaa

>comp28658_c0_seq3 calcineurin b-like protein 1-like

tgttttatccagtattatctcaatggtttcatcagccagcttcatctcagactcgcatagaagggcaatcaacatttgcttaacctcttgtcgctcgatatacccagttccatccaagtcatatagcttaaatgaaaaattgattttgtcctcttgtggtgcatttggatggaaaacattgagtgatctaacaaaatcaccaaaatcaattactcctttttgcttcacatcaaatagatcaaaaatccggtttgcgaaaagattctcttttttcctgttcttgaacaaagccaattgaagttcttccttattgataagcccatcatcaataacagaactgctaatgctcttaaacagctcaaataatgcttcaacctcgcttacactaaaagccgtttgggaagcaagtagaatggggtcttcatgtccaggaaactgctttcttgaagtagattgaaaacagcccatctgagggaacaaatccccactatacttgagtgtagtgcctcaggcctcacaacaaaacaaaacggaaggggcaaaaggatcaacgcctcaagatgtatggggctcttcaggggcgatatatacatacaattgtatgtatatcatgtaatatacacatatctaagatgtaaagtggctgtgctgataacacattaacacaatctgacttggttgggggcgagagagagagagagaagccaaattggaaacagccgacgaggaaaagagagccaaa

>comp28658_c2_seq1 calcineurin b-like protein 1-like

gatggaaaaatagacaagtccgaatggcacaactttgtgacccgaaatccttcgctgttgaagattatgacactcccatatttaagggatataacaacgacatttcctagttttgttttcaactcagaggttgatgaagttgctacataaaaattgatttgacttttaccagtttgtgaaccttagtttagaggatatagtgaattctaatggcagcatatatttgacattttgaggagatatttggctctcgatggatgttcaaggcggatttactgcatggtgattttttatgagggttgtggctcgcctctccatttgtagaattgcagtgtatttctaacggtgtattttactagttcacctatcttgatctgccgagaatggtgcttgcgtcaatagaatttaccgtctttttcatggtacagaaattgaagtgataactgccaagaaaaatatttgatcttagactagacgaatgtacgacaagataggttaaagaacgtattgatatttggattagcctgaccaaaaaaattgggttgattgaggaaatttaaaaaa

>comp28710_c0_seq2 er lumen protein retaining receptor

agaagatatgatcaatggcagtaccagaagctaatcacattctctttttagctactaccactatgagtttcaagtgttaacctatgcaagcaaagaacaaagagcaacagttccaaacagaacgagacgggcttgaaactaagaccattaccagcagattcattgatgataaatccattaaagcttaggcacttctctatgtattcgccgacaatacaagtagcatatgggaaaaaacagcgaacccaaaatatagtgttcgataagtagtgcaatcagtgagctaggttaagcaggcgtccagagtttcacagggaaaactgaagagattataagatagaagtcgatatggaagagtagaaaatgagagaatcatggatcttatcgtatttttccagtatgaattattgagccaaagttgattttaggcaggaagcttgagtttctcattgtttttccagctttttatgtagtagtagaagaagtcggcataaagagcagtctgaaccaaacccgatagccaaggaatccagagaactttgggtttctccataaagaatcgatatatccagttaaggatatagaaagcacgataagcactaaaagaggaggaaaagaaaaagaaatacaatgttatatctgcataaatagtgcaaattatgccttcatga

>comp28710_c0_seq5 er lumen protein retaining receptor

agccacatgttagaatacaacatcgcgtagttctacaggtacagtggaagaggttgcatggttcatcaaccacgcaacatctaaccaggcaggataggataaggaggagtagaaaatgagagaatcatggatcttatcgtatttttccagtatgaattattgagccaaagttgattttaggcaggaagcttgagtttctcattgtttttccagctttttatgtagtagtagaagaagtcggcataaagagcagtctgaaccaaacccgatagccaaggaatccagagaactttgggtttctccataaagaatcgatatatccagttaaggatatagaaagcacgataagcactaaaagaggaggaaaagaaaaagaaatacaatgttatatctgcataaatagtgcaaattatgccttcatga

>comp28761_c6_seq1 protein phosphatase 2c

cacctaccctgatgttaatcagcggacaaaatcatcaaatttctcgaaaggaaacttattctcacatatgacaaactgtcaagttgttttggacatgttaagtacaaaactattggttttcaaacttatagttgaataacctgtacaaatcttccaacgataatgctacatctatcaagcacgcaggagatgaagttaaaccttttgagttacatctgtgatcgttgcagttccatcaagtctatacatgcacttacatacgaacccgtcctagtacagtgccgtcagtaacaatgtgctacgaacaagtccgcatcttactaagatccgctcccacctcgcttcgactcagtgtgcgcttcctcagaagctgaggatttctcgtctgaaggttcaccacctgattttttgggtttcttaaattggaccagtatcatggtcatgttgtcgcagccctcgcctatggcagtagaaggtgccaagcacctgtcgagtactttttcgcaaaccagcgagagcttactctcatatttcagctgctcatgtataaaatctactagttgttggcttgacatgcaatcccaaatgccatcacaggcaagcacaatgaactcatcatcatcactgagttcaacctggtttagaaattgaagaaatcacaagtgttataaaaatacaggagccacgtaaagttcatatttttttctcatgctgtaaaagataaaagaacagaataaattttgatcttatgcccaaataatcctgaactatgttaaatcatagtttgataaattaagttcaccatatggctgtttctgttgtctacttctttctt

>comp28761_c6_seq5 probable protein phosphatase 2c 60-like

gatttttccactaatggaaaacacgttcttttcttccatgacataaagtggcttcattatttcaactataaaacagagtgaaaggttgcaaaagataataagcaaaagaagacaaaaacaaggtagccccaatgaatgattaaacatcaaaagcagagacatctgtacaatcttggaagtctgaagggaagaagaagtacacttctgtctgtgattttttcagctccaacaaaattatgtacattttacagattaaaagctctcaacacagcaccacctattgggatcagatgttatgaaacagtctatggcgattatcattcagtatttgttggatttgtctcggcctgcacttcctcagaagctgaggatttgtcatctgaagatgtacctgattgaataggtttcttaaattgcaccaatatcatggtcatgttgtcgcagccctcgcctccagcagtcgatggtgccagacatctgtcaagtactttttcacagaccacagaaagcttactttcagatttcagctgctcacgtataaaatctactaattgctggctagacatgcaatcccaaatgccatcacaggcaagcacgatgaactcatcatcatcgcaaaggtcaacaatgtttatatcgggattggcagtaacaatttgtttatcaggaggtaaaaacttgttctgcttgaattccatatcacctgaaataaacttggggcgatcagtcgtcagaaatcatagaactacctctcctatcagtctatcactgtatatgtctaaattaactatcgaagatcggcagatcatccacaacagtaactaattcattcatcagactgatattacataattgcaactatgagaatagtgcaagcaa

>comp28761_c6_seq10 probable protein phosphatase 2c 60-like

gatttttccactaatggaaaacacgttcttttcttccatgacataaagtggcttcattatttcaactataaaacagagtgaaaggttgcaaaagataataagcaaaagaagacaaaaacaaggtagccccaatgaatgattaaacatcaaaagcagagacatctgtacaatcttggaagtctgaagggaagaagaagtacacttctgtctgtgattttttcagctccaacaaaattatgtacattttacagattaaaagctctcaacacagcaccacctattgggatcagatgttatgaaacagtctatggcgattatcattcagtatttgttggatttgtctcggcctgcacttcctcagaagctgaggatttgtcatctgaagatgtacctgattgaataggtttcttaaattgcaccaatatcatggtcatgttgtcgcagccctcgcctatggcagtagaaggtgccaagcacctgtcgagtactttttcgcaaaccagcgagagcttactctcatatttcagctgctcatgtataaaatctactagttgttggcttgacatgcaatcccaaatgccatcacaggcaagcacaatgaactcatcatcatcactgagttcaacctggtttagaaattgaagaaatcacaagtgttataaaaatacaggagccacgtaaagttcatatttttttctcatgctgtaaaagataaaagaacagaataaattttgatcttatgcccaaataatcctgaactatgttaaatcatagtttgataaattaagttcaccatatggctgtttctgttgtctacttctttctt

>comp28800_c1_seq2 nac domain protein

cccttgtaaactttcccaaaacccctttcctcccacttttctcttagttcacactttccccaaatttttcatcccgaaaaattttcccctttttttctgaattaattaattcaaaaaaaaaaaaacaattttcaatcgagaacataaaaaatggccgccgccatggatttgcagttgccacccggattccgattccacccgaccgacgaagagctcgttacgcattacttggtgccgagatgcgctacgcaatcgatctctgttccgattattgcggagatcgacctctacaaggtcgatccatgggaattgccaggtattggatcggaattcctttcttcttcctgattttgtgacgtttttgtcgattttaatggattaattagttaatgactgctgcattgttgattaatttg

>comp28800_c1_seq3 nam-like protein

cccttgtaaactttcccaaaacccctttcctcccacttttctcttagttcacactttccccaaatttttcatcccgaaaaattttcccctttttttctgaattaattaattcaaaaaaaaaaaaacaattttcaatcgagaacataaaaaatggccgccgccatggatttgcagttgccacccggattccgattccacccgaccgacgaagagctcgttacgcattacttggtgccgagatgcgctacgcaatcgatctctgttccgattattgcggagatcgacctctacaaggtcgatccatgggaattgccaggtaaggctttgtacggcgagaaggaatggtacttcttctcgccgagggaccggaagtatccgaacgggtcgagaccgaatcgcgccgccggcagcgggtattggaaggcgaccggcgccgataagccgatcggacatccgaaggcggttggtattaagaaggctctggtgttctacgcaggcaaggctccgaaaggcgagaagacgaactggattatgcacgagtatcgcctcgccgatgtggaccgctcagctcgcaagaagaacaacagtcttagggtgggtaccccgacctggattggagcttgaaaactgattgaattggaattgaaatgaacggaaatggattcttgattcgacgaacgatgtcgttagattgttttatgaaaaagcggaccaattaaagataatgatgataataattagtaaattaattgttcaattattccttcttatctctttaatttgacattcaaagaatatgattggtgctaaaggtctcagattttccacggtttggattgaacgtgggttctaatctgacggctgtcgtttgatctccacagctggatgattgggtactgtgccgcatatacaacaagaaaggtacaatcgagaaacagcagcagcagcagcagcagagcgtcagcagccggaggatgacgagcgcagtgacgtcagaggaggacacgaagcccgtcatcatgacgtcagcgctcccacaggcgtcgccgatggtctacgacgacttcatgtacctcgacgcatcggattccatcccgcgtctgcacaccgactcgagctgctccgagcacgtgctgtcgccggaggtggagagcgcgccgaagctgacggagtgggggaagcccgagctcgagttcccgttcacttacctggactccgccgacggcgggctgacggggtcgcagttccaggcaaattatcatgtggatccagtgcccgatatgttcgcgtacatgagcaaaccgttttgaagtgggagat

>comp28805_c0_seq1 dnaj-related protein scj1-like

gtagtataaatggcaaaaatcatgtcatgtactacaagtgctagcagttttaccagttatccctcaggttggcgatcatgagatatacaacaaaatcatagcatatgatgcatcctactctcactcttttacacatgacagaataccaatgccaacaagtttcagttgtccaactgcacataacatgcattgcgatctgagttcatgctctgcagttctgtataaaagcctgctgcatccaagagaggcaaatctgtccacttcaaaaaacatagtgcaagatgcataaatcaccacatcgagcatttcatgccaagaccattgcacgttagaataaggctcttatagcgattaaggtactccgcaatatcgctatcatacgaataaagcatccctttcttgtcatactcatgccgttttacaggatcactcaatacaagcaaactggcaggctgaacaagtttgctgacattgaacataacgtaacatatcctccaattttagctggcttcaaaccctgataagcttcattgatttcttgaaattttgaagtggcagtgtcctcacccatctttttatcaggatgccacttcaatgcaagacggatataattggaacgaatggcttcctctgaagcatcgtaatccaattctaaaatcttgtaataatccttaggttttgataaaacggagaaaaaatcgaaattgacatgagaatcgtcttgggattgttcttggtctggatgctggtcgaaatcgtaccattcatcccacatcat

>comp28805_c0_seq2 dnaj-related protein scj1-like

gtagtataaatggcaaaaatcatgtcatgtactacaagtgctagcagttttaccagttatccctcaggttggcgatcatgagatatacaacaaaatcatagcatatgatgcatcctactctcactcttttacacatgacagaataccaatgccaacaagtttcagttgtccaactgcacataacatgcattgcgatctgagttcatgctctgcagttctgtataaaagcctgctgcatccaagagaggcaaatctgtccacttcaaaaaacatagtgcaagatgcataaatcaccacatcgagcatttcatgccaagaccattgcacgttagaataaggctcttatagcgattaaggtactccgcaatatcgctatcatacgaataaagcatccctttcttgtcatactcatgccgttttacaggatcactcaatacctgataagcttcattgatttcttgaaattttgaagtggcagtgtcctcacccatctttttatcaggatgccacttcaatgcaagacggatataattggaacgaatggcttcctctgaagcatcgtaatccaattctaaaatcttgtaataatccttaggttttgataaaacggagaaaaaatcgaaattgacatgagaatcgtcttgggattgttcttggtctggatgctggtcgaaatcgtaccattcatcccacatcat

>comp28808_c3_seq1 thiazole biosynthetic enzyme

ataaaaggctgatatatatatatatataaaattctttccatggtgaatgaaaatcatgaacaggaaagccaagaagatagcgtttcaagcaaaatccatactccatctccacatttcaaccagcccctaacatttaatcaaactatcttcttatttctgtttatttcagagtaatcatctacattatgcatctacaatctcatcggactccgccgaggccagaataaattccggctgagcggttttcacttcgctgtaagatccatccaatgcgtttggctgtcccaaagccctcagtgccaagtgcgctgctttctggcccgatatcatcatcgccccaaatgtcgggcccattcttggtgctccgtcgatttccgcaacttccatgccggtaacgatcattcctggcacaatctctctcgtgagcctaacaatggcgtcctcggcggtgttcatgtccagggccttcattccgggcacgctgtcgatcattccgatgctcttcagccgcttcacaccggtggcgccgaagggtccgtcatggccgcaggagctcaccaccactttcgcctccattacattggggtccatgcacgactgtgtgtcg

>comp28869_c0_seq1 stromal 70 kda heat shock-related chloroplastic-like

agcatccttggttgcagtcctttgagagtcgttgaagtatgcaggaactgttactactgctttagtaaccttgtcattcaaaaactttgatgcatcatccacaagcttcctcaaaacctgagcggatatttcctcagcggcaaactgctttccaatggcagggcactcaagcttgacattaccattttcatccctaaccacatgatacgaaacctgcttcgactcatcatcaacttcggacattttcctcccaataaacctcttgacggagaagaaagtattctcagggttcaccacagcctgcctctttgcaatctgccccaccaacctatcgccattcttagtataagcgactacagatggtgtcgtccgctggccctccgcgttggttacaatggtaggcttccctccttccatcgccgccaccgcagaattcgtcgtccccaagtcaattccgacaacctt

>comp28869_c3_seq1 stromal 70 kda heat shock-related chloroplastic-like

agcttccgccttgatggaattccacctgctcctcgtggagttccacaaattgaggtcaaatttgacatagatgcaaatggaatcctctcagtcactgctgttgacaaggggactgggaagaaacaagatatcactattactggtgcaagcaccttgcctagtgatgaggtggagagaatggttagtgaagccgacaaatttgccaaggaagacaaggagaagagagatgccatagataccaagaaccaggctgattcggtagtttaccaaacggagaagcagttgaaggaacttggggacaaagttcctgcccccgtaaaagagaaggttgaggctaaacttggagaacttaaggatgctatttctggaggatcaacccaagcgatgaaggatgctatgaccgccctgaatcaggaagttatgcagcttggccagtcactgtacaaccagccaggtgcagcacctggtgccggcccaacacctggaggcgaactacaacggtgaa

>comp28869_c4_seq1 stromal 70 kda heat shock-related chloroplastic-like

gcaatgaaactattctggtttttgaccttggaggtggtacttttgatgtttcagttcttgaggttggtgatggtgtgttcgaggtgctctctacttctggagatactcatcttggtggagatgactttgacaagagagttgttgattggcttgctgctaatttcaagaaggatgaagggatagatctattaaaggacaaacaagctcttcagcgtttgacggagactgcggagaaagctaaaatggaactctcatctttgactcaaactaatattagtttgcctttcattactgccactgcagatggccccaaacatattgaaactacacttacacgggccaagtttgaggaactatgctcagatttgcttgacagactgaaaacacctgttcaaaatgcattgagggatgcaaagctctccttcagtgacatagatgaggtaatcctggtgggtggttccacacgtattccagccgtccagcaacttgttaaaaaaatgactggaaaggacccaaatgtcactgtaaatccagatgaagttgtagctcttggagctgcagttcaggctggtgttttggcgggcgatgttagtgatatcgtccttctggatgtatcaccactatctttagggctggaaactcttggtggagtgatgacaaagattatacccagaaataccacattacctacttcaaaatcagaagttttctcaacagctgctgatggtcagaccagtgttgagatcaatgtccttcaaggtgaaagagag

>comp28971_c0_seq1 bag family molecular chaperone regulator 7-like

ctgccgctggttctgcagaaccaaaaccgacgtgtatgtatatgcatatagagacagataaccagctctatcgagagggttctctgaaaacaaaaaatgagcagattcggaagatttgacattgttgaacgctgtccatcctacttcctcaatgaaacaaccattctcacacctccaaaaaccctcttcctaaacccttgcttctctgcatttccctttgttgaagacgagctcgacctcctggaccttcaccatcctccatctgctttgttcgatgaatttgacacagtcacggatctgatccagaccgaaataacccgattccgcacctcaagtctccgggtctcccgccgggtcggactcggtgagctctacttgcaagctctatgcgatcgcgtctcggatctggagtcgggttttgagcggctgctgagggaggagaaggccagtaagaagaggattgatgagcggaagtacacttggacggctgagataaagacgccggagaaagaccggaagtacaaatggacggcggagattaagaacgggaaggatgagaagaagagtttggagaggagctacaaatggacggcgcagattaaggggaacggcggcgactacctgccgatcgagcgcacttacacagttaaggtgtccggtgacgagagcagcgaatcggaaaaggagggaaagaagaagaaaagaacgacgaaagttgagaaagtgaaggagaagggtaactcaaatgctcgcattgttgagattgaagaaccatctgatcatggtggcattgtcctaagacaggcttttgccaagagactggaaaagaaaaagggcaagagtaaggagttgtcaccacaagatgcagctactttgattcagatttgtttccggacttatctgattcgaaggtctcaggctctacgtgccctaagggagctggcaattgccaagaccaagttgaaggaggttagggctttgttcaacaatttctcttatcgtcgtcgtctaacccgtgatgcagaagagcgtcagaggttctctgaaaagatcattgtactgcttctcactgttgatgccattgagggtgctgatctgatggttcgagctgcaaaaaactcgatggtggatgagctggaagcaatgcttgatgcggtggaccctcaacctcccggaaaatctttgtccatgaggaggagaacgtttgatatgcctgatggtgtgatcaacaaggaacttgctgcaggtgttgcacaggtggttcgaatgcttgatgaagaatctggttcttaaacttttgaagcatgattgtaacttaagtttgatatgccaaacaatctagggcaatgttgtatgccttagtgtaagtttggattagtatcatcttgtttgtcaaatatttgagaaatggatgctagtatgacaagcttggtagggtttctacttttcaagtacgttctgtctgatttaaatatgagatacttgtttcctcggcatgcttcagcctatgcatctatggttggatattcgttgtatgacgttttctatccacaatg

>comp28978_c0_seq1 phosphatase 2c family protein

gggacattttgtggcaaagagtgttagggagtcgatgccttcatctcttctttgcaattggcaagagacgcttgttgaagcctcagttgattctgattttgatttggaatccgagaaaaagcttcacggattcaatatatggaagcattcctatttgaagacctgtgctgccgtagatcaggagctggagcaacaccgtaaaattgatgcattttacagcgggactacagccctgacaattgttaggcagggagata

>comp28978_c2_seq1 protein phosphatase 2c 73

tcacgttaaaataacatccagacaaagcaccaaactatggattttgatgcttacgcaccttcactctctcgatatctcagctcaaaccgtcggaatcccgtcattttccggcgactagcgtcatctttctggccagttcttggtttcccggcgtgctgacattccattttctctaaaattcctgtctcaaaattgaaggttgtaaggaagtggcggtggtgagctatggggcatttttcttcgatgttcattgggctggcgaggtcactttcgatcaggagaacgaagggtttgaaggacaattgtgcaaattgtgatggaagagaggccgttaaggctatggcaaaggacgcgaagaaaagtgacttgatattgacgacatcaggcattgtcaatgttgatgggtcgaagaatttggcttctgttttctcaaagaggggagagaaaggtgtgaatcaagattgctgcattgtttgggaggaatttggatgtcaagaagacatgatattctgtgggatatttgacggccatggtccttggggacattttgtggcaaagagtg

>comp28978_c2_seq3 protein phosphatase 2c 73

gagtctttcgatgtctgtttgttcgatcatcctctgacattttctgaatcaccagacatggtgacatactcgtattgttggaattccatctaagattgcaagattctctgctttttccgcacaggttgtaaggaagtggcggtggtgagctatggggcatttttcttcgatgttcattgggctggcgaggtcactttcgatcaggagaacgaagggtttgaaggacaattgtgcaaattgtgatggaagagaggccgttaaggctatggcaaaggacgcgaagaaaagtgacttgatattgacgacatcaggcattgtcaatgttgatgggtcgaagaatttggcttctgttttctcaaagaggggagagaaaggtgtgaatcaagattgctgcattgtttgggaggaatttggatgtcaagaagacatgatattctgtgggatatttgacggccatggtccttggggacattttgtggcaaagagtg

>comp28978_c3_seq1 probable protein phosphatase 2c 73-like isoform x1

gaaattgatcagatgaaggtgatgaatgaaaaaaaagtacaattgctgatatatcatccgtagctattccctttctcttctttttccacgcatgagcagcgcactcaaccagacgcttagcagattttgcccggtcaggagtcgtcgatacaatttgcaccgcttcttgatttgatataacatcccaaaccccatctgttgccagcacgatgaattggtctttactcgttatatgtctctgagtaacttcaggcaccgaaataagtccaaaattcttcacacaatagtccccaaacgctcgagacattgctagtccaggagattcttcatgtggcaaccacactctgtgaaaccctggctcatcatccagacaatacactctgccattgcactgaattattcgctcagcctcttgaggaagattgggcttgaaatccactgtgagctgcactgctaccacggttccgttgtcagaagtggtgcccagcacagcacgcgagtctccaacatttgctaagaaaacagtatctccctggcagaacattaaaa

>comp28985_c0_seq1 uncharacterized loc101214727

atttgcaagccaagtggcagtctccatatcacaggaaatttgattctgatgaacagaactgcaacacgactgcacctgtagaacacaagaagtgcgctggacatcttaagagactgcattcttcacttttgtccgagattgaaaaagagcgaacgctgaggccctttaacggtgataggagggtgaaaaatgaaaggcaaaagtatcgagctttccattacggggtatagaaccaattttggacggatgagaaattttgagttggcgacattttagaaataaattttgtgggaatgatgtaagttcctcttaagttgaaaaatgtagtgatcatgtacaattagtactgcgctgttacatgttactttaattttttttaatttttaattattattattattttttccttctctctgccagtgaaggaca

>comp29013_c0_seq2 universal stress protein

atctcccttagatgccataaaactgaaagagttgtgtttgcaaagcagtttgaacaataacttacaccacactgaaagcggtgtgatgttttcaggaactaaaacaaaagacagaaactcagtaacaatgatgatcattcaaacttcagagagaaaatacagcaaccaaagcataaacagaaagaaaaacctcgacacggcacaaaatctctctcacacaaacaaatatccatgatgatcatttcaccatctccggaaaacccacctcctccaatcatcagctcaagaagtcttttccacatctgcattcttatcagaagcatcaagatacaccggctcttcctcctcggggacggggtgcagcccagaagcatcctcatcaacatcctttgccaaacccccgctttcataggcagaatccacatctttctcatctggatacctcaccacaactacagggcaaacacagtgatgcacgcaataatcactcacactgcccagcctccctttactgctcctcctggacgctccaaatcctctactccccatgatcactgcacttaaccctagcctctccacctccaagcacagcctctctttcatatcatgatccttcacaatatggatcttgaacggaatattcgcttccaccaagggctgcgccaagtcatttgctttaatggtagtgaaattgtcgaaatcgtcctcgagtttctgctgcgatttctcgtcagtgttgtcgaccgagaggtcgatggcgccccaatcagcaccgtataggacggaggtcggccgtacgtggaggagaatgacggcgtcgcccgggcggagataattctggacagcccacttaacggcgaaggcgctctcgtcactgagatcgacggcgatggcgatcttgcgctgtgcgccggcggttggagttccggaggggaagcgaggagaggctggctgatggagaatcgcggccggtgggaggtcgttctccacctgaggtttctttggtgatgccatgcagtaaactatgttgattgtggtatacaatttgggggttttgggggtttgtgatgaacagttcgatttgcaaatgaatagggtttgacgattcgtttgttaattttcgcgacttatggtttttgctctgtttgcaggagtacgttcactgtgtgaattcgctcccgaataagaaaggttgggaacagga

>comp29039_c2_seq1 leucine-rich repeat receptor-like serine threonine-protein kinase bam1-like

gcaatatatccataagaaccagcaatggctgacatgcattccgatgccccagagtcttgcaagaacttggccagtccaaaatcagctacatgagcttcaaaattggaatccaggaggatattgttcgacttgacatcacgatgaacgatcaacggtgagcaatcatgatgaagatagcaaaggccctttgccgcctccacggctatgttataccgtgtatcccaatgcaaatgacctcctttcttaccatgaagcatttcccccaagctcccgttaggcatgtattcataaaccaaaaggtttgtttcatgatttgaacagaaccccaataacctaacaatgtgcctgtgcctgatcctccctagagtctgtatctcggcattgaacccatgatcatgagaagagccacgactcattgctggcaaccttttcacagctacctgctctccatttggcatcactcctttgtaaacaataccagcacctcctttaccaataatattatcctccttaagactatccaagacatcgtcacaagtaaaatccaaccgctggaaggcagtaagcttccaagcacgagcatcgctagccttcttcaatgaccgagccttgataatcgcagccacagcaaaaacaatggaacaaacaagcaacccaatcacaagcaaaagcttcatggaaggagagaaagtccccttctcatgcggcctttcagccccactagcaacgccctctttgcaaggacccaaatacgggccacaaagattcggattacccaagaacgaagtgtaattgaaataactaaactgaccagtcccaggaaccagaccagacaaattgttatacgaaaaatcaacagaagttaagctctgcatactagcaatggaggacggaatactacccaccaaatgatttctcgacaaattcaagtagttcaaaatcctcataccagtaatctctgctggaatttcaccggagagctgattgtggctgagatcaacgaatgtcaacagcttgcattggctaatctccggcgcaatggggcctccaaattcattgtggctgaaatccatctttgacaactgctgcaacttccgtatttcagctggaatccggccggaaaacttgttcccgtcgagcaaaagcttctgaacaccaacaaaattcccaatacttggcggcaaaagcccggtgagatggttgttcgaaagactaatttgcccaaggctcgtcgacgatccatcagtctccggaaaagcaccagacaaaaaattattttgaagctcaacttgagttagtttgggcaaactcaatagccctttcggaatggacccgtttaggtagttttcgcccattcgaatccgggtcagcgaccggcactgtcccaatgactcaggaatcgggccgaagaggaagttgcccagagtgatcaatgtatgcagctgattgccattgcatagatttggaggcagatttccggtcaatttgtttgaactaacatcaacttcttgaagttttccattagttcccaaactctgcggaatgatttctgtgaaattgttctcccacaactgcaaaacttgcagctccggcaactctccgataaaattcggtatcgagccggtaagcttattccggaaaagatttaagagggttatattttttagctgcgcaaacgacggcggtatttcaccggaaagcatattgtttgacagatccatggatttcaagctcttcaagttgccaagctccggtgtcaagccaccggaaagcccatttacttggaggaaaagagtgtcgagattctgcaaattgcctaactccggcggtatctcgccagagagaccgcaatttgcagcgtccaaccgcaccaactgagacaggtttcccatctccctaggtatttcgccggaaaatgcgttgaaatacccaatgtaaagctccttaagctgagtcaagtttccgatttccggcggtatcttaccggtgagctcgttaccggaaacagccaggtattcgaggtgcggaaacgagccaaactccggcgggatttcaccggtgaagaaattcccaccaagatgcaaatggcggagattggtgagcaaatacgcctgcgaagggaagtcgccggtcatgttattattgtaaaggtcgagaacttcaaggtttctgaggcggtaaagctgcggcgggaggctctcgttgaagatattgttggaaaggttgagatatcggaggttggggatgtcggagatttgtggcgggacagggccggagatggagttggcggcggcagagaagttaacaaggaaccggagattgcccacatccggagagaggatgccgctgaggctgaggttcga

>comp29039_c3_seq1 leucine-rich repeat receptor-like serine threonine-protein kinase bam1-like

gaggctgaggttcgagatgtcgagcgctgtcacgtgccggcctgaggggtcgcacgtgactcccttccacgtgcaatggttggtgtcgggtttccacgacgccagggcggactgcgggtcgtcggtgatggcggctttaatggagagtaaggcgcggtattccggcatccgcggcggtcttgcggcggaggaacggctgaggagaaggtggaagaggagggaaagtaggaggagacgcatttttttgtgtgtttttcccagtgtatttgtgctttttttttccgtttctgaggttatttgttttgaaagcaaaggaaacggttggggggtgggtagtgggagagt

>comp29048_c1_seq1 plasma membrane h+-atpase

ccctcatcccgatcgacacgcgtgcagtaaaatacagcgttgattgaacacaatataacgggatgattgaagcacaaaactgaaccagggacaaataagagacttctgagttaaaaaagtgggttacaaggagatgccattgtactcataaatgagtcaaagatcagatagagaccctatcttgagatatattaggaaacaaaaattaaatagacgaggaatgatggcacgttactgccttcttccgcctaagaacagttttatctaaaaggcactcgtctgttctttaaatcatttgcttgttttctggagcagttcttcgtgaagatttcatttggcttcttcaaacggtgtaatgttgctggattgtctcgatgtcgaggcctttaagcttcacaacagactcaacgtggcccttgagtgtatgcaactcacgtagcctggcaatctcggctctcctcttggcctgttctgcaatttctgacaattcgcgatagctgctcttctcgttgaagagattagtagcttctggtgtttgaaggccatgcagagtcctctgggcaagagcccattgagcttctctctcctcttttccataatctttcttggtggtgaaagcagtcttgttgtcatacaggttttgccaagcctttccacttaagatgtaacggatagcgaacttcattaggtcaaggggcacatagaagacaacactgtaaatccagattacgccagcccatccccaaccacagcctttaattcttgcaaaaccccagtttgcatacacagctattagagttgcaaccatttgagcaatgacgaaagcagtgactaggagaagtccaggacgttcaacaaatgaccaactccgcgacctggtgacgaaaataagggcttggctaacaatactcacttgcaagtatagagcagccatcatttcgtgctcactatgtttaatgtttctaactccaaatttctccgggaagaagtcagttttatgcatcaaccagaagaaaagaatagtcatcaatgcaagataacctccaagtgctatgccggtggcaaaaatttctttcaacttccagctatcaggcaatggggatggcttcactctatccttcgagatggtcataatggtaccgtcatttaggatggcaatgatcaaaaccatgaagggggagaaatcgaacttccaaatcaaggcaataaacatgaaaccaaacacaatacggatggtgatggacaccgcatatattgtataattcttcattctctggaaaatagctctgctggtcaacactgcactaatgataacactaagtccaggttcagtaagcacaatgtcagaagcacttcttgcagcatcagtagcatcggcgacagcaattccgatatctgccttctttagagcaggggcatcattgacaccatcacctgtcattccaacaatgtgtttcctctcttgcaacttcttcactatttcatatttgtgctctggaaagaccccagcaaaaccatctgccttttcaatcagttcttcaacaggaaggccagctatagactcatccttatgttgacccagtaaagaagcagatgggtacatatttactcccattccaagcctgcggccagtttccttagcaatagcaagttgatcaccagtaatcattttgacattcacaccaagattcagagcccttcggatggtttccgcactatcatgtctgggaggatcaaaaagggacaagagtccaacaaattgccatgggccaccagggctatcctttgatttctctggaatttcctgtctagcaacagccaaagaccggagcccacgctccgcaaatttatcaataacactgtgaaccttcttctttatgtcttccttgcagttgcagagagccaaaatctgttcaggggctcctttgctagctcgatgccagttcccattagagtcaatgtaagtcaaagcagtccttttgtccacagggttgaaggggaagaaatgaacctctctgatgccagctcttgcctcctttggatcagcaagtgtaccaacaatggcagcatcaatggcatcttgattttcaactctggaagcccttgcagcaagaagtagcacatgatcaggatccacacccttcgcaaaaacctcaatcaagcttttatcaacagtcagtttatttagtgttaaagtgccagtcttgtcactgcacaagacatccattccagccatttcttcaatagcagtcattctctttgtgatagccccctgctgtgatagcctgtgggatccaatagccatggtgacagacaagacagtgggcatagcaatgggaatgcctccgatcaaaagaacaagaagattatctatgccttttctgtactccctgtgctgaattgggtacattactattatctcggccatcataccgacagcaatggagcaaatgcagaagttaccaattgcagtgaggactttctggaagtgccccacctggttggtgctgtccacaagatgtgctgcctttccaaagaaagtatggacaccagtggcaatgacgactgcttcaatctcaccttgtttgcatgttgaaccggaaaaaacttcgttgtaggggtgcttggtcacaggaagcgactctcctgtcagagcagattgatccaccttcaacggatcaccttcaagaagacgagcatcagcaggaatgatatctcctaatttgatgcttataatgtctccaggaaccaaaatggctgcttcttgctcactccatctaccatctctgaggaccttagttttgggagcaagaccagccataagggctgcagcagcatttccagcattgttttcttcaatgaaactgatggtcgagttgataagaagcaagcatacaataccaacaaagtcttgccaatccgggggcttgccgcctccatttgccagcacaattgccatcaaagctgcagcctccatgacccatgagagtggattccacataaaaccgaggaacttgagtattttgctttccttcttctcttccaacttgttgggtccaaaaatctgaagcctgttggccccttcttcggaactcagtccctctcgggtgcatttcaactgctcaaagacttcctcaattggaactttttccagatcaacagtctcattcttgatttcttcaaggctaatacccttttcaccaaccatgtttggtcacagcactcacagcgactctctcccctgctagatcagctctttttcaagatatgagagagaaaggaagaagaaga

>comp29048_c1_seq3 plasma membrane h+-atpase

gactgcatatatctgccaaagagtaacgtgaaatataaacacttgcttcaactgaaaaaccagttgagaattgaagaaagaagacagaaactaacggtgtaattcttcattctctggaaaatagctctgctggtcaacactgcactaatgataacactaagtccaggttcagtaagcacaatgtcagaagcacttcttgcagcatcagtagcatcggcgacagcaattccgatatctgccttctttagagcaggggcatcattgacaccatcacctgtcattccaacaatgtgtttcctctcttgcaacttcttcactatttcatatttgtgctctggaaagaccccagcaaaaccatctgccttttcaatcagttcttcaacaggaaggccagctatagactcatccttatgttgacccagtaaagaagcagatgggtacatatttactcccattccaagcctgcggccagtttccttagcaatagcaagttgatcaccagtaatcattttgacattcacaccaagattcagagcccttcggatggtttccgcactatcatgtctgggaggatcaaaaagggacaagagtccaacaaattgccatgggccaccagggctatcctttgatttctctggaatttcctgtctagcaacagccaaagaccggagcccacgctccgcaaatttatcaataacactgtgaaccttcttctttatgtcttccttgcagttgcagagagccaaaatctgttcaggggctcctttgctagctcgatgccagttcccattagagtcaatgtaagtcaaagcagtccttttgtccacagggttgaaggggaagaaatgaacctctctgatgccagctcttgcctcctttggatcagcaagtgtaccaacaatggcagcatcaatggcatcttgattttcaactctggaagcccttgcagcaagaagtagcacatgatcaggatccacacccttcgcaaaaacctcaatcaagcttttatcaacagtcagtttatttagtgttaaagtgccagtcttgtcactgcacaagacatccattccagccatttcttcaatagcagtcattctctttgtgatagccccctgctgtgatagcctgtgggatccaatagccatggtgacagacaagacagtgggcatagcaatgggaatgcctccgatcaaaagaacaagaagattatctatgccttttctgtactccctgtgctgaattgggtacattactattatctcggccatcataccgacagcaatggagcaaatgcagaagttaccaattgcagtgaggactttctggaagtgccccacctggttggtgctgtccacaagatgtgctgcctttccaaagaaagtatggacaccagtggcaatgacgactgcttcaatctcaccttgtttgcatgttgaaccggaaaaaacttcgttgtaggggtgcttggtcacaggaagcgactctcctgtcagagcagattgatccaccttcaacggatcaccttcaagaagacgagcatcagcaggaatgatatctcc

>comp29048_c1_seq12 plasma membrane h+-atpase

atctttcttttcattcacctcaagattttttccttttcatcgattttgtttggatacatcttttcttacatctaaagatcattcatttactcgatgcactaggtattgcataagtactatggactatctgtattagtcctcaaactgttgccacaacacaagcatataagactagggcaacaaactagagttttgaaaacatagaaacatcattaaactcatcatagaaagagccaaagatcagcggttatggctctttcctgcctcattcctcttacaaggcagagttttatctagcaatacaactccttttgcagtacgagttcctcctgtcttgtggcattcgttcgccttcttcaaacggtgtaatgctgctgtatcgtctcaatgtcaaggcctttgagcttcacaactgactcgacatgacccttgagtgtatgcagctcgcggagccttgcaatctcagctctcctcttggcttgttctgcaatttcagacagttctctgtaactgttcttctcatggaagatattactagcttctggtggttgaaggccatgcaaagtcctttgagcgagggcccattgcgcctctctctcctctttgccgtaatctttcttggtggtgaaagcaatcttgttgtcgtacaagttctgccaggcctttccacttaagatgtagcgtatggagaatttcattaggtcgagaggcacgtagaagacgacgctgtaaatccagatgacaccagcccatccccatccacatcctttaattcttgcaaatccccagttggcgtagacagcaataagagttgcaaccagttgtgcaatgataaaagcagtcattagcagcaagccagggcgttcgacatatgaccaactgcgcgacctggtgacgaaaataagggcttggctaacaatactcacttgcaagtatagagcagccatcatttcgtgctcactatgtttaatgtttctaactccaaatttctccgggaagaagtcagttttatgcatcaaccagaagaaaagaatagtcatcaatgcaagataacctccaagtgctatgccggtggcaaaaatttctttcaacttccagctatcaggcaatggggatggcttcactctatccttcgagatggtcataatggtaccgtcatttaggatggcaatgatcaaaaccatgaagggggagaaatcgaacttccaaatcaaggcaataaacatgaaaccaaacacaatacggatggtgatggacaccgcatatattgtataattcttcattctctggaaaatacatctactggtcaagactgcactaatgatcacactaagtccaggttctgttagcacaatatcagaagcacctcttgctgcatctgtagcatcagcaacagcaatgccgatatcagccttcttcaaagcaggtgcgtcgttgacaccatctcccgtcattccaacaaggtgtttcctttcttgcaacttcttcactatctcatatttgtgctctggaaagaccccggcaaaaccatctgccttctcaatcaattcttcaacaggaaggccagctatagactcatccttatgttgacccagtaaagaagcagatgggtacatatttactcccattccaagcctgcggccagtttccttagcaatagcaagttgatcaccagtaatcattttgacattcacaccaagattcagagcccttcggatggtttccgcactatcatgtctgggaggatcaaaaagggacaagagtccaacaaattgccatgggccaccagggctatcctttgatttctctggaatttcctgtctagcaacagccaaagaccggagcccacgctccgcaaatttatcaataacactgtgaaccttcttctttatgtcttccttgcagttgcagagagccaaaatctgttcaggggctcctttgctagctcgatgccagttcccattagagtcaatgtaagtcaaagcagtccttttgtccacagggttgaaggggaagaaatgaacctctctgatgccagctcttgcctcctttggatcagcaagtgtaccaacaatggcagcatcaattgcatcttgattctcagttcttgaagctcttgcagccagaagtaacacatgctcaggatcggctccctttgcaaagacctcgatcaagcttttatcgactgtcaacttgttgagggtcaaagtcccagtcttgtcactgcacaggacatccatgccagccagttcctcaatggccgtcattctcttagtgatggcgccctgctgcgatagcttgtgggatccaata

>comp29055_c0_seq1 brassinosteroid signaling positive regulator-related protein

tgaaaccaaatggatttgctgaaacaaggctgaatgttggagaagaaggcccatcttgaggagtctgtacaccagaaagccatcctgaatcaggtggagtttgcagaccaggactctgaggagtagatgaaggtaaaaatgggaagttctgcccatgccaagcagtaccagttgccgggtcatcccagttacccttcattcta

>comp29055_c2_seq1 bes1 bzr1 homolog protein 4-like isoform x1

gcgtatttcagtttccctggttcagcacccacatacttctattttcagatatcagaaggatcgactctaattagtggtacaacaatgtgcaataacagatgacagcatctaaaaatatttctgaatattttagccgtttcttccatgacctcttcacctggtgctagaattcccaagtgtaagctcaaggtcgtcaggaacacactcatcatgtattctctcaccttcccatggtttcactaatcccttgggattactaccaaatgcaaactcagcagcgattgcatctgacatcagaacatcagctgtttggtcaatccctgctctaatggccggagagcatgttccactttgtccaggggtccacatacgagagcctccatttgacaatggttccttgaaaccaaatggattt

>comp29055_c2_seq3 bes1 bzr1 homolog protein 4-like

tgttctcttcttccaaataaacggaaagaggtagagtgaagctcgttatataaaatacctggtgctagaattcccaagtgtaagctcaaggtcgtcaggaacacactcatcatgtattctctcaccttcccatggtttcactaatcccttgggattactaccaaatgcaaactcagcagcgattgcatctgacatcagaacatcagctgtttggtcaatccctgctctaatggccggagagcatgttccactttgtccaggggtccacatacgagagcctccatttgacaatggttccttgaaaccaaatggattt

>comp29055_c3_seq1 bes1 bzr1 homolog protein 4-like isoform x1

cggtgagcttaatggaggtgtgactggagcacttatagaacctcctgggatgtaaggataatggggaagcttagatgaagctggcaaagtgcctgatgataggtttttaagccaggggataagggagttgggatcagcagtattattggcattggcagcataatgggatgagacagggcttggaaaagaagatgatgcagggcttggaaaagaagatgatgcagggcttggattgaaagaggctcctgggcttggttgatatgatgtgcaagg

>comp29079_c1_seq1 high-affinity nitrate transporter -like

acttgatcttcactgtcttgtagcccgaatctgtcccggctgggaggctggtgttgagtgaccatgtcactgtgatattgtcttccccagcgttaagaacttgtcctggtttcgtggaggcagagacgatgagagtattaggaagagaagagaaggtgacgccgtaagaagtgtctgcaagacaggagagaacaagggaaactacaagaaaatcttgaactgccattgatgatttcttgtgttccagattgagccaaatgaagtagcctttgccttaatccaaatgaggcttaaagtggttgagttgatga

>comp29117_c0_seq1 mitogen-activated protein kinase kinase kinase 3-like

aaggagctaacaacagatcaaacacttgcaaaaaccaaatctggaatcatatataaataaaactgcctttttaatcgcataaaagatctaactgccaactacaatttatacacactaccaccagtcacctcgtcctttaagaaaaatcatgctgaagggatacttgcaaatgtaatacaacatgccatgactgagttccaaggcaattaattttagttagagacgcaatcaacaagatccagcataagtttacagttaatgacgacatctatgaaccataaaagtagttcttatcaatttccttctagcagttattccataacagatccagtacaacaagcagatctggaagaggtgccattttcaccatctggaaaggtcactccaattagttactatacatatcaacggccctccaaatttcatggcatgggaagattctttgctccattgtgatagtttaacccttttttcccaattccacatatgtgaacacgcatttcacatttcccaacaagctcaaagatgcagatatctccaatatcaatattattattgcgaacaaaactcaaccacccaccacagaaagtatgtgatgtttggacttttgtagttggaatcgaattaaccgtccaactttctccttttaaattgcgaagcacaatctttactttgcatttagggaggtgttctgttgcaaactggtaggggacattcagagtgtaagaaccacttatgttgaatctcttcatgactttcaaaaagctggggaaagacgatttaaatgatcgggctattctttctgcttctgatgctgacaatagcatatctagcttggtggttctatttctggtagatggatctgggttgtcacgtggaactgctgtaagaggtaaagcaacagtgacagcattcttcatgttactgttgccatcagcttgttgcatccattcttctgcattgaccaaatttgcatggttttcgtgattatcacacatgctttcagagtgcagttgagagcatctcatctttttgggaacaccttcaatgatgctatctaagagagctgatctttccctgtctctttttttcagcatattactatcatatttacttaaatcttggctgcttgcagcaataaatgcagcttccttttcgcacatactccggtcaaaaatttgcaatgtgaaattcaaatctccatcatacctaaaaactattgaatcaccttgttcaagaaagtgatctttaacaaaatcggcccatccatcatcgaggaacaaaccattttcaagcataattaagtccacaaaccaattattcccactaggacctaccaaaactgctgggccaggagctgttccttccatatattttacaaagttacatggtatattcagtctttcagagtaaagagaagggtcgaaagccagaagaaagtgaggcctcctcatatcccacatctctctctctctctccccccacaagtaaaaacgcagaagctagattgattcaagaaaattcaaagagaagtagaaagcccttttgatttctagatatggattggattcgcggtgaaagattgggccatggaagttttgccaaggtcaatttagcagtgccgagaagacagagttcatgtcttcctccattgatggcggtgaagtcttgtggagtttcgcactcttcgtctctcgtcaatgagaagtttatattggaggaactcaaagactgcccggaaatcattcggtgcttcggcgagagtttttccgacgagaatggcgagaagttgtataatgtgttgttggagtatgcttccggcggctctttggctgataagctcaagaattccgacgatcagaggctgccggaatctgaagtgcggcaatacaccaaggctttactcaaggggcttcattatattcacaagtttggatatgtccactgcgatattaagcttcaaaatattctcttgtgccccaatggcggcgtgaagattgctgatttcgggttggcgaagcgaggtgggggaaaaggcggtgtctccgggtgcgagctgaggggaacgcctatgtacatgtcgccggagatggtcgccggcagcgagcagggggctccggctgatatttgggcactcgggtgcgcagtgctggagatggtctccggatctccggcgtggagttgttccgatgtggcggcgctgctgatgagaattggagtcggtgaggagctgcctgagatccccgcgattttgtcggaggaagtcagagattttcttcaaaaatgttttgtcaaagatcccagacaaagatggacggctgagatgcttctgaatcatccgtttgttagcgccatagattttgatgataacaaaagcagcgtccctaccttgaaagacaggcacgaaaacacagcgtcaacgtctcccagatgcccattcgatttccaagattgggaatcatcgacaatatgttcaattgcatctttgtcctcatcggaggaatccggcttatggttctccgaggagtcgaattcgtggtccatttctgcggcggagcggctgcggacgctggtcagtgaacacagtcctaattggtctgtctccgacgattgggtcaccattagatgacagaaaagaatgtatagtcaattttgatcgcaagagtcagcatcagattttttaagattttcttccgcgcgacgctgattttttgattagatttttttactttatgaactttacctttattaaggtagtgaaaacaggctttttttatctgtttaattgtgtacattagatatgacgataatatatacaaattcaatggatgctgttatagttagcatctatctgacttgataaaagtattcgaattggagactgaaattatgagccgcagagtgtacagagcag

>comp29128_c0_seq1 sal1 phosphatase-like

agagagagagagagagagagatgagtgtatcaattgggcatgccccaatgacgagaggagcgttactgcctataaattccacgacaccgccccaggccaaatcaaaattcccatctctcttctcactctctctctatactaccccgcgcctgcgagctgtaagaaaaacagcggctgcttcaatgtcttacgacaaacaactcgccgctgccaagaaagctgcatctcttgctgctcgcttatgccagaaggtgcaaaaggctttgttgcaatctgatgtccaaacaaaatctgatagaagtcctgtcacagtggctgattatggttcacaggctattgttagctttattttggagaaagagttggcatcagttccattctcattagtagctgaagaggactctggagacctcaggaaagaagaatccagagaaacactgcatcgcataacagaacttgtcaatgatacacttgctagtgatgtaacaatagatatatcacctctctctgaggaagatgtgcttatagctatcgacaatggaaaatctgaagggggt

>comp29128_c1_seq1 sal1 phosphatase-like

cacatgactttcctatgcctgtcaggtacatgtcagtactactgaaaaccctgaagaggcatccttctttgaatcttttgaagctgcacattcttcccatgacttatctagcttgatagcagggaaacttggtgttaaagtgccacccgttaggattgatagccaagcaaagtatgctgctctatctagaggcgacggaacaatatatatgcggttccctcgcaaaggttacagagaaaagatatgggaccatgcagctggatacatagttgttgcagaagctggaggtgttgtcacagatgctggtgggaatcctctggacttctcaaagggaagatatcttgatctggacaccggaataattgttacgaatcagaaattgatgcctgttctcctgaaggctgttcaggagtccatcaaggagaaagcttcgtctttgtgattcagagactgaagcatatgtcaataagcaacaacagtcctgctaaacagacctagtatgaatgaggcagatttgttgttcacatgatttctgttttgctcatccccttaatggtaatagctttcttcagtaaaagaatctggaattgcagcaacatgcttaagacgaccctttctcaataaacaactcagttttcctggtccttctacaacattgtatataacttaatttgtagtactgctcaaacaatattgatgcttaaattgttttacctgtttctctgcaatatttggcatgtctctcagctggcagattaggcatatgaacttttgtttcacaccagtttctttttctcttttggcagcaagaatcgaatacgt

>comp29128_c1_seq2 sal1 phosphatase-like

gtcaacattgggttttggatccaattgatggcactaaaggatttctgagaggagatcaatatgttattgcattagcattattagataaggggaaagttgtattgggagtgttagcatgtccaaatcttcccttaacatctattgctagacgcactccaaacatttctgaagataaggctggttgtctttttttcgcccaagttgatgctggaacttatatgcaaagtctagatggctccctcccaacgaaggtacatgtcagtactactgaaaaccctgaagaggcatccttctttgaatcttttgaagctgcacattcttcccatgacttatctagcttgatagcagggaaacttggtgttaaagtgccacccgttaggattgatagccaagcaaagtatgctgctctatctagaggcgacggaacaatatatatgcggttccctcgcaaaggttacagagaaaagatatgggaccatgcagctggatacatagttgttgcagaagctggaggtgttgtcacagatgctggtgggaatcctctggacttctcaaagggaagatatcttgatctggacaccggaataattgttacgaatcagaaattgatgcctgttctcctgaaggctgttcaggagtccatcaaggagaaagcttcgtctttgtgattcagagactgaagcatatgtcaataagcaacaacagtcctgctaaacagacctagtatgaatgaggcagatttgttgttcacatgatttctgttttgctcatccccttaatggtaatagctttcttcagtaaaagaatctggaattgcagcaacatgcttaagacgaccctttctcaataaacaactcagttttcctggtccttctacaacattgtatataacttaatttgtagtactgctcaaacaatattgatgcttaaattgttttacctgtttctctgcaatatttggcatgtctctcagctggcagattaggcatatgaacttttgtttcacaccagtttctttttctcttttggcagcaagaatcgaatacgt

>comp29157_c1_seq1 at4g02380 t14p8_2

ggcaaaagaactgaaaataagcccaagtgctacaatcatattctctagaaaagagtaatcaacaataataaaacgaatgttcaagtttcatcaaaatgaaattaaatagcatgacacacacaatatatttttttgtaaaaataaaattgaaattctgagaattagctggtttattatgtcttcttgattctgtaaagtacctaggtaagcttccttcatcatctaagacaggacaggaaccatgtccaaagccttcaaaacaaaagtatatgtaaaggcatcttcttggagcttctcgaatcttcctgactttgaaaaatggcccgcacctccatactagtagaaacacagagctttccccaaattatttctgcataaataaatcccattttcttctaacaaaaaaaatcttccctgcggctgcggttttcgaatttttctccaattttcttccgagaaaaagaaaaagaaaaatggctcgctctttctccagcgtcaagaccgtctctgctttcatcgctaacgaaatctctgccgttgccgccaggaggggatactctgctgctgcatctcagggtgtcgtttcgagcagcgtgagaatcggagctccgaacgtgatgttgaagaaagggtccgaagaatccgccaagactgcatgggtgcccgacccggttaccgggtattaccgacccgagaacaagggtaaggagatcgatccggccgagctgagggagatgctcatcaagaataaaatgagacgaaactgaatcaaatcctccatgaaaatggggatttggaacccatttcggacgacccgtgattggatccgaattctttctgtcgttggttttctttctaggataacaatatggcggaaagctcgtggaatttaagaattcttttagcagaaactatagaaatttgccattgttggtgttgctcatcgtattctatgttttctcaggcgtttggcctgaggtggcggtggtgaagctattttctttaacgcttataataaaatataaattttatcatatgtgattgatgttcctatttattattcttgaacattttttatgttttcttgttgcgtgcggagaattttggggaaaggatgctctatttccaaatcagttaaatgtattttgaaagtcttatatttatattataaa

>comp29168_c0_seq1 indole-3-acetic acid-induced protein arg2-like

ataaacaactcttaccaaggtttcccatgaatcacaatctccacactttaattttctatatgtaataacaacagacttaggaaatagtggatttctatttctagctctcttaaaatttcaacaaaatatctgaaaattaccaaagcccaatcacagctgaaatttgattatggaaattctcttttattccagacaacattgtttcaaagaaagtaacagataacccccaaacaggggacacagcacagaggagctgcggctacaacaacagtggatatctcaacccgacttcttccgacggcccgattgtcgatggtgggatgtgccgtggccccccccccccaaaaaaaaaatcgggtaggatctaatctagtgtagtctagttttgttcttcacaagcatcgcgcgcagttcagcggcgtcgatttccttcgcgtgattctcgggtcggtagcacccggtgaccgggtcgggcacccatgaagtcttggcggactcttcggatcctttcttcaacatcacattcggagccccgcctctcgcgctgctcgatacgccgccctgtgttgcagctgcgtagcccctcctggagacaacatctgagattctgttggtgatgaaagcagagacagtcttagcattggagaaagagcgagccattttcttttatgtggatcgaaaggctatgtgtgatttaagagaaatttgaaaagcagctgcagaaaattttcgacactggaaaatctcctgcagcagccctctctcttcctctctctcttcaccctctccctctctctctacttt

>comp29296_c0_seq1 nac domain-containing protein 72-like

aaataatattcttgatttttcaagggggggaaaggaaaagctctttgttttctgctggatcaatttcaaaaggtgcaacccgaatcattatcatcattaaaatatgggcgtcggacaacccgatccatgttcccaattgagtctgccgccgggctttcggttcttcccgaccgacgaagagcttttggtgcagtatctatgcagaaaagtcgcaggccatcattttcctctgcaaatcattggagatgttgatttgtacaagtttgatccttgggatcttccaagtaagtattcatttagtttttcgatgatgtcgtgttatacacgtgactgacatcccctgatttcaggggatgaaacattgtttgcaacagaggcgactcgagtttctaaatttggaatctttggtgttgcaggcaaagctttgttcggagagaaagaatggtactttttcagcccgagggacagaaagtacccgaacggatcgaggccgaacagagtagccggctccggttattggaaagcgaccggcactgataagattattacaactgagggaagaaaagttgggatcaagaaagcacttgttttttacatcggaaaagctccgaaagggacgaaaactaattggattatgcatgagtacagactgtctgattctccgagaaaaaatggcggcaccaggttggacgattgggttctatgtagaatttacaagaagaactcgagcgggctgaatccgacccccacttctggcggccagagcaaagaatacagccacgactcgccgtcgtcgtgttcgtcgcaatacgacgacgtgctggagtctttgccagaactcgatcacaatttcttctcaatgccaagaatggattctctcaagaattttcagcaggaagataatcacaagttgaatctccagcgcttggggtcagacaaccttgattgggccacgctagctgggcttaacccgatacccgaactcggacaacagcaaccgggtctgacccagcagccaca

>comp29296_c0_seq2 nac domain-containing protein 72-like

aatttttcctcgaccgcgaacaagggaaaaaacggatttcttgcctttgtctgcaaattagttgaattaaggtatttgctcagaaaaaatgggcgtcagggaaaccgacccgctcttacaattgagtttgcctccgggctttcggttctatcccacggatgaggagctcttggtgcagtacctctgcaggaaagttgcaggccaccatttttctttacaaattattggggatattgatttgtacaaacatgatccttgggatcttcccagcaaagctatatttggagagaaagaatggtattttttcaccccaagagacagaaagtatccgaatggatcgaggccgaacagagtggcagggtctggatattggaaagccaccgggactgataaggttattacaacggagggtagaaaagttggtataaagaaagcccttgtcttttacattgggaaagcacctaaaggaaccaaaacaaactggattatgcatgaataccgactttctgaatctcccagaaaaaatggcagcgctaggttagatgattgggtgctttgccggatttacaagaaaaattcaagtgcacagaagcctgccatttctggtgtccagagcaaagactacagtctcggctcatcgtcatcgtcttcgtctcaatatgacgacgtgctcgaatcattgccggccattgatgaccgtttcttctctttgccaaaaatgaattctctcaagaatttccatgagcaggaagaccaaaagctgaatcttcagcacttgggttctgggaatttcgactgggccacgctagctgggcttaacccgatacccgaactcggacaacagcaaccgggtctgacccagcagccaca

>comp29296_c0_seq3 nac domain-containing protein 72-like

aaataatattcttgatttttcaagggggggaaaggaaaagctctttgttttctgctggatcaatttcaaaaggtgcaacccgaatcattatcatcattaaaatatgggcgtcggacaacccgatccatgttcccaattgagtctgccgccgggctttcggttcttcccgaccgacgaagagcttttggtgcagtatctatgcagaaaagtcgcaggccatcattttcctctgcaaatcattggagatgttgatttgtacaagtttgatccttgggatcttccaagcaaagctttgttcggagagaaagaatggtactttttcagcccgagggacagaaagtacccgaacggatcgaggccgaacagagtagccggctccggttattggaaagcgaccggcactgataagattattacaactgagggaagaaaagttgggatcaagaaagcacttgttttttacatcggaaaagctccgaaagggacgaaaactaattggattatgcatgagtacagactgtctgattctccgagaaaaaatggcggcaccaggttggacgattgggttctatgtagaatttacaagaagaactcgagcgggctgaatccgacccccacttctggcggccagagcaaagaatacagccacgactcgccgtcgtcgtgttcgtcgcaatacgacgacgtgctggagtctttgccagaactcgatcacaatttcttctcaatgccaagaatggattctctcaagaattttcagcaggaagataatcacaagttgaatctccagcgcttggggtcagacaaccttgattgggccacgctagctgggcttaacccgatacccgaactcggacaacagcaaccgggtctgacccagcagccaca

>comp29307_c1_seq1 cbl-interacting protein kinase 6

agcattcaaagtctctgtttccttgccctttccaacgttcgcctcatcttccacggtgaattcttgctcgtccttagtcctcaaacttctaggggtcgatttcttgaaccagggtgagtccatgatcctcactatgcttattctcgtgcttggattgggatccaacattttcgtgattaatttccgggagtcagaagacaaccacggcgggcacttgaaatctcccctgtaaatcttccgatacagggaaacaatgttatcatcctgaaaaggcaaataacccgccaacaacacaaaaag

>comp29307_c2_seq1 cbl-interacting protein kinase 6

agactttgaatgctttccacaccatttcgttgtctgaaggatttgatctgtcgccactcttcgaggagaagaagagggtggaaaaagaagaattgagattcgccacaacaaggccagcaagcagcgtgatttcgaagattgaagaggtggcgaagacgaagaatttcagcgtgaagaagagcgattcatgtgtaaggctgcaggggcaacagaacgggagaaaagggaagctgggaatagctgccgatatatttgccgtaacgccatcgtttctggtagtgcaggtgaagaaatgcagtggagatactcttgagtacaatcagttctgcagcaaagagcttcggcccgcgctgaaagatatcgaatggacctcaacaggtgggagttcaatggctgtttgaagtgtgtgacaaaattcctcaacaaatttatggcggatgtttgaattcatgatcattggattgagcaactgttgttgtttttgatcaggatatgttattcttggattgtttgttttgatttgagagacctaatgtgagagtctcggaattgaatttggtacaatttcaattgtcatcttgttttgtgtgtgtgtgtgaaaatggatattctgtttcagcaatgttgtgtccattgtttctctgtaaaaagatctctctgattcacagtatctgtgcaagtggatgatttagttttgtgtaaaagggatcattctgtttt

>comp29307_c3_seq2 protein kinase

catgaccaaatatcggccttcgccccatcatacccctttttcccaatcacctccggagcgacatacgccggcgtcccacacgtcgtgtgcagcaaaccgtcctgcctgagatgatctgaaaacgcactcaacccaaaatccgcaaccttaagattaccttcctcgtccaacaacagattttccggcttcagatcacgatgataaacaccgcggctatggcaaaaatcaatcgccgatatcaattgctggaaataattccgagctgcttcctcgcgtaatcggcccttggcaattttcgcaaatagttcgccgccacggactaattccatggcgaaatagatcttgcctttgctggccattacctcgtggagctcgacgatgttgggatgatgcaccattttcatgacggagatc

>comp29333_c1_seq1 e3 ubiquitin-protein ligase chip-like

tgctgaagatgatactccaactgacgtgccagactatctgtgctgtaagatcactttagatatttttcgtgatcctgtaattactccaagtggggttacatatgagagagcagtgatcctacaccatctacagaaggtgggtaaatttgatccgatcacccgtgaaccgctatatccatctcagttggtaccaaatttggccataaaagaag

>comp29333_c2_seq3 e3 ubiquitin-protein ligase chip-like

cgcccgccctcaaacacaccatttctgttttcagtctaacacattattctacgtataacatacattcttttgtgtgtgtctgtgcgtgtttttgtgtgtgtgtatatatatattagacatatagagagagaaggcgtaaagaatagggagaggggagaaaaggaaaatgtcgccaacagtggcggcggcgaaacaggcggagcagctgagacaggacggcaacacctacttcaaaaaagatcggttaggggccgccattgatgcttatacagaggcgattgctttgtgccctaatgttccggtttattggacgaatcgggctctctgtcatcgcaagaggaatgaatggacgcgagtggaggaggattgcaggagagcaattcagcttgatcaccattctgtaaaggcccactatatgcttggtcttgcactgctacaaaggaaagaatatggggaaggtgtcaaggaattggagaaggcattggaccttgggacgggtgcaaaccccaagggttacatggtggaggagatatggcaagagcttgcaagagcaaaatacttagcatgggaacatgaatctagcgagcgttcttgggaacttcaaaacttaaaagaagcatgtgaagcagccctgaaggagaaatgtagccgtgatgctcctcgaacagaaggttttgtagatgaaactgccaagtctaatttagatcagttggaagctttgggccaagtattcaagaaagctgctgaagatgatactcca

>comp29346_c1_seq2 heat stress transcription factor a-4a-like isoform x1

aggggcagaattcatgtccattaaagatgcattgcccccaatatcaacatttagttgggtgtaagatatgcaggggctgggcacacaatttgtagattcaaccatgtccaaggatgaattacattgcactaaaccttcaccaacatcaagtactattttctcccagaacattatagaagactctatctgcagcaattccttgttcaaagtcagaagtggatcagcatctaaattttcaattgatgaattttgtgaagattgcctaagattatcttctgtgctggtttcatcgcataagtaactattgccaggaaatcttctctttctatcttgaacttccgcttgtggcatgagatccaaagaaaaagcaggttggcgtaatgtctcagctaaggaggttaacatatttgcatgtcggtgttccacattttgaacgcgttcagtcaaagcccccatttgcattttaagagcttcttcctcctctttgtgcctttgcagttccacattaagtgattctttttcatgcttcagtttatcgatatcatccttatatcctttcctttcctgttcagttaggggaggtagcgagcccagattcagcacggagtgactgtgaacaggcttgcgcctatggatattctttaaaagctgcggctgacctctaacaaaatcttcatttgcaaattcccattgttcaggatcaacttttctgaaaccatatgtattcagctgcctaataaagctggagaaattgttgtgcttaaagaatcttggcagaagctccctggaaaattctggaggattccacacaacaaaacttttattaccctgactccaggatacaattgagtcggtggatggatcgtccaccatctcatacgtcttggcaaggaaaggaggcaatgagttagagctgcacgaagcttcatccatcatggcaaatttatctcacaaacacagatgccctaaaattatcacctgaagatatcaagtagaaaaccaaatcaacaaaccccttaaggttttaacagcctccacatcactgatttgtcttcaggct
[truncated: 498,425 more chars]
