# Supplementary material for: Physiological and molecular responses to drought stress in teak (Tectona grandis L.f.)
Source: PLoS One. 2019 Sep 9;14(9):e0221571. doi: 10.1371/journal.pone.0221571 (PMC6733471; doi:10.1371/journal.pone.0221571)
Supplement: S13 File — Number of metabolic maps of the KEGG platform for Root Library transcripts referenced for water stress. "S" is the number of sequences assigned to each map, and "E" is the number of localized Ecs. The five metabolisms with the highest number of transcripts are highlighted in grey. (DOCX) [file pone.0221571.s013.docx]

**S13 File. Metabolic pathways using KEGG.** Number of metabolic maps of the KEGG platform for Root Library transcripts referenced for water stress. "S" is the number of sequences assigned to each map, and "E" is the number of localized Ecs. The five metabolisms with the highest number of transcripts are highlighted in grey.

| **Nº** | **Name** | **S** | **E** | |
| --- | --- | --- | --- | --- |
| 1 | Glycerolipid metabolism | 30 | | 8 |
| 2 | Phenylalanine metabolism | 24 | | 3 |
| 3 | T cell receptor signaling pathway | 24 | | 2 |
| 4 | Drug metabolism - cytochrome P450 | 24 | | 2 |
| 5 | Metabolism of xenobiotics by cytochrome P450 | 24 | | 2 |
| 6 | Starch and sucrose metabolism | 24 | | 4 |
| 7 | Galactose metabolism | 23 | | 6 |
| 8 | Glutathione metabolism | 19 | | 3 |
| 9 | Pyruvate metabolism | 18 | | 3 |
| 10 | Glycolysis / Gluconeogenesis | 17 | | 6 |
| 11 | beta-Alanine metabolism | 17 | | 3 |
| 12 | Pentose and glucuronate interconversions | 16 | | 3 |
| 13 | Amino sugar and nucleotide sugar metabolism | 16 | | 6 |
| 14 | Carotenoid biosynthesis | 16 | | 3 |
| 15 | Tyrosine metabolism | 16 | | 2 |
| 16 | Ether lipid metabolism | 14 | | 2 |
| 17 | Glycerophospholipid metabolism | 14 | | 2 |
| 18 | Glycine, serine and threonine metabolism | 13 | | 4 |
| 19 | Arginine and proline metabolism | 12 | | 2 |
| 20 | Oxidative phosphorylation | 12 | | 2 |
| 21 | Fatty acid degradation | 11 | | 2 |
| 22 | mTOR signaling pathway | 11 | | 1 |
| 23 | Tryptophan metabolism | 11 | | 2 |
| 24 | Drug metabolism - other enzymes | 11 | | 2 |
| 25 | Chloroalkane and chloroalkene degradation | 10 | | 1 |

| **Nº** | **Name** | **S** | | **E** |
| --- | --- | --- | --- | --- |
| 26 | Purine metabolism | 10 | 5 | |
| 27 | Limonene and pinene degradation | 10 | 1 | |
| 28 | Valine, leucine and isoleucine degradation | 10 | 1 | |
| 29 | Lysine degradation | 10 | 1 | |
| 30 | Ascorbate and aldarate metabolism | 10 | 1 | |
| 31 | Histidine metabolism | 10 | 2 | |
| 32 | Phenylpropanoid biosynthesis | 9 | 2 | |
| 33 | Selenocompound metabolism | 9 | 2 | |
| 34 | Fructose and mannose metabolism | 8 | 2 | |
| 35 | Tropane, piperidine and pyridine alkaloid biosynthesis | 7 | 1 | |
| 36 | Isoquinoline alkaloid biosynthesis | 7 | 1 | |
| 37 | Pentose phosphate pathway | 6 | 3 | |
| 38 | Cysteine and methionine metabolism | 5 | 3 | |
| 39 | Methane metabolism | 4 | 2 | |
| 40 | Riboflavin metabolism | 4 | 2 | |
| 41 | Caprolactam degradation | 4 | 2 | |
| 42 | Sulfur metabolism | 4 | 2 | |
| 43 | Phosphatidylinositol signaling system | 4 | 2 | |
| 44 | Inositol phosphate metabolism | 4 | 2 | |
| 45 | Sesquiterpenoid and triterpenoid biosynthesis | 3 | 2 | |
| 46 | Steroid biosynthesis | 3 | 2 | |
| 47 | Terpenoid backbone biosynthesis | 3 | 2 | |
| 48 | Thiamine metabolism | 3 | 1 | |
| 49 | Novobiocin biosynthesis | 3 | 1 | |
| 50 | Phenylalanine, tyrosine and tryptophan biosynthesis | 3 | 2 | |
| 51 | Biotin metabolism | 2 | 1 | |
| 52 | Streptomycin biosynthesis | 2 | 1 | |

| **Nº** | **Name** | **S** | **E** |
| --- | --- | --- | --- |
| 53 | Caffeine metabolism | 2 | 1 |
| 54 | Fatty acid biosynthesis | 2 | 1 |
| 55 | Biosynthesis of unsaturated fatty acids | 2 | 1 |
| 56 | Glyoxylate and dicarboxylate metabolism | 2 | 2 |
| 57 | alpha-Linolenic acid metabolism | 2 | 1 |
| 58 | Arachidonic acid metabolism | 2 | 1 |
| 59 | Carbon fixation in photosynthetic organisms | 1 | 1 |
| 60 | Glucosinolate biosynthesis | 1 | 1 |
| 61 | Retinol metabolism | 1 | 1 |
| 62 | Sphingolipid metabolism | 1 | 1 |
| 63 | Citrate cycle (TCA cycle) | 1 | 1 |
