# Supplementary material for: Physiological and molecular responses to drought stress in teak (Tectona grandis L.f.)
Source: PLoS One. 2019 Sep 9;14(9):e0221571. doi: 10.1371/journal.pone.0221571 (PMC6733471; doi:10.1371/journal.pone.0221571)
Supplement: S14 File — Genes related with drought stress used in this study and submitted to the NCBI. (DOCX) [file pone.0221571.s014.docx]

**S14 File. Gene submission to NCBI.** Genes related with drought stress used in this study and submitted to the NCBI.

- *TgAREB1* corresponds to name “**comp32065_c11_seq6**” in the root transcritome
- *TgDREB1* corresponds to name “**comp1924_c0_seq1**” in the root transcritome
- *TgPIP1* corresponds to name “**comp30227_c3_seq10**” in the root transcritome
- *TgTPS1* corresponds to name “**comp49883_c0_seq1**” in the root transcritome

>TgAREB1 (Accession number MH003887.1)

atgtcttatgcagcagagttgtgtgatagcggtctagtgggagacatgaaagttatgacaagttacacttcagaagctagatctcatttgagacaaggtaatgtagtgtgtgctctaagaaacatgcagctgcaattaataaacaaaatgagttttagctacaatgacgaaggctttaatattgttttctgcttaaaattttctgataggatgaaatgccctgaactagttctgtttttttttggattcttccaggcatacactatggagttggaagcagaagttgcaaaactgaaagaggaaaatgaacaattgcagaagaaacaggaagaaataatggaaatgcagaagaatcaggttctggagatgatgaaacagcagaatggggccaagaggcaatgcttgaggaggacgcagacgggtccatgggcggtgcattcaatgactgaacatatgttgaacgatatatgttgtaaatacatagagacttggtgctggtgtagatcaatagaaattagagtagtagcgctccattatagaattttccttctttttgatggtttcccacgagagggatatagctgcaacgtttcactgcacccccagcttctgagcacgatagatagattgcctgaagttaaggcagttgatgtacctgttgagtacagagatttgaagatatatttttcagttcaaagtgtcgtgcttttgcagttatgtaacactagtattcttgttcgtgtgggcataacgtag

>TgAREB1 (Accession number AWW87323.1)

MSYAAELCDSGLVGDMKVMTSYTSEARSHLRQGNVVCALRNMQLQLINKMSFSYNDEGFNIVFCLKFSDRMKCPELVLFFFGFFQAYTMELEAEVAKLKEENEQLQKKQEEIMEMQKNQVLEMMKQQNGAKRQCLRRTQTGPWAVHSMTEHMLNDICCKYIETWCWCRSIEIRVVALHYRIFLLFDGFPREGYSCNVSLHPQLLSTIDRLPEVKAVDVPVEYRDLKIYFSVQSVVLLQLCNTSILVRVGIT

>TgDREB1 (Accession number MH003888.1)

atgtatggatcttgtgcccggcttaatttccccagttatggtccatctgcaaaatgcactaatgactcctcttcgctgcttgctacatcaactgactcgacaaactctagcatctcagaggtatgttgtagtgatgagggaccaaattctgatgttcccaagataaaaactgaagaggctgaaggtttgtcacggatcattgacaataggcatgctgcgtcgcatgaagctggcaccccgatgagtgtagtcaaagaggaagtaatagaagaagctccaagggagaaagtgcaggaagaagcaatgaaggaggaaggtaagacagagtcgatggatcccttgtatggtggtgatgctgagaagaagcgtactgcggagaaaccttatataagccaccagagtgctgacgctgggcaac

>TgDREB1 (Accession number AWW87324.1)

MYGSCARLNFPSYGPSAKCTNDSSSLLATSTDSTNSSISEVCCSDEGPNSDVPKIKTEEAEGLSRIIDNRHAASHEAGTPMSVVKEEVIEEAPREKVQEEAMKEEGKTESMDPLYGGDAEKKRTAEKPYISHQSADAGQ

>TgPIP1 (Accession number MH003889.1)

atggcagaaagcaaggaggaagatgttaagcttggagcaaacaagtttacagagaggcagccattgggcacagctgctcaatcagacaaagattacaaggagccaccaccagctcctttgtttgaaccaggagagctgaaatcatggtccttttacagggctgggattgcagaatttatagctacattcttgttcttgtacatcactatcttgactgtgatgggggttggtagagctaagaacaagtgtgcctctgtgggtattcaggggattgcttgggcttttggtggcatgatcttcgcacttgtttactgcactgcgggaatttcagggggacacataaatcctgccgtaacatttgggctgttcttggcgaggaaattgtcgttgacgcgggcggtgttctacatggtgatgcaatgcctaggagccatctgtggtgccggagtggtgaagggcttcggcaaaaccctgtatcagaccaagggtggtggtgcaaatgtggtcaaccacggctacaccaagggcgacggccttggggctgaaatcgtcggcactttcgtgctcgtttacaccgtcttttctgccacggacgcgaagcgcagcgccagagactcccatgtccctatattggcaccattgccaattgggtttgctgtgttcttggtgcatttggcaactattccaataactggtactggtattaacccagccaggagtcttggagcagcaatcatctacaacaaggaccatgcttgggatgatcattggatattctgggttggaccattcattggtgcagcactagcagccctgtaccaccaggtggtgatcagggccattccattcaagtccaggtga

>TgPIP1 (Accession number AWW87325.1)

MAESKEEDVKLGANKFTERQPLGTAAQSDKDYKEPPPAPLFEPGELKSWSFYRAGIAEFIATFLFLYITILTVMGVGRAKNKCASVGIQGIAWAFGGMIFALVYCTAGISGGHINPAVTFGLFLARKLSLTRAVFYMVMQCLGAICGAGVVKGFGKTLYQTKGGGANVVNHGYTKGDGLGAEIVGTFVLVYTVFSATDAKRSARDSHVPILAPLPIGFAVFLVHLATIPITGTGINPARSLGAAIIYNKDHAWDDHWIFWVGPFIGAALAALYHQVVIRAIPFKSR

>TgTPS1 (Accession number MH003890.1)

atgggtcaactttgctttgtgctggatcttccagagacagaacgtaaggtggtgcaattgcgggatagatttcagggtcagactgtcttgcttggggtggatgacatggatattttcaaaggaataagcttgaaacttctggcctttgagcaattgcttactctccatcctagaaaaagaggtaaagtggttttggttcagatagctaatcctgccagaggtcgagggaaagatgtacaagaggttcaatctgaaactcacgctattgttgcaagaattaaccgtctctttggaaaaccaggatatgagccagtagttttgattgatacctcacttcacttctacgagcgtattgcgtactatgtcattgctgagtgttgtcttgtcactgcagtaagagatgggatgaatttaataccttatgaatacattgtatgcagacaaggaactgataagctagatcaaacattgaacttgaacccatcatcaccgaagaagagcatgttggtggtttctgaatttattgggtgctcaccatctcttagtggtgcaattagggtcaatccttggaacattgatgccgttgtggaagctatggattctgctttcatggtgcctgagccagagaaacaactgcgtcatgaaaagcactacaagtatgtaagtagtcatgatgttgcctattgggcccacagcttttttgttgatcttgaaagagcatgtagagatcacataaggagaagatgctggggtattgggtttgggttaggatttcgtgttattgccttggatccgagtttcaggaagctgtcagttgaacacatagtatctgcatataaaaggacaaagaaccgtgcaattcttttggactatgatggcacaatgacatctcagaatgctgctgacacaagccctaatgctgaggctattggaattctaaataaactgtgcagagaccccaagaatgtagtttttgttgtaagtgggaaaaataaagagatcttaacacgatggttttcttcctgcgaaaacctaggcattggagcagagcatggatattttgtgaggccacaacacagtgcagactgggaaacttgttttgccgttcaagatttttactggaaacagattgccgaacctgtaatgcagttatatacagaaacaactgatggttctttcatagaaaccaaagagagtgcacttgtctggaattaccaacttgcagatccagattttgggtcatgtcaggctaaggagcttcatgatcacctggagagtgttctggcaaatgaacctgtttcagtcaagagtggccagcacattgtagaagtaaagcctcagggggtcaacaaaggacttgtagcggaaagacttctagcaatgatgcgtcagaaggaagtgcttcctgatttcgtgctttgtataggggatgaccggtctgatgaggacatgtttgaggcgataatgagtgccagggacgacggttctctctccccggttgctgaagtgtttgcttgcacagttggacagaagcctagcaaggccaagtactatttggaagatacgtcggagatactgagaatgtttcaaagccttgctgatgcctctgagcagtcttcaagaaccgtctcgttttctccacaacgggtggtcatcaataaagcatga

>TgTPS1 (Accession number AWW87326.1)

MGQLCFVLDLPETERKVVQLRDRFQGQTVLLGVDDMDIFKGISLKLLAFEQLLTLHPRKRGKVVLVQIANPARGRGKDVQEVQSETHAIVARINRLFGKPGYEPVVLIDTSLHFYERIAYYVIAECCLVTAVRDGMNLIPYEYIVCRQGTDKLDQTLNLNPSSPKKSMLVVSEFIGCSPSLSGAIRVNPWNIDAVVEAMDSAFMVPEPEKQLRHEKHYKYVSSHDVAYWAHSFFVDLERACRDHIRRRCWGIGFGLGFRVIALDPSFRKLSVEHIVSAYKRTKNRAILLDYDGTMTSQNAADTSPNAEAIGILNKLCRDPKNVVFVVSGKNKEILTRWFSSCENLGIGAEHGYFVRPQHSADWETCFAVQDFYWKQIAEPVMQLYTETTDGSFIETKESALVWNYQLADPDFGSCQAKEL

HDHLESVLANEPVSVKSGQHIVEVKPQGVNKGLVAERLLAMMRQKEVLPDFVLCIGDDRSDEDMFEAIMSARDDGSLSPVAEVFACTVGQKPSKAKYYLEDTSEILRMFQSLADASEQSSRTVSFSPQRVVINKA
