# Supplementary material for: Physiological and molecular responses to drought stress in teak (Tectona grandis L.f.)
Source: PLoS One. 2019 Sep 9;14(9):e0221571. doi: 10.1371/journal.pone.0221571 (PMC6733471; doi:10.1371/journal.pone.0221571)

**S15 File. Alignments of drought stress genes.** Blastx and Alignment using Clustal Omega for TgTPS1, TgPIP1, TgAREB1 and TgDREB1 genes.

Main results of the alignment of the *TgTPS1* gene through Blastx:

| Description | E-value | Identity | Access |
| --- | --- | --- | --- |
| Glycosyl transferase family 20 family protein [*Populus trichocarpa*] | 0 | 81% | XP_002304347.1 |
| Alpha,alpha-trehalose-phosphate synthase [UDP-forming] 5 [*Morus notabilis*] | 0 | 80% | EXB66534.1 |
| Trehalose-6-phosphate synthase, putative [*Ricinus communis*] | 0 | 82% | XP_002527658.1 |
| Trehalose phosphatase/synthase 5 isoform 1 [*Theobroma cacao*] | 0 | 80% | XP_007040643.1 |
| Trehalose-6-phosphate synthase domain protein [*Medicago truncatula*] | 0 | 78% | KEH44193.1 |
| Putative trehalose phosphatase/synthase 5 [*Arabidopsis thaliana*] | 0 | 75% | NP_567538.1 |
| Trehalose-6-phosphate synthase like protein [*Arabidopsis thaliana*] | 0 | 75% | CAB10557.1 |
| Trehalose-6-phosphate synthase  [*Ginkgo biloba*] | 0 | 74% | AAX16014.1 |
| Trehalose-6-phosphate synthase, putative [*Brassica oleracea*] | 0 | 74% | ABD65165.1 |

**Alignment of the TPS enzyme protein sequence, indicating the Glycosyltransferase domain (green rectangle) and percentage of identity (blue tones)**


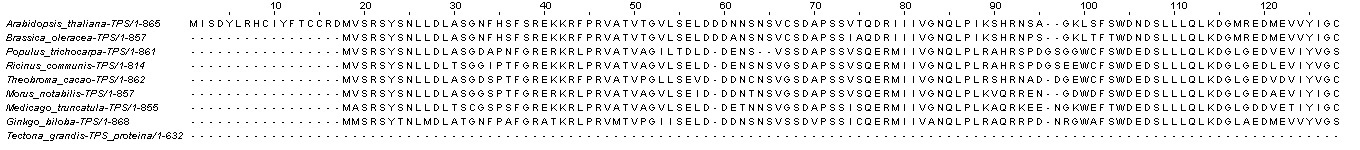


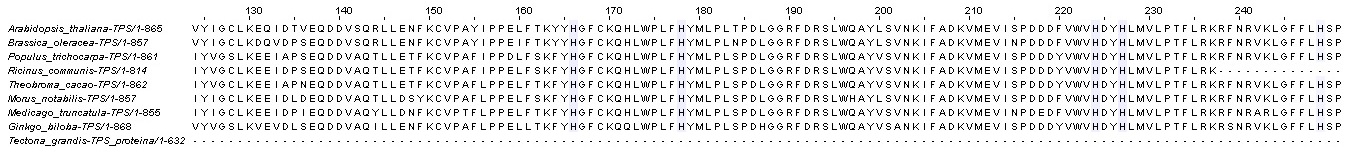


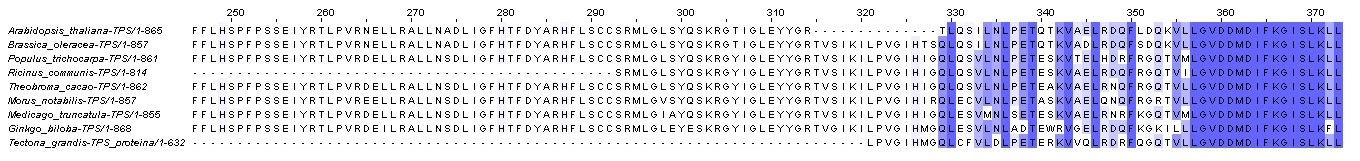


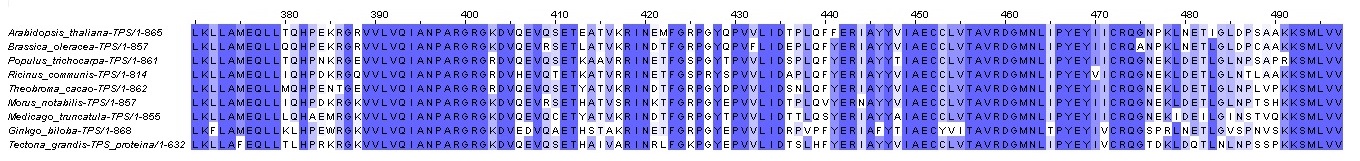


**Alignment of the TPS enzyme protein sequence, indicating the domain Glycosyltransferase (green rectangle), domain Trehalose-phosphatase (orange rectangle) and percentage of identity (blue tones)**


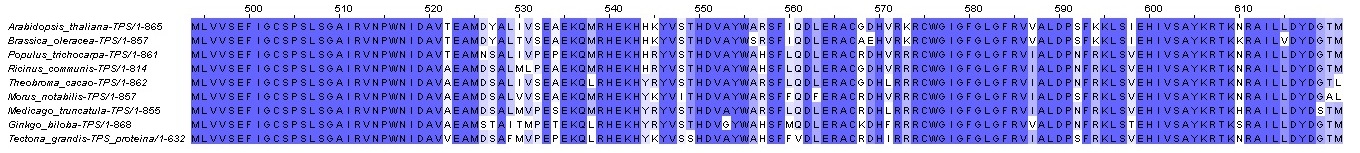


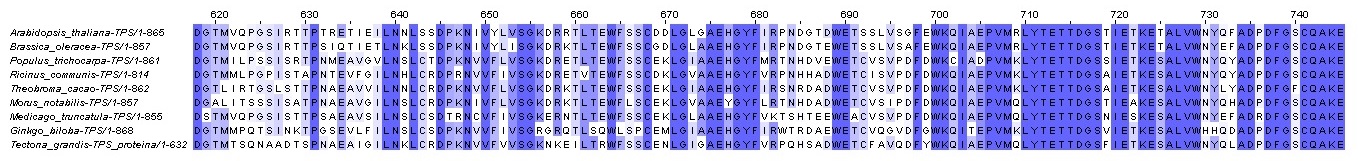


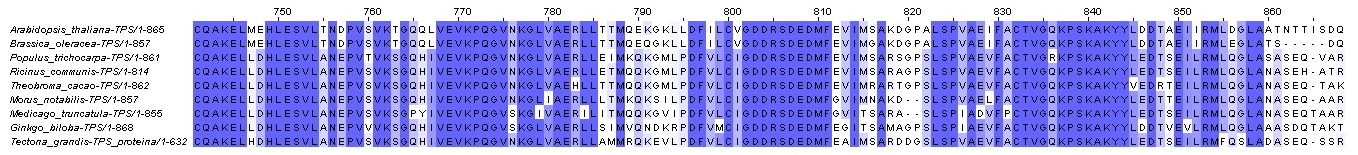


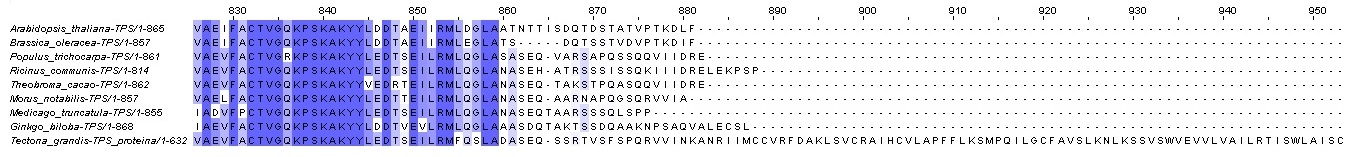


**Main results of the alignment of the *TgPIP1* gene through Blastx:**

| Description | E-value | Identity | Access |
| --- | --- | --- | --- |
| Water channel protein  [*Nicotiana excelsior*] | 0 | 93% | BAA20074.1 |
| Aquaporin PIP1;3  [*Quercus petraea*] | 0 | 93% | AFH36341.1 |
| Aquaporin  [*Iris x hollandica*] | 0 | 92% | BAF44223.1 |
| Aquaporin protein 7  [*Camellia japonica*] | 0 | 92% | AHI54567.1 |
| Plasma membrane intrinsic protein 1B  [*Theobroma cacao*] | 0 | 91% | XP_007051783.1 |
| Aquaporin 1  [*Gossypium hirsutum*] | 0 | 90% | ABD63904.1 |
| Plasma membrane intrinsic protein  [*Olea europaea*] | 0 | 93% | ABB13429.1 |
| Plasma membrane intrinsic protein 1;4  [*Arabidopsis thaliana*] | 0 | 92% | NP_567178.1 |
| Plasma membrane aquaporin 1  [*Hevea brasiliensis*] | 0 | 90% | ACV66985.1 |

**Alignment of the PIP protein sequence, indicating the Major Intrinsic Protein domain (pink rectangle) and the percentage of identity (blue tones)**


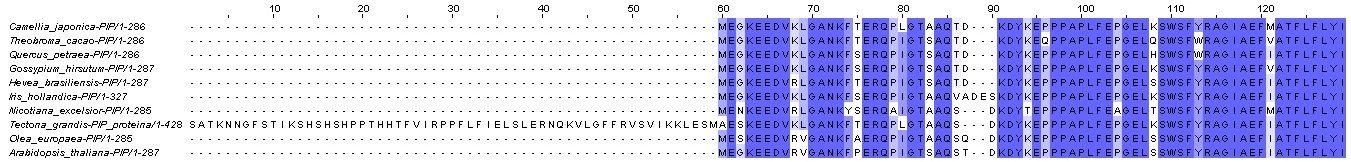


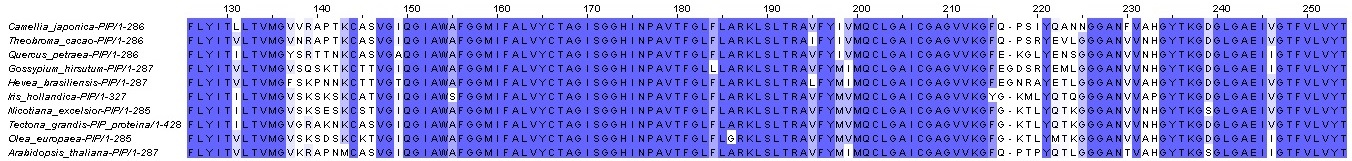


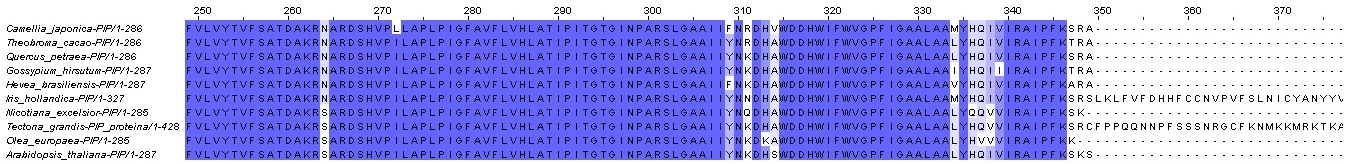


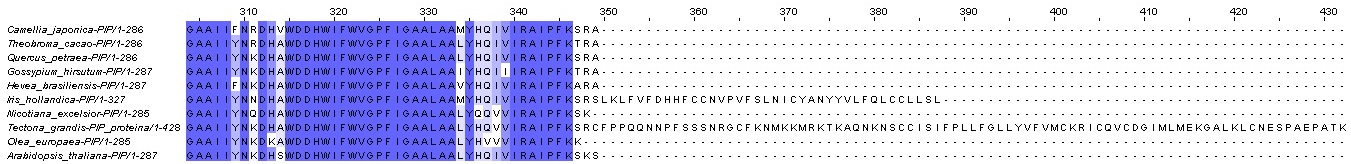


Main results of the alignment of the *TgAREB1* gene through Blastx:

| Description | E-value | Identity | Access |
| --- | --- | --- | --- |
| AREB-like protein  [*Solanum lycopersicum*] | 8.0e-07 | 85% | NP_001234596.1 |
| ABA responsive element-binding protein  [*Solanum torvum*] | 1e-06 | 86% | AFA37978.1 |
| AREB-like protein  [*Solanum nigrum*] | 1e-06 | 85% | AHA43415.1 |
| Abscisic acid responsive element-binding protein 2  [*Populus suaveolens*] | 1e-06 | 83% | ABF29696.1 |
| Abscisic acid responsive elements-binding factor 2 isoform 1  [*Theobroma cacao*] | 2e-06 | 80% | XP_007017213.1 |
| ABA-responsive element binding factor 4  [*Brassica napus*] | 0.003 | 69% | AGG35957.1 |
| Abscisic acid responsive element-binding factor 1 family protein  [*Populus trichocarpa*] | 0.003 | 69% | XP_006384405.1 |
| Abscisic acid responsive elements-binding factor 3  [*Arabidopsis thaliana*] | 0.010 | 74% | NP_567949.1 |
| ABA-responsive element binding protein 1  [*Beta vulgaris* subsp. *vulgaris*] | 0.001 | 73% | CAP66259.1 |

**Alignment of the protein sequence of the AREB transcription factor, indicating the bZIP (red rectangle) domain and the percentage of identity (blue tones)**


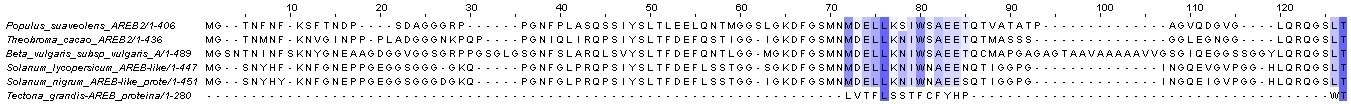


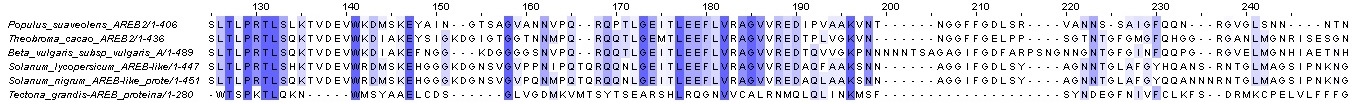


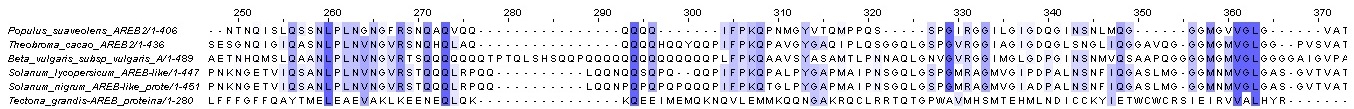


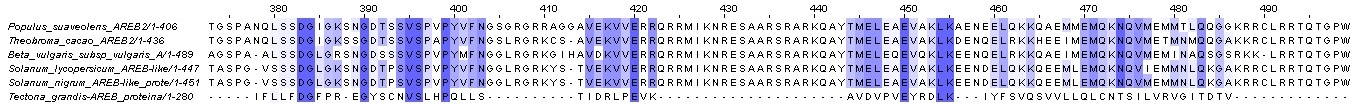


Main results of the alignment of the *TgTPS1* gene through Blastx:

| Description | E-value | Identity | Access |
| --- | --- | --- | --- |
| DREB2  [*Solanum lycopersicum*] | 2e-22 | 49% | ADZ15315.1 |
| dehydration responsive element binding transcription factor  [*Morus notabilis*] | 3e-22 | 51% | AHJ25962.1 |
| DREB2A  [*Salicornia brachiata*] | 6e-20 | 49% | ADD92167.1 |
| DREB2-like protein  [*Chrysanthemum vestitum*] | 5e-19 | 88% | ABR23508.1 |
| dehydration responsive element binding protein 2A  [*Vigna unguiculata*] | 6e-19 | 90% | AEO50757.2 |
| DRE-binding protein 2  [*Leymus qinghaicus*] | 1e-18 | 76% | AFO12477.1 |
| DRE-binding protein 2  [*Populus trichocarpa*] | 2e-18 | 89% | ABO48361.1 |
| DRE-binding protein 2A;2  [*Glycine max*] | 3e-18 | 89% | AFU35563.1 |
| DREB2A  [*Arabidopsis thaliana*] | 4e-17 | 84% | AAU93685.1 |

**Alignment of the DREB transcription factor protein sequence, indicating the AP2 domain (purple rectangle) and the percentage of identity (shades of blue)**


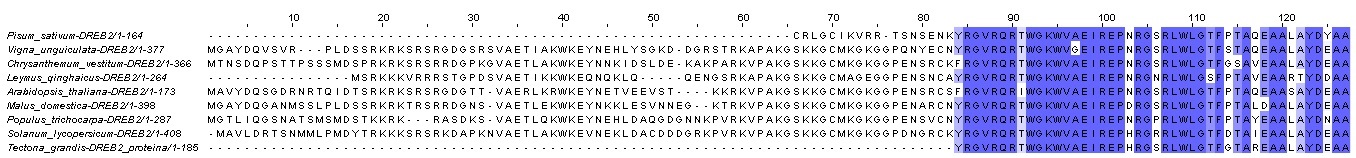


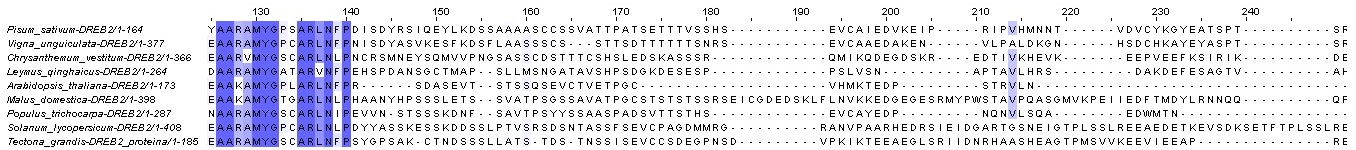


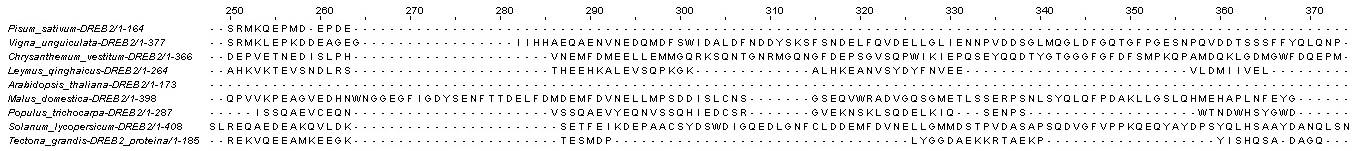


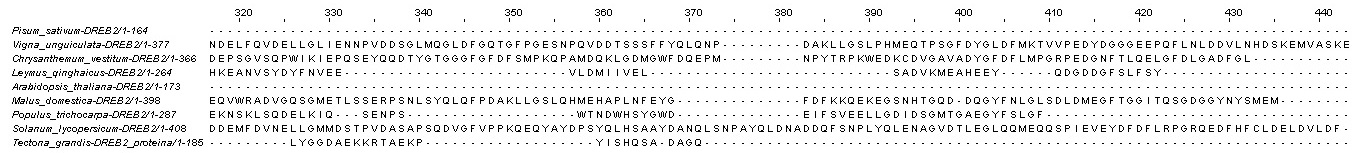

Supplement: S15 File — Blastx and Alignment using Clustal Omega for TgTPS1, TgPIP1, TgAREB1 and TgDREB1 genes. (DOCX) [file pone.0221571.s015.docx]
